# Porphyrin-fused graphene nanoribbons

**DOI:** 10.1038/s41557-024-01477-1

**Published:** 2024-03-08

**Authors:** Qiang Chen, Alessandro Lodi, Heng Zhang, Alex Gee, Hai I. Wang, Fanmiao Kong, Michael Clarke, Matthew Edmondson, Jack Hart, James N. O’Shea, Wojciech Stawski, Jonathan Baugh, Akimitsu Narita, Alex Saywell, Mischa Bonn, Klaus Müllen, Lapo Bogani, Harry L. Anderson

**Affiliations:** 1 Department of Chemistry, University of Oxford, Chemistry Research Laboratory, Oxford, UK; 2 Max Planck Institute for Polymer Research, Mainz, Germany; 3 Department of Materials, University of Oxford, Oxford, UK; 4 School of Physics & Astronomy, University of Nottingham, Nottingham, UK; 5 Institute for Quantum Computing, University of Waterloo, Waterloo, Ontario Canada; 6 Present Address: Institute of Functional Nano & Soft Materials (FUNSOM), Soochow University, Suzhou, China; 7 Present Address: Nanophotonics, Debye Institute for Nanomaterials Research, Utrecht University, Utrecht, the Netherlands; 8 Present Address: Department of Chemistry & Physics, University of Florence, Sesto Fiorentino, Italy

**Keywords:** Electronic materials, Electronic properties and materials, Synthetic chemistry methodology, Molecular electronics, Polymer synthesis

## Abstract

Graphene nanoribbons (GNRs), nanometre-wide strips of graphene, are promising materials for fabricating electronic devices. Many GNRs have been reported, yet no scalable strategies are known for synthesizing GNRs with metal atoms and heteroaromatic units at precisely defined positions in the conjugated backbone, which would be valuable for tuning their optical, electronic and magnetic properties. Here we report the solution-phase synthesis of a porphyrin-fused graphene nanoribbon (PGNR). This PGNR has metalloporphyrins fused into a twisted fjord-edged GNR backbone; it consists of long chains (>100 nm), with a narrow optical bandgap (~1.0 eV) and high local charge mobility (>400 cm^2^ V^–1^ s^–1^ by terahertz spectroscopy). We use this PGNR to fabricate ambipolar field-effect transistors with appealing switching behaviour, and single-electron transistors displaying multiple Coulomb diamonds. These results open an avenue to *π*-extended nanostructures with engineerable electrical and magnetic properties by transposing the coordination chemistry of porphyrins into graphene nanoribbons.

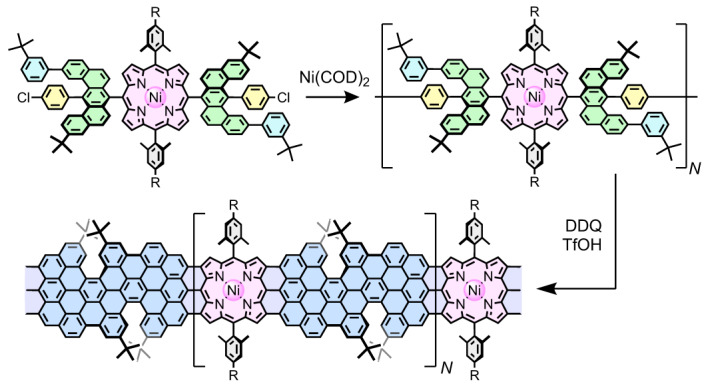

## Abstract (web summary)

The insertion of metal atoms and heteroaromatic units provides a way to tune the optical, electronic and magnetic properties of graphene nanoribbons. Now the synthesis of a porphyrin-fused graphene nanoribbon with a narrow bandgap and high charge mobility has been achieved, and this material used to fabricate field-effect and single-electron transistors.

## Main

Graphene nanoribbons (GNRs) have attracted attention because of their non-zero bandgaps, which arise from the quantum confinement of charge carriers^1–3^. They promise to function as key components in nanoscale electronic devices, as part of the drive towards smaller dimensions and lower energy consumption^4^. Graphene nanoribbons have been produced using so-called top-down strategies such as cutting graphene sheets, unzipping and squashing carbon nanotubes^5–7^, but these methods lack control over the precise edge structure and electronic properties. In recent years, bottom-up synthesis has enabled the preparation of atomically precise GNRs with variable edges and widths^3,8–10^, which have been integrated into single-molecule field-effect transistors^11^, illustrating their potential as materials for electronic devices. However, for practical applications, it would be desirable to incorporate diverse functional aromatic components such as quantum dots or heteroaromatic units into the conjugated backbones to tune their electronic properties. So far, this idea has been realized only by synthesizing GNR heterostructures on metal surfaces under ultra-high vacuum (UHV) conditions^12–16^. Solution-phase synthesis has inherent advantages of low cost and large-scale production, but GNR heterostructures, especially those containing heteroaromatic moieties, have rarely been prepared this way^17^.

**Fig. 1:**
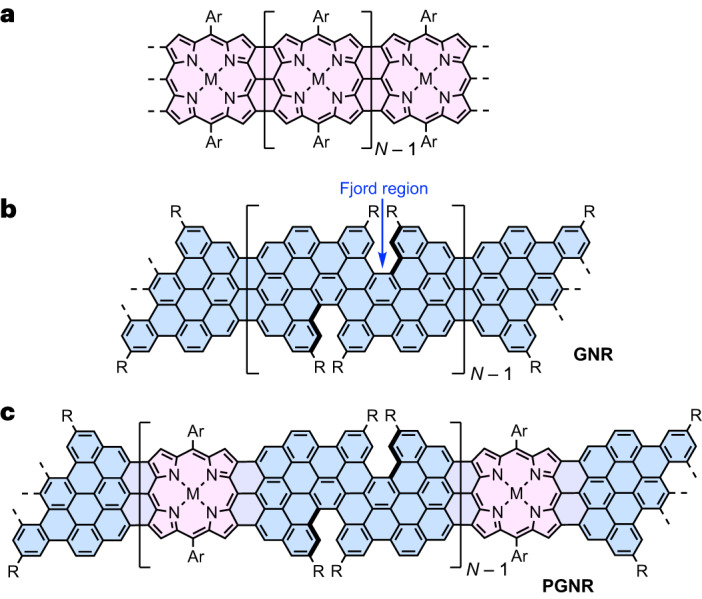
The design concept. **a**, Edge-fused porphyrin ribbon. **b**, Graphene nanoribbon with fjord-type edges. **c**, Porphyrin-fused fjord-edged graphene nanoribbon. The porphyrin and graphene nanoribbon moieties are highlighted in pink and blue, respectively. R = ^*t*^Bu- and Ar = bulky substituted phenyl.

Porphyrins constitute a remarkable class of functional molecules, not only because of their rigid geometry and robustness, but also for their photophysical properties, redox activity and coordination behaviour that make them suitable for organic electronics, including molecular switches^18^, molecular wires^19–21^ and spintronics^22–25^. Fully *π*-conjugated edge-fused porphyrin ribbons (Fig. 1a) are regarded as ideal molecular wires due to their extreme electronic delocalization, as indicated by electronic transitions at wavelengths >2,000 nm for neutral oligomers^26^ and a single-molecule conductance that is almost independent of length^27^. Porphyrins have been incorporated covalently into the edge of graphene^28^ and nanographenes under UHV conditions at submonolayer scales^14,15,24,25^. Some of the authors of this article recently reported the synthesis of porphyrin-fused nanographenes^29^, paving the way to *π*-extended porphyrin-containing graphene nanostructures.

**Fig. 2:**
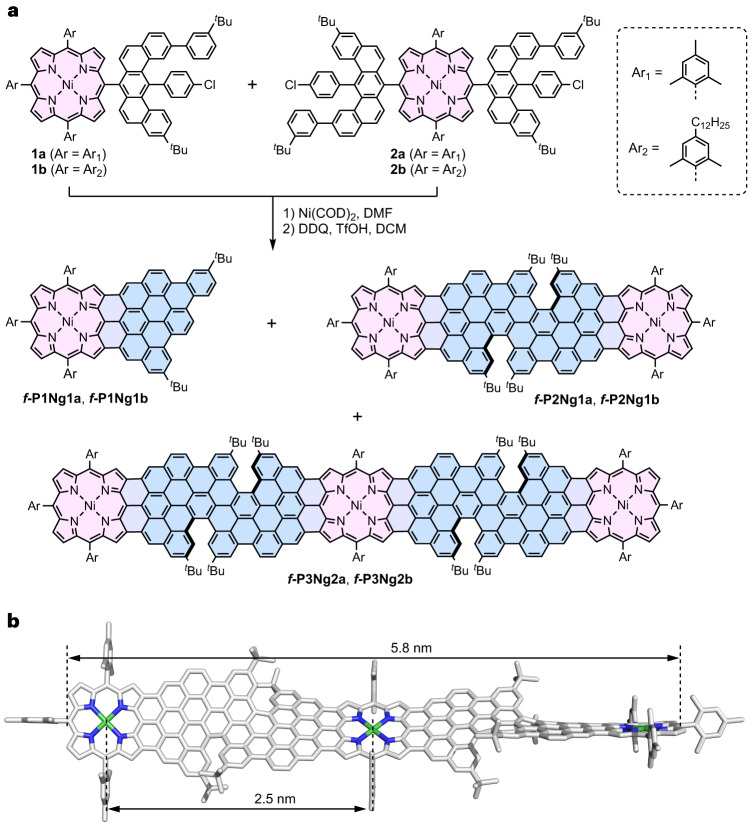
Synthesis of porphyrin-fused nanographene oligomers as model compounds. **a**, Synthetic route to ***f*-P1Ng1**, ***f*-P2Ng1** and ***f*-P3Ng2**. COD, 1,5-cyclooctadiene; DMF, *N*,*N*-dimethylformamide; DDQ, 2,3-dichloro-5,6-dicyano-1,4-benzoquinone; TfOH, trifluoromethanesulfonic acid; DCM, dichloromethane. **b**, DFT-optimized geometry of ***f*-P3Ng2a** (M,M,M,M configuration), showing the twisted edge structure, the molecular size and the distance between two porphyrin centres. Source data

Here we present the solution-phase synthesis of porphyrin-fused graphene nanoribbons (PGNRs) and investigation of their electrical properties. Our PGNR is a hybrid of an edge-fused porphyrin ribbon and a fjord-edged GNR (Fig. 1). The combination of a twisted fjord structure and flexible sidechains (Ar in Fig. 1) was chosen to provide high solubility and solution processability. Our synthesis is based on Yamamoto polymerization of a porphyrin monomer containing two chlorinated benzo[*m*]tetraphenes (Fig. 2a, **2a/b**), followed by cyclodehydrogenation^29^. The high efficiency of the cyclodehydrogenation has been verified by the synthesis and unambiguous characterization of model compounds with 1 to 3 porphyrin units and lengths of up to 6 nm (***f-*P1Ng1a/b**, ***f-*P2Ng1a/b** and ***f-*P3Ng2a/b**, Fig. 2). The structure of the PGNR has been characterized by solid-state NMR, ultraviolet–visible–near infrared (UV–vis–NIR) absorption, infrared, Raman, and X-ray photoelectron spectroscopy (XPS). An optical bandgap of 1.0 eV was inferred from the absorption spectrum, representing one of the narrowest bandgaps for solution-synthesized GNRs^17,30,31^. This PGNR provides an opportunity to investigate charge transport within the hybridized backbone and test the effect of incorporating porphyrin units. To this end, using contact-free ultrafast optical-pump terahertz-probe (OPTP) spectroscopy, a high local (short-range over tens of nanometres) charge mobility of 450 ± 60 cm^2^ V^–1^ s^–1^ was measured. In single-molecule field-effect transistors using graphene-based electrodes, the first prototypical devices already show mobilities of up to 40 cm^2^ V^–1^ s^–1^. These results highlight the potential application of PGNRs with ambipolar semiconductor character for single-molecule electronic devices.

## Results and discussion

### Design concept

Our synthetic route to PGNR involves two key steps: (1) synthesis of a high-molecular-weight, porphyrin-containing polyphenylene chain and (2) the subsequent planarization. After exploring several unsuccessful routes to porphyrin-GNR hybrids (Supplementary Figs. 1–3 and Supplementary Section 1.3), we chose to synthesize the hybrid of an edge-fused porphyrin ribbon (Fig. 1a) and a fjord-edged GNR (Fig. 1b), as displayed in Fig. 1c. Two *tert*-butyl groups were attached in close proximity at the edge of the GNR segments to form a fjord region and forcibly twist the molecular backbone, hindering *π*–*π* stacking and enhancing solubility^30,32–34^. Three considerations guided the design of dichloroporphyrin monomer **2a/2b** (Fig. 2a): (1) 3-*tert*-butylphenyl groups were attached to the benzo[*m*]tetraphene core instead of the 4-chlorophenyl to reduce the steric hindrance and facilitate polymerization; (2) 2,4,6-trimethylphenyl (mesityl) or 2,6-dimethyl-4-dodecylphenyl groups on the porphyrin hinder intermolecular aggregation (and thus enhance the solubility of PGNR) and the formation of pentagon rings between *meso*-phenyl and the *β*-positions of the porphyrin core in the fusion step^29,35^; (3) Ni(II) was inserted into the porphyrin to avoid protonation of the porphyrin core by the strong acid (TfOH) used during planarization^29^.

### Synthesis and characterization of model oligomers

The synthesis of model compounds ***f*-P1Ng1**, ***f*-P2Ng1** and ***f*-P3Ng2** starts from the porphyrin building blocks **1a**/**1b** and **2a**/**2b** (Fig. 2a; see Supplementary Section 1.4 for details on the synthesis). Yamamoto dimerization of **1a** in DMF/toluene gave the corresponding dimer (81% yield) along with the dechlorinated **1a** (16% yield). Yamamoto coupling of a 2:1 mixture of **1a** and **2a** afforded the porphyrin-terminated trimer (20% yield). Subsequent cyclodehydrogenation using DDQ/TfOH provided the fused oligomers ***f*-P1Ng1a**, ***f*-P2Ng1a** and ***f*-P3Ng2a** in 71%, 84% and 67% yield, respectively. In spite of their high molecular weights and rigid structures, the fused porphyrin oligomers ***f*-P2Ng1a** and ***f*-P3Ng2a** are soluble in organic solvents, benefiting from the *meso*-aryl substituents and twisted fjord edge, which hinder intermolecular *π*–*π* aggregation. The ^1^H-NMR spectrum of ***f*-P2Ng1a** (in CDCl_3_/CS_2_ = 1:1, v/v, 298 K) is well resolved (Supplementary Fig. 92), and the mass spectra of ***f*-P1Ng1a**, ***f*-P2Ng1a** and ***f*-P3Ng2a** exhibit single peaks at *m/z* = 1,250.61, 2,495.12 and 4,151.86 Da, respectively, with isotopic distributions that align with those calculated for C_89_H_68_N_4_Ni, C_178_H_130_N_8_Ni_2_, C_300_H_208_N_12_Ni_3_, reflecting the complete dehydrogenation of their precursors (Supplementary Figs. 110–115). The density functional theory (DFT)-optimized geometries of ***f*-P2Ng1a** and ***f*-P3Ng2a** are twisted with a dihedral angle of ~40° between the two planes defined by the 24 heavy atoms of the porphyrin cores, and a 2.5 nm separation between two neighbouring nickel atoms. The length of the backbone of ***f*-P3Ng2a** is 5.8 nm (Fig. 2b). The fused oligomers ***f*-P1Ng1b**, ***f*-P2Ng1b**, ***f*-P3Ng2b** with 2,6-dimethyl-4-dodecylphenyl groups on the porphyrins were synthesized using the same method, and the change of sidechain had a negligible effect on the cyclodehydrogenation reaction.

**Fig. 3:**
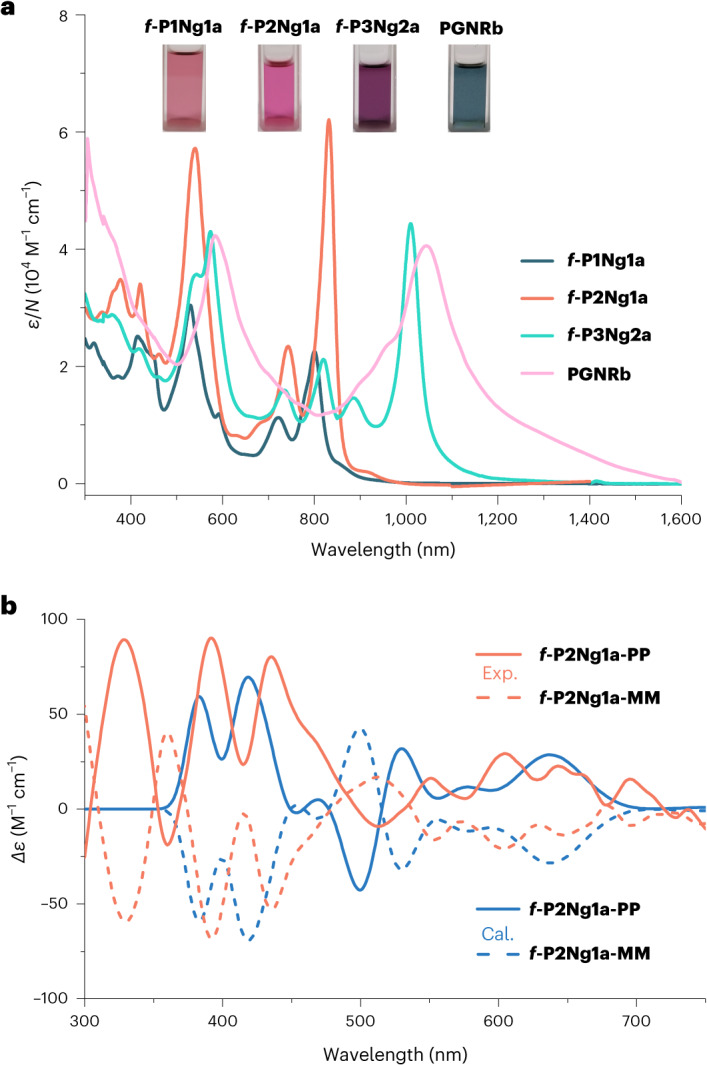
Room-temperature optical properties. **a**, Ultraviolet–visible–near infrared absorption spectra of ***f*-P1Ng1a**, ***f*-P2Ng1a** and ***f*-P3Ng2a** (in chloroform, *c* = 10^–5^ M) and **PGNRb** (in 1,2,4-trichlorobenzene, *c* = 0.025 mg ml^–1^). The molar absorption coefficient is normalized by the number of porphyrin units in each molecule, *N*. Photographs of the solutions are shown in the inset. **b**, Circular dichroism spectra (orange) of the two enantiomers of ***f*-P2Ng1a** measured in chloroform (*c* = 10^–5^ M), and the TD-DFT-calculated spectra using the LC-*ω*HPBE (*ω* = 0.1) functional (blue). Source data

Solutions of ***f*-P1Ng1a**, ***f*-P2Ng1a** and ***f*-P3Ng2a** in chloroform are pink to purple, and their UV–vis–NIR absorption bands shift to longer wavelengths with increasing molecular size, with absorption maxima at 800, 832 and 1,010 nm, respectively (Fig. 3a). The red-shift of ***f*-P2Ng1a**, relative to ***f*-P1Ng1a**, reflects the effective electronic conjugation between two porphyrins. The more significant red-shift of ***f*-P3Ng2a** compared with ***f*-P2Ng1a** mainly originates from the *π*-extension of the porphyrin in the middle by fusing one more benzo[*m*]tetraphene units on the other side of the porphyrin. Due to the asymmetric configuration of the fjord edges, ***f*-P2Ng1a** exists as two enantiomers that do not interconvert at 298 K. This was confirmed by chiral high-performance liquid chromatography (HPLC) separation, which afforded two fractions (***f*-P2Ng1a-MM** and ***f*-P2Ng1a-PP**), with circular dichroism spectra displaying mirror-symmetric patterns, in agreement with time-dependent DFT (TD-DFT) simulations (Fig. 3b). The experimental dissymmetry factor |*g*_abs_| (*g*_abs_ = Δ*ε*/*ε*) values of ***f*-P2Ng1a-PP** are 0.0013 (604 nm) and 0.00056 (695 nm). The fused trimer is probably a mixture of the enantiomers ***f-*P3Ng2a-MMMM** (calculated structure: Fig. 2b) and **f*-*P3Ng2a-PPPP**, and the *meso* diastereomer ***f-*P3Ng2a-MMPP**. We have not attempted to separate these stereoisomers, but we expect ***f-*P3Ng2a-MMMM** and ***f-*P3Ng2a-MMPP** to have very similar lengths and electronic absorption spectra.

### Synthesis and characterization of the PGNR

**Fig. 4:**
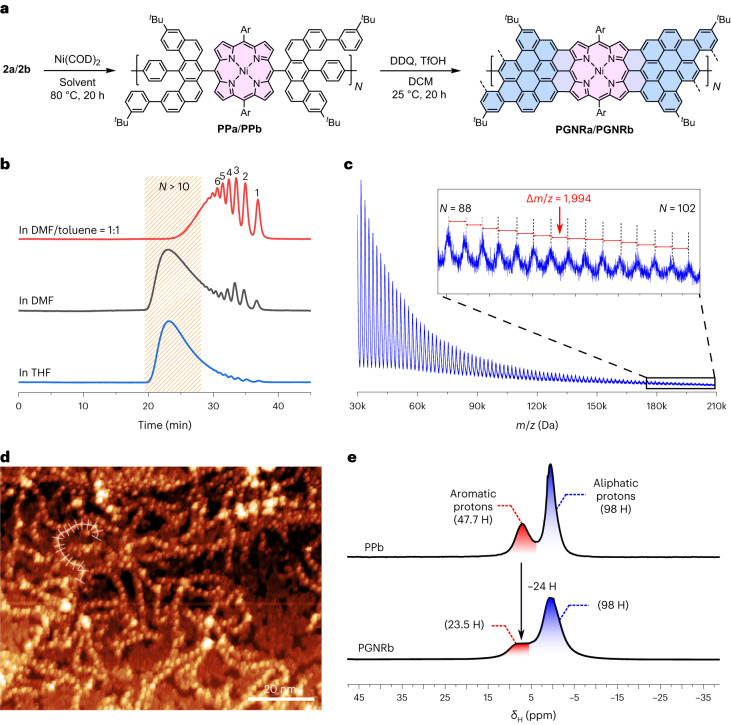
Synthesis and structural characterization of PGNRs. **a**, Synthesis of PGNRs, with the porphyrin and graphene nanoribbon moieties highlighted in pink and blue, respectively. **b**, Analytical GPC traces of Yamamoto polymerization products of dichloroporphyrin **2b** from reaction in DMF/toluene (1:1, v/v), DMF and THF (eluent: THF/1% pyridine, flow rate = 1 ml min^–1^, detection at 430 nm). Peaks are labelled with *N*, the number of repeat units. **c**, MALDI-TOF mass spectrum of **PPb** measured with *trans*-2-[3-(4-*tert*-butylphenyl)-2-methyl-2-propenylidene]malononitrile (DCTB) as the matrix, conducted in the linear mode. Peaks from the polymer extend up to a molecular weight of 210 kDa (102 repeat units) and the average *m/z* difference between neighbouring peaks corresponds to one repeat unit. **d**, STM topograph of **PPa** transferred from toluene solution to a Au(111) surface via electro-spray deposition, and subsequently annealed to 250 °C (*T*_sample_ = 4.7 K, *V*_sample-bias_ = 2 V, *I*_set-point_ = 50 pA). **e**, CP-MAS solid-state ^1^H-NMR spectra of **PPb** in comparison with **PGNRb**, and the integration of protons in the aromatic and aliphatic regions. Source data

Yamamoto polymerization of monomer **2a** using Ni(COD)_2_ (with DMF as the solvent) provided the polymer **PPa** in 94% yield (Fig. 4a); however, the cyclodehydrogenated product, **PGNRa**, was insoluble in common solvents such as dichloromethane, chloroform, toluene and tetrahydrofuran. To enhance the solubility, we changed to monomer **2b** with dodecyl chains on the *meso*-aryl groups. Although the polymerization of **2b** in DMF—or a mixture of DMF/toluene (1:1, v/v)—gave relatively short oligomers, using THF as the solvent afforded high-molecular-weight **PPb** (93% yield). Gel permeation chromatography (GPC) analysis of **PPb** gave one broad band (*t* = 23 min) along with tiny peaks from short oligomers (Fig. 4b). The number- and weight-average molar mass were 53.9 kDa and 132.7 kDa (polydispersity index = 2.46), respectively, using polystyrene as the standard and chloroform as the solvent (Supplementary Section 4). A weight-average molar mass of 67.9 kDa (*N̅* = 34) was deduced by using the porphyrin oligomers (*N* = 1–11) as standards to calibrate the GPC column, assuming the retention time is proportional to log*M* (where *M* is molecular weight; Supplementary Section 4). Matrix-assisted laser desorption/ionization-time of flight (MALDI-TOF) mass spectra of **PPb**—measured in the linear mode—detected polymer chains with *M* values of up to 210 kDa. All of the observed peaks from polymer chains are hydrogen terminated and the average separation between neighbouring peaks is 1.986 ± 0.048 kDa, which is close to the *M* (1.991 kDa) of one repeat unit (Fig. 4c). Mass spectra of the reisolated porphyrin monomer after the Yamamoto polymerization in THF show one single peak corresponding to protodechlorinated **2b**, which indicates that the Yamamoto coupling proceeds with a small amount of reduction without forming other by-products (Supplementary Fig. 116). The chain-like structure of **PPa** is confirmed via scanning tunnelling microscopy (STM) of **PPa** deposited on a gold surface under vacuum by electrospray deposition: it is possible to resolve the polymer chains with bright, repeating features arranged linearly with a period of 1.8–2.5 nm (Fig. 4d; refer to Supplementary Section 5 for STM characterization of **PPb**), in agreement with its non-planarity and Ni–Ni distance of 2.5 nm from DFT modelling (Fig. 2b). These images confirm the successful Yamamoto polymerization. Finally, **PPb** was planarized by applying the same condition used to synthesize the model compounds, to give **PGNRb** in 94% yield. Solid-state cross-polarization magic-angle spinning (CP-MAS) ^1^H NMR spectra of **PPb** exhibit relatively sharp peaks in the aromatic and aliphatic regions, and these peaks become broader after planarization to **PGNRb** (Fig. 4e). The integration ratio of protons in the aromatic versus aliphatic region decreased from 47.7/98 (expected 48/98) to 23.5/98 (expected 24/98), reflecting nearly complete dehydrogenation. Further evidence for the clean conversion of **PPb** to **PGNRb** was provided by solid-state ^1^H–^1^H double quantum–single quantum NMR, CP-MAS ^13^C NMR, Raman and Fourier-transform infrared spectroscopy (Supplementary Section 7).

**PGNRb** is soluble in organic solvents such as toluene, 1,2-dichlorobenzene and 1,2,4-trichlorobenzene, which facilitates device fabrication and investigation of its photophysical properties. **PGNRb** is probably a complex mixture of stereoisomers, because there is no control over the axial chirality of the helical nanographene segments between consecutive porphyrins during the cyclodehydrogenation step, which may contribute towards its high solubility. The UV–vis–NIR absorption spectra of the fused oligomers are similar to those of their mesityl analogues, with absorption maxima at 807 nm, 836 nm and 1,019 nm for ***f*-P1Ng1b**, ***f*-P2Ng1b** and ***f*-P3Ng2b**, respectively, recorded in 1,2,4-trichlorobenzene (Supplementary Fig. 20). The absorption spectrum of **PGNRb** has a peak at 1,045 nm (Fig. 3a), with a tail extending to 1,600 nm, and resembles that of ***f*-P3Ng2a**, confirming that they both contain the fused nanographene–porphyrin–nanographene moiety. The optical energy gap can be estimated from the onset of the absorption band at 1,200 nm, giving a value of 1.0 eV.

### Ultrafast OPTP spectroscopy

**Fig. 5:**
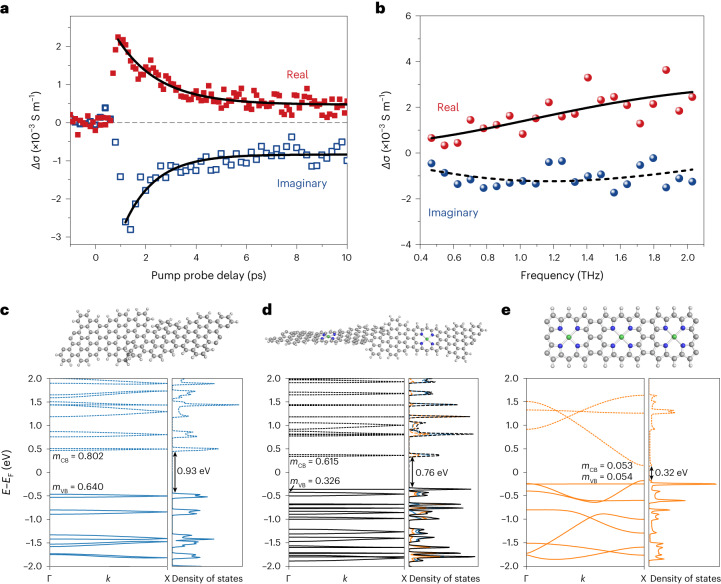
Ultrafast photoconductivity by terahertz spectroscopy and band structure. **a**, Time-resolved complex photoconductivity dynamics of **PGNRb** measured in 1,2,4-trichlorobenzene (0.33 mg ml^–1^) at room temperature. The solid lines represent fitted photoconductivity dynamics by a single-exponential decay (+long-lived offset) with the same decay time of 1.5 ± 0.2 ps for both the real and imaginary parts. **b**, Frequency-resolved terahertz photoconductivity of **PGNRb** measured at *t*_*p*_ = 2 ps. The data are fitted by the Drude–Smith model described in the main text. **c–e**, Energy bands as a function of the wavevector *k* and density of states of GNR (**c**), PGNR (**d**) and porphyrin ribbon (**e**). The energy band gap (*E*_g_), valence and conduction bands, and effective mass (*m*_CB_ and *m*_VB_) are shown; energies are reported relative to the intrinsic Fermi level (*E*_F_). The colour scale for PGNR represents the proportion of electron density on GNR (blue) or porphyrin (orange); occupied (unoccupied) levels are shown as solid (dotted) lines. Source data

To assess the potential of **PGNRb** for applications in electronic devices, we examined its charge-transport behaviour in solution by ultrafast, contact-free OPTP spectroscopy^36,37^. An optical pulse (400 nm, 50 fs duration, 280 µJ cm^–2^) was employed to photoinject charge carriers in **PGNRb** by promoting electrons from the valence band to the conduction band across the electronic bandgap. The terahertz probe is a freely propagating, single-cycle electromagnetic pulse. The transient nature of the terahertz probe field (~1 ps duration) ensures that photogenerated charges are driven and measured on a short length scale of tens of nanometres—comparable with the average ribbon length (~85 nm, with average degree of polymerisation *N̅* = 34)^31^. The interaction with charges leads to attenuation and phase-shift of the terahertz pulse. The relative attenuation of terahertz field −∆*E*/*E* is directly proportional to the real part of the transient complex photoconductivity, whereas the phase shift is related to the imaginary part of the photoconductivity. By varying the time delay *t*_P_ between the optical and terahertz pulses, the conductivity and recombination dynamics of photogenerated charge carriers are obtained (Fig. 5a). Following the free carrier generation after *t* = 0, photogenerated free electrons and holes rapidly (within ~1.5 ps) combine to form electrically insulating excitons, in line with previously reported sub-10 ps exciton formation time in GNRs^31,38^. Excitons feature a vanishing real part (owing to their net zero charge) yet a finite imaginary part (owing to their polarizability) of photoconductivity^31,39,40^. Accordingly, during the decay, the ratio of imaginary to real parts of the photoconductivity increases from ~1.3 to ~1.6.

By measuring the frequency-resolved photoconductivity spectra at *t*_p_ = 2 ps, when free-carrier response is dominant, we can characterize the free-carrier transport properties (Fig. 5b and Supplementary Section 11). **PGNRb** has negative imaginary conductivity and positive real conductivity, with an absolute amplitude increasing with frequency, in agreement with the Drude–Smith model^36,41^:

1 $${\sigma }_{\rm{DS}}=\frac{{\varepsilon }_{0}{\omega }_{\rm{p}}^{2}\tau }{1-i\omega \tau }\left(1+\frac{{c}_{\rm{p}}}{1-i\omega \tau }\right)$$

where, *ε*_0_ is the vacuum permittivity, *ω*_p_ is the plasma frequency and *τ* is the averaged carrier momentum scattering time. The parameter *c*_p_ (–1 ≤ *c*_p_ ≤ 0) describes the extent of the preferential backscattering effect that limits the long-range direct current transport in **PGNRb** (due to effects such as limited length or structural defects^42^), and is found to be *c*_p_ = –0.93 ± 0.04. The inferred scattering time for **PGNRb** (*τ* = 54 ± 7 fs) is much longer than for most pure carbon-based GNRs (~30 fs)^42–44^, suggesting superior charge transport in **PGNRb**.

The band structures of GNR, PGNR and the porphyrin ribbon were calculated using DFT (Fig. 5c–e; see also Methods and Supplementary Section 10) to provide insights into the charge mobility in these materials, and to estimate the effective masses of the charge carriers (*m*_CB_ and *m*_VB_). Porphyrin-fused graphene nanoribbon has a greater band dispersion than GNR, leading to a smaller effective mass and higher charge mobility (for a given *τ*). The local charge mobility (that is, without considering any backscattering events) was estimated with: *μ* = *eτ*/m*, which yields *µ* = 450 ± 60 cm^2^ V^–1^ s^–1^ by using the effective reduced mass m* = 0.213 *m*_0_. By considering the backscattering effect, we can further estimate the mobility in the direct current (dc) limit with *μ*_dc_ = *μ*(1 + *c*_p_) = 32 ± 4 cm^2^ V^–1^ s^–1^. These results imply that **PGNRb** possess one of the highest local (and direct current) mobilities among the known GNRs characterized by terahertz spectroscopy^33,45^. The band dispersion in the porphyrin ribbon (Fig. 5e) is greater than that in GNR or PGNR, resulting in a smaller effective reduced mass (m* = 0.027, Supplementary Table 2) and a higher charge carrier mobility, while also reflecting the greater planarity of the porphyrin ribbon.

### Single molecular electronics

**Fig. 6:**
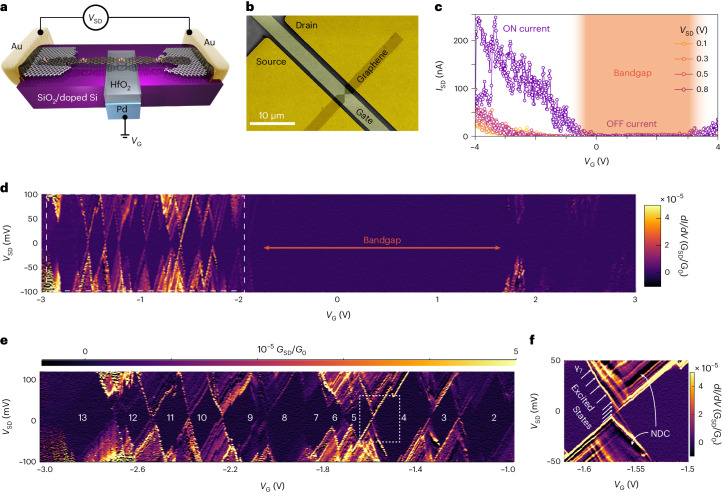
Single-molecule charge transport. **a**, Scheme of the electronic devices, where a single strand of **PGNRb** bridges two graphene electrodes (separated by a nanogap) connected to gold pads. A palladium gate is deposited on the SiO_2_/Si surface and covered with a 10-nm-thick HfO_2_ dielectric. **b**, False-colour scanning electron image of a typical device, showing the pads, graphene electrodes and gate. **c**, Characteristic curve for a field-effect transistor obtained with a single **PGNRb** at room temperature and a *V*_G_ sweep rate of 44 mV s^–1^ for various source-drain voltages. The electronic bandgap region and the ON and OFF states are highlighted. **d**, Single-electron transistor map of the differential conductance *G*_SD_ (colour scale bar, in units of the quantum of conductance *G*_0_) observed at 25 mK. **e**, Enlarged view of the Coulomb blockade regions, with the number of holes highlighted. **f**, Enlarged view of the region enclosed within the dashed white box in **e**, showing vibrational sublevels and negative differential conductance regions (labelled NDC). Source data

The charge-transport behaviour of **PGNRb** was investigated by fabricating single-nanoribbon devices, in which the **PGNRb** spans a 3–7 nm gap between two graphene electrodes, so that source-drain (*V*_SD_) and gate (*V*_G_) voltages can be applied while measuring the source-drain current *I*_SD_ (Fig. 6a,b). These devices were fabricated from electro-burnt graphene nanogaps, as described previously^45–47^. At room temperature (*T* = 300 K), the devices behave as field-effect transistors with a clearly observable bandgap and mild p-doping in most cases. Several devices exhibited ambipolar behaviour, with access to both n- and p- ON-states (Fig. 6c). The ON current is typically 10^3^-times greater than the OFF current (*I*_ON_/*I*_OFF_ ≈ 10^3^ at *V*_SD_ = 0.1 V), with the OFF-state conductance (*G*_OFF_ ≈ 7 pS) limited by both the bandgap energy (*E*_g_) and the presence of source-drain tunnelling currents resulting from the nanometre-scale width of the electro-burnt gaps. The highest ON-state conductance observed (*G*_ON_ ≈ 70 nS) corresponds to an ON-state conductivity in the region $\sigma =G \frac{L}{{w}_{{\rm{PGNR}}}} \approx 2.5\,{{\upmu}}{\rm{S}}$, for a **PGNRb** with width *w*_PGNR_ = 1.1 nm and an average length *L* ≈ 40 nm, estimated from the transport data of multiple devices at low temperature (as described below). Many devices show a subthreshold swing (SS) of approximately 800 mV dec^–1^, but some devices reach as low as SS ≈ 400 mV dec^–1^ at *V*_SD_ = 0.1 V, indicating that excellent gate control of the transistor switching is achievable (Supplementary Fig. 33). Field-effect-transistors are frequently compared using linear and saturation field-effect mobilities, which, in our devices, reach 40 ± 5 and 4 ± 1 cm^2^ V^–1^ s^–1^, respectively, in overall agreement with the direct current limit of the terahertz mobility. The PGNR transistors in air exhibit no or very little hysteresis (~1 V; see Supplementary Fig. 33), whereas hysteresis generally plagues the behaviour of carbon nanotube devices when sweeping the transistor^48^.

At low temperatures, when electrons do not have enough thermal energy to overcome the electrode–PGNR barrier, conduction can only happen via single-electron processes when one of the PGNR levels is in resonance with the Fermi level of one of the leads. Mapping *G*_SD_ versus *V*_G_ and *V*_SD_ at millikelvin temperatures (Fig. 6d,e) reveals a *V*_G_ region of suppressed conductance that corresponds with the bandgap. Periodic Coulomb diamonds are observed outside of this region; they have similar sizes and are separated by ridges of high *G*_SD_ that always display the same slope, as is typical of a single conduction channel. We estimate the lengths *L* of the PGNR from the Coulomb diamonds addition energies (*E*_add_) by considering the PGNRs as rectangular-shaped electron boxes^45,49^:

2 $$L=\frac{{\rm{e}}^{2}\left[8d\arctan \left(\frac{w}{4d}\right)+w\log \left(1+\frac{16{d}^{2}}{{w}^{2}}\right)\right]}{4\uppi {\varepsilon }_{0}{\varepsilon }_{\rm{r}}{E}_{\rm{add}}},$$

where *e* is the electron charge, *ε*_0_ is the vacuum permittivity, and *ε*_r_ = 15.4 is the relative permittivity of HfO_2_ (Supplementary Section 12); *w* is the PGNR width and *d* is the thickness of HfO_2_. In our devices, we detected PGNRs of lengths of *L* = 40 ± 3, 35 ± 3 and 10 ± 3 nm, respectively.

The vibrational levels—visible inside the Coulomb diamonds (Fig. 6f)—provide a fingerprint of the PGNR and can be related to the Raman and infrared modes reported for nickel-porphyrins (Supplementary Table 4). Furthermore, we observe substantial regions of negative differential conductance (NDC), which account for up to 70% of the conductance peak (Supplementary Section 12). This verifies predictions made for doped GNRs, which have a characteristic evolution of the local density of states^50^: in PGNRs the nickel *d*-electrons form isolated states that become mismatched when the *V*_SD_ window is increased, so that the current will suddenly decrease on increasing the bias, producing the NDC peaks.

## Conclusion

Our solution-phase synthesis of PGNR takes advantage of Yamamoto polymerization of a dichloroporphyrin monomer, followed by cyclodehydrogenation using DDQ/TfOH. A high average degree of polymerization (*N̅* ≈ 34) was achieved, giving polymer chains with a weight-average length of 85 nm. This synthesis opens the possibility of creating numerous new PGNR hybrids. Introducing the coordinating porphyrin ligands into the nanoribbon will allow incorporation of diverse transition and rare-earth metals, including magnetic ones. The optical bandgap of PGNR estimated from absorption spectra (1.0 eV) is among the lowest reported for solution-synthesized GNRs. The terahertz measurements and single-molecule charge-transport experiments reveal high charge carrier mobilities (*μ*_dc_ ≈ 32 ± 4 cm^2^ V^–1^ s^–1^), appealing field-effect-transistor characteristics at room temperatures, and clean single-electron-transistor behaviour at millikelvin temperatures. The minimum voltage of the NDC regions is below 10 mV, indicating the possibility of low-power operation. Porphyrin-fused graphene nanoribbons with these characteristics, and the potential to bind a multitude of metal ions, open up new pathways for a variety of areas that range from magnetism, molecular electronics, spintronics and memory applications.

## Methods

### General methods for synthesis

All reactions with air- or moisture-sensitive compounds were performed under argon atmosphere using standard Schlenk techniques. Unless otherwise noted, all starting materials were purchased from commercial sources and used without further purification. All other reagents were used as received. Thin layer chromatography was performed on silica gel-coated aluminium sheets with F254 indicator; column chromatography separation was performed with silica gel (particle size = 0.063−0.200 mm). Solution NMR spectra were recorded using Bruker 300, 400 and 600 MHz NMR spectrometers. Chemical shifts (*δ*) were expressed in parts per million relative to the residual of solvents (dichloromethane-*d*_2_, ^1^H: 5.32 ppm, ^13^C: 54 ppm; chloroform-*d*, ^1^H: 7.26 ppm, ^13^C: 77.16 ppm; tetrahydrofuran-*d*_8_, ^1^H: 3.58 ppm, ^13^C: 67.57 ppm). Abbreviations: s = singlet, d = doublet, t = triplet, q = quartet, m = multiplet. Coupling constants (*J*) were recorded in hertz. Solid-state NMR experiments were recorded on a Bruker Avance IIIHD WB400 operating at 399.89 MHz for ^1^H and 100.57 MHz for ^13^C (14 T). Samples were packed in 3.2 mm O.D. rotors and experiments were recorded using a X/Y/F/H quad MAS probe. A MAS rate of 12 kHz was used for the ^13^C CP-MAS NMR spectra; the set-up involved applying a sequence of variable X-amplitude spin-lock pulses^51^, and SPINAL-64 proton decoupling. Typically, 24,000 transients were acquired using a contact time of 2.5–5 ms, an acquisition time of 25 ms (2,048 data points zero filled to 24 K) and a recycle delay of 0.5–2 s. All of the ^1^H detected experiments were acquired with a MAS rate of 20 kHz. The direct polarization magic-angle spinning (DPMAS) method used a background suppression sequence^52^. The 2D ^1^H–^1^H single quantum–double quantum correlation experiments were recorded using the compensated back-to-back sequence^53^ with 1 rotor period dipolar recoupling; 128 scans, 2,048 points and 64 increments were acquired using a 3.5 µs π/2 pulse and a 2.5 s recycle delay. ^13^C NMR spectra were referenced to glycine (the carbonyl resonance was taken to be at *δ* = 176.5 ppm on a scale where *δ*_TMS_ = 0 ppm) as a secondary reference. ^1^H spectra were referenced to adamantane (*δ* = 1.82 ppm on a scale where *δ*_TMS_ = 0 ppm) as a secondary reference. High-resolution mass determinations were performed with electrospray ionization or atmospheric pressure chemical ionization on a Thermo Exactive High-Resolution Orbitrap FTMS. MALDI-TOF measurements were performed on a Bruker Autoflex Speed MALDI TOF/TOF mass spectrometer using DCTB in THF as the supporting matrix. Analytical GPC was performed on a VWR-Hitachi HPLC-unit LaChrom Elite equipped with a L-2130 quaternary pump, L-2455 diode array detector, L-2200 autosampler, and a set of JAIGEL-3H-A (8 × 500 mm) and JAIGEL-4H-A (8 × 500 mm) columns using THF/1% pyridine as the eluent at a flow rate of 1.0 ml min^–1^. Preparative GPC was performed on a Shimadzu UFLC HPLC (recycling) system equipped with a LC-20 AD pump, SPD-20A UV detector, and a set of JAIGEL 3H (20 × 600 mm) and JAIGEL 4H (20 × 600 mm) columns, using toluene/1% pyridine as the eluent at a flow rate of 3.5 ml min^–1^. Preparative size exclusion chromatography was performed using Bio-Beads S-X1, 40–80 µm bead size (Bio Rad) with toluene as the eluent. Number- (*M*_n_) and weight-average (*M*_w_) molecular weights were determined using an Agilent Technologies 1260 infinity GPC at 40 °C in chloroform, using two PLgel 10 μm Mixed-B columns in series (300 × 7.5 mm), and calibrated against narrow dispersity (PDI < 1.10) polystyrene standards.

### Chiral resolution

Chiral resolution of ***f*-P2Ng1a** was performed on an Agilent 1260 infinity LC system equipped with a Chiralpak ID column (5 µm particle sizes, 250 × 4.6 mm) at 298 K. Eluent: *n*-hexane/isopropanol/dichloromethane at 96/2/2, v/v; flow rate = 0.6 ml min^–1^. ***f*-P2Ng1a** was detected by the absorption at 378 nm.

### STM measurement

Images were acquired using a Scienta Omicron POLAR SPM operating under UHV, with a base pressure of 3 × 10^–10^ mbar; samples were imaged at a temperature of 4.7 K. All STM measurements were performed in constant current mode, using electrochemically etched tungsten tips coated in gold during tip optimization, by controlled indentation into the Au(111) single-crystal substrate. The Au(111) single crystal (Surface Preparation Laboratory) was prepared by cycles of argon ion sputtering for 30 min at 1.0 keV, followed by annealing at 770 K for 30 min. The polymers **PPa** and **PPb** were transferred from a toluene/methanol solution (3:1 ratio, 100 µg ml^–1^) onto a Au(111) surface held under vacuum using electrospray deposition (using a potential of 1.2–2.0 kV to initiate the electrospray event, base pressure during deposition was 1–9 × 10^–7^ mbar).

### Spectroscopy analysis

Ultraviolet–visible–NIR absorption spectra were recorded on a Perkin-Elmer Lambda 20 spectrometer or a Jasco V-770 UV–vis–NIR spectrophotometer in chloroform or 1,2,4-trichlorobenzene using a 10 mm quartz cuvette 3.5 mm × 10.0 mm at 298 K and a concentration of 10^–5^ M. Circular dichroism spectra were recorded in a ultraviolet-grade quartz cuvette with a 10 mm path-length on a Chirascan circular dichroism spectrometer (Applied Photophysics) at 298 K with a concentration of 10^–5^ M. Infrared spectra were obtained on a Bruker Tensor 27 Fourier-transform infrared spectrometer equipped with an attenuated total reflection set-up. Raman spectra were recorded on a DXR3 Raman spectrometer (Thermo Fisher Scientific) using 532 nm excitation. X-ray photoelectron spectroscopy samples were analysed using a Kα XPS instrument (Thermo Fisher Scientific) equipped with a microfocused monochromated aluminium X-ray source. The source was operated at 12 keV and a 300 μm spot size was used. The analyser operates at a constant analyser energy 200 eV for survey scans and 50 eV for detailed scans. Charge neutralization was applied using a combined low energy/ion flood source. Data acquisition and analysis were performed using the Avantage software by Thermo Fisher Scientific. Normalized atomic percentages were determined from peak areas of the elemental main peaks detected on the survey scan following background subtraction and application sensitivity factors provided by Thermo Fisher Scientific. The OPTP set-up was driven by a commercial mode-locked titanium sapphire femtosecond laser with central wavelength of ~800 nm, pulse duration of 50 fs and repetition rate of 1 kHz. The output laser was separated into three beamlines for terahertz generation, sampling and pump. The terahertz pulse was generated by optical rectification in a 1 mm <110>-oriented ZnTe crystal upon 800 nm laser impingement. The terahertz pulse was focused and transmitted through the sample (**PGNRb** solution in a 2-mm-thick cuvette or **PGNRb** film on a fused silica substrate), and collected by a 90° off-axis parabolic mirror before being refocused onto another ZnTe crystal. The transmitted single cycle terahertz waveform was then detected on the second ZnTe crystal by a time-delayed weak 800 nm pulse through electrooptic sampling. To optically pump the sample, a frequency-doubled 400 nm pulse—generated via a barium borate crystal—was employed to propagate collinearly through the sample with a terahertz pulse. The relative time delay between the terahertz and pump pulses was controlled by a mechanically adjustable delay stage.

### Device fabrication

Photolithography was used to pattern arrays of gate electrodes (Ti/Pd 5/25 nm) on a silicon wafer with 300 nm of SiO_2_ on top. A 10-nm-thick dielectric layer of HfO_2_ was deposited using atomic layer deposition, and a second photolithography step was used to pattern source and drain electrodes (Ti/Au 5/65 nm), aligned so that the gate electrode sits between source and drain. Chemical vapour deposited (Graphenea) graphene was transferred and shaped via electron beam lithography, followed by O_2_ plasma etching. Feedback-controlled electroburning was used to produce nanogaps with widths of 1–10 nm (refs. ^46,54^). The **PGNRs** were added stochastically into the junctions from a ~8 μl suspension prepared by sonicating powders in toluene (0.02 mg ml^–1^) for 60 min. *I*_SD_–*V*_SD_ characteristics were recorded for all devices immediately after nanogap formation and after drop-casting of **PGNRs**; the presence of **PGNR** was detected by an increase of the conductance *G* = ∂*I*_SD_/∂*V*_SD_) from <1 nS to hundreds of nanosiemens. Gating was obtained by a palladium electrode etched into undoped silicon and covered by 10 nm of HfO_2_

### Electronic transport

Cryogenic measurements were performed in an OI-Triton 200 dilution refrigerator using low-noise direct current electronics and a room-temperature probe station (Keithley). Room-temperature measurements were taken at ambient conditions with a Cascade Microtech Summit 12000 semi-automated, three-probe system. CuBe probes with tip diameters of 30 μm were used to contact the source and drain the gold pads, while tungsten probes with tip diameters of 10 μm were used for the gate probe.

### DFT calculations

Density functional theory calculations on porphyrin-fused oligomers were performed using the Gaussian 16/A.03 software package^55^ at the B3LYP level of theory with the 6-31G(d,p) basis set for carbon, nitrogen and hydrogen atoms, and the LanL2DZ basis set for nickel atoms. TD-DFT calculations were performed using the B3LYP, BLYP35 and LC-*ω*HPBE (*ω* = 0.1) functionals, and used a polarizable continuum model with chloroform as the solvent. The band structures were calculated using DFT implemented in SIESTA. We employed a Perdew–Burke–Ernzerhof generalized gradient approximation functional with the DZP basis set. The energy shift of the localized basis was set to 100 meV. An energy cut-off of 1,000 Ry and a Monkhorst-Pack grid of (50,1,1) were used to ensure the convergence of the results. A vacuum region of at least 30 Å was used in non-periodic directions to prevent unwanted interactions. The structure was optimized until the maximum force on the atoms was less than 0.01 eV Å^–1^. In the density of states calculation, Gaussian broadening of 0.01 eV was used for all bands.

## Online content

Any methods, additional references, Nature Portfolio reporting summaries, source data, extended data, supplementary information, acknowledgements, peer review information; details of author contributions and competing interests; and statements of data and code availability are available at 10.1038/s41557-024-01477-1.

### Supplementary information

Supplementary Information Supplementary Figs. 1–116, Tables 1–6, Experimental Procedures and Discussion.

Supplementary Data 1 Crystallographic data file for **12** (CCDC 2225521).

### Source data

Source Data Fig. 2 Coordinates of ***f*-P3Ng2a** shown in Fig. 2b.

Source Data Fig. 3 Data for UV-vis absorption spectra of ***f*-P1Ng1a**, ***f*-P2Ng1a**, ***f*-P3Ng2a** and **PGNRb** (shown in Fig. 3a), and data for experimental and simulated circular dichroism spectra of ***f*-P2Ng1a-PP** and ***f*-P2Ng1a-MM** (shown in Fig. 3b).

Source Data Fig. 4 Data for analytical GPC traces of polymerization products of **2b** in DMF+toluene, DMF and THF (shown in Fig. 4b), data for MALDI-TOF mass spectrum of **PPb** in linear mode (shown in Fig. 4c), and data for CP-MAS solid-state ^1^H NMR spectra of **PPb** and **PGNRb** (shown in Fig. 4e).

Source Data Fig. 5 Data for time-resolved complex photoconductivity dynamics of **PGNRb** (shown in Fig. 5a), data for frequency-resolved THz photoconductivity of **PGNRb** measured at *t*_*p*_ of 2 ps (shown in Fig. 5b), and data for coordinates, band structure and density of states of GNR, PGNR and porphyrin nanoribbon (shown in Fig, 5c, 5d and 5e, respectively).

Source Data Fig. 6 Data for characteristic curve for a field-effect transistor for various source-drain voltages (shown in Fig. 6c), data for single-electron transistor map observed at 25 mK (shown in Fig. 6d), and data for enlarged views of Coulomb blockade regions (Fig. 6e) and diamonds (Fig. 6f).

## Data Availability

All data supporting the findings of this study are available within the paper and its Supplementary Information. Crystallographic data for the structure reported in this manuscript have been deposited at the Cambridge Crystallographic Data Centre under deposition no. CCDC 2225521 (**12**). These data can be obtained free of charge via https://www.ccdc.cam.ac.uk/structures. Source Data are provided with this paper.
