# Supplementary material for: Porphyrin-fused graphene nanoribbons
Source: Nat Chem. 2024 Mar 8;16(7):1133–40. doi: 10.1038/s41557-024-01477-1 (PMC11230900; doi:10.1038/s41557-024-01477-1)
Supplement: Supplementary file 1 — Supplementary Figs. 1–116, Tables 1–6, Experimental Procedures and Discussion. [file 41557_2024_1477_MOESM1_ESM.pdf]

nature chemistry

Article

https://doi.org/10.1038/s41557-024-01477-1

Porphyrin-fused graphene nanoribbons

In the format provided by the authors and unedited

Table of Contents

1. Supplementary Methods 2

1.1 General Methods 2
1.2 Materials 3
1.3 Initial Abortive Synthetic Routes 3
1.4 Synthetic Details 5

2. X-Ray Structure 29

3. Chiral Resolution 31

4. GPC Measurements 32

5. STM Characterization 33

6. Solid-State NMR Measurements 35

7. FT-IR and Raman Spectroscopy 36

8. XPS Analysis 37

9. UV-vis-NIR Absorption Spectra 38

10. DFT Calculations 38

11. THz Spectroscopy 40

12. Single-Molecule Devices 43

12.1 Experimental set-up and device fabrication 43
12.2 Results from low temperature measurements 44
12.3 Results from room temperature measurements 50

13. NMR and MALDI-TOF Mass Spectra 53

13.1 NMR Spectra 53
13.2 MALDI-TOF Mass Spectra 91

14. Supplementary References 94

1. Supplementary Methods

1.1 General Methods

All reactions with air- or moisture-sensitive compounds were carried out in oven-dried glassware under argon atmosphere using standard Schlenk techniques. Thin layer chromatography (TLC) was done on silica gel coated aluminum sheets with F254 indicator and visualized using UV irradiation (λ = 254 nm). Flash column chromatography separation was performed with silica gel (particle size 0.063–0.200 mm) as the stationary phase. Solution nuclear magnetic resonance (NMR) spectra were recorded using Bruker 300, Bruker 400 and Bruker 600 MHz NMR spectrometers. NMR spectra were processed using MestReNova v14.3.0. Chemical shifts (δ) were expressed in ppm relative to the residual of solvents (dichloromethane-d_2, ^{1}H: 5.32 ppm, ^{13}C: 54.00 ppm; chloroform-d, ^{1}H: 7.26 ppm, ^{13}C: 77.16 ppm; tetrahydrofuran-d_8, ^{1}H: 3.58 ppm, ^{13}C: 67.57 ppm). Abbreviations: s = singlet, d = doublet, t = triplet, q = quartet, m = multiplet. Coupling constants (J) were recorded in Hertz. Solid-state NMR experiments were recorded on a Bruker Avance IIIHD WB400 operating at 399.89 MHz for ^{1}H and 100.57 MHz for ^{13}C (14T). Samples were packed in 3.2 mm O.D. rotors and experiments were recorded using a X/Y/F/H quad magic-angle spinning (MAS) probe. For the ^{13}C CP-MAS NMR spectra, a MAS rate of 12 kHz was used and a sequence with a variable X-amplitude spin-lock pulse[1] and spinal64 proton decoupling. Typically, 24000 transients were acquired using a contact time of 2.5–5 ms, an acquisition time of 25 ms (2048 data points zero filled to 24 K) and a recycle delay of 0.5–2 s. All the ^{1}H detected experiments were acquired with a MAS rate of 20 kHz. The DPMAS used a background suppression sequence[2]. The 2D ^{1}H-^{1}H single quantum-double quantum (SQ-DQ) correlation experiments were recorded using the compensated Back-to-Back (BaBa) sequence[3] with 1 rotor period dipolar recoupling, 128 scans, 2048 points and 64 increments were acquired using a 3.5 μs $\pi/2$ pulse and 2.5 s recycle delay. ^{13}C NMR spectra were referenced to Glycine (the carbonyl resonance was taken to be at δ = 176.5 ppm on a scale where δ(TMS) = 0 ppm) as a secondary reference. ^{1}H spectra were referenced to Adamantane (δ = 1.82 ppm on a scale where δ(TMS) = 0 ppm) as a secondary reference. High-resolution mass (HR MS) determinations were carried out on a Thermo Exactive Orbitrap mass spectrometer equipped with a Waters Acquity liquid chromatography system or a G6545A Q-ToF (Agilent GmbH, Waldbronn, Germany) using either the heated electrospray (HESI-II) probe for positive electrospray ionization (ESI$^+$) or the atmospheric pressure chemical ionization (APCI). Matrix-assisted laser desorption/ionization-time of flight (MALDI-TOF) mass spectra (MS) were measured using a Bruker Autoflex MALDI-TOF/TOF instrument using *trans*-2-(3-(4-(*t*-butyl)phenyl)-2-methylallylidene)malononitrile (DCTB) in THF as supporting matrix. Analytical gel permeation chromatography (GPC) was carried out on VWR-Hitachi HPLC-unit LaChrom Elite equipped with L-2130 quaternary pump, L-2455 diode array detector, L-2200 autosampler and a set of JAIGEL-3H-A (8 × 500 mm) and JAIGEL-4H-A (8 × 500 mm) columns using THF/1% pyridine as eluent with a flow rate of 1.0 mL/min. Preparative GPC were carried out on a Shimadzu UFLC HPLC (recycling) system equipped with a LC-20 AD pump, SPD-20A UV detector and a set of JAIGEL 3H (20 × 600 mm) and JAIGEL 4H (20 × 600 mm) columns in toluene/1% pyridine as the eluent with a flow rate of 3.5 mL/min. Preparative size exclusion chromatography (SEC) was carried out using Bio-Beads S-X1, 40–80 μm bead size (Bio Rad) with toluene as eluent. Number-average (M_n) and weight-average (M_w) molecular weights were determined using an Agilent Technologies

1260 infinity GPC at 40 °C in chloroform with a flow rate of 1.0 mL/min, using two PLgel 10 micrometer Mixed-B columns in series (300 × 7.5 mm), and calibrated against narrow dispersity (PDI < 1.10) polystyrene standards. UV-vis absorption spectra were recorded on a Perkin-Elmer Lambda 20 spectrometer or a Jasco V-770 UV-vis/NIR spectrophotometer using a 10 mm quartz cuvette at 298 K. Chiral resolution was performed on an Agilent 1260 infinity LC system equipped with a Chiralpak® ID column (5 µm particle sizes, 250 × 4.6 mm). Circular dichroism spectra were recorded in a UV-grade quartz cuvette with a 10 mm path-length on a Chirascan circular dichroism spectrometer (Applied Photophysics) at 298 K. IR spectra were recorded as the neat compound on a Bruker Tensor 27 FT-IR spectrometer equipped with an attenuated total reflection (ATR) setup. The samples were deposited on the diamond crystal and pressed on it with a stamp. Each sample was measured with a scan number of 256 and the background was subtracted. Raman spectra were recorded on a DXR3 Raman spectrometer from Thermo Fisher Scientific using 532 nm excitation. X-ray photoelectron spectroscopy (XPS) were measured using a Thermo Scientific K-Alpha XPS instrument equipped with a microfocus monochromated Al X-ray source. The source was operated at 12 keV and a 300 µm spot size was used. The analyzer operates at a constant analyzer energy (CAE) 200 eV for survey scans and 50 eV for detailed scans. Charge neutralization was applied using a combined low energy / ion flood source. The data acquisition and analysis were performed with Thermo Scientific Avantage software. Normalized atomic percentages were determined from peak areas of the elemental main peaks detected on the survey scan following background subtraction and application of Thermo sensitivity factors. X-ray single-crystal data was collected at 150 K on Rigaku Supernova A diffractometer using a copper radiation source.

1.2 Materials

All starting materials were purchased from major commercial suppliers (Sigma-Aldrich, Merck, Fluorochem, Fischer Scientific, Tokyo Chemical Industry, and Acros Organics) and used without further purification, unless otherwise noted. Dry solvents (dichloromethane, chloroform, *N*,*N*-dimethylformamide, tetrahydrofuran, 1,4-dioxane, and toluene) used for reactions were purified by a MBraun MB-SPS-5 bench-top solvent purification system having been passed through anhydrous alumina column under nitrogen atmosphere (H_2O content < 20 ppm).

1.3 Initial Abortive Synthetic Routes

We initially attempted to fuse porphyrin motifs to the backbone of 9-atoms wide armchair-edged graphene nanoribbon (9-AGNR) to synthesize porphyrin-fused 9-AGNRs as shown in Supplementary Figures 1–3[4, 5]. Although we could obtain the monomeric building blocks **S8** and **S24**, the synthesis of the target porphyrin-GNR hybrids was not successful: we could not convert the methoxy groups on **S8** to pinacol boronic ester groups (BPin) or the polymerization of **S24** did not work because of steric hindrance. It is also important to mention that, the solution synthesis of GNRs always suffers from low solubility, due to strong π-π aggregation induced by their planar π-conjugation backbone. This problem could not be avoided with this design strategy.

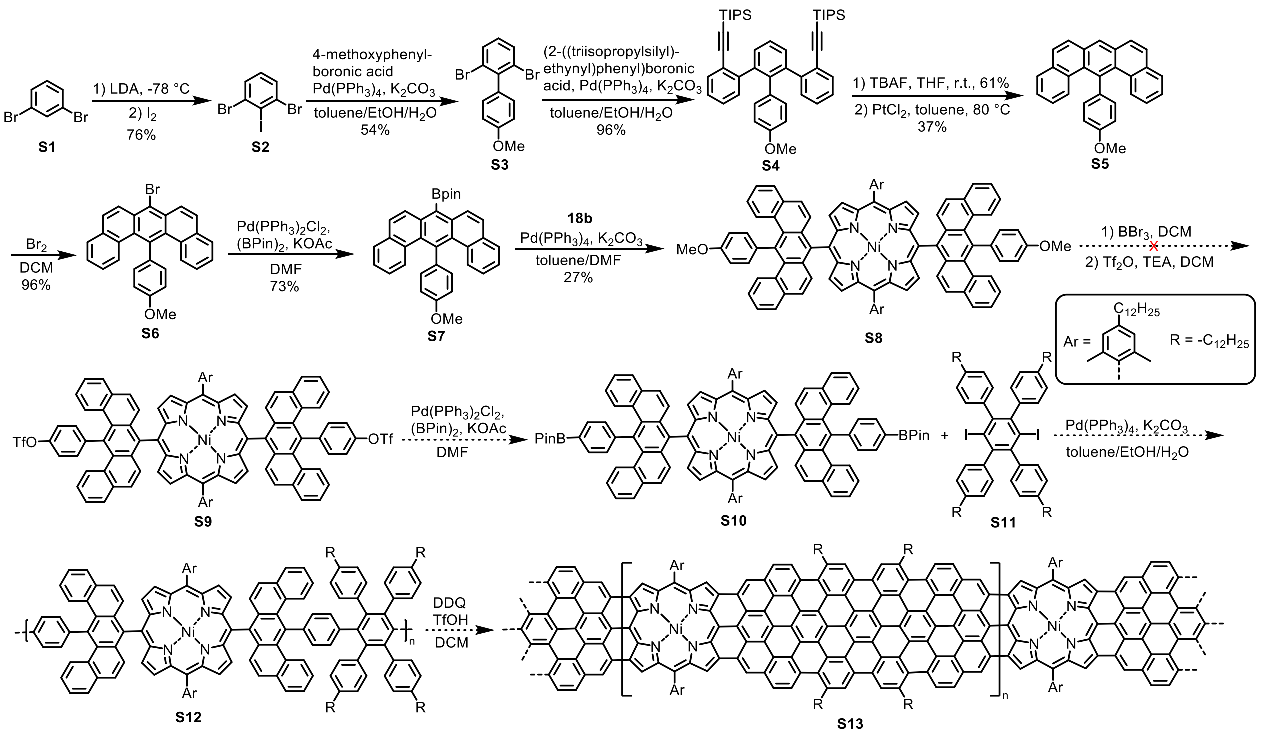

Supplementary Figure 1. Synthetic route 1 towards a porphyrin-fused 9-AGNR using Suzuki coupling polymerization as key step, which failed due to the difficulty of converting methoxy groups on intermediate **S8** to pinacol boronic ester (-BPin) to synthesize intermediate **S10**. The synthesis of porphyrin **18b** is shown in Supplementary Figure 5 and **S11** was synthesized using the literature reported method[6].

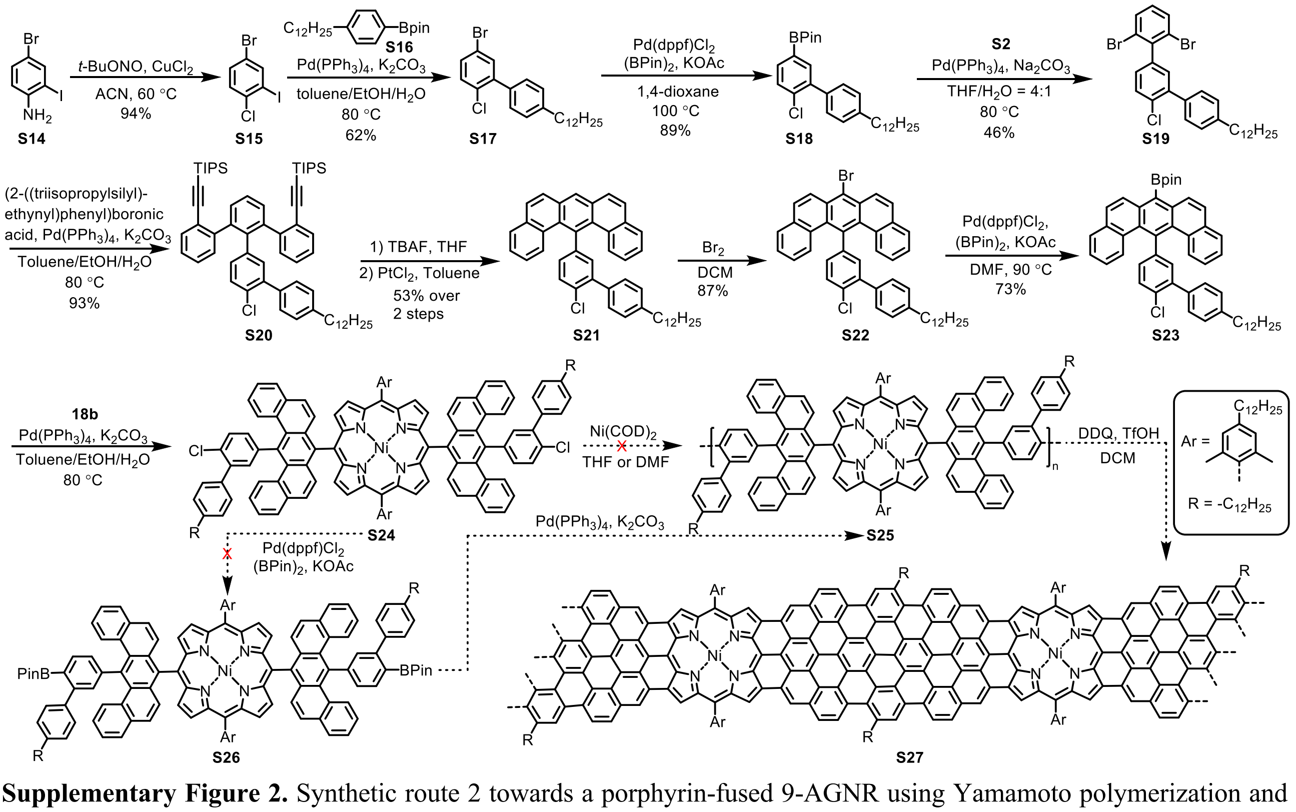

Supplementary Figure 2. Synthetic route 2 towards a porphyrin-fused 9-AGNR using Yamamoto polymerization and Suzuki coupling polymerization of a dichloroporphyrin monomer **S24**. The dichloroporphyrin **S24** could be successfully synthesized, however, the Yamamoto coupling using $Ni(COD)_2$ as catalyst or in-situ generated Ni(0) catalyst under the condition of $NiBr_2/Zn/Et_4NI/PPh_3/AcNMe_2$ only gave dechlorination by-product or no reaction. Borylation of **S24** was also not successful because of the low reactivity of arylchloride and the steric hindrance from the *ortho*-phenyl group. The synthesis of porphyrin **18b** is shown in Supplementary Figure 5.

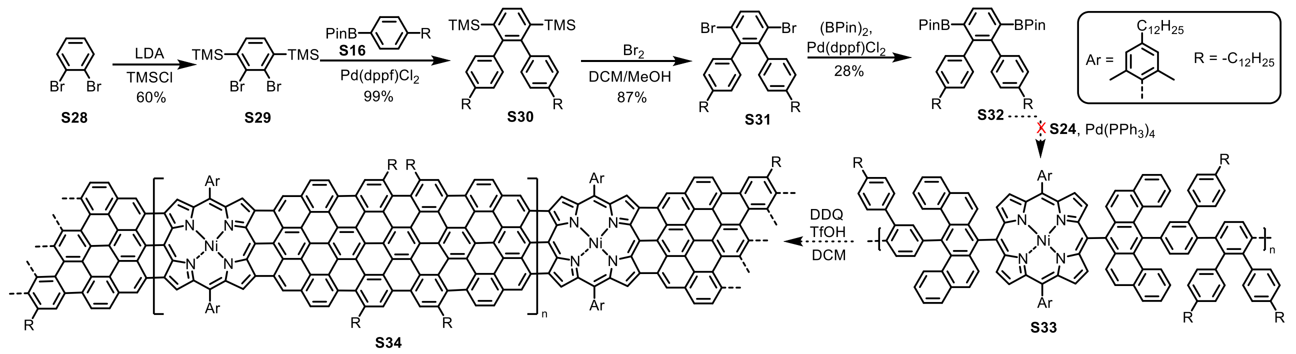

Supplementary Figure 3. Synthetic route 3 towards a porphyrin-fused 9-AGNR using Suzuki coupling strategy. The intermediate **S32** could be synthesized, however, the Suzuki coupling with arylchloride **S24** is not successful due to low reactivity of the arylchloride substrate and the steric hindrance of *ortho*-substituted phenyl group.

1.4 Synthetic Details

Synthesis of benzo[*m*]tetraphene pinacol borate ester **12**

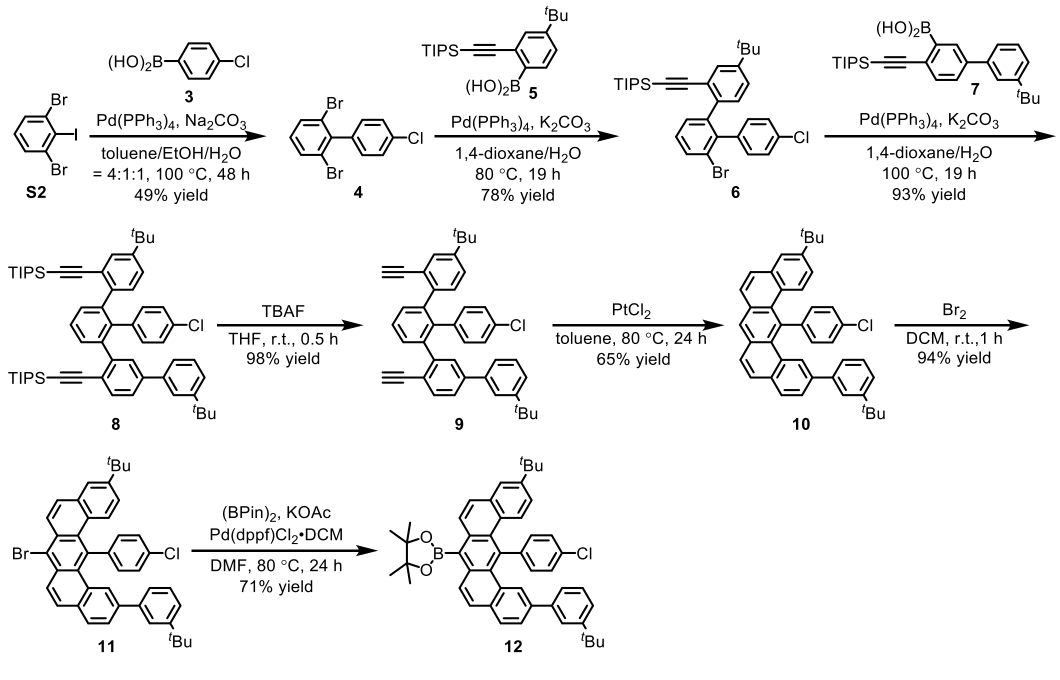

Supplementary Figure 4. Synthetic route to benzo[*m*]tetraphene pinacol borate ester **12**. TBAF: tetra-*n*-butylammonium fluoride; THF: tetrahydrofuran; DCM: dichloromethane; (BPin)$_2$: bis(pinacolato)diboron; dppf: 1,1′-bis(diphenylphosphino)ferrocene; DMF: dimethylformamide.

The key building block benzo[*m*]tetraphene pinacol borate ester **12** was synthesized using the method shown in Supplementary Figure 4. First, 1,3-dibromo-2-iodobenzene (**S2**)[7], boronic acid **5**[8] and **7**[7] were synthesized adapting the literature procedures. Suzuki coupling of **S2** and 4-chlorophenylboronic acid (**3**) gave 2,6-dibromo-4'-chloro-1,1'-biphenyl **4** in 49% yield. Then, Suzuki coupling of **4** with boronic acid **5** in 1:1 ratio provided substituted *o*-terphenyl **6** in 78% yield, which was further coupled with boronic acid **7** to provide **8** in 93% yield. After deprotection with tetra-*n*-butylammonium fluoride (TBAF) at room temperature, terminal alkyne **9** was obtained in 98% yield. Then, $PtCl_2$-catalyzed two-fold alkyne cyclization of **9** afforded benzo[*m*]tetraphene **10** with 65% yield, which was then regioselectively brominated using bromine to give **11** in 94% yield. Subsequently, Miyaura borylation of **11** provided the target compound **12** in 71% yield. The

structure of **12** has been unambiguously characterized by NMR, HR MS and X-ray single-crystal diffraction analysis.

Synthesis of 2,6-dibromo-4'-chloro-1,1'-biphenyl (**4**)

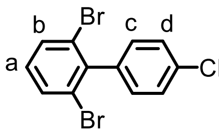

To a 250-mL Schlenk flask was added 1,3-dibromo-2-iodobenzene (**S3**) (6.00 g, 16.6 mmol), (4-chlorophenyl)boronic acid (**3**) (3.11 g, 19.9 mmol), $Pd(PPh_3)_4$ (767 mg, 0.663 mmol), and Na_2CO_3 (5.27 g, 49.8 mmol). The flask was evacuated and backfilled with argon for three times before a mixture of toluene/EtOH/H_2O (60 mL/15 mL/15 mL) was added. After degassing by bubbling with argon for 15 min, the mixture was heated at 100 °C for 48 h. Then the reaction mixture was cooled to room temperature and extracted twice with ethyl acetate (60 mL). The organic phases were combined, washed with brine (100 mL), dried over Na_2SO_4, and evaporated. The residue was purified by silica gel column chromatography (eluent: petroleum ether) to give compound **4** (2.84 g, 49% yield) as colorless oil. ^{1}H NMR (400 MHz, $CDCl_3$, 298 K) δ 7.63 (d, J = 8.1 Hz, 2H; **b**), 7.46 – 7.42 (m, 2H; **d**), 7.18 – 7.13 (m, 2H; **c**), 7.08 (t, J = 8.0 Hz, 1H; **a**); ^{13}C NMR (100 MHz, $CDCl_3$, 298 K) δ 142.0, 139.6, 134.3, 132.1, 130.8, 130.3, 128.7, 124.6; HRMS (EI, positive) *m/z*: $[M]^+$ calcd for $C_{12}H_7Br_2Cl$ 343.8598, found 343.8603.

Synthesis of {[3'-bromo-4-(*tert*-butyl)-4''-chloro-(1,1':2',1''-terphenyl)-2-yl]ethynyl}triisopropylsilane (**6**)

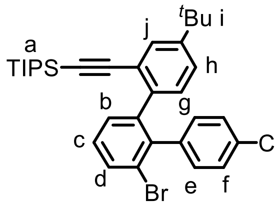

To a 250-mL Schlenk flask was added 2,6-dibromo-4'-chloro-1,1'-biphenyl (**4**) (2.47 g, 7.13 mmol), (4-(*tert*-butyl)-2-((triisopropylsilyl)ethynyl)phenyl)boronic acid (**5**) (2.68 g, 7.49 mmol), $Pd(PPh_3)_4$ (412 mg, 0.356 mmol), and K_2CO_3 (2.96 g, 21.4 mmol). The flask was evacuated and backfilled with argon for three times before addition of degassed 1,4-dioxane (48 mL) and water (12 mL). After heating at 80 °C for 19 h, the reaction mixture was cooled to room temperature, then ethyl acetate was added (20 mL). The organic phase was separated and the aqueous solution was extracted twice with ethyl acetate (20 mL). The combined organic layers were washed with brine (50 mL), dried over Na_2SO_4, and evaporated. The residue was purified by silica gel column chromatography (eluent: petroleum ether) to give compound **6** (3.22 g, 78% yield) as colorless oil. ^{1}H NMR (400 MHz, $CDCl_3$, 298 K) δ 7.64 (dd, J = 8.0, 1.3 Hz, 1H; **d**), 7.38 (d, J = 2.2 Hz, 1H; **j**), 7.32 (dd, J = 7.6, 1.3 Hz, 1H; **b**), 7.20 (t, J = 8.1 Hz, 1H; **c**), 7.17 – 7.09 (m, 4H; **e**/**f**), 7.07 (d, J = 2.4 Hz, 1H; **h**), 6.78 (d, J = 8.1 Hz, 1H; **g**), 1.26 (s, 9H; **i**), 0.99 (s, 21H; **a**); ^{13}C NMR (100 MHz, $CDCl_3$, 298 K) δ 150.0, 142.8, 141.0, 140.6, 138.6, 133.0, 132.0 (**d**), 131.5 (**e**/**f**), 130.0 (**b**), 129.6, 129.1 (**j**), 128.5 (**c**), 127.8 (**e**/**f**), 125.0 (**g**), 124.0, 122.8, 106.7 (SiC≡**C**), 93.9 (Si**C**≡C), 31.3 (**i**), 22.8 (**i**), 18.7 (**a**), 11.4 (**a**); HRMS (EI, positive) *m/z*: $[M]^+$ calcd for $C_{33}H_{41}ClBrSi$ 579.1844, found 579.1852.

Synthesis of {[3''',4-di-*tert*-butyl-2'-(4-chlorophenyl)-(1,1':3',1'':3'',1'''-quaterphenyl)-2,6''-diyl]bis(ethyne-2,1-diyl]}bis(triisopropylsilane) (**8**)

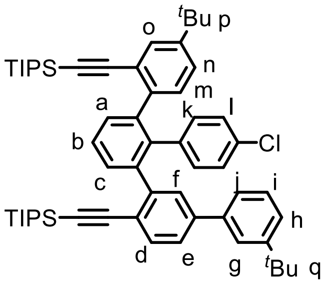

To a 250-mL Schlenk flask was added {[3'-bromo-4-(*tert*-butyl)-4''-chloro-(1,1':2',1''-terphenyl)-2-yl]ethynyl}triisopropylsilane (**6**) (4.45 g, 7.67 mmol), {3'-(*tert*-butyl)-4-[(triisopropylsilyl)ethynyl]-(1,1'-biphenyl)-3-yl}boronic acid (**7**) (3.66 g, 8.43 mmol), $Pd(PPh_3)_4$ (443 mg, 0.383 mmol), and K_2CO_3 (3.18 g, 23.0 mmol). The flask was evacuated and backfilled with argon for three times before addition of degassed 1,4-dioxane (80 mL) and water (20 mL). After heating at 100 °C for 19 h, the mixture was cooled down to room temperature and ethyl acetate was added (200 mL). The organic phase was separated, then the aqueous solution was extracted twice with ethyl acetate (100 mL). The combined organic layers were washed with brine (150 mL), dried over Na_2SO_4, and evaporated. The residue was purified by silica gel column chromatography (eluent: petroleum ether) to give compound **8** (6.32 g, 93% yield) as colorless oil. ^{1}H NMR (600 MHz, $CDCl_3$, 298 K) δ 7.58 (d, J = 8.0 Hz, 1H; **d**), 7.52 (d, J = 2.0 Hz, 1H; **o**), 7.46 (dd, J = 7.5, 1.3 Hz, 1H; **c**), 7.41 (dd, J = 7.6, 1.3 Hz, 1H; **a**), 7.36 (d, J = 7.7 Hz, 1H; **e**), 7.35 – 7.32 (m, 2H; **b**/**h**), 7.30 (t, J = 7.9 Hz, 1H; **i**), 7.17 – 7.13 (m, 2H; **j**/**g**), 7.05 (dd, J = 8.2, 2.0 Hz, 2H; **n**), 7.04 – 6.88 (m, 5H; **f**/**k**/**l**), 6.65 (d, J = 8.2 Hz, 1H; **m**), 1.32 (s, 9H; **q**), 1.30 (s, 9H; **p**), 1.05 (s, 21H; TIPS), 1.01 (s, 21H; TIPS); ^{13}C NMR (150 MHz, $CDCl_3$, 298 K) δ 151.7, 149.4, 145.5, 142.4, 140.9, 140.7, 140.5, 140.2, 138.7, 138.5, 132.7, 132.6, 131.9, 131.9, 130.8, 130.4, 130.2, 129.5, 128.9, 128.5, 127.4, 127.2, 126.6, 125.3, 125.1, 124.6, 124.3, 124.2, 123.0, 122.4, 107.4, 106.7, 94.9, 93.5, 34.9, 34.5, 31.5, 31.3, 18.8, 11.4, 11.4; HRMS (APCI, positive) *m/z*: $[M+H]^+$ calcd for $C_{60}H_{77}ClSi_2$ 889.5325, found 889.5388.

Synthesis of 3'''-(*tert*-butyl)-6'-[4-(*tert*-butyl)-2-ethynylphenyl]-4-chloro-6''-ethynyl-1,1':2',1'':3'',1'''-quaterphenyl (**9**)

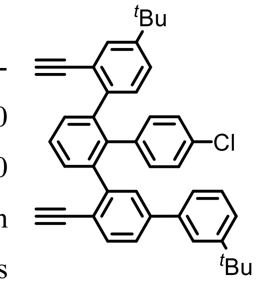

To a solution of {[3''',4-di-*tert*-butyl-2'-(4-chlorophenyl)-(1,1':3',1'':3'',1'''-quaterphenyl)-2,6''-diyl]bis(ethyne-2,1-diyl]}bis(triisopropylsilane) (**8**) (6.32 g, 7.10 mmol) dissolved in dry THF (100 mL) was added tetra-*n*-butylammonium fluoride (1.0 M in dry THF, 10.7 mL, 10.7 mmol) at room temperature. After stirring at room temperature for another 0.5 h, methanol (50 mL) was added. The solvent was concentrated to ca. 50 mL and the precipitates were collected by filtration and washed with methanol to give compound **9** (4.02 g, 98% yield) as white solid. ^{1}H NMR (400 MHz, $CDCl_3$, 298 K) δ 7.63 – 7.40 (m, 5H), 7.39 – 7.27 (m, 3H), 7.15 (s, 4H), 6.90 (s, 5H), 3.17 – 2.78 (m, 2H), 1.32 (s, 9H), 1.28 (s, 9H); ^{13}C NMR (100 MHz, $CDCl_3$, 298 K) δ 151.8, 149.8, 141.5, 140.0, 133.3, 132.7, 132.0, 129.7, 128.6, 127.2, 126.6, 125.6, 125.5, 124.8, 124.4, 124.2, 83.9, 83.3, 81.0, 79.8, 34.9, 34.5, 31.5, 31.3; HRMS (APCI, positive) *m/z*: $[M+H]^+$ calcd for $C_{42}H_{37}Cl$ 577.2657, found 577.2660.

Synthesis of dibenzo[*m*]tetraphene (**10**)

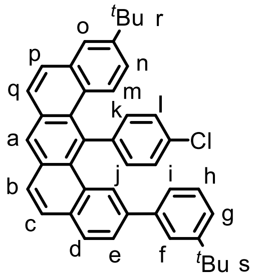

To a 250-mL Schlenk flask was added 3'''-(*tert*-butyl)-6'-[4-(*tert*-butyl)-2-ethynylphenyl]-4-chloro-6''-ethynyl-1,1':2',1'':3'',1'''-quaterphenyl (**9**) (2.40 g, 4.16 mmol) and $PtCl_2$ (276 mg, 1.04 mmol). The flask was dried under vacuum for 2 h at room temperature before toluene (100 mL) was added. After heating at 80 °C for 24 h, the mixture was cooled down to room temperature and the solvent was evaporated. The residue was purified by silica gel column chromatography (eluent: petroleum ether/dichloromethane = 4/1, *v*/*v*) to give compound **10** (1.56 g, 65% yield) as yellow solid. ^1H NMR (400 MHz, $CDCl_3$, 298 K) δ 8.35 (s, 1H; **a**), 7.88 (d, J = 7.9 Hz, 1H; **m**), 7.82 – 7.76 (m, 3H; **b**/**q**), 7.74 – 7.69 (m, 3H; **c**/**p**/**n**), 7.69 – 7.67 (m, 1H; **o**), 7.66 (d, J = 8.4 Hz, 2H; **k**), 7.51 (d, J = 8.4 Hz, 2H; **l**), 7.48 – 7.43 (m, 1H; **g**), 7.41 – 7.40 (m, 1H; **f**), 7.38 (d, J = 7.9 Hz, 1H; **g**), 7.15 (dd, J = 9.1, 2.4 Hz, 1H; **e**), 7.06 (d, J = 9.0 Hz, 1H; **d**), 6.90 – 6.84 (m, 1H; **i**), 1.38 (s, 9H; **s**), 1.35 (s, 9H; **r**); ^{13}C NMR (100 MHz, $CDCl_3$, 298 K) δ 151.5, 149.1, 144.4, 141.0, 138.1, 137.2, 133.5, 133.1 (**l**), 131.7, 131.6, 131.5, 131.3 (**k**), 129.1, 128.9 (**m**), 128.9, 128.7 (**h**/**d**), 128.4, 128.3, 128.2 (**a**), 127.8, 127.1 (**b**/**q**), 127.0 (**b**/**q**), 125.6, 124.9 (**i**), 124.8 (**j**), 124.5 (**f**), 124.3 (**g**), 123.1 (**e**), 34.9 (**r**/**s**), 34.6 (**r**/**s**), 31.6 (**r**/**s**), 31.4 (**r**/**s**); MALDI-TOF MS (positive): *m/z*: $[M+H]^+$ calcd for $C_{42}H_{37}Cl$ 577.2657, found 577.2655; UV-vis (chloroform, 298 K): λ (ε) = 321 nm (7.36×10^4 M^{-1} cm^{-1}), 352 nm (2.16×10^4 M^{-1} cm^{-1}), and 367 nm (1.41×10^4 M^{-1} cm^{-1}).

Synthesis of brominated dibenzo[*m*]tetraphene **11**

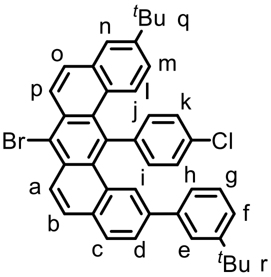

To a solution of 11-(*tert*-butyl)-2-(3-(*tert*-butyl)phenyl)-14-(4-chlorophenyl)benzo[*m*]tetraphene (**10**) (500 mg, 0.866 mmol) in dry dichloromethane (50 mL) was added bromine (0.15 g, 0.91 mmol) dissolved in dichloromethane (1.0 mL) at room temperature. After stirring for 1 h, saturated aqueous solution of $Na_2S_2O_3$ (20 mL) was added to quench the excess bromine. The organic phase was separated and the aqueous phase was extracted with dichloromethane (30 mL) for three times. The combined organic phases were washed with brine (100 mL), dried over Na_2SO_4, and evaporated. The residue was purified by silica gel column chromatography (eluent: petroleum ether/dichloromethane = 8/1, *v*/*v*) to give compound **11** (0.536 g, 94% yield) as yellow solid. ^1H NMR (400 MHz, $CDCl_3$, 298 K) δ 8.38 (dd, J = 9.2, 2.4 Hz, 2H; **a**/**p**), 7.88 (d, J = 8.1 Hz, 1H; **l**), 7.81 – 7.77 (m, 2H; **b**/**o**), 7.76 (s, 1H; **i**), 7.68 (dd, J = 8.1, 1.7 Hz, 1H; **m**), 7.65 – 7.62 (m, 1H; **n**), 7.59 – 7.54 (m, 2H; **j**), 7.47 – 7.42 (m, 2H; **k**), 7.39 (td, J = 7.5, 0.8 Hz, 1H; **g**), 7.37 – 7.33 (m, 2H; **e**/**f**), 7.09 (dd, J = 9.1, 2.1 Hz, 1H; **d**), 7.05 (d, J = 9.1 Hz, 1H; **c**), 6.79 (dt, J = 7.4, 1.6 Hz, 1H; **h**), 1.38 (s, 9H; **r**), 1.34 (s, 9H; **q**); ^{13}C NMR (100 MHz, $CDCl_3$, 298 K) δ 151.6, 149.8, 147.6, 146.1, 144.4, 144.3, 143.7, 140.8, 138.0, 136.2, 134.8, 134.1, 133.6, 133.1, 131.1, 130.8, 130.7, 130.6, 130.2, 129.4, 129.3, 129.1, 128.9, 128.7, 128.6, 128.2, 126.2, 126.1, 126.06, 125.0, 124.4, 124.3, 123.0, 34.9, 34.7, 31.6, 31.4; HRMS (APCI, positive) *m/z*: $[M+H]^+$ calcd for $C_{42}H_{36}BrCl$ 655.1762, found 655.1819; UV-vis (chloroform, 298 K): λ (ε) = 319 nm (5.86×10^4 M^{-1} cm^{-1}), 331 nm (8.04×10^4 M^{-1} cm^{-1}), 364 nm (2.54×10^4 M^{-1} cm^{-1}), and 380 nm (1.78×10^4 M^{-1} cm^{-1}).

Synthesis of benzo[*m*]tetraphene pinacol borate ester **12**

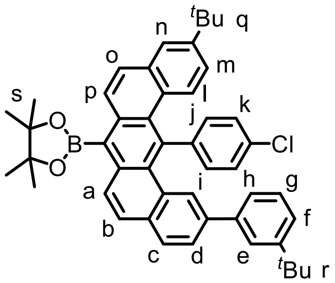

To a 250-mL Schlenk flask was added brominated dibenzo[*m*]tetraphene **11** (1.20 g, 1.83 mmol), bis(pinacolato)diboron (928.9 mg, 3.658 mmol), $Pd(dppf)Cl_2{\cdot}CH_2Cl_2$ (149.4 mg, 182.9 μmol), and KOAc (1.08 g, 11.0 mmol). The flask was evacuated and backfilled with argon for three times before degassed DMF (60 mL) was added. The mixture was heated at 80 °C for 24 h. After cooling to room temperature, ethyl acetate (120 mL) and water (120 mL) were added and the organic phase was separated. The aqueous phase was extracted twice with ethyl acetate (50 mL). The combined organic layers were washed with brine (120 mL), dried over Na_2SO_4, and evaporated. The residue was purified by silica gel column chromatography (eluent: *n*-hexane/ethyl acetate = 4/1 to 2/1, *v*/*v*) to give compound **12** (908 mg, 71% yield) as white solid. ^{1}H NMR (400 MHz, $CDCl_3$, 298 K) δ 8.12 – 8.06 (m, 2H; **a**/**p**), 7.86 (d, J = 8.7 Hz, 1H; **l**), 7.76 (s, 1H; **i**), 7.74 – 7.69 (m, 2H; **b**/**o**), 7.68 (d, J = 7.1 Hz, 2H; **m**/**n**), 7.60 (d, J = 8.4 Hz, 2H; **j**), 7.48 (d, J = 8.6 Hz, 2H; **k**), 7.45 – 7.39 (m, 2H; **e**/**g**), 7.37 (dd, J = 7.5, 1.8 Hz, 1H; **f**), 7.08 (d, J = 1.8 Hz, 2H; **c**/**d**), 6.83 (dt, J = 7.6, 1.5 Hz, 1H; **h**), 1.63 (s, 12H; **s**), 1.40 (s, 9H; **r**), 1.37 (s, 9H; **q**); ^{13}C NMR (101 MHz, $CDCl_3$, 298 K) δ 151.4, 149.0, 144.4, 141.0, 138.0, 137.4, 135.1, 135.0, 134.4, 134.0, 133.6, 133.1, 131.4, 130.9, 129.3, 128.9, 128.6, 128.4, 127.8, 127.6, 127.6, 126.6, 126.5, 125.4, 125.0, 124.4, 124.2, 124.0, 122.4, 84.8, 34.9, 34.6, 31.6, 31.4, 25.4; HRMS (EI, positive) *m/z*: $[M+H]^+$ calcd for $C_{48}H_{48}BClO_2$ 702.3545, found 702.3577; UV-vis (chloroform, 298 K): λ (ε) = 314 nm (6.22×10^4 M^{-1} cm^{-1}), 326 nm (8.16×10^4 M^{-1} cm^{-1}), 358 nm (2.50×10^4 M^{-1} cm^{-1}), 373 nm (1.85×10^4 M^{-1} cm^{-1}), and 416 nm (1.84×10^3 M^{-1} cm^{-1}). **12** was further characterised by X-ray crystallographic analysis. CCDC: 2225521

Synthesis of porphyrin-benzo[*m*]tetraphene conjugates

5,15-Dibromo-10,20-dimesitylporphyrin (Ni) (**18a**) was synthesized using the literature procedure[7]. 5,15-Dibromo-10,20-bis(2,6-dimethyl-4-dodecylphenyl)porphyrin (Ni) (**18b**) was synthesized using the method shown in Supplementary Figure 5: the iodo-magnesium exchange of 5-bromo-2-iodo-1,3-dimethylbenzene (**13**) with *i*-PrMgCl·LiCl followed by nucleophilic addition with DMF and hydrolysis provided 4-bromo-2,6-dimethylbenzaldehyde (**14**) in 95% yield. After Suzuki coupling with 1-dodecylboronicacid pinacol ester, generated by reaction of 1-dodecene and 9-BBN, 4-dodecyl-2,6-dimethylbenzaldehyde (**15**) was obtained in 85% yield. The condensation of **15** with dipyrromethane provided free base porphyrin (**16**) in 32% yield, which is a little higher than the yield of synthesizing dimesitylporphyrin (25% yield). After metalation with $Ni(OAc)_2{\cdot}4H_2O$ and selective bromination, dibromoporphyrin **18** could be obtained in high yield of 87% over two steps. The other key building block 2,6-dimethyl-4-dodecylphenylboronic acid pinacol borate ester (**22b**) was synthesized. The first step is Sandmeyer iodination of commercially available starting material 4-bromo-3,5-dimethylaniline (**19**) to provide 2-bromo-5-iodo-1,3-dimethylbenzene (**20**) in 24% yield. Next, regioselective Suzuki coupling of **20** with 1-dodecylboronic acid pinacol ester generated in situ by reaction of 1-dodecene and 9-BBN provided 2-bromo-5-dodecyl-1,3-dimethylbenzene (**21**) in 48% yield. Then, Miyaura borylation of **21** afforded pinacol borate ester **22b** in 97% yield. Suzuki coupling of dibromoporphyrin **18** and dibenzo[*m*]tetraphene pinacol borate ester **12** gave both one-fold and two-fold coupling products **23** and **2**. The

singly-coupled product **23** was then selectively brominated using NBS to give *meso*-bromoporphyrin **24** in 93–97% yield, which underwent Suzuki coupling with **22** to provide triarylporphyrin **1** in 66–76% yield.

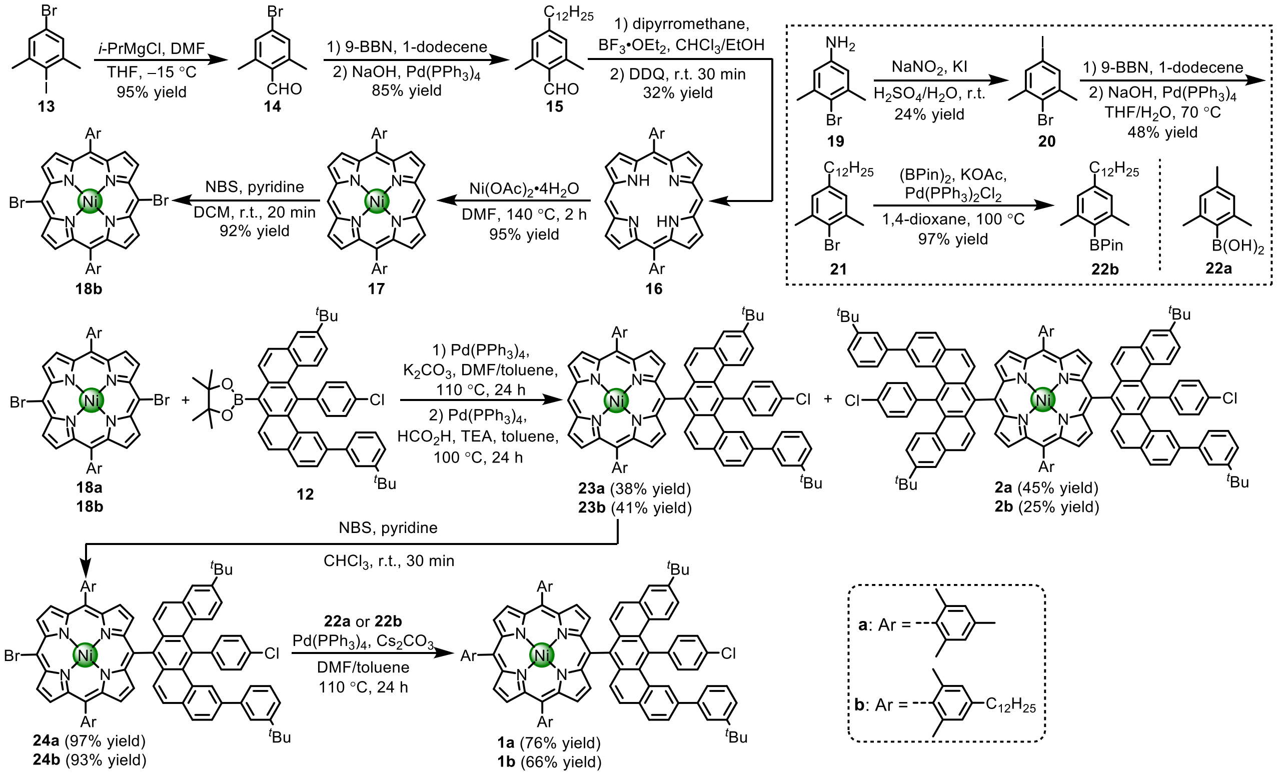

Supplementary Figure 5. Synthetic route to porphyrin-benzo[*m*]tetraphene conjugates **1** and **2**. DMF: *N*,*N*-dimethylformamide; THF: tetrahydrofuran; 9-BBN: 9-borabicyclo[3.3.1]nonane; DDQ: 2,3-dichloro-5,6-dicyanobenzoquinone; TEA: triethylamine; NBS: *N*-bromosuccinimide.

Synthesis of 4-bromo-2,6-dimethylbenzaldehyde (**14**)

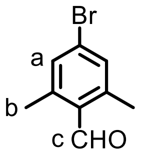

To a stirred solution of 5-bromo-2-iodo-1,3-dimethylbenzene (**13**) (12.73 g, 40.94 mmol) dissolved in dry THF (180 mL) was added *i*-PrMgCl·LiCl solution in THF (2.0 M, 100 mL, 200 mmol) dropwise at –5 °C under argon atmosphere. After stirring the mixture at this temperature for 1 h, dry DMF (20 mL, 0.26 mol) was slowly added. The solution was stirred at –5 °C for 1.5 h, then warmed up to room temperature and stirred for another 1 h. The reaction was quenched by adding 1.0 M HCl solution (360 mL, 360 mmol) and the organic layer was extracted with ethyl acetate (300 mL) twice. The combined organic layer was washed with brine (150 mL), dried over anhydrous Na_2SO_4 and evaporated under reduced pressure. The residue was purified by silica gel column chromatography (eluent: ethyl acetate/*n*-hexane = 1/20, *v*/*v*). After removal of solvents, compound **14** (8.08 g, 95% yield) was obtained as white solid. ^{1}H NMR (300 MHz, tetrahydrofuran-d_8, 298 K) δ 10.52 (s, 1H; **c**), 7.32 (s, 2H; **a**), 2.56 (s, 6H; **b**); ^{13}C NMR (75 MHz, tetrahydrofuran-d_8, 298 K) δ 192.8, 144.0, 133.3, 132.7, 127.9, 20.3; HR MS (APCI, positive) *m/z*: $[M+H]^+$ calcd for $C_9H_{10}BrO$ 212.9910, found 212.9913.

Synthesis of 4-dodecyl-2,6-dimethylbenzaldehyde (**15**)

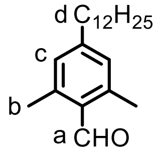

A solution of 9-BBN (0.50 M in THF, 60 mL, 30 mmol) was added to 1-dodecene (4.8 g, 29 mmol) in a 250-mL Schlenk flask at room temperature under argon atmosphere. After stirring for 12 h, the reaction was treated with aqueous solution of NaOH (3.0 M, 40 mL, 120 mmol) and diluted with THF (60 mL). Then 4-bromo-2,6-dimethylbenzaldehyde (**14**) (4.588 g, 21.53 mmol) and $Pd(PPh_3)_4$ (1.61 g, 1.39 mmol) were added and the resulting reaction mixture was heated at 80 °C for 4 h. The mixture was cooled to room temperature and neutralized with saturated $NaHCO_3$ solution (20 mL), then extracted with ethyl acetate (100 mL) for two times. The organic layers were combined, washed with brine, dried over anhydrous Na_2SO_4 and evaporated. The residue was purified by silica gel column chromatography (eluent: petroleum ether) to give compound **15** (5.54 g, 85% yield) as white solid. ^{1}H NMR (300 MHz, CD_2Cl_2, 298 K) δ 10.55 (s, 1H; **a**), 6.92 (s, 2H; **c**), 2.67 – 2.51 (m, 8H; **b**/**d**), 1.69 – 1.54 (m, 2H; **d**), 1.38 – 1.20 (m, 18H; **d**), 0.88 (t, J = 6.5 Hz, 3H; **d**); ^{13}C NMR (75 MHz, CD_2Cl_2) δ 193.2, 149.2, 141.7, 130.7, 130.2, 36.3, 32.4, 31.4, 30.1, 30.1, 30.0, 29.9, 29.8, 29.7, 23.1, 20.7, 14.3; HR MS (APCI, positive): *m/z*: $[M+H]^+$ calcd for $C_{21}H_{34}O$ 303.2682, found 303.2689.

Synthesis of 5,15-bis(2,6-dimethyl-4-dodecylphenyl)porphyrin (**16**)

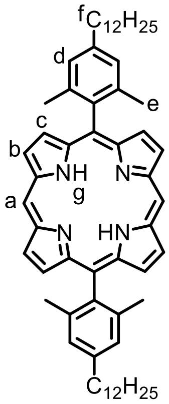

A solution of dipyrromethane (450 mg, 3.08 mmol) and 4-dodecyl-2,6-dimethylbenzaldehyde (**15**) (931 mg, 3.08 mmol) in chloroform (300 mL) containing ethanol (15 mL) was degassed by bubbling with argon for 10 min. After addition of $BF_3{\cdot}OEt_2$ (0.13 mL), the resulting solution was stirred in dark for 3 h. 2,3-Dichloro-5,6-dicyano-1,4-benzoquinone (1.04 g, 4.56 mmol) was then added in one portion and the resulting mixture was stirred at room temperature for another 1 h. The reaction was subsequently quenched with triethyl amine (4 mL) and passed through a pad of silica gel (eluent: dichloromethane). The solution was concentrated in vacuo and the residue was purified by silica gel column chromatography (eluent: petroleum ether/dichloromethane = 3/1, *v*/*v*) and recrystallized from dichloromethane and methanol to give compound **16** (417 mg, 32% yield) as purple solid. ^{1}H NMR (600 MHz, $CDCl_3$, 298 K) δ 10.22 (s, 2H; **a**), 9.33 (d, J = 4.5 Hz, 4H; **b**), 8.89 (d, J = 4.5 Hz, 4H; **c**), 7.32 (s, 4H; **d**), 2.98 – 2.85 (m, 4H; **f**), 1.94 (t, J = 7.9 Hz, 4H; **f**), 1.86 (s, 12H; **e**), 1.64 – 1.56 (m, 4H; **f**), 1.56 – 1.49 (m, 4H; **f**), 1.49 – 1.29 (m, 28H; **f**), 0.92 (t, J = 6.9 Hz, 6H; **f**), –3.04 (s, 2H; **g**); ^{13}C NMR (151 MHz, $CDCl_3$, 298 K) δ 147.0, 145.5, 143.1, 139.5, 137.9, 131.9, 130.2, 127.2, 117.6, 104.7, 36.3, 32.1, 31.9, 30.0, 29.9, 29.9, 29.9, 29.6, 22.9, 21.9, 14.3; HR MS (APCI, positive) *m/z*: $[M+H]^+$ calcd for $C_{60}H_{78}N_4$ 855.6299, found 855.6292; UV-vis (chloroform, 298 K): λ (ε) = 407 nm (3.56×10^5 M^{-1} cm^{-1}), 502 nm (1.79×10^4 M^{-1} cm^{-1}), 531 nm (4.13×10^4 M^{-1} cm^{-1}), 574 nm (6.07×10^4 M^{-1} cm^{-1}), and 629 nm (1.44×10^4 M^{-1} cm^{-1}).

Synthesis of 5,15-bis(2,6-dimethyl-4-dodecylphenyl)porphyrin (Ni) (**17**)

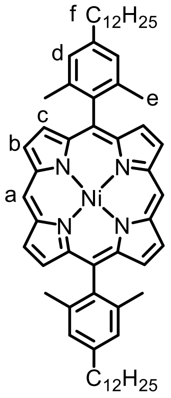

A mixture of 5,15-bis(2,6-dimethyl-4-dodecylphenyl)porphyrin (**16**) (200 mg, 0.234 mmol) and $Ni(OAc)_2 \cdot 4H_2O$ (780 mg, 4.41 mmol) in dry DMF (25 mL) was heated at 140 °C for 2 h. After completion of the reaction, the mixture was cooled down to room temperature. Methanol (50 mL) was added and the suspension was stirred overnight. The precipitates were filtered and washed with methanol (20 mL) to give compound **17** (405 mg, 95% yield) as purple solid. ^{1}H NMR (600 MHz, $CDCl_3$, 298 K) δ 9.90 (s, 2H; **a**), 9.15 (d, J = 4.6 Hz, 4H; **b**), 8.78 (d, J = 4.6 Hz, 4H; **c**), 7.24 (s, 4H; **d**), 2.85 (t, J = 7.8 Hz, 4H; **f**), 1.93 – 1.85 (m, 4H; **f**), 1.80 (s, 12H; **e**), 1.60 – 1.54 (m, 4H; **f**), 1.51 – 1.46 (m, 4H; **f**), 1.45 – 1.28 (m, 28H; **f**), 0.91 (t, J = 7.0 Hz, 6H; **f**); ^{13}C NMR (150 MHz, $CDCl_3$, 298 K) δ 143.0, 142.9, 142.8, 139.2, 137.7, 132.5, 131.5, 127.2, 117.0, 104.9, 36.2, 32.1, 31.9, 30.0, 29.9, 29.9, 29.9, 29.9, 29.6, 22.9, 21.6, 14.3; HR MS (APCI, positive) *m/z*: $[M+H]^+$ calcd for $C_{60}H_{77}N_4Ni$ 911.5496, found 911.5492; UV-vis (chloroform, 298 K): λ (ε) = 401 nm (2.49×10^5 M^{-1} cm^{-1}), 516 nm (1.76×10^4 M^{-1} cm^{-1}), and 549 nm (9.32×10^4 M^{-1} cm^{-1}).

Synthesis of 5,15-dibromo-10,20-bis(2,6-dimethyl-4-dodecylphenyl)porphyrin (Ni) (**18b**)

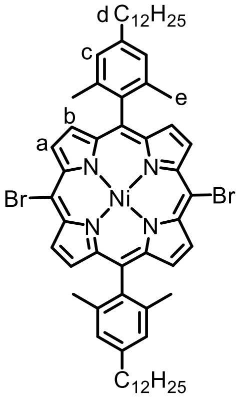

To a solution of 5,15-bis(2,6-dimethyl-4-dodecylphenyl)porphyrin (Ni) (**17**) (330 mg, 0.362 mmol) dissolved in dichloromethane (100 mL) and pyridine (1.0 mL) was added *N*-bromosuccinimide (NBS) (132 mg, 0.742 mmol). After stirring for 20 min at room temperature, acetone (15 mL) was added to quench the reaction and the solvents were evaporated under reduced pressure. The residue was purified by silica gel column chromatography (eluent: petroleum ether/dichloromethane = 10/1, *v*/*v*). After concentration in vacuo, the residue was recrystallized from dichloromethane and methanol to give compound **18b** (305 mg, 92% yield) as purple solid. ^{1}H NMR (600 MHz, $CDCl_3$, 298 K) δ 9.43 (d, J = 4.9 Hz, 4H; **a**), 8.58 (d, J = 4.9 Hz, 4H; **b**), 7.21 (s, 4H; **c**), 2.82 (t, J = 7.8 Hz, 4H; **d**), 1.86 (p, J = 7.6 Hz, 4H; **d**), 1.81 (s, 12H; **e**), 1.57 – 1.52 (m, 4H; **d**), 1.49 – 1.44 (m, 4H; **d**), 1.42 – 1.27 (m, 28H; **d**), 0.91 (t, J = 6.9 Hz, 6H; **d**); ^{13}C NMR (150 MHz, $CDCl_3$, 298 K) δ 143.4, 143.1, 139.0, 136.7, 134.0, 132.9, 127.3, 118.8, 102.7, 36.2, 32.1, 31.8, 29.9, 29.9, 29.9, 29.8, 29.6, 22.9, 21.5, 14.3; MALDI-TOF MS (positive) *m/z*: $[M]^+$ calcd for $C_{60}H_{74}Br_2N_4Ni$, 1066.36, found 1066.39; UV-vis (chloroform, 298 K): λ (ε) = 419 nm (2.52×10^5 M^{-1} cm^{-1}), and 534 nm (1.91×10^4 M^{-1} cm^{-1}).

Synthesis of 2-bromo-5-iodo-1,3-dimethylbenzene (**20**)

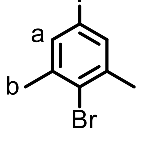

To a solution of 4-bromo-3,5-dimethylaniline (**19**) (5.00 g, 25.0 mmol) in aqueous sulfuric acid (225 mL, 6.0 M, 1.35 mol) was added a solution of sodium nitrite (3.45 g, 50.0 mmol) in water (20 mL) dropwise over a period of 10 min at –10 °C. After addition, the resulting mixture was stirred at –10 °C for an additional 15 min. Then, a solution of potassium iodide (8.30 g, 50.0 mmol) in water (20 mL) was slowly added to the mixture over a period of 5 min. The reaction mixture was stirred at –10 °C for 15 min, then stirred at 0 °C for 2 h. After stirring the mixture overnight at room temperature, the resulting solution was neutralized by adding Na_2CO_3 to pH = 7 and subsequently extracted with Et_2O (100

mL) for four times. The combined organic layers were washed with water (200 mL), aqueous Na_2SO_3 (1 M, 50 mL × 2), aqueous NaOH (2.5 M, 50 mL ×2), brine (100 mL), and dried over anhydrous $MgSO_4$. After filtration, the filtrate was evaporated to dryness. The residue was purified by silica gel column chromatography (eluent: *n*-hexane) to give compound **20** (1.82 g, 24% yield) as colorless oil. ^{1}H NMR (400 MHz, $CDCl_3$, 298 K) δ 7.40 (s, 2H; **a**), 2.36 (s, 6H; **b**); ^{13}C NMR (100 MHz, $CDCl_3$, 298 K) δ 140.6, 136.9, 128.3, 127.8, 23.6; HR MS (EI, positive) *m/z*: $[M+H]^+$ calcd for C_8H_8BrI 309.8849, found 309.8854.

<u>Synthesis of 2-bromo-5-dodecyl-1,3-dimethylbenzene (**21**)</u>

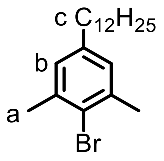

To a solution of 9-BBN (9.65 mL, 0.50 M in THF, 4.82 mmol) was added 1-dodecene (1.07 mL, 4.82 mmol) at room temperature under argon atmosphere. After stirring for 12 h, 2-bromo-5-iodo-1,3-dimethylbenzene (**20**) (1.50 g, 4.82 mmol), $Pd(PPh_3)_4$ (279 mg, 241 µmol), K_2CO_3 (1.33 g, 9.65 mmol), THF (10 mL) and degassed water (2 mL) were added. The reaction mixture was refluxed under argon for 12 h, and then cooled to room temperature. Water (20 mL) was added and the mixture was extracted twice with ethyl acetate (50 mL). The organic layers were combined, washed with brine and dried over anhydrous $MgSO_4$. After removal of the solvent under reduced pressure, the residue was purified by silica gel column chromatography (eluent: petroleum ether) and size exclusion column chromatography (Bio-Beads S-X3, eluent: chloroform) to give compound **21** (0.81 g, 48% yield) as colorless oil. ^{1}H NMR (400 MHz, $CDCl_3$, 298 K) δ 6.89 (s, 2H; **b**), 2.49 (t, J = 8.0 Hz, 2H; **c**), 2.39 (s, 6H; **a**), 1.62 – 1.51 (m, 2H; **c**), 1.35 – 1.22 (m, 18H; **c**), 0.89 (t, J = 6.8 Hz, 3H; **c**); ^{13}C NMR (100 MHz, $CDCl_3$, 298 K) δ 141.6, 138.0, 128.5, 124.6, 35.4, 32.1, 31.6, 29.8, 29.8, 29.8, 29.7, 29.7, 29.51, 29.48, 23.93, 22.85, 14.3; HR MS (EI, positive) *m/z*: $[M+H]^+$ calcd for $C_{20}H_{33}Br$ 352.1760, found 352.1766.

<u>Synthesis of 2,6-dimethyl-4-dodecylphenylboronic acid pinacol ester (**22b**)</u>

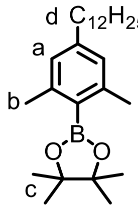

To a 25-mL Schlenk tube was added 2-bromo-5-dodecyl-1,3-dimethylbenzene (**21**) (500 mg, 1.41 mmol), bis(pinacolato)diboron (719 mg, 2.83 mmol), $Pd(PPh_3)_2Cl_2$ (99.3 mg, 141 µmol) and KOAc (833 mg, 8.49 mmol). The reaction tube was evacuated and backfilled with argon for three times before 1,4-dioxane (5.00 mL) was added. The mixture was degassed by three times freeze-pump-thaw cycles and heated at 100 °C for 24 h. After completion of the reaction, the reaction mixture was cooled to room temperature and diluted with ethyl acetate (50 mL), washed with water, brine, and dried over anhydrous Na_2SO_4. The solvent was evaporated under reduced pressure and the residue was purified by silica gel column chromatography (eluent: petroleum ether) to give compound **22b** (550 mg, 97 %) as colorless oil. ^{1}H NMR (400 MHz, $CDCl_3$, 298 K) δ 6.76 (s, 2H; **a**), 2.48 (t, J = 7.5 Hz, 2H; **d**), 2.38 (s, 6H; **b**), 1.60 – 1.48 (m, 2H; **d**), 1.37 (s, 12H; **c**), 1.31 – 1.21 (m, 18H; **d**), 0.91 – 0.86 (t, J = 7.3 Hz, 3H; **d**); ^{13}C NMR (101 MHz, $CDCl_3$, 298 K) δ 144.2, 142.2, 127.0, 83.6, 36.0, 32.1, 31.5, 29.8, 29.8, 29.8, 29.7, 29.5, 29.5, 25.1, 22.8, 22.4, 14.3; HRMS (ESI, positive) *m/z*: $[M+H]^+$ calcd for $C_{26}H_{46}BO_2$ 401.3585, found 401.3579.

Synthesis of porphyrin-benzo[*m*]tetraphene conjugates **23a** and **2a**

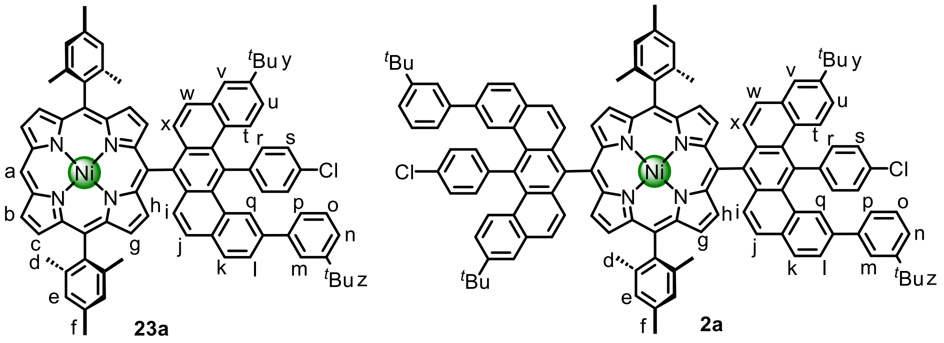

23a **2a**

To a 50-mL Schlenk tube was added 5,15-dibromo-10,20-dimesitylporphyrin **18a** (30 mg, 39 μmol), benzo[*m*]tetraphene pinacol borate ester **12** (83 mg, 0.12 mmol), $Pd(PPh_3)_4$ (9.11 mg, 7.88 μmol), and K_2CO_3 (32 mg, 0.24 mmol). A mixture of toluene/DMF (6 mL/6 mL) was added after three cycles of evacuation and backfilling with argon. The mixture was heated at 110 °C for 12 h, and another portion of $Pd(PPh_3)_4$ (9.11 mg, 7.88 μmol) was added. The resulting mixture was heated at 110 °C for 24 h. After cooling to room temperature, the red solution was diluted with ethyl acetate (50 mL), washed with brine (30 mL), dried over Na_2SO_4, and evaporated. The residue was transferred into a 25-mL Schlenk tube. To the tube was added $Pd(PPh_3)_4$ (9.0 mg, 7.8 μmol), triethylamine (30 μL, 0.22 mmol), formic acid (30 μL, 0.80 mmol) and toluene (10 mL). The mixture was heated at 100 °C for 2 h under argon atmosphere, and then cooled to room temperature. After evaporation of the solvent, the residue was purified by silica gel column chromatography (eluent: petroleum ether/dichloromethane = 5/1 to 4/1, *v*/*v*) to give one-side coupling product **23a** (17.5 mg, 38% yield) and two-sides coupling product **2a** (30.9 mg, 45% yield) as red solid. **23a**: ^{1}H NMR (600 MHz, $CDCl_3$, 298 K) δ 9.87 (s, 1H; **a**), 9.14 (d, J = 4.7 Hz, 2H; **b**), 8.74 (d, J = 4.7 Hz, 2H; **c**), 8.49 (d, J = 4.8 Hz, 2H; **g**), 8.34 (d, J = 4.8 Hz, 2H; **h**), 7.91 (s, 1H; **q**), 7.77 (d, J = 8.4 Hz, 2H; **r**), 7.72 (d, J = 8.3 Hz, 2H; **s**), 7.70 (d, J = 8.1 Hz, 1H; **k**), 7.66 (dd, J = 8.0, 1.6 Hz, 1H; **l**), 7.58 (d, J = 2.2 Hz, 1H; **v**), 7.46 (t, J = 7.7 Hz, 1H; **o**), 7.42 (t, J = 1.6 Hz, 1H; **m**), 7.41 – 7.37 (m, 1H; **n**), 7.33 (d, J = 9.3 Hz, 1H; **t**), 7.19 (s, 4H; **e**), 7.19 – 7.14 (m, 3H; **u**/**j**/**w**), 6.93 (d, J = 7.6 Hz, 1H; **p**), 6.87 – 6.83 (m, 2H; **i**/**x**), 2.55 (s, 6H; **f**), 1.81 (d, J = 2.2 Hz, 12H; **d**), 1.37 (s, 9H; **z**), 1.35 (s, 9H; **y**); ^{13}C NMR (150 MHz, $CDCl_3$, 298 K) δ 151.5, 149.4, 144.5, 144.1, 143.12, 143.11, 142.8, 141.1, 139.19, 139.18, 138.0, 137.9, 137.8, 137.4, 137.3, 135.7, 134.7, 134.4, 134.3, 133.8, 133.8, 132.9, 132.72, 132.65, 131.8, 131.6, 131.2, 131.1, 129.6, 129.3, 129.2, 128.7, 128.5, 128.4, 127.9, 127.6, 127.5, 127.3, 127.0, 126.9, 125.8, 125.5, 125.1, 124.6, 124.4, 124.3, 122.9, 117.5, 115.1, 105.0, 34.94, 34.7, 31.6, 31.4, 29.9, 21.61, 21.58, 21.57, 21.53; MALDI-TOF MS (positive) *m/z*: $[M]^+$ calcd for $C_{80}H_{67}ClN_4Ni$, 1176.44, found 1176.49; UV-vis (chloroform, 298 K): λ (ε) = 325 nm (7.43 × 10^4 M^{-1} cm^{-1}), 412 nm (2.48 × 10^5 M^{-1} cm^{-1}) , 522 nm (2.13 × 10^4 M^{-1} cm^{-1}), and 554 nm (7.48 × 10^4 M^{-1} cm^{-1}). **2a**: ^{1}H NMR (600 MHz, CD_2Cl_2, 298 K) δ 8.48 (d, J = 4.9 Hz, 4H; **g**), 8.38 (d, J = 4.9 Hz, 4H; **h**), 7.94 (s, 2H; **q**), 7.80 (d, J = 8.2 Hz, 4H; **r**), 7.78 – 7.75 (m, 6H; **s**/**k**), 7.71 (dt, J = 8.0, 1.4 Hz, 2H; **l**), 7.66 (t, J = 2.2 Hz, 2H; **v**), 7.48 – 7.44 (m, 4H; **o**/**m**), 7.41 (d, J = 8.0 Hz, 2H; **n**), 7.36 (d, J = 9.2 Hz, 2H; **t**), 7.30 – 7.45 (m, 4H; **j**/**w**), 7.24 – 7.21 (m, 2H; **u**), 7.16 (s, 4H), 6.98 – 6.94 (m, 4H), 6.96 (d, J = 9.2 Hz, 2H; **p**), 6.96 – 6.89 (m, 4H; **i**/**x**), 2.48 (s, 6H; **e**), 1.85 (t, J = 4.0 Hz, 12H; **c**), 1.38 (s, 18H; **z**), 1.36 (d, J = 1.1 Hz, 18H; **y**); ^{13}C NMR (150 MHz, CD_2Cl_2, 298 K) δ 152.0, 149.9, 144.9, 144.6, 143.3, 141.2, 139.2, 138.3, 138.2, 137.9, 137.3, 135.7, 135.0, 134.6, 134.6, 134.6, 134.5, 134.1, 133.2, 133.0, 132.2,

131.5, 131.5, 129.9, 129.4, 129.0, 128.9, 128.8, 128.7, 128.1, 128.0, 127.9, 127.6, 127.0, 126.8, 126.8, 126.1, 125.2, 124.8, 124.7, 123.2, 118.4, 115.6, 35.1, 34.9, 31.6, 31.4, 21.5, 21.5; MALDI-TOF MS (positive) *m/z*: $[M]^+$ calcd for $C_{122}H_{102}Cl_2N_4Ni$ 1750.68, found 1750.77; UV-vis (dichloromethane, 298 K): λ (ε) = 324 nm (1.46×10^5 M^{-1} cm^{-1}), 423 nm (2.59×10^5 M^{-1} cm^{-1}), and 531 nm (3.20×10^4 M^{-1} cm^{-1}).

Synthesis of *meso*-bromoporphyrin **24a**

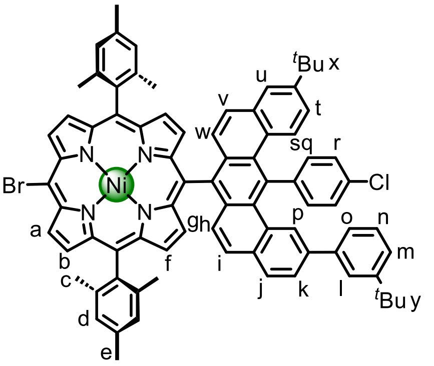

To a solution of porphyrin **23a** (42 mg, 36 μmol) dissolved in chloroform (15 mL) and pyridine (0.3 mL) was added *N*-bromosuccinimide (7.6 mg, 43 μmol). After stirring at room temperature for 60 min, acetone (1.5 mL) was added to quench the reaction. The solvents were evaporated and the residue was purified by silica gel column chromatography (eluent: dichloromethane/petroleum ether = 1/2 *v*/*v*). After concentration in vacuo, the residue was recrystallized from dichloromethane and methanol to give compound **24a** (43 mg, 97% yield) as red solid. ^{1}H NMR (600 MHz, CD_2Cl_2, 298 K) δ 9.54 (d, *J* = 4.9 Hz, 2H; **a**), 8.64 (d, *J* = 4.9 Hz, 2H; **b**), 8.41 (d, *J* = 4.9 Hz, 2H; **f**), 8.29 (d, *J* = 4.8 Hz, 2H; **g**), 7.91 (s, 1H; **p**), 7.76 (d, *J* = 8.2 Hz, 2H; **q**), 7.74 (d, *J* = 8.4 Hz, 2H; **r**), 7.72 (d, *J* = 8.2 Hz, 1H; **j**), 7.67 (d, *J* = 8.0 Hz, 1H; **k**), 7.61 (d, *J* = 1.9 Hz, 1H; **u**), 7.47 – 7.42 (m, 2H; **n/l**), 7.39 (d, *J* = 7.9 Hz, 1H; **m**), 7.32 (d, *J* = 9.2 Hz, 1H; **s**), 7.22 (d, *J* = 9.3 Hz, 1H; **i** or **v**), 7.20 (s, 4H; **d**), 7.19 (d, *J* = 2.9 Hz, 1H; **v** or **i**), 6.93 (d, *J* = 7.4 Hz, 1H; **o**), 6.85 (d, *J* = 9.2 Hz, 1H; **h** or **w**), 6.81 (d, *J* = 9.2 Hz, 1H; **w** or **h**), 2.53 (s, 6H; **e**), 1.81 (d, *J* = 3.7 Hz, 12H; **c**), 1.37 (s, 9H; **y**), 1.33 (s, 9H; **z**); ^{13}C NMR (150 MHz, CD_2Cl_2, 298 K) δ 152.00, 149.88, 145.03, 144.80, 143.82, 143.18, 142.91, 141.16, 139.22, 138.48, 138.22, 137.95, 136.97, 135.24, 135.03, 134.52, 134.42, 134.08, 134.06, 133.88, 133.34, 133.12, 132.64, 132.63, 131.51, 131.39, 129.80, 129.31, 128.96, 128.92, 128.74, 128.73, 128.19, 127.99, 127.82, 127.63, 126.86, 126.65, 126.05, 125.14, 124.72, 123.20, 118.71, 115.81, 102.71, 35.10, 34.82, 31.58, 31.34, 21.48, 21.46; MALDI-TOF MS (positive) *m/z*: $[M]^+$ calcd for $C_{80}H_{66}BrClN_4Ni$ 1254.35 found 1254.41; UV-vis (chloroform, 298 K): λ (ε) = 326 nm (6.76×10^4 M^{-1} cm^{-1}), 421 nm (2.23×10^5 M^{-1} cm^{-1}), and 533 nm (1.90×10^4 M^{-1} cm^{-1}).

Synthesis of porphyrin **1a**

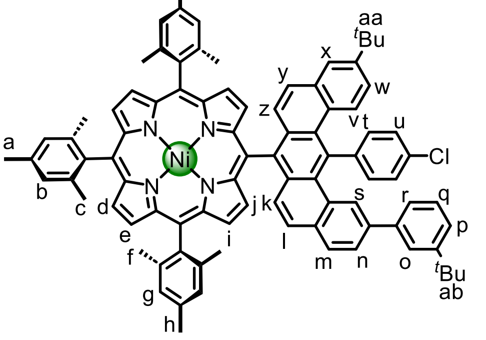

To a Schlenk flask charged with *meso*-monobromoporphyrin **24a** (35 mg, 28 μmol), mesitylboronic acid (91 mg, 0.56 mmol), $Pd(PPh_3)_4$ (3.2 mg, 2.8 μmol), and Cs_2CO_3 (0.27 g, 0.84 mmol) was added anhydrous toluene (3 mL) and DMF (0.5 mL) under argon. The mixture was degassed by three times freeze-pump-thaw cycles. The reaction mixture was heated at 80 °C for 13 h and protected from light. After completion of the reaction, the mixture was diluted with ethyl acetate (30 mL), washed with water (20 mL) for three times, dried over Na_2SO_4 and evaporated. The residue was purified by column chromatography (eluent:

dichloromethane/petroleum ether = 1/9 to 1/6, *v*/*v*), and recrystallized with dichloromethane/MeOH to give compound **1a** (28 mg, 76 % yield) as red solid. ^{1}H NMR (600 MHz, CD_2Cl_2, 298 K) δ 8.58 (d, J = 4.8 Hz, 2H; **d**), 8.56 (d, J = 4.8 Hz, 2H; **e**), 8.44 (d, J = 4.8 Hz, 2H; **i**), 8.32 (d, J = 4.9 Hz, 2H; **j**), 7.92 (s, 1H; **s**), 7.79 – 7.77 (m, 2H; **t**), 7.76 – 7.74 (m, 2; **u**), 7.72 (s, 1H; **m**), 7.68 (dd, J = 8.0, 1.6 Hz, 1H; **n**), 7.62 (d, J = 2.2 Hz, 1H; **x**), 7.47 – 7.43 (m, 2H; **o/q**), 7.42 – 7.38 (m, 1H; **p**), 7.34 (d, J = 9.2 Hz, 1H; **v**), 7.25 (s, 2H; **b**), 7.24 – 7.21 (m, 2H; **l/y**), 7.21 – 7.20 (m, 1H; **w**), 7.19 (s, 4H; **g**), 6.96 – 6.92 (m, 1H; **r**), 6.90 (d, J = 9.2 Hz, 1H; **k** or **z**), 6.85 (d, J = 9.2 Hz, 1H; **z** or **k**), 2.58 (s, 3H; **a**), 2.52 (s, 6H; **h**), 1.88 (m, 3H; **c**), 1.87 (m, 3H; **c**), 1.834 (s, 6H; **f**), 1.825 (s, 6H; **f**), 1.37 (s, 9H; **ab**), 1.34 (s, 9H; **aa**); ^{13}C NMR (150 MHz, CD_2Cl_2, 298 K) δ 151.99, 149.83, 144.89, 144.52, 143.30, 143.04, 142.89, 141.20, 139.34, 139.29, 138.30, 138.23, 138.17, 137.81, 137.56, 137.47, 135.83, 135.00, 134.61, 134.52, 134.13, 134.12, 133.18, 132.66, 131.97, 131.91, 131.78, 131.50, 131.44, 129.83, 129.35, 128.95, 128.93, 128.80, 128.60, 128.17, 128.12, 127.97, 127.80, 127.50, 127.02, 126.80, 126.01, 125.15, 124.73, 124.71, 124.70, 123.15, 117.94, 117.83, 114.93, 35.11, 34.83, 31.60, 31.36, 21.53, 21.51, 21.48; MALDI-TOF MS (positive) *m/z*: $[M]^+$ calcd for $C_{89}H_{77}ClN_4Ni$ 1294.52, found 1295.52; UV-vis (chloroform, 298 K): λ (ε) = 325 nm (5.85 × 10^4 M^{-1} cm^{-1}), 418 nm (2.15 × 10^5 M^{-1} cm^{-1}), and 527 nm (1.85 × 10^4 M^{-1} cm^{-1}).

Synthesis of porphyrin **23b** and **2b**

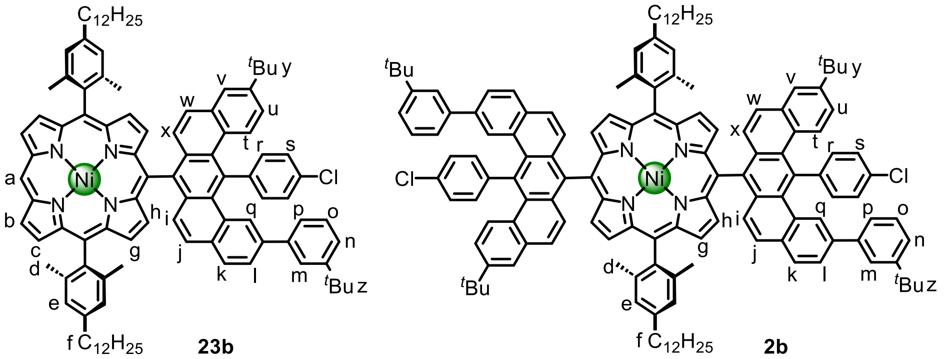

23b **2b**

To a 50-mL Schlenk tube was added 5,15-dibromo-10,20-bis(2,6-dimethyl-4-dodecylphenyl)porphyrin (Ni) (**22b**) (34 mg, 32 µmol), benzo[*m*]tetraphene pinacol borate ester **12** (91 mg, 0.13 mmol), $Pd(PPh_3)_4$ (9.9 mg, 8.6 µmol), and K_2CO_3 (42 mg, 0.30 mmol). A mixture of toluene/DMF (6 mL/6 mL) was added after three cycles of evacuation and backfilling with argon. The mixture was heated at 110 °C for 12 h, then another portion of $Pd(PPh_3)_4$ (8 mg, 7 µmol) was added. The resulting mixture was heated at 110 °C for another 24 h. After cooling to room temperature, the red solution was diluted with ethyl acetate (50 mL), washed with brine (30 mL), dried over Na_2SO_4 and evaporated. The residue was transferred into a 25-mL Schlenk tube. To the tube was added $Pd(PPh_3)_4$ (8 mg, 7 µmol), triethylamine (30 µL, 0.22 mmol), formic acid (30 µL, 0.80 mmol) and toluene (10 mL). The mixture was heated at 100 °C for 2 h under argon atmosphere, and then cooled to room temperature. After evaporation of the solvent, the residue was purified by silica gel column chromatography (petroleum ether/dichloromethane = 10/1, *v*/*v*), followed by SEC (Biobeads SX-1, chloroform) to give compounds **23b** (21 mg, 41% yield) and **2b** (18 mg, 25% yield) both as red solid. **23b**: ^{1}H NMR (600 MHz, CD_2Cl_2, 298 K) δ 9.90 (s, 1H; **a**), 9.18 (d, J = 4.7 Hz, 2H; **b**), 8.73 (d, J = 4.7 Hz, 2H; **c**), 8.50 (d, J = 4.9 Hz, 2H; **g**), 8.37 (d, J = 4.9 Hz, 2H; **h**), 7.92 (s, 1H; **q**), 7.78 (d, J = 8.3 Hz, 2H; **r**), 7.74 (d, J = 8.3 Hz, 2H;

s), 7.72 (d, J = 8.0 Hz, 1H; **k**), 7.68 (dd, J = 8.0, 1.5 Hz, 1H; **l**), 7.61 (d, J = 2.1 Hz, 1H; **v**), 7.47 – 7.43 (m, 2H; **o**/**m**), 7.41 – 7.38 (m, 1H; **n**), 7.33 (d, J = 9.3 Hz, 1H; **t**), 7.21 (s, 4H; **e**), 7.21 – 7.18 (m, 2H; **j**/**u**), 7.17 (d, J = 9.3 Hz, 1H; **w**), 6.94 (d, J = 7.6 Hz, 1H; **p**), 6.85 (d, J = 9.3 Hz, 1H, **i**), 6.81 (d, J = 9.3 Hz, 1H; **x**), 2.80 (t, J = 7.7 Hz, 4H, **f**), 1.82 (d, J = 2.0 Hz, 16H; **d**/**f**), 1.52 – 1.47 (m, 4H; **f**), 1.46 – 1.41 (m, 4H; **f**), 1.39 – 1.35 (m, 13H, **f**/**z**), 1.33 (s, 9H, **y**), 1.33 – 1.24 (m, 24H; **f**), 0.87 (t, J = 7.0 Hz, 6H, **f**); ^{13}C NMR (150 MHz, CD_2Cl_2, 298 K) δ 151.99, 149.82, 144.89, 144.31, 143.49, 143.44, 143.35, 143.08, 141.20, 139.19, 138.18, 137.84, 137.57, 135.87, 135.00, 134.67, 134.56, 134.13, 134.09, 133.16, 133.00, 132.77, 132.01, 131.86, 131.50, 131.43, 129.82, 129.34, 128.94, 128.92, 128.78, 128.59, 127.95, 127.78, 127.49, 127.02, 126.80, 126.01, 125.15, 124.73, 124.71, 124.69, 123.15, 117.84, 115.46, 105.15, 36.32, 35.11, 34.82, 32.35, 32.06, 31.59, 31.35, 30.15, 30.10, 30.05, 29.99, 29.79, 23.11, 21.58, 14.30; MALDI-TOF MS (positive) *m/z*: $[M]^+$ calcd for $C_{102}H_{111}ClN_4Ni$ 1484.78, found 1484.89; UV-vis (chloroform, 298 K): λ (ε) = 325 nm (7.41×10^4 M^{-1} cm^{-1}), 412 nm (2.48×10^5 M^{-1} cm^{-1}), 523 nm (2.13×10^4 M^{-1} cm^{-1}), and 554 nm (7.12×10^3 M^{-1} cm^{-1}). **2b**: ^{1}H NMR (600 MHz, CD_2Cl_2, 298 K) δ 8.48 (d, J = 4.9 Hz, 4H; **g**), 8.37 (d, J = 4.9 Hz, 4H; **h**), 7.94 (s, 2H; **q**), 7.80 (d, J = 8.2 Hz, 4H; **r**), 7.78 – 7.75 (m, 6H; **s**/**k**), 7.72 – 7.69 (m, 2H; **l**), 7.66 (t, J = 2.1 Hz, 2H; **v**), 7.48 – 7.44 (m, 4H; **o**/**m**), 7.41 (d, J = 8.0 Hz, 2H; **n**), 7.36 (d, J = 9.2 Hz, 2H; **t**), 7.29 (dd, J = 9.2, 3.4 Hz, 2H; **i** or **w**), 7.26 (dd, J = 9.2, 3.4 Hz, 2H; **w** or **i**), 7.22 (dd, J = 9.2, 1.7 Hz, 2H; **u**), 7.15 (s, 4H; **e**), 6.99 – 6.94 (m, 4H; **p**), 6.92 (t, J = 9.7 Hz, 2H; **i**/**x**), 2.73 (t, J = 7.4 Hz, 4H; **f**), 1.88 – 1.83 (m, 12H; **d**), 1.77 (q, J = 7.6 Hz, 4H; **f**), 1.46 – 1.41 (m, 4H; **f**), 1.38 (s, 18H; **z**), 1.36 (d, J = 1.0 Hz, 18H; **y**), 1.32 – 1.19 (m, 32H), 0.84 (t, J = 7.0 Hz, 6H; **f**); ^{13}C NMR (151 MHz, CD_2Cl_2, 298 K) δ 152.00, 149.87, 144.89, 144.58, 143.45, 143.24, 141.20, 139.11, 138.22, 137.91, 137.37, 135.64, 135.03, 134.64, 134.54, 134.13, 133.20, 132.99, 132.23, 131.52, 131.46, 129.85, 129.36, 128.98, 128.94, 128.82, 128.70, 128.03, 127.85, 127.60, 127.46, 127.01, 126.79, 126.05, 125.16, 124.74, 124.71, 123.20, 118.43, 115.57, 36.25, 35.12, 34.84, 32.30, 31.96, 31.60, 31.37, 30.09, 30.08, 30.04, 29.99, 29.94, 29.73, 23.07, 21.62, 14.27; MALDI-TOF MS (positive) *m/z*: $[M]^+$ calcd for $C_{144}H_{146}Cl_2N_4Ni$ 2059.03, found 2059.17; UV-vis (chloroform, 298 K): λ (ε) = 324 nm (1.41×10^5 M^{-1} cm^{-1}), 426 nm (2.64×10^5 M^{-1} cm^{-1}), 531 nm (2.81×10^4 M^{-1} cm^{-1}), and 559 nm (5.49×10^3 M^{-1} cm^{-1}).

Synthesis of *meso*-bromoporphyrin **24b**

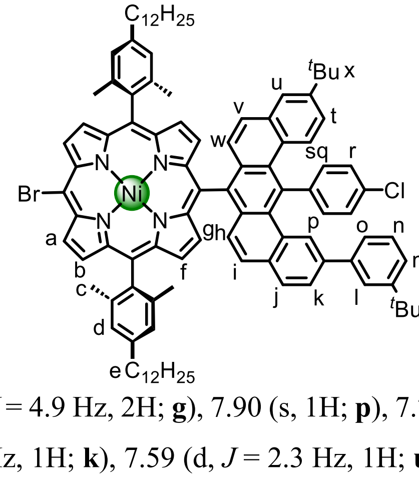

To a solution of porphyrin **23b** (60 mg, 40 μmol) dissolved in chloroform (20 mL) and pyridine (0.4 mL) was added *N*-bromosuccinimide (7.9 mg, 44 μmol) in one portion. After stirring at room temperature for 1 h, acetone (1.5 mL) was added. The solvent was evaporated under reduced pressure and the obtained residue was purified by silica gel column chromatography (petroleum ether/dichloromethane = 4/1, *v*/*v*) to give compound **24b** (59 mg, 93% yield) as red solid. ^{1}H NMR (600 MHz, $CDCl_3$) δ 9.52 (d, J = 4.9 Hz, 2H; **a**), 8.65 (d, J = 4.9 Hz, 2H; **b**), 8.39 (d, J = 4.9 Hz, 2H; **f**), 8.26 (d, J = 4.9 Hz, 2H; **g**), 7.90 (s, 1H; **p**), 7.71 (d, J = 7.4 Hz, 2H; **q**), 7.73 – 7.69 (m, 3H; **r**/**j**), 7.66 (dd, J = 8.0, 1.7 Hz, 1H; **k**), 7.59 (d, J = 2.3 Hz, 1H; **u**), 7.46 (t, J = 7.7 Hz, 1H; **n**), 7.42 (d, J = 1.9 Hz, 1H; **l**), 7.40 – 7.37 (m, 1H; **m**), 7.32 (d, J = 9.2 Hz, 1H; **s**), 7.23

– 7.17 (m, 3H; **i**/**v**), 7.16 (s, 4H; **d**), 6.92 (dt, *J* = 7.6, 1.4 Hz, 1H; **o**), 6.86 (dd, *J* = 9.2, 3.3 Hz, 2H; **h**/**w**), 2.78 (m, *J* = 7.6 Hz, 4H; **e**), 1.85 – 1.78 (m, 16H; **c**/**e**), 1.48 (q, *J* = 7.5 Hz, 4H; **e**), 1.45 – 1.40 (m, 4H; **e**), 1.37 (s, 9H; **y**), 1.35 (s, 9H; **z**), 1.34 – 1.22 (m, 28H; **e**), 0.88 (t, *J* = 6.8 Hz, 6H; **e**); ^{13}C NMR (150 MHz, $CDCl_3$, 298 K) δ 151.54, 149.47, 144.69, 144.45, 143.52, 143.15, 142.85, 142.62, 141.03, 138.97, 137.99, 137.51, 136.99, 135.04, 134.75, 134.26, 134.18, 133.80, 133.65, 133.23, 132.84, 132.51, 132.43, 131.25, 131.06, 129.55, 129.21, 128.71, 128.69, 128.49, 128.46, 127.63, 127.50, 127.40, 127.21, 126.81, 126.70, 125.84, 125.04, 124.54, 124.40, 124.35, 122.90, 118.47, 115.33, 102.64, 36.10, 34.94, 34.68, 32.09, 31.74, 31.59, 31.38, 29.90, 29.88, 29.84, 29.79, 29.78, 29.53, 22.85, 21.63, 14.28; MALDI-TOF MS (positive) *m/z*: $[M]^+$ calcd for $C_{102}H_{110}BrClN_4Ni$ 1562.70, found 1562.75; UV-vis (chloroform, 298 K): λ (ε) = 326 nm (8.07×10^4 M^{-1} cm^{-1}), 421 nm (2.70×10^5 M^{-1} cm^{-1}), and 533 nm (2.34×10^4 M^{-1} cm^{-1}).

Synthesis of tri(2,6-dimethyl-4-dodecylphenyl)porphyrin **1b**

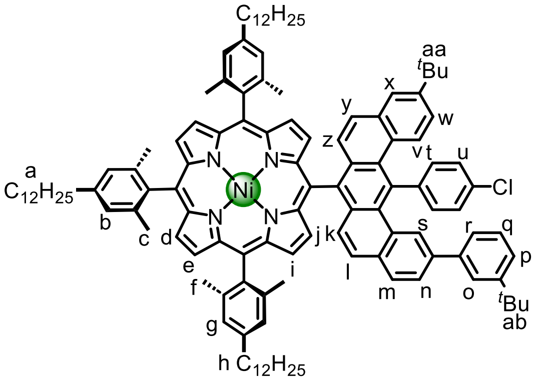

To a 25-mL Schlenk tube was added *meso*-bromoporphyrin **24b** (27 mg, 17 μmol), 2,6-dimethyl-4-dodecylphenyl boronic acid pinacol ester **22b** (138 mg, 345 μmol), $Pd(PPh_3)_4$ (2.0 mg, 2.0 μmol), and Cs_2CO_3 (169 mg, 517 μmol). A mixture of toluene/DMF (4 mL/1 mL) was added after three cycles of evacuation and backfilling with Ar. The resulting mixture was degassed by three times freeze-pump-thaw and heated at 110 °C for 13 h. After cooling to room temperature, the red solution was diluted with ethyl acetate (50 mL), washed with brine (30 mL), dried over Na_2SO_4 and evaporated. The residue was purified by silica gel column chromatography (petroleum ether/dichloromethane = 10/1, *v*/*v*) to give compound **1b** (20 mg, 66% yield) as red solid. ^{1}H NMR (600 MHz, $CDCl_3$) δ 8.59 (d, *J* = 4.8 Hz, 2H; **d**), 8.57 (d, *J* = 4.8 Hz, 2H; **e**), 8.44 (d, *J* = 4.8 Hz, 2H; **i**), 8.30 (d, *J* = 4.9 Hz, 2H; **j**), 7.94 – 7.91 (m, 1H; **s**), 7.81 – 7.77 (m, 2H; **t**), 7.75 – 7.71 (m, 3H; **u**/**m**), 7.67 (dd, *J* = 7.9, 1.7 Hz, 1H; **n**), 7.60 (d, *J* = 2.3 Hz, 1H; **x**), 7.47 (t, *J* = 7.7 Hz, 1H; **q**), 7.43 (d, *J* = 1.9 Hz, 1H; **o**), 7.41 – 7.38 (m, 1H; **p**), 7.34 (d, *J* = 9.3 Hz, 1H; **v**), 7.23 (s, 2H; **b**), 7.22 – 7.17 (m, 4H; **w**/**l**/**y**), 7.16 (s, 4H; **g**), 6.95 (d, *J* = 7.6 Hz, 1H; **r**), 6.90 (d, *J* = 9.3 Hz, 2H; **k**/**z**), 2.87 – 2.82 (m, 2H; **a**), 2.81 – 2.75 (m, 4H; **h**), 1.90 (s, 6H; **c**), 1.85 (d, *J* = 4.8 Hz, 12H; **f**), 1.84 – 1.79 (m, 4H; **h**), 1.54 (d, *J* = 3.5 Hz, 2H; **a**), 1.51 – 1.45 (m, 6H; **a**/**h**), 1.45 – 1.40 (m, 6H; **a**/**h**), 1.38 (s, 9H; **ab**), 1.36 (s, 9H; **aa**), 1.34 – 1.24 (m, 42H; **a**/**h**), 0.91 (s, 3H; **a**), 0.88 (t, *J* = 7.0 Hz, 6H; **h**); ^{13}C NMR (150 MHz, $CDCl_3$, 298 K) δ 151.52, 149.37, 144.57, 144.42, 143.19, 142.94, 142.92, 142.87, 142.77, 141.09, 139.13, 139.06, 139.05, 138.02, 137.91, 137.73, 137.62, 137.36, 135.82, 134.70, 134.40, 134.33, 133.85, 132.91, 132.44, 131.73, 131.57, 131.42, 131.23, 131.13, 129.57, 129.25, 129.19, 129.12, 128.78, 128.69, 128.53, 128.38, 128.34, 127.59, 127.46, 127.26, 127.17, 127.11, 127.02, 126.91, 125.78, 125.45, 125.05, 124.55, 124.38, 124.32, 122.83, 117.87, 117.72, 114.71, 36.15, 36.09, 34.94, 34.68, 32.11, 32.07, 31.79, 31.72, 31.60, 31.39, 29.92, 29.89, 29.87, 29.83, 29.80, 29.78, 29.75, 29.56, 29.52, 22.87, 22.84, 21.74, 21.71, 14.30, 14.27; MALDI-TOF MS (positive) *m/z*: $[M]^+$ calcd for $C_{122}H_{143}ClN_4Ni$ 1757.03, found

1757.09; UV-vis (chloroform, 298 K): λ (ε) = 324 nm (6.05 × 10^4 M^{-1} cm^{-1}), 419 nm (2.26 × 10^5 M^{-1} cm^{-1}), 529 nm (2.01 × 10^4 M^{-1} cm^{-1}), and 559 nm (4.21 × 10^3 M^{-1} cm^{-1}).

Synthesis of fused porphyrin oligomers

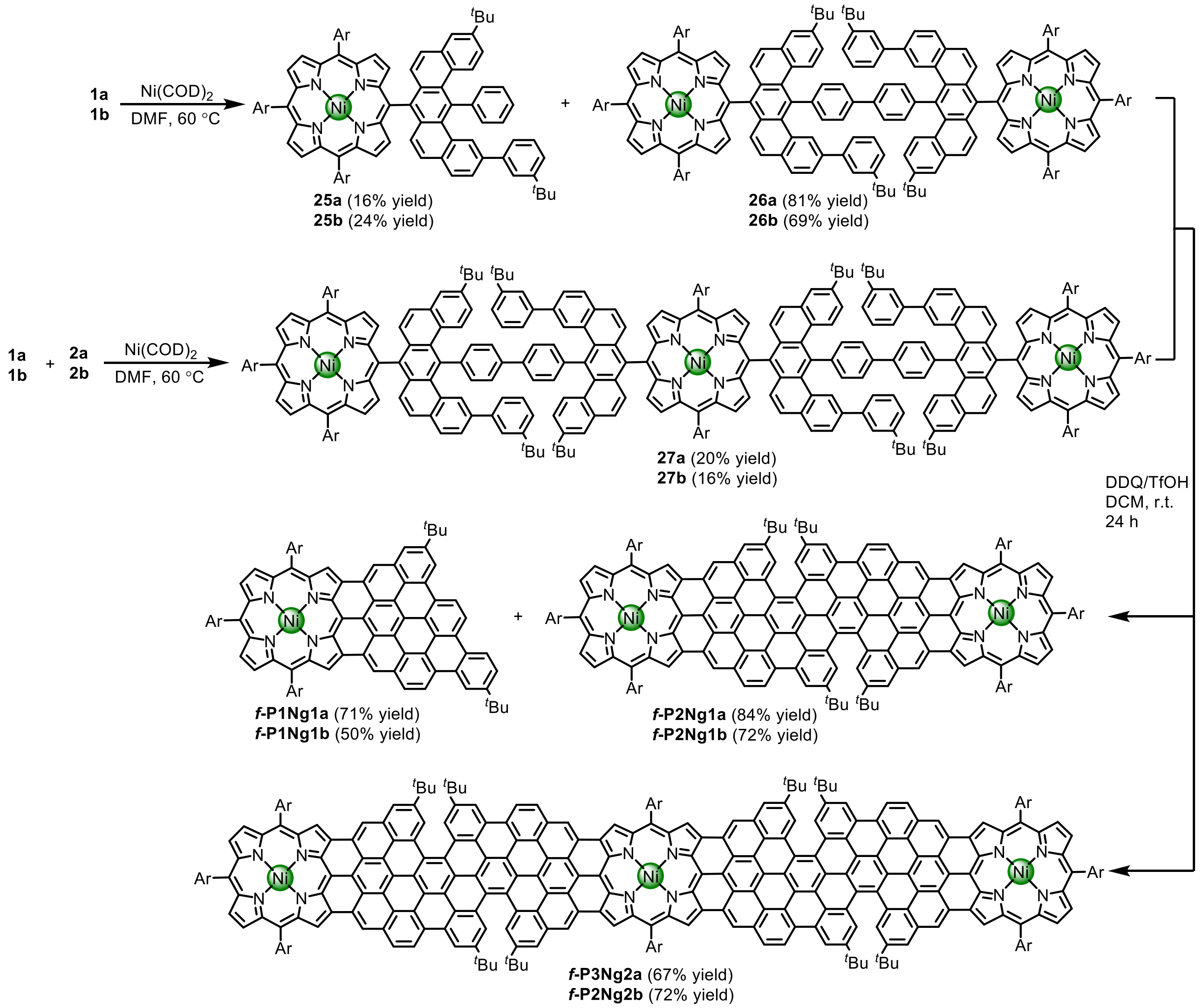

Supplementary Figure 6. Synthetic scheme of fused porphyrin oligomers as models.

Synthesis of porphyrin monomer **25a** and dimer **26a**

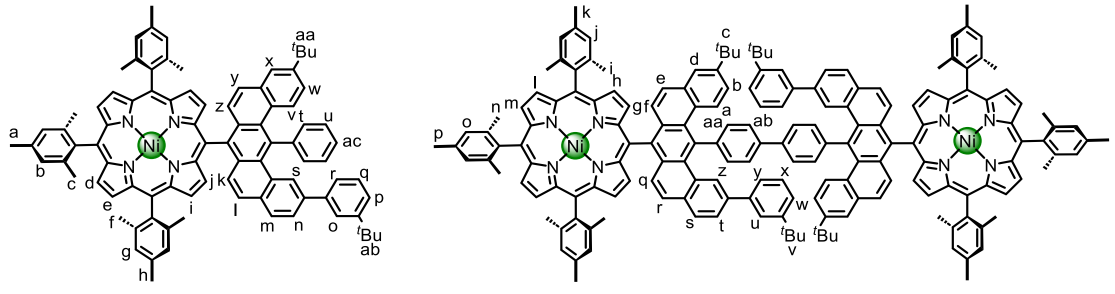

25a **26a**

Trimesitylporphyrin **1a** (20 mg, 15 μmol), 2,2'-bipyridine (12 mg, 77 μmol), and 1,5-cyclooctadiene (8.3 mg, 77 μmol) were dissolved in DMF (0.5 mL) and toluene (0.5 mL). The mixture was degassed by three freeze-pump-thaw cycles, then Ni(COD)2 (21 mg, 77 μmol) was added under the protection of argon. The mixture

was degassed by one more time freeze-pump-thaw and then heated at 60 °C for 30 min. The temperature was increased to 110 °C and the resulting mixture was stirred for 18 h in dark. After cooling to room temperature, the solution was diluted with dichloromethane and passed through a short plug of silica (eluent: dichloromethane). The solvent was evaporated and the residue was purified by silica gel column chromatography (dichloromethane/petroleum ether = 1/4, *v*/*v*), followed by recrystallization from dichloromethane and methanol to give dechlorinated product **25a** (3.1 mg, 16% yield) and porphyrin dimer **26a** (15 mg, 81% yield) as red solid. **25a**: ^{1}H NMR (600 MHz, CD_2Cl_2, 298 K) δ 8.58 (d, J = 4.8 Hz, 2H; **d**), 8.56 (d, J = 4.8 Hz, 2H; **e**), 8.44 (d, J = 4.8 Hz, 2H; **i**), 8.33 (d, J = 4.8 Hz, 2H; **j**), 8.07 (s, 1H; **s**), 7.84 – 7.79 (m, 2H; **t**), 7.78 – 7.73 (m, 3H; **u**/**ac**), 7.72 (d, J = 8.1 Hz, 1H; **m**), 7.66 (dd, J = 8.0, 1.6 Hz, 1H; **n**), 7.59 (d, J = 2.1 Hz, 1H; **x**), 7.39 (s, 1H; **o**), 7.36 (d, J = 7.9 Hz, 1H; **p**), 7.31 (t, J = 7.6 Hz, 1H; **q**), 7.25 (s, 2H; **b**), 7.24 – 7.22 (m, 1H; **l**), 7.19 (s, 4H; **g**), 7.18 (d, J = 3.5 Hz, 1H; **y**), 7.16 (s, 1H; **v**), 7.09 (dd, J = 9.3, 2.3 Hz, 1H; **w**), 6.93 (d, J = 7.4 Hz, 1H; **r**), 6.89 (d, J = 9.2 Hz, 1H; **k**), 6.85 (d, J = 9.2 Hz, 1H; **z**), 2.58 (s, 3H; **a**), 2.52 (s, 6H; **h**), 1.88 (s, 3H; **c**), 1.87 (s, 3H; **c**), 1.83 (s, 6H; **f**), 1.83 (s, 6H; **f**), 1.37 (s, 9H; **ab**), 1.32 (s, 9H; **aa**); ^{13}C NMR (150 MHz, CD_2Cl_2, 298 K) δ 151.84, 149.57, 146.33, 144.58, 143.28, 143.01, 142.89, 141.24, 139.79, 139.38, 139.35, 139.30, 138.29, 138.22, 137.92, 137.58, 137.48, 135.48, 134.62, 134.47, 134.02, 133.14, 132.73, 132.49, 131.95, 131.87, 131.79, 131.75, 131.29, 129.85, 129.34, 129.12, 128.83, 128.79, 128.52, 128.44, 128.17, 128.11, 127.75, 127.43, 127.04, 126.83, 125.93, 125.35, 124.72, 124.53, 123.01, 117.89, 117.80, 115.16, 114.18, 32.35, 31.62, 31.33, 23.11, 21.53, 21.51, 21.48; MALDI-TOF MS (positive) *m/z*: $[M]^+$ calcd for $C_{89}H_{78}N_4Ni$ 1260.56, found 1260.69; UV-vis (chloroform, 298 K): λ (ε) = 316 nm (3.36×10^4 M^{-1} cm^{-1}), 420 nm (1.17×10^5 M^{-1} cm^{-1}), 542 nm (7.57×10^3 M^{-1} cm^{-1}), and 575 nm (2.40×10^3 M^{-1} cm^{-1}). **26a**: ^{1}H NMR (400 MHz, CD_2Cl_2, 298 K) δ 8.60 (d, J = 4.9 Hz, 4H; **m**), 8.58 (d, J = 4.9 Hz, 4H; **l**), 8.49 (d, J = 4.9 Hz, 4H; **h**), 8.41 (d, J = 4.9 Hz, 4H; **g**), 8.24 (d, J = 8.3 Hz, 6H; **z**/**ab**), 8.07 (d, J = 8.1 Hz, 4H; **aa**), 7.80 (d, J = 8.1 Hz, 2H; **s**), 7.74 – 7.73 (m, 2H; **t**), 7.72 – 7.68 (m, 4H; **a**/**d**), 7.52 (s, 2H; **u**), 7.34 – 7.29 (m, 6H; **b**/**e**/**r**), 7.27 (s, 4H; **o**), 7.25 – 7.23 (m, 4H; **x**/**w**), 7.21 (s, 8H; **j**), 7.02 – 6.99 (m, 2H; **y**), 6.98 – 6.92 (m, 4H; **f**/**q**), 2.59 (s, 6H; **p**), 2.54 (s, 12H; **k**), 1.90 (s, 6H; **n**), 1.89 (s, 6H; **n**), 1.87 (s, 12H; **i**), 1.86 (s, 12H; **i**), 1.34 (s, 18H; **c**), 1.29 (s, 18H; **v**); ^{13}C NMR (150 MHz, CD_2Cl_2, 298 K) δ 151.86, 149.82, 145.89, 144.63, 143.48, 143.37, 143.35, 143.32, 143.14, 143.09, 143.05, 142.93, 141.70, 141.53, 139.36, 139.33, 138.97, 138.35, 138.31, 138.25, 137.59, 137.51, 137.47, 135.71, 134.72, 134.61, 134.21, 134.13, 133.24, 133.22, 133.03, 132.78, 132.02, 131.97, 131.92, 131.84, 131.80, 131.51, 130.47, 130.05, 129.83, 129.79, 129.24, 129.01, 128.93, 128.64, 128.19, 128.15, 128.12, 127.55, 127.08, 127.02, 126.07, 125.41, 125.23, 125.15, 124.87, 124.73, 124.71, 124.62, 123.22, 117.94, 117.85, 117.77, 117.75, 115.15, 35.07, 34.87, 31.58, 31.47, 21.55, 21.50; MALDI-TOF MS (positive) *m/z*: $[M]^+$ calcd for $C_{178}H_{154}N_8Ni_2$ 2519.10, found 2519.20; UV-vis (chloroform, 298 K): λ (ε) = 322 nm (7.78×10^4 M^{-1} cm^{-1}), 419 nm (2.78×10^5 M^{-1} cm^{-1}), 528 nm (2.73×10^4 M^{-1} cm^{-1}), and 558 nm (6.53×10^3 M^{-1} cm^{-1}).

Synthesis of porphyrin trimer **27a**

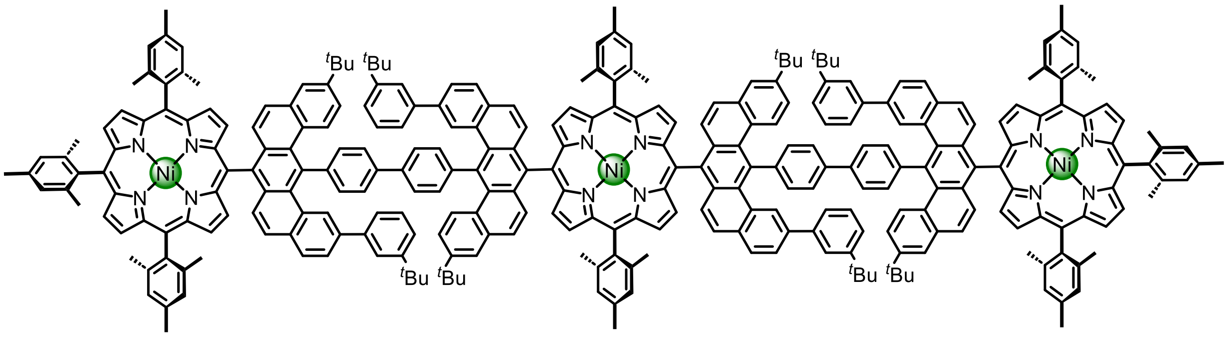

Trimesitylporphyrin **1a** (5.0 mg, 3.9 μmol), dichloroporphyrin **2a** (3.4 mg, 2.4 μmol), 2,2'-bipyridine (12 mg, 77 μmol), and 1,5-cyclooctadiene (8.3 mg, 77 μmol) were dissolved in DMF (0.5 mL) and benzene (0.3 mL). The solution was degassed by three times freeze-pump-thaw cycles, then Ni(COD)$_2$ (21 mg, 77 μmol) was added in one portion under argon atmosphere. The mixture was degassed by one more time freeze-pump-thaw and then heated at 60 °C for 30 min. The temperature was increased to 80 °C and the reaction mixture was stirred for 23 h in dark. After cooling to room temperature, the solution was diluted with dichloromethane and passed through a short plug of silica (eluent: dichloromethane). The solvent was evaporated and the residue was purified by silica gel column chromatography (THF/petroleum ether = 1/20, *v*/*v*) and recycling GPC (toluene/pyridine = 100/1, *v*/*v*) to give **27a** (1.55 mg, 20% yield) as red solid. ^{1}H NMR (600 MHz, CD_2Cl_2, 298 K) δ 8.61 (d, J = 4.8 Hz, 4H; β-H), 8.59 (d, J = 4.7 Hz, 4H; β-H), 8.55 (d, J = 4.7 Hz, 4H; β-H), 8.49 (t, J = 4.9 Hz, 8H; β-H), 8.42 (d, J = 4.7 Hz, 4H; β-H), 8.29 (s, 2H; Ar-H), 8.28 – 8.23 (m, 10H; Ar-H), 8.11 – 8.07 (m, 8H; Ar-H), 7.85 (d, J = 7.4 Hz, 2H; Ar-H), 7.81 (d, J = 7.9 Hz, 2H; Ar-H), 7.77 – 7.71 (m, 12H; Ar-H), 7.54 (d, J = 6.0 Hz, 4H; Ar-H), 7.39 – 7.33 (m, 8H; Ar-H), 7.31 (dd, J = 13.8, 4.0 Hz, 4H; Ar-H), 7.26 (d, J = 11.7 Hz, 14H; Ar-H), 7.21 (d, J = 5.9 Hz, 12H; Mes-H), 7.06 – 7.00 (m, 8H; Ar-H), 6.96 (dd, J = 12.0, 9.4 Hz, 4H; Ar-H), 2.59 (s, 6H; Me-H), 2.54 (s, 12H; Me-H), 2.52 (s, 6H; Me-H), 1.93 – 1.89 (m, 24H; Me-H), 1.87 (d, J = 4.2 Hz, 24H; Me-H), 1.37 (s, 18H; ^{t}Bu-H), 1.36 (s, 18H; ^{t}Bu-H),); ^{13}C NMR (150 MHz, CD_2Cl_2, 298 K) δ 151.88, 149.89, 149.83, 145.91, 145.89, 144.73, 144.63, 143.32, 143.05, 142.93, 141.74, 141.70, 141.54, 139.37, 139.33, 139.30, 139.08, 138.97, 138.41, 138.35, 138.31, 138.25, 137.59, 137.51, 137.32, 135.72, 135.56, 134.75, 134.72, 134.65, 134.62, 134.24, 133.24, 133.14, 132.78, 132.24, 132.02, 131.92, 131.88, 131.85, 131.79, 130.49, 130.08, 130.06, 129.82, 129.27, 129.24, 129.03, 128.98, 128.94, 128.76, 128.64, 128.35, 128.29, 128.19, 128.15, 127.67, 127.55, 127.08, 127.03, 126.13, 126.07, 125.42, 125.25, 124.91, 124.87, 124.65, 123.28, 123.23, 118.39, 117.94, 117.85, 115.80, 115.14, 114.18, 35.09, 34.90, 34.88, 31.59, 31.49, 31.48; MALDI-TOF MS (positive) *m/z*: $[M]^+$ calcd for $C_{300}H_{256}N_{12}Ni_3$ 4199.85, found 4199.83; UV-vis (chloroform, 298 K): λ (ε) = 323 nm (2.10 × 10^5 M^{-1} cm^{-1}), 420 nm (5.61 × 10^5 M^{-1} cm^{-1}), 529 nm (6.41 × 10^4 M^{-1} cm^{-1}), and 560 nm (1.54 × 10^4 M^{-1} cm^{-1}).

Synthesis of porphyrin monomer **25b** and dimer **26b**

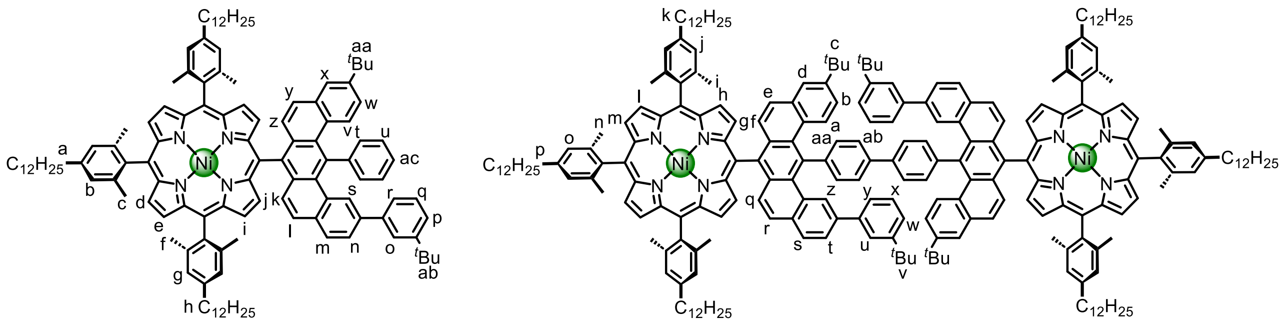

25b **26b**

Tri(2,6-dimethyl-4-dodecylphenyl)porphyrin **1b** (5.00 mg, 2.84 μmol), 2,2'-bipyridine (4.44 mg, 77 μmol), and 1,5-cyclooctadiene (3.6 μL, 38 μmol) were dissolved in DMF (1 mL). The mixture was degassed by three freeze-pump-thaw cycles, then $Ni(COD)_2$ (7.70 mg, 28.4 μmol) was added under the argon atmosphere. The mixture was degassed by one more time freeze-pump-thaw cycle and then heated at 60 °C for 30 min. The temperature was increased to 110 °C and the resulting solution was stirred for 13 h in dark. After cooling to room temperature, the solution was diluted with dichloromethane and passed through a short plug of silica (eluent: dichloromethane). The solvent was evaporated and the residue was purified by silica gel column chromatography (dichloromethane/petroleum ether = 1/6, *v*/*v*), followed by recrystallization from dichloromethane and methanol to give dechlorinated product **25b** (1.2 mg, 24% yield) and porphyrin dimer **26b** (3.4 mg, 69% yield) as red solid. **25b**: ^{1}H NMR (600 MHz, $CDCl_3$, 298 K) δ 8.58 (d, J = 4.8 Hz, 2H; **d**), 8.56 (d, J = 4.8 Hz, 2H; **e**), 8.42 (d, J = 4.8 Hz, 2H; **i**), 8.31 (d, J = 4.8 Hz, 2H; **j**), 8.06 (s, 1H; **s**), 7.83 (d, J = 6.3 Hz, 2H; **t**), 7.74 – 7.67 (m, 4H; **u**/**ac**/**m**), 7.64 (dd, J = 8.0, 1.5 Hz, 1H; **n**), 7.57 (d, J = 2.1 Hz, 1H; **x**), 7.37 (s, 1H; **o**), 7.35 (d, J = 7.9 Hz, 1H; **p**), 7.31 (t, J = 7.6 Hz, 1H; **q**), 7.22 (s, 2H; **b**), 7.21 – 7.16 (m, 3H; **l**/**y**/**v**), 7.15 (s, 4H; **g**), 7.08 (dd, J = 9.3, 2.2 Hz, 1H; **w**), 6.94 (d, J = 7.4 Hz, 1H; **r**), 6.88 (dd, J = 9.2, 3.8 Hz, 2H; **k**/**z**), 2.86 – 2.80 (m, 2H; **a**), 2.80 – 2.74 (m, 4H; **h**), 1.88 (d, J = 2.3 Hz, 6H; **c**), 1.82 (dd, J = 16.2, 7.1 Hz, 18H; **f**/**a**/**h**), 1.49 – 1.44 (m, 6H; **a**/**h**), 1.41 (d, J = 6.9 Hz, 6H; **a**/**h**), 1.37 (s, 9H; **ab**), 1.33 (s, 9H; **aa**), 1.30 – 1.24 (m, 48H; **a**/**h**), 0.88 – 0.87 (m, 9H; **a**/**h**); ^{13}C NMR (150 MHz, $CDCl_3$, 298 K) δ 151.33, 149.07, 146.05, 144.51, 143.17, 142.92, 142.89, 142.85, 142.79, 141.20, 139.14, 139.07, 138.97, 137.77, 137.65, 137.62, 135.46, 134.42, 134.30, 133.74, 132.86, 132.56, 132.31, 131.70, 131.52, 131.49, 131.38, 130.98, 129.64, 129.27, 128.87, 128.55, 128.37, 128.27, 128.11, 127.82, 127.43, 127.16, 127.10, 126.94, 125.69, 125.23, 124.58, 124.18, 124.08, 122.68, 117.80, 117.69, 115.00, 77.37, 77.16, 76.95, 53.56, 39.23, 37.26, 36.81, 36.15, 36.09, 34.97, 34.93, 34.60, 34.28, 32.91, 32.42, 32.11, 32.09, 32.07, 31.79, 31.71, 31.62, 31.59, 31.39, 31.36, 30.20, 29.92, 29.89, 29.87, 29.83, 29.80, 29.78, 29.75, 29.66, 29.56, 29.51, 28.13, 27.58, 27.25, 26.91, 23.21, 22.87, 22.85, 22.84, 22.82, 22.77, 22.49, 21.73, 21.70, 20.30, 19.89, 19.39, 14.57, 14.29, 14.27, 14.21, 11.56, 1.20; MALDI-TOF MS (positive) *m/z*: $[M]^+$ calcd for $C_{122}H_{144}N_4Ni$ 1723.07, found 1723.17; UV-vis (chloroform, 298 K): λ (ε) = 324 nm (5.79 × 10^4 M^{-1} cm^{-1}), 418 nm (2.11 × 10^5 M^{-1} cm^{-1}), 528 nm (1.92 × 10^4 M^{-1} cm^{-1}), and 558 nm (4.26 × 10^3 M^{-1} cm^{-1}). **26b**: ^{1}H NMR (600 MHz, CD_2Cl_2, 298 K) δ 8.60 (d, J = 4.8 Hz, 4H; **m**), 8.58 (d, J = 4.8 Hz, 4H; **l**), 8.49 (d, J = 4.8 Hz, 4H; **h**), 8.40 (d, J = 4.8 Hz, 4H; **g**), 8.26 (s, 2H; **z**), 8.24 (d, J = 7.8 Hz, 4H; **ab**), 8.06 (d, J = 7.8 Hz, 4H; **aa**), 7.80 (d, J = 8.0 Hz, 2H; **s**), 7.75 – 7.68 (m, 6H; **t**/**d**/**a**), 7.52 (s, 2H; **u**), 7.34 – 7.28 (m, 6H; **b**/**e**/**r**), 7.27 (s, 4H; **o**), 7.25 – 7.23 (m, 4H; **x**/**w**), 7.21 (s, 8H; **j**),

7.00 (d, J = 6.6 Hz, 2H; **y**), 6.95 (dd, J = 10.9, 9.4 Hz, 4H; **f/q**), 2.87 – 2.82 (m, 4H; **p**), 2.81 – 2.77 (m, 8H; **k**), 1.90 (d, J = 4.7 Hz, 12H; **n**), 1.87 (d, J = 3.2 Hz, 24H; **i**), 1.83 (dd, J = 15.4, 7.9 Hz, 12H; **p**/**k**), 1.48 (dd, J = 13.6, 6.5 Hz, 12H; **p**/**k**), 1.44 – 1.41 (m, 12H; **p**/**k**), 1.34 (s, 18H; **c**), 1.34 – 1.30 (m, 42H; **p**/**k**), 1.29 (s, 18H; **v**), 1.27 (s, 42H; **p**/**k**), 0.92 – 0.89 (m, 6H; **p**), 0.86 (d, J = 7.1 Hz, 12H; **k**); ^{13}C NMR (150 MHz, CD_2Cl_2, 298 K) δ 151.85, 149.80, 145.89, 144.58, 143.47, 143.41, 143.29, 143.02, 142.90, 141.69, 141.53, 139.24, 139.20, 138.95, 138.39, 138.33, 137.72, 137.63, 135.70, 134.72, 134.61, 134.21, 133.24, 133.22, 132.74, 132.05, 131.94, 131.83, 131.82, 130.46, 130.04, 129.78, 129.38, 129.23, 129.00, 128.97, 128.92, 128.61, 128.57, 128.11, 127.53, 127.47, 127.08, 127.02, 126.05, 125.65, 125.40, 125.22, 124.86, 124.61, 123.21, 118.02, 117.93, 115.09, 36.36, 36.31, 35.06, 34.86, 32.38, 32.34, 32.09, 32.03, 31.57, 31.46, 30.17, 30.13, 30.09, 30.03, 30.01, 29.98, 29.82, 29.78, 23.14, 23.10, 21.62, 14.32, 14.29; MALDI-TOF MS (positive) m/z: $[M]^+$ calcd for $C_{244}H_{286}N_8Ni_2$ 3444.13, found 3444.03; UV-vis (chloroform, 298 K): λ (ε) = 324 nm (9.59 × 10^4 M^{-1} cm^{-1}), 420 nm (3.71 × 10^5 M^{-1} cm^{-1}), 527 nm (3.54 × 10^4 M^{-1} cm^{-1}), and 558 nm (8.03 × 10^3 M^{-1} cm^{-1}).

Synthesis of porphyrin **27b**

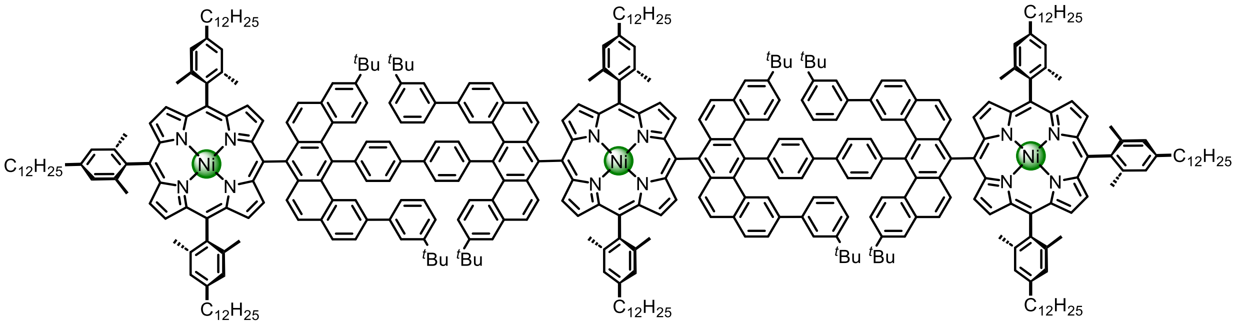

Tri(2,6-dimethyl-4-dodecylphenyl)porphyrin **1b** (5.00 mg, 2.84 μmol), dichloroporphyrin **2b** (5.86 mg, 2.84 μmol), 2,2'-bipyridine (8.0 mg, 51 μmol), and 1,5-cyclooctadiene (6.4 μL, 51 μmol) were dissolved in DMF (1.0 mL). The solution was degassed by three times freeze-pump-thaw, then $Ni(COD)_2$ (14 mg, 51 μmol) was added in one portion under argon atmosphere. The mixture was degassed by one more time freeze-pump-thaw, then heated at 60 °C for 30 min. The temperature was increased to 110 °C and the reaction was stirred for 13 h in dark. After cooling to room temperature, the solution was diluted with dichloromethane and passed through a short plug of silica (eluent: dichloromethane). The solvent was evaporated and the residue was purified by preparative size exclusion chromatography column (Bio-Beads S-X1, toluene) followed by silica gel column chromatography (dichloromethane/petroleum ether = 1/4, v/v) to give porphyrin **27b** (1.25 mg, 16% yield) as red solid. ^{1}H NMR (600 MHz, CD_2Cl_2, 298 K) δ 8.60 (d, J = 4.8 Hz, 4H; β-H), 8.58 (d, J = 4.8 Hz, 4H; β-H), 8.55 (d, J = 4.7 Hz, 4H; β-H), 8.52 – 8.46 (m, 8H; β-H), 8.41 (d, J = 4.8 Hz, 4H; β-H), 8.31 – 8.21 (m, 12H), 8.12 – 8.06 (m, 8H), 7.85 (d, J = 7.7 Hz, 3H), 7.81 (d, J = 8.0 Hz, 3H), 7.78 – 7.69 (m, 12H), 7.54 (d, J = 5.9 Hz, 5H), 7.40 – 7.23 (m, 25H), 7.22 – 7.19 (m, 10H), 7.07 – 7.00 (m, 8H), 6.99 – 6.92 (m, 6H), 2.86 – 2.83 (m, 4H), 2.82 – 2.75 (m, 12H), 1.93– 1.90 (m, 12H), 1.90 – 1.86 (m, 24H), 1.86 – 1.76 (m, 16H), 1.50 – 1.46 (m, 16H), 1.44 – 1.40 (m, 16H), 1.37 (s, 18H), 1.35 (s, 18H), 1.37 – 1.20 (m, 112H), 0.90 – 0.83 (m, 24H); ^{13}C NMR (150 MHz, CD_2Cl_2, 298 K) δ 151.44, 145.45, 144.27, 144.16, 143.03, 142.97, 142.86, 142.60, 142.48, 141.11, 138.81, 138.77, 138.63, 137.97, 137.91, 137.28, 137.20, 134.29, 134.18, 133.78,

132.80, 132.30, 131.85, 131.63, 131.51, 131.38, 130.05, 129.63, 129.37, 128.82, 128.59, 127.74, 127.68, 127.09, 127.05, 126.78, 126.58, 125.64, 124.98, 124.81, 124.47, 124.19, 122.80, 118.04, 117.60, 117.50, 114.66, 35.88, 34.65, 31.95, 31.91, 31.88, 31.66, 31.60, 31.15, 31.04, 29.74, 29.70, 29.66, 29.61, 29.58, 29.55, 29.39, 29.35, 29.31, 22.71, 22.67, 22.65, 21.25, 21.19, 13.88, 13.86; MALDI-TOF MS (positive) *m/z*: $[M]^+$ calcd for $C_{388}H_{432}N_{12}Ni_3$ 5433.22, found 5433.52; UV-vis (chloroform, 298 K): λ (ε) = 324 nm (1.70×10^5 M^{-1} cm^{-1}), 421 nm (4.68×10^5 M^{-1} cm^{-1}), 529 nm (5.26×10^4 M^{-1} cm^{-1}), and 558 nm (1.35×10^4 M^{-1} cm^{-1}).

Synthesis of ***f*-P1Ng1a**

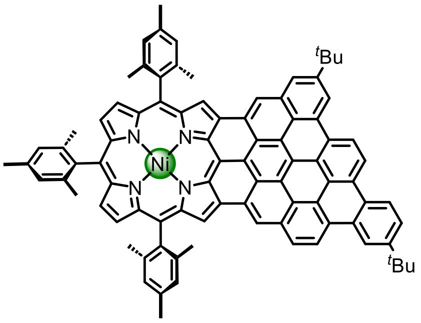

To a solution of **25a** (2.0 mg, 1.6 μmol) and DDQ (2.7 mg, 11 μmol) dissolved in dry degassed dichloromethane (6 mL) was added triflic acid (0.06 mL). The mixture was stirred at 22 °C for 6 h. Triethylamine (0.2 mL) was added via a syringe to quench the reaction. After addition of methanol (20 mL), the precipitate was collected by filtration and washed with MeOH (20 mL). The crude product was further purified by silica gel column chromatography (dichloromethane/petroleum ether = 1/10, *v*/*v*) to give compound ***f*-P1Ng1a** (1.4 mg, 71% yield) as purple solid. It is impossible to get well-resolved ^1H NMR spectra because of strong aggregation in solvents. MALDI-TOF MS (positive) *m/z*: $[M]^+$ calcd for $C_{89}H_{68}N_4Ni$ 1250.48, found 1250.61; UV-vis (chloroform, 298 K): λ (ε) = 319 nm (2.40×10^4 M^{-1} cm^{-1}), 415 nm (2.51×10^4 M^{-1} cm^{-1}), 530 nm (3.05×10^4 M^{-1} cm^{-1}), 592 nm (1.19×10^4 M^{-1} cm^{-1}), 722 nm (1.13×10^4 M^{-1} cm^{-1}), and 800 nm (2.26×10^4 M^{-1} cm^{-1}).

Synthesis of ***f*-P2Ng1a**

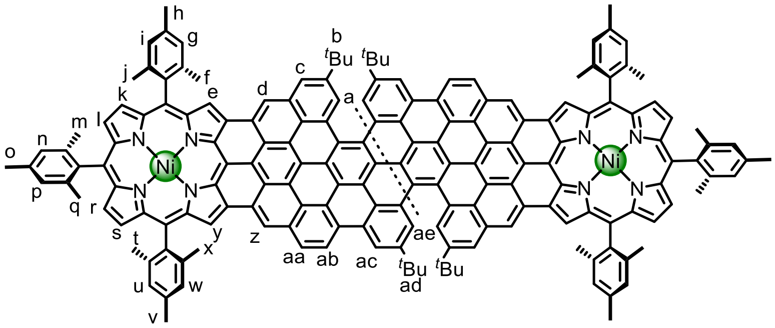

To a solution of **26a** (3.0 mg, 1.2 μmol) and DDQ (4.9 mg, 21 μmol) dissolved in dry degassed dichloromethane (10 mL) was added triflic acid (0.15 mL). The mixture was stirred at 22 °C for 24 h. Triethylamine (0.4 mL) was added via a syringe to quench the reaction. After addition of methanol (20 mL), the precipitate was collected by filtration and washed with MeOH (20 mL). The crude product was further

purified by silica gel column chromatography (dichloromethane/petroleum ether = 1/10, *v*/*v*) to give compound ***f*-P2Ng1a** (2.5 mg, 84% yield) as purple solid. ^{1}H NMR (600 MHz, $CDCl_3/CS_2$ = 1/1, *v*/*v*, 298 K) δ 10.13 (s, 2H; **a**), 10.07 (s, 2H; **z**), 9.91 (d, J = 9.4 Hz, 2H; **ab**), 9.80 (s, 2H; **ae**), 9.74 (s, 2H; **d**), 9.56 (s, 2H; **ac**), 9.45 (s, 2H; **y**), 9.37 (s, 2H; **e**), 9.26 (d, J = 8.6 Hz, 2H; **aa**), 8.73 (s, 2H; **d**), 8.47 (t, J = 4.4 Hz, 4H; **k**/**s**), 8.36 (t, J = 3.8 Hz, 4H; **l**/**r**), 7.43 (s, 2H; **g** or **i**), 7.41 (s, 2H; **i** or **g**), 7.39 (s, 2H; **u** or **w**), 7.36 (s, 2H; **w** or **u**), 7.24 (s, 2H; **n** or **p**), 7.21 (s, 2H; **p** or **n**), 2.77 (s, 6H; -Me), 2.75 (s, 6H; -Me), 2.61 (s, 6H; -Me), 2.21 – 2.16 (m, 12H; -Me), 2.05 (s, 12H; -Me), 2.02 (s, 6H; -Me), 1.90 (s, 6H; -Me), 1.51 (s, 18H; **ad**), 1.44 (s, 18H; **b**); ^{13}C NMR (150 MHz, $CDCl_3/CS_2$ = 1/1, *v*/*v*, 298 K) δ 148.38, 148.33, 144.94, 144.91, 143.70, 143.59, 142.89, 142.63, 139.49, 139.39, 139.18, 139.12, 138.99, 138.97, 137.79, 137.76, 137.71, 137.51, 136.31, 135.80, 132.33, 131.96, 131.56, 130.72, 130.64, 130.23, 130.20, 129.59, 129.57, 128.51, 128.33, 128.30, 128.23, 128.20, 127.95, 127.88, 127.54, 126.33, 126.09, 125.34, 125.14, 124.96, 124.59, 124.53, 124.47, 123.89, 123.40, 123.36, 123.14, 123.02, 122.97, 122.67, 122.38, 121.84, 121.23, 120.79, 120.68, 120.10, 119.93, 119.80, 116.73, 111.15, 31.81, 31.70, 21.78, 21.75, 21.64, 21.61, 21.59, 21.57, 21.54, 21.53; MALDI-TOF MS (positive) *m/z*: $[M]^+$ calcd for $C_{178}H_{130}N_8Ni_2$ 2494.91, found 2495.12; UV-vis (chloroform, 298 K): λ (ε) = 334 nm (5.94×10^4 M^{-1} cm^{-1}), 376 nm (7.08×10^4 M^{-1} cm^{-1}), 420 nm (6.97×10^4 M^{-1} cm^{-1}), 462 nm (4.57×10^4 M^{-1} cm^{-1}), 540 nm (1.16×10^5 M^{-1} cm^{-1}), 742 nm (4.83×10^4 M^{-1} cm^{-1}), and 832 nm (1.25×10^5 M^{-1} cm^{-1}).

Synthesis of ***f*-P3Ng2a**

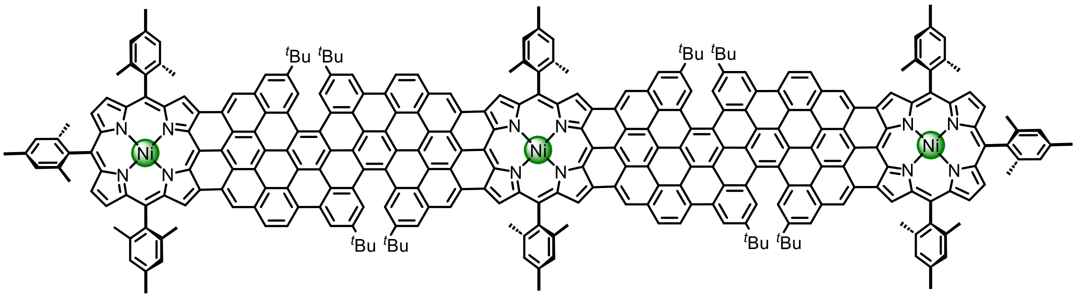

To a solution of **27a** (1.5 mg, 0.36 μmol) and DDQ (2.9 mg, 13 μmol) dissolved in dry degassed dichloromethane (5 mL) was added triflic acid (0.05 mL). The reaction mixture was stirred at 22 °C for 17 h, then triethylamine (0.4 mL) was added via a syringe to quench the reaction. After addition of methanol (20 mL), the precipitate was collected by filtration and washed with MeOH (20 mL). The crude product was further purified by silica gel column chromatography (eluent: dichloromethane) to give compound ***f*-P3Ng2a** (1.0 mg, 67% yield) as purple solid. MALDI-TOF MS (positive) *m/z*: $[M]^+$ calcd for $C_{300}H_{208}N_{12}Ni_3$ 4151.47, found 4151.86; UV-vis (chloroform, 298 K): λ (ε) = 360 nm (8.65×10^4 M^{-1} cm^{-1}), 417 nm (6.90×10^4 M^{-1} cm^{-1}), 543 nm (1.07×10^5 M^{-1} cm^{-1}), 573 nm (1.29×10^5 M^{-1} cm^{-1}), 732 nm (4.79×10^4 M^{-1} cm^{-1}), 819 nm (6.36×10^4 M^{-1} cm^{-1}), 884 nm (4.38×10^4 M^{-1} cm^{-1}), and 1010 nm (1.33×10^5 M^{-1} cm^{-1}).

Synthesis of ***f*-P1Ng1b**

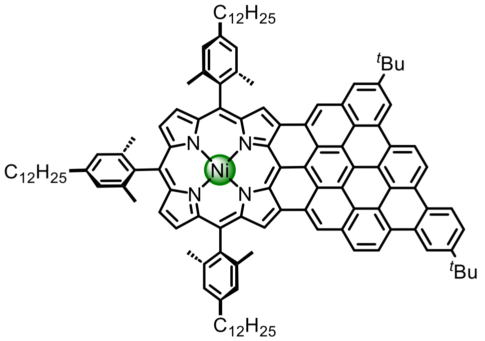

To a solution of **25b** (3.0 mg, 1.7 μmol) and DDQ (3.0 mg, 13 μmol) dissolved in dry degassed dichloromethane (10 mL) was added triflic acid (0.15 mL). The mixture was stirred at 22 °C for 2 h, then triethylamine (0.4 mL) was added via a syringe to quench the reaction. After addition of methanol (20 mL), the precipitate was collected by filtration and washed with MeOH (20 mL). The crude product was further purified by silica gel column chromatography (eluent: dichloromethane) to give compound ***f*-P1Ng1b** (1.5 mg, 50% yield) as purple solid. It is impossible to get well-resolved ^{1}H NMR spectra because of strong aggregation in solvents. MALDI-TOF MS (positive) *m/z*: $[M]^+$ calcd for $C_{122}H_{134}N_4Ni$ 1713.00, found 1712.92; UV-vis (1,2,4-trichlorobenzene, 298 K): λ (ε) = 323 nm (2.71 × 10^4 M^{-1} cm^{-1}), 373 nm (2.20 × 10^4 M^{-1} cm^{-1}), 417 nm (3.18 × 10^4 M^{-1} cm^{-1}), 454 nm (2.50 × 10^4 M^{-1} cm^{-1}), 532 nm (3.96 × 10^4 M^{-1} cm^{-1}), 597 nm (1.31 × 10^4 M^{-1} cm^{-1}), 727 nm (1.46 × 10^4 M^{-1} cm^{-1}), and 807 nm (3.32 × 10^4 M^{-1} cm^{-1}).

Synthesis of ***f*-P2Ng1b**

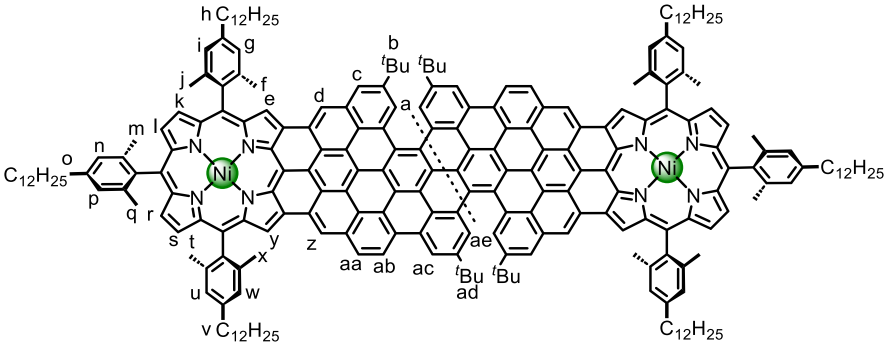

To a solution of **26b** (4.50 mg, 1.30 μmol) and DDQ (5.33 mg, 21 μmol) dissolved in dry degassed dichloromethane (15 mL) was added triflic acid (0.15 mL). The resulting mixture was stirred at 22 °C for 24 h, then triethylamine (0.4 mL) was added via a syringe and the resulting mixture was stirred for another 10 min to quench the reaction. After addition of methanol (20 mL), the precipitate was collected by filtration and washed with MeOH (20 mL). The crude product was further purified by silica gel column chromatography (eluent: dichloromethane) to give compound ***f*-P2Ng1b** (3.2 mg, 72% yield) as purple solid. ^{1}H NMR (600 MHz, CD_2Cl_2/CS_2 = 1/1, *v*/*v*, 298 K) δ 10.17 (s, 2H; **a**), 10.11 (s, 2H; **z**), 9.95 (d, *J* = 9.8 Hz, 2H; **ab**), 9.84 (s, 2H; **ae**), 9.78 (s, 2H; **d**), 9.61 (s, 2H; **ac**), 9.48 (s, 2H; **y**), 9.41 (s, 2H; **e**), 9.29 (d, *J* = 8.2 Hz, 2H; **aa**), 8.77 (s, 2H; **d**), 8.48 (s, 4H; **k/s**), 8.37 (d, J = 3.8 Hz, 4H; **l/r**), 7.45 (s, 2H; **g** or **i**), 7.43 (s, 2H; **i** or **g**), 7.41 (s, 2H; **u** or **w**), 7.38 (s, 2H; **w** or **u**), 7.26 (s, 2H; **n** or **p**), 7.23 (s, 2H; **p** or **n**), 3.02 – 3.00 (m, 4H; dodecyl-H), 2.86 (m, 8H; dodecyl-H), 2.23 (s, 12H; -Me), 2.06 (s, 12H; -Me), 2.04 (s, 8H; dodecyl-H), 1.93 (s, 12H; -Me), 1.90 (d,

J = 7.4 Hz, 16H; dodecyl-H), 1.72 – 1.70 (m, 16H; dodecyl-H), 1.62 (s, 32H; dodecyl-H), 1.54 (s, 18H; **ad**), 1.47 (s, 18H; **b**), 0.96 (s, 6H; dodecyl-H), 0.92 (s, 12H; dodecyl-H); MALDI-TOF MS (positive) *m/z*: $[M]^+$ calcd for $C_{244}H_{262}N_8Ni_2$ 3419.95, found 3420.99; UV-vis (1,2,4-trichlorobenzene, 298 K): λ (ε) = 383 nm (4.81 × 10^4 M^{-1} cm^{-1}), 418 nm (4.87 × 10^4 M^{-1} cm^{-1}), 465 nm (3.44 × 10^4 M^{-1} cm^{-1}), 538 nm (7.42 × 10^4 M^{-1} cm^{-1}), 745 nm (3.35 × 10^4 M^{-1} cm^{-1}), and 836 nm (6.30 × 10^4 M^{-1} cm^{-1}).

Synthesis of ***f*-P3Ng2b**

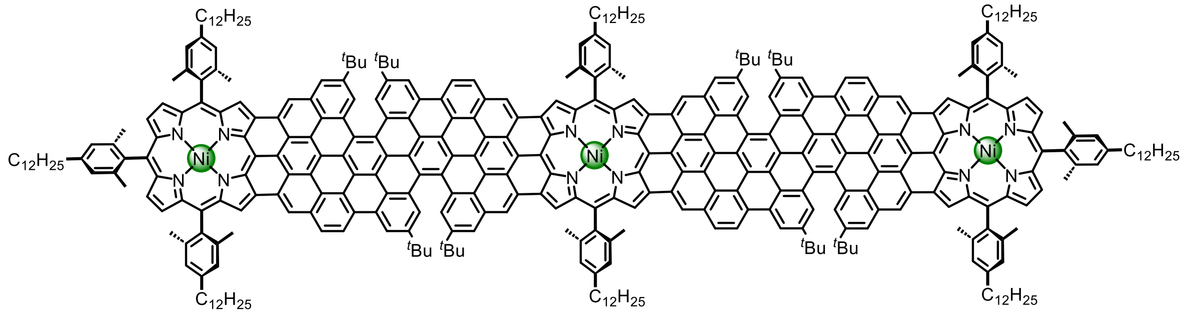

To a solution of **27b** (0.8 mg, 0.2 μmol) and DDQ (1.2 mg, 5.3 μmol) dissolved in dry degassed dichloromethane (5 mL) was added triflic acid (0.05 mL). The reaction mixture was stirred at 22 °C for 19 h, then triethylamine (0.4 mL) was added via a syringe to quench the reaction. After addition of methanol (50 mL), the precipitate was collected by filtration and washed with MeOH (30 mL). The crude product was further purified by silica gel column chromatography (eluent: dichloromethane) to give compound ***f*-P3Ng2b** (0.57 mg, 72% yield) as purple solid. MALDI-TOF MS (positive) *m/z*: $[M]^+$ calcd for $C_{388}H_{384}N_{12}Ni_3$ 5384.85, found 5384.51 (100%); UV-vis (1,2,4-trichlorobenzene, 298 K): λ (ε) = 420 nm (7.32 × 10^4 M^{-1} cm^{-1}), 458 nm (6.17 × 10^4 M^{-1} cm^{-1}), 544 nm (8.18 × 10^4 M^{-1} cm^{-1}), 578 nm (9.76 × 10^4 M^{-1} cm^{-1}), 740 nm (3.34 × 10^4 M^{-1} cm^{-1}), 824 nm (4.12 × 10^4 M^{-1} cm^{-1}), 886 nm (3.27 × 10^4 M^{-1} cm^{-1}), and 1019 nm (1.02 × 10^5 M^{-1} cm^{-1}).

Synthesis of **PPa**

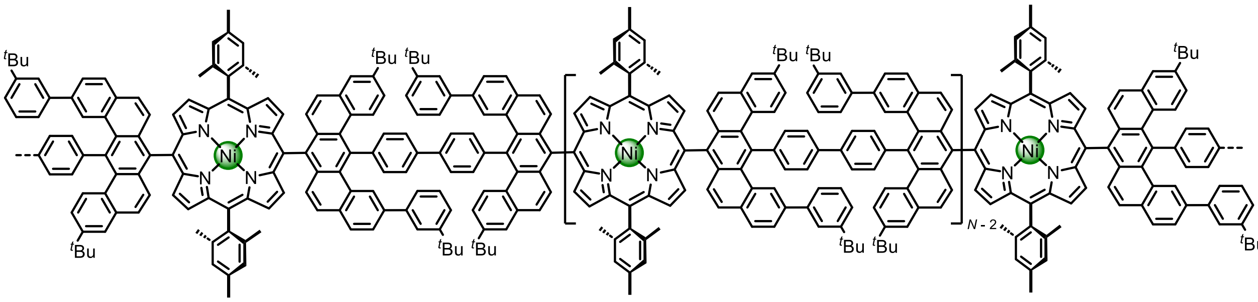

Dichloroporphyrin **2a** (5.0 mg, 2.9 μmol), 2,2'-bipyridine (4.5 mg, 29 μmol), and 1,5-cyclooctadiene (3.1 mg, 29 μmol) were dissolved in DMF (1 mL). The mixture was degassed by three freeze-pump-thaw cycles, then $Ni(COD)_2$ (7.7 mg, 29 μmol) was added under argon flow. The mixture was degassed by one more time freeze-pump-thaw and heated at 60 °C for 30 min, then heated at 110 °C for 24 h in dark. After cooling to room temperature, the solution was diluted with dichloromethane and passed through a short plug of silica (eluent: dichloromethane). The solvent was evaporated and the residue was purified by preparative size exclusion chromatography (Bio-Beads S-X1, toluene), followed by recrystallization from dichloromethane and methanol

to give **PPa** (4.5 mg, 94% yield) as red solid. ^{1}H NMR (600 MHz, CD_2Cl_2) δ 8.68 – 8.52 (m, 4H; β-H), 8.52 – 8.41 (m, 4H; β-H), 8.37 – 8.20 (m, 5H), 8.16 – 7.99 (s, 4H), 7.90 – 7.83 (m, 2H), 7.82 – 7.71 (m, 6H), 7.61 – 7.52 (m, 2H), 7.46 – 7.32 (m, 6H), 7.32 – 7.24 (m, 4H), 7.24 – 7.12 (m, 5H), 7.11 – 6.91 (m, 6H), 2.66 – 2.44 (m, 6H; Me-H), 2.11 – 1.73 (m, 12H; Me-H), 1.45 – 1.36 (m, 18H; ^{t}Bu-H), 1.36 – 1.29 (m, 18H; ^{t}Bu-H); ^{13}C NMR (150 MHz, CD_2Cl_2) δ 151.91, 149.90, 144.75, 143.33, 141.56, 139.28, 138.42, 137.33, 135.58, 134.66, 134.25, 133.26, 132.26, 131.89, 130.52, 130.10, 129.84, 129.38, 129.28, 128.99, 128.57, 128.18, 127.69, 126.14, 125.65, 125.44, 125.27, 124.93, 124.75, 118.40, 35.16, 35.11, 34.92, 31.64, 31.61, 31.51, 31.37, 21.62, 21.58, 21.50; UV-vis (chloroform, 298 K): λ (ε, , normalized to number of porphyrin unit) = 323 nm (1.08×10^5 M^{-1} cm^{-1}), 430 nm (2.16×10^5 M^{-1} cm^{-1}), 531 nm (2.90×10^4 M^{-1} cm^{-1}), and 559 nm (7.93×10^3 M^{-1} cm^{-1}).

<u>Synthesis of **PPb**</u>

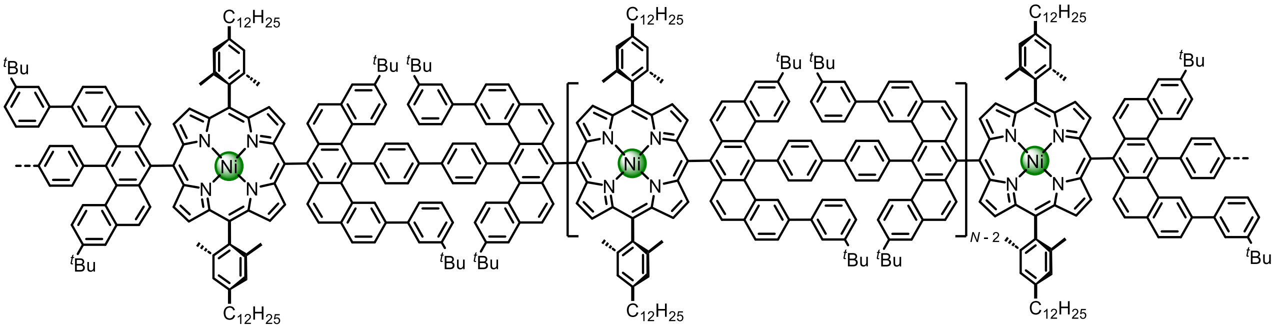

Dichloroporphyrin **22b** (7.21 mg, 3.50 µmol), 2,2'-bipyridine (5.46 mg, 35.0 µmol), and 1,5-cyclooctadiene (4.3 µL, 35 µmol) were dissolved in THF (0.6 mL). The mixture was degassed by three freeze-pump-thaw cycles, then $Ni(COD)_2$ (9.48 mg, 35 µmol) was added under argon flow. The mixture was degassed by one more time freeze-pump-thaw, then heated at 85 °C for 20 h in dark. After cooling to room temperature, the solution was diluted with dichloromethane and passed through a short plug of silica (eluent: dichloromethane). The solvent was evaporated and the residue was further purified by preparative size exclusion chromatography (Bio-Beads S-X1, toluene), followed by recrystallization from dichloromethane and methanol to give **PPb** (6.5 mg, 93% yield) as red solid. ^{1}H NMR (600 MHz, CD_2Cl_2, 298 K) δ 8.62 – 8.54 (m, 4H; β-H), 8.37 – 8.24 (m, 4H; β-H), 8.37 – 8.24 (m, 5H), 8.22 – 8.00 (m, 4H), 7.94 – 7.83 (m, 2H), 7.81 – 7.74 (m, 6H), 7.61 – 7.52 (m, 2H), 7.42 – 7.34 (m, 6H), 7.31 – 7.25 (m, 4H), 7.24 – 7.16 (m, 5H), 7.11 – 7.00 (m, 6H), 2.84 – 2.71 (m, 4H; dodecyl-H), 2.00 – 1.85 (m, 12H; Me-H), 1.84 – 1.78 (m, 4H; dodecyl-H), 1.50 – 1.46 (m, 4H; dodecyl-H), 1.42 – 1.38 (m, 24H; dodecyl-H), 1.34 – 1.28 (m, 40H; dodecyl-H/^{t}Bu-H), 0.87 – 0.85 (m, 6H; dodecyl-H); ^{13}C NMR (150 MHz, CD_2Cl_2, 298 K) δ 151.91, 149.89, 145.94, 144.72, 144.69, 143.45, 143.32, 143.28, 141.74, 141.57, 139.19, 138.42, 135.58, 134.93, 134.77, 134.68, 134.55, 134.29, 133.30, 133.10, 132.51, 132.26, 131.90, 130.52, 130.13, 129.86, 129.29, 129.00, 128.79, 128.21, 127.65, 127.51, 126.23, 126.15, 126.13, 125.48, 125.45, 125.38, 125.27, 124.95, 118.50, 116.02, 115.83, 36.31, 35.11, 34.92, 32.33, 32.01, 31.62, 31.52, 31.37, 30.13, 30.12, 30.08, 30.04, 29.99, 29.77, 23.10, 21.70, 21.67, 14.30; Solid-state ^{1}H NMR (400 MHz, 300 K) δ 16.08 – 3.79 (m, 48H), 3.63 – –7.65 (s, 98H); ^{13}C NMR (101 MHz, 300 K) δ 148.61, 143.27, 137.33, 133.77, 130.60, 127.43, 117.54, 34.21, 30.84, 22.33, 13.42; UV-vis (chloroform, 298 K): λ (ε,

normalized to number of porphyrin unit) = 323 nm (9.09×10^4 M^{-1} cm^{-1}), 430 nm (1.90×10^5 M^{-1} cm^{-1}), and 531 nm (2.05×10^4 M^{-1} cm^{-1}).

Synthesis of **PGNRb**

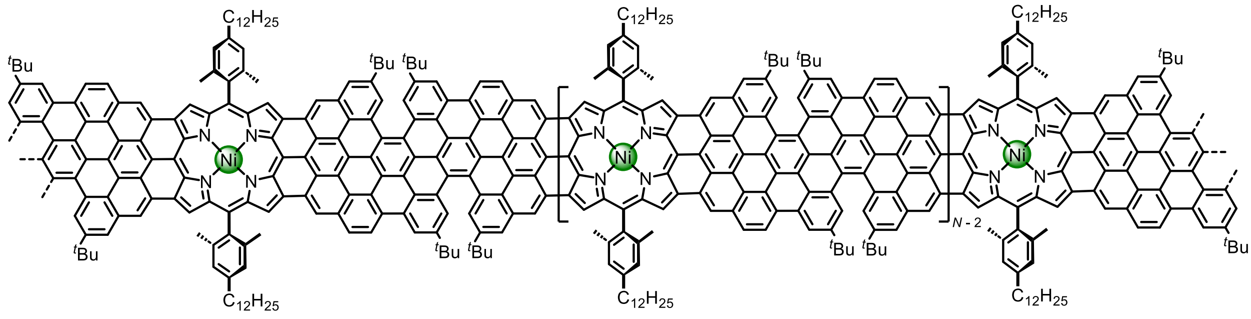

To a solution of **PPb** (4.51 mg, 2.26 μmol) and DDQ (7.70 mg, 33.9 μmol) dissolved in dry degassed dichloromethane (25 mL) was added triflic acid (0.25 mL). The reaction mixture was stirred at 22 °C for 24 h, then triethylamine (1.0 mL) was added via a syringe to quench the reaction. After addition of methanol (30 mL), the precipitate was collected by filtration and washed with MeOH (30 mL), then saturated $NaHCO_3$ solution (100 mL), water (50 mL) and methanol (50 mL) to give **PGNRb** (4.20 mg, 94% yield) as black solid. Solid-state ^1H NMR (400 MHz, 300 K) δ 20.93 – 5.69 (m, 24H; aromatic protons), 5.51 – –6.90 (m, 98H; aliphatic protons); Solid-state ^{13}C NMR (100 MHz, 298 K) δ 148.18, 137.39, 128.89, 123.58, 30.89, 20.79, 12.78; UV-vis (1,2,4-trichlorobenzene, 298 K): λ (ε, normalized to number of porphyrin unit) = 584 nm (4.24×10^4 M^{-1} cm^{-1}) and 1044 nm (4.07×10^4 M^{-1} cm^{-1}).

2. X-Ray Structure

Compound **12** was crystallized by slow evaporation of its solution in a mixture of dichloromethane and methanol to give thin colorless needles. A suitable crystal with dimensions of $0.46 \times 0.06 \times 0.01$ mm^3 was selected and mounted on a MiTeGen 200 μm loop using Fomblin YR-1800 oil on Rigaku Supernova A diffractometer using a copper radiation source. The crystal was kept at 150 K during data collection. The structure was solved with the ShelXT 2018/2 solution program[9] using dual methods and by using Olex2 1.5[10] as the graphical interface. The model was refined with ShelXL 2018/3[11] using full matrix least squares minimization on F^2.

Two ^{t}Bu groups are disordered and were modelled using similar distance (SADI) and ADP (SIMU, RIGU) restraints. One of the phenyl rings is disordered too and was modelled similarly, additionally using FLAT command on both components of disorder. Small residual electron density in voids (6 electrons per cell in total) was accounted for, using solvent mask (SQUEEZE, Platon)[12].

X-ray structure of **12** – CCDC: 2225521

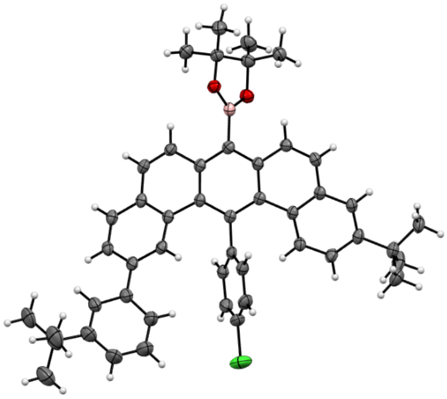

Supplementary Figure 7. Thermal ellipsoid drawing of the molecular structure of **12** at 50% probability. Atom colors: H, white; B, pink; C, gray; O, red; Cl, green. Disorder atoms are omitted for clarity.

Supplementary Table 1. Crystal data and structure refinement for **12**.

CCDC code	2225521
Identification code	039wps22
Empirical formula	$C_{48}H_{48}BClO_2$
Formula weight	703.12
Temperature/K	150.01(10)
Crystal system	monoclinic
Space group	P2/c
a/Å	27.0823(9)
b/Å	6.1137(2)
c/Å	24.9379(7)
α/°	90
β/°	105.412(3)
γ/°	90
Volume/Å^3	3980.6(2)
Z	4
ρ_{calc}g/cm^3	1.173
μ/mm^{-1}	1.129
F(000)	1496
Crystal size/mm^3	0.46 × 0.06 × 0.01
Radiation	Cu Kα (λ = 1.54184)
2Θ range for data collection/°	7.232 to 140.15
Index ranges	$-31 \le h \le 33$, $-7 \le k \le 7$, $-30 \le l \le 21$
Reflections collected	32789
Independent reflections	7584 [R_{int} = 0.0520, R_{sigma} = 0.0425]
Data/restraints/parameters	7584/651/586
Goodness-of-fit on F^2	1.033
Final R indexes [I >= 2σ (I)]	R_1 = 0.0453, wR_2 = 0.1132
Final R indexes [all data]	R_1 = 0.0674, wR_2 = 0.1281
Largest diff. peak/hole / e Å^{-3}	0.25/-0.22

3. Chiral Resolution

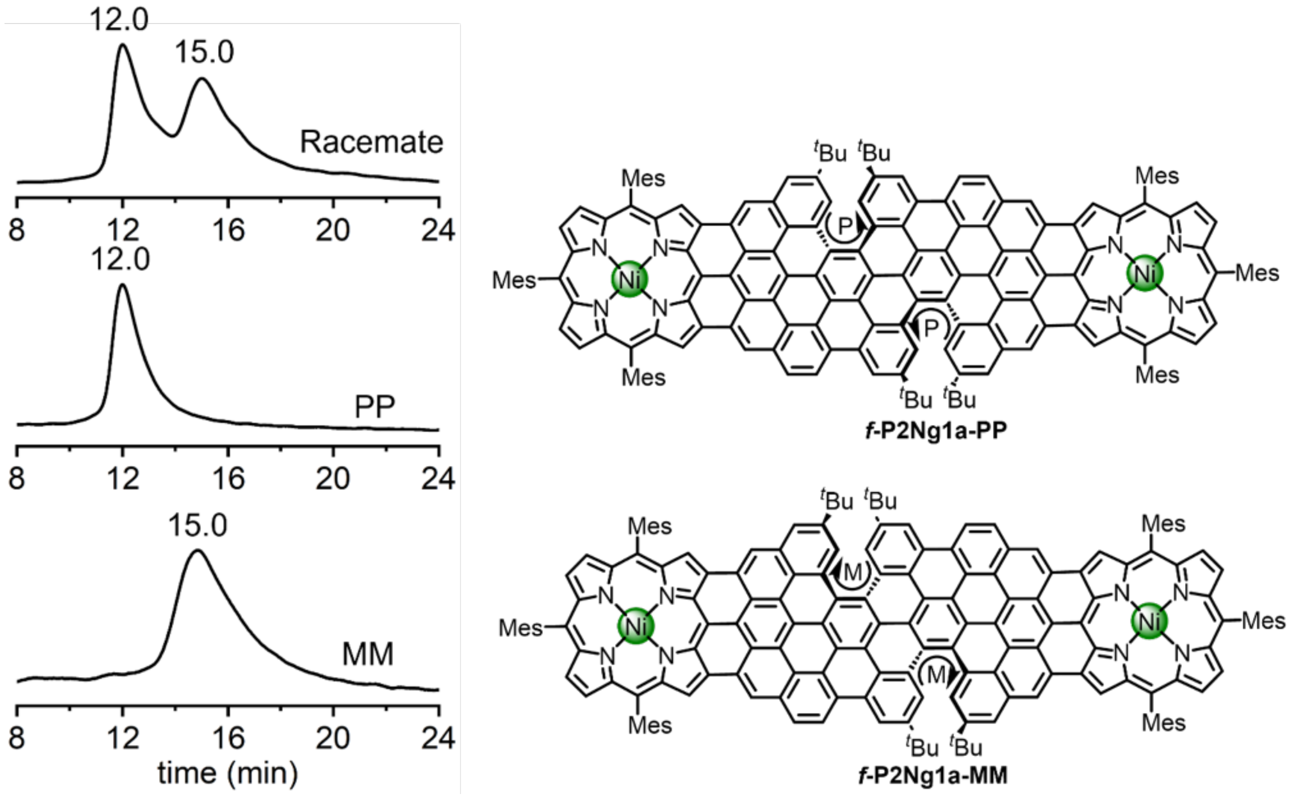

Supplementary Figure 8. Chiral HPLC traces of racemic mixtures of ***f*-P2Ng1a** and separated two enantiomers with PP and MM configuration. Chiral resolution of ***f*-P1Ng1a** was conducted at 298 K on an Agilent 1260 infinity liquid chromatography system equipped with a Chiralpak® ID column (250 × 4.6 mm). Eluent: *n*-hexane/isopropanol/dichloromethane = 96/2/2, *v*/*v*; flow rate: 0.6 mL/min; detected by the absorption at 378 nm. The right side shows the absolute structures of two enantiomers of ***f*-P1Ng1a**.

4. GPC Measurements

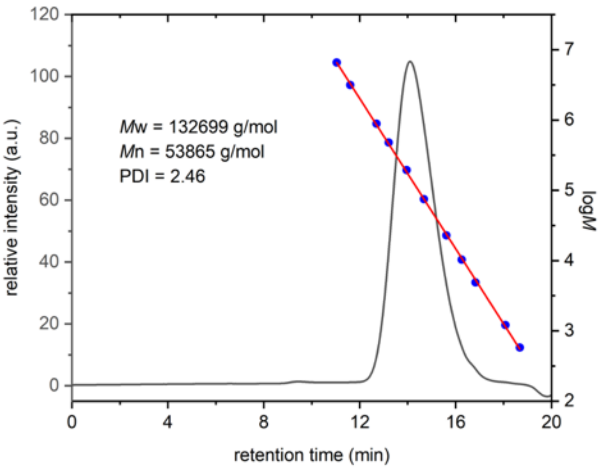

Supplementary Figure 9. GPC trace (detection wavelength = 250 nm, flow rate = 1.0 mL/min) of **PPb** in chloroform measured at 40 °C calibrated with low dispersity (PDI < 1.10) polystyrene standards.

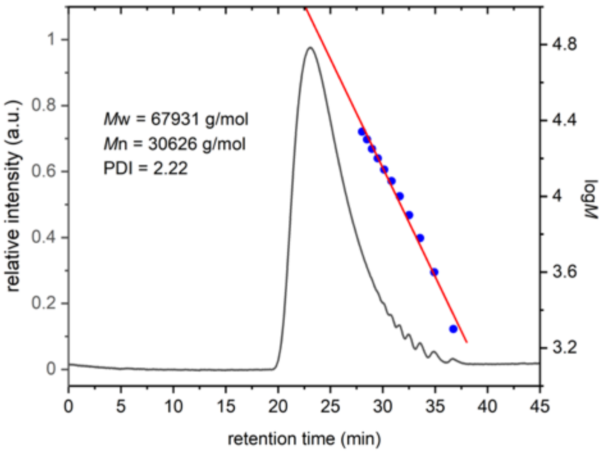

Supplementary Figure 10. GPC trace (detection wavelength = 430 nm, flow rate = 1.0 mL/min) of **PPb** in tetrahydrofuran/pyridine = 100/1 (*v*/*v*) measured at 25 °C calibrated with porphyrin oligomers (N = 1–11).

5. STM Characterization

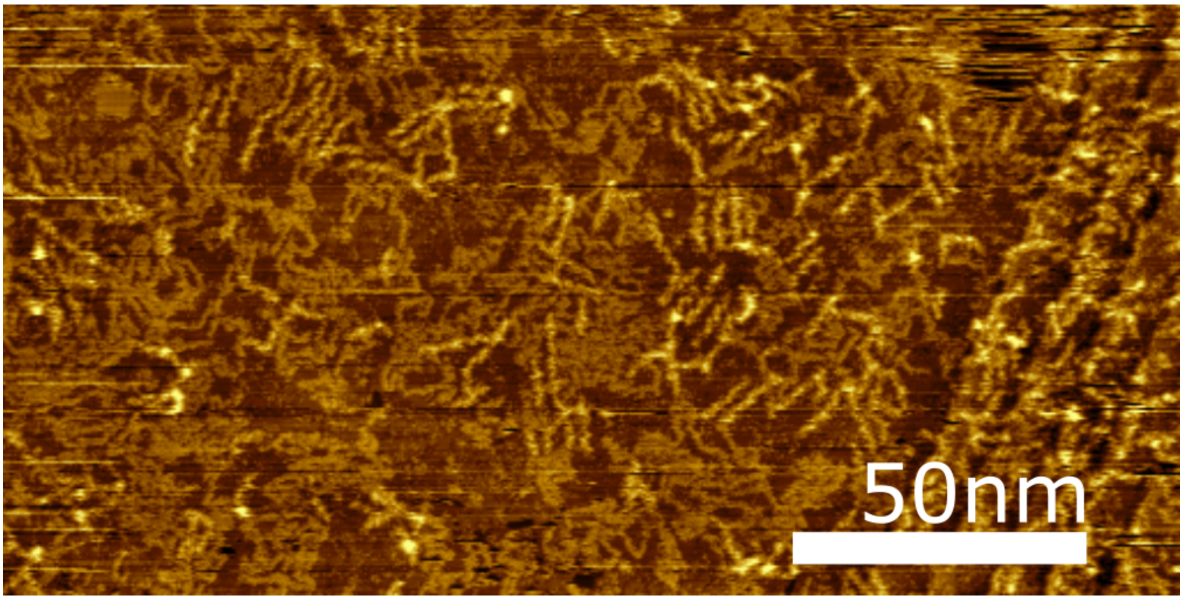

Supplementary Figure 11. Topograph of **PPb** transferred from solution to a Au(111) surface by electrospray (T_{sample} = 4.7 K, $V_{sample\text{-}bias}$ = −1.5 V, $I_{set\text{-}point}$ = 50 pA). Ribbon-like structures are observed; ribbons with a darker contrast are assigned to 'flat-lying' **PPb**, while features with a brighter contrast may be elevated from the surface by underlying material.

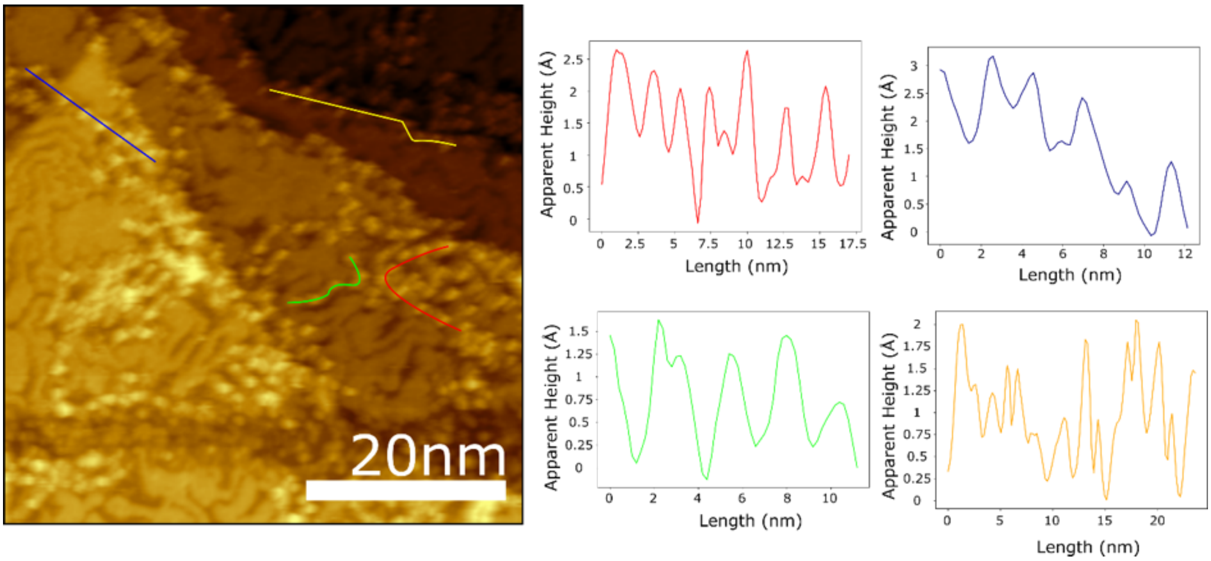

Supplementary Figure 12. STM topograph showing an overview of the Au(111) surface with electrospray deposited **PPb** following annealing to 150 °C (T_{sample} = 4.7 K, $V_{sample\text{-}bias}$ = −2 V, $I_{set\text{-}point}$ = 50 pA). Height profiles are acquired along periodic chain structures (shown to right of STM image, position of profiles indicated with coloured lines). The average peak separation along these chains is 2.5 ± 0.6 nm.

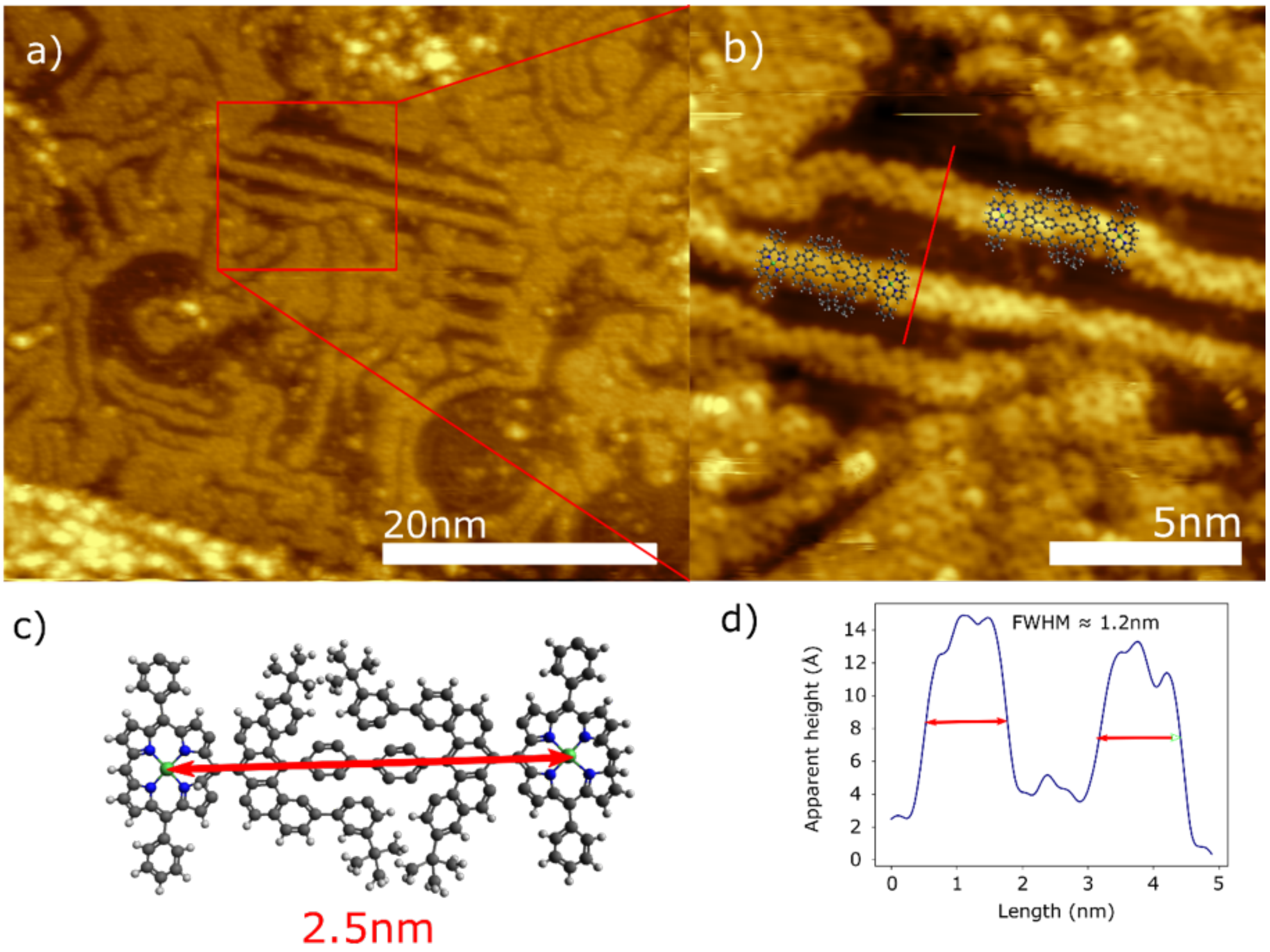

Supplementary Figure 13. STM topographs showing details of **PPb** on Au(111) following electrospray deposition and annealing to 150 °C. **a**, Ribbon-like structures observed in individual straight (and curved) arrangements, as well as in close-packed islands (T_{sample} = 4.7 K, $V_{sample\text{-}bias}$ = –1.6 V, $I_{set\text{-}point}$ = 100 pA). **b**, STM topograph acquired in the region highlighted in (**a**) showing linear segments of ribbon structure. A model of **PPb** (with alkyl chains removed for clarity) is overlaid (T_{sample} = 4.7 K, $V_{sample\text{-}bias}$ = –2 V, $I_{set\text{-}point}$ = 100 pA). **c**, Molecular model of a section of **PPb** (not including alkyl chains). (d) Line profile acquired over two parallel ribbon features, shown in (**a**); the FWHM of these two features is measured to be ≈ 1.2 nm, compatible with the width of the nanoribbon.

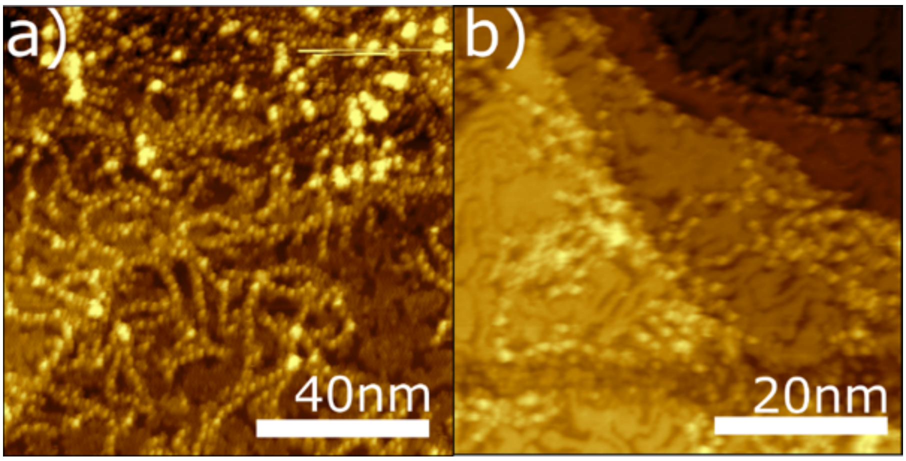

Supplementary Figure 14. STM topographs of the Au(111) surface following deposition of, (**a**) **PPa** with subsequent annealing to 250 °C and, (**b**) **PPb** with subsequent annealing to 150 °C. (T_{sample} = 4.7 K, $V_{sample\text{-}bias}$ = –2 V, $I_{set\text{-}point}$ = 50 pA). A similar variation in contrast along the 'bright' ribbons is observed. Separation between features along chains in (a) is in the range 1.8–2.5 nm.

6. Solid-State NMR Measurements

The 2D $^1H–^1H$ double quantum-single quantum (DQ-SQ) solid-state NMR correlation experiments provide information about the spatial proximity between different protons[13]. As shown in Supplementary Figure 15c, the 2D $^1H–^1H$ DQ-SQ spectrum of **PPb** exhibits relatively narrow correlation signals between protons on the aromatic groups (phenyl/porphyrin) as well as between the protons on the aromatic groups and the aliphatic groups (dodecyl/methyl/*tert*-butyl), because of the flexible 3-dimensional structure of the polymer precursor. However, for the 2D $^1H–^1H$ DQ-SQ spectrum of **PGNRb** displayed in Supplementary Figure 15d, a broad and stretched correlation signal was observed between aliphatic protons and the correlation signals between aromatic protons, characteristic for **PPb**, were attenuated, as expected for the successful removal of aromatic protons in **PGNRb**. The observed broadening of the ^1H NMR signals of **PGNRb** further demonstrates that in the solid state the molecules are packed heterogeneously, which results in the shifts of ^1H NMR signals in opposite directions arising from the shielding/de-shielding effect of stacked PGNRs. In the CP-MAS ^{13}C NMR spectra of **PPb** shown in Supplementary Figure 16, signals at 125–135 ppm mainly come from aromatic carbons attached to protons, while the other signals come from quaternary carbon atoms. After planarization, the signals of C-H carbons decreased and quaternary carbons enhanced, indicating the removal of hydrogens from **PPb**. Moreover, the whole spectrum also become broad due to the formation of large π-systems that shield/de-shield the carbons of the stacked **PGNRb** backbone and shift the ^{13}C NMR signals to different directions.

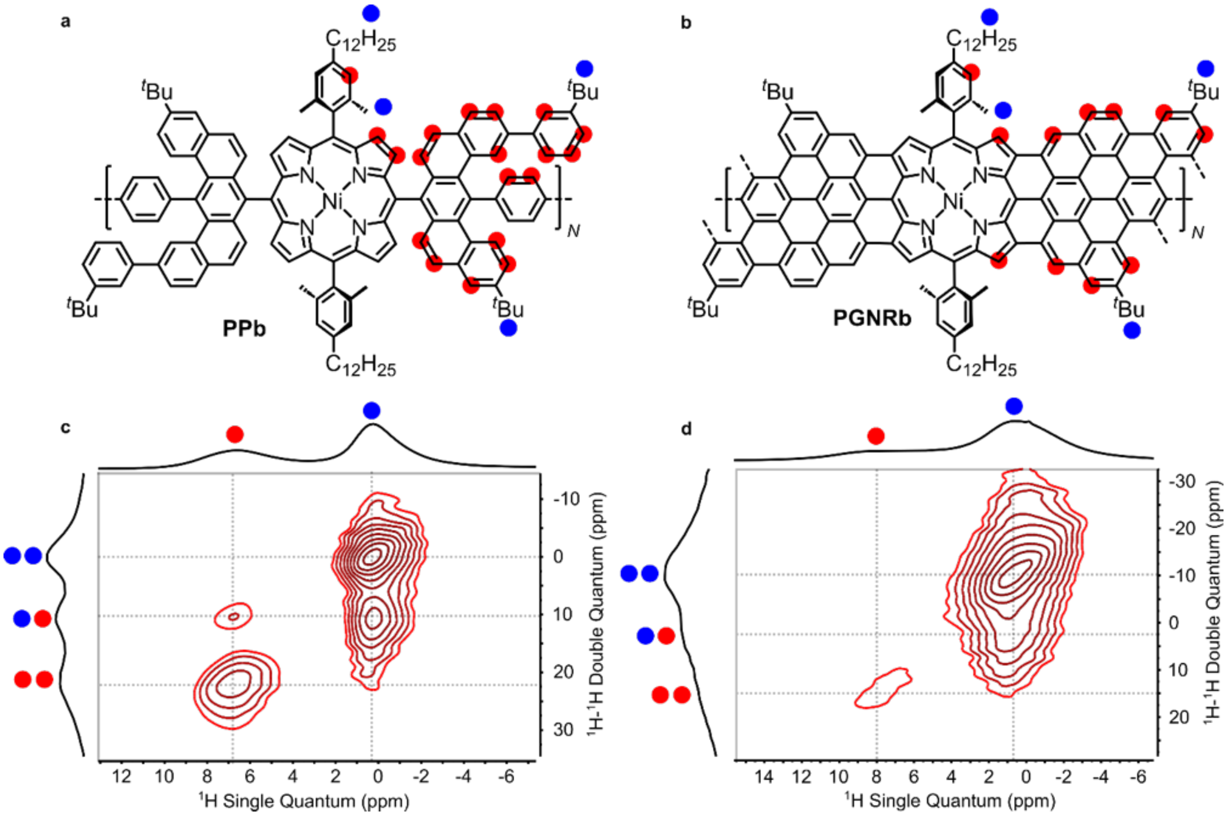

Supplementary Figure 15. a,b) Chemical structures of **PPb** and **PGNRb**. c,d) 2D ^1H-^1H DQ-SQ correlation spectra for **PPb** and **PGNRb** recorded using compensated Back-to-Back (BaBa) sequence with 1 rotor period dipolar recoupling. 128 scans, 2048 points and 64 increments were acquired using a 3.5 μs π/2 pulse and 2.5 s recycle delay. Both spectra were recorded at 400 MHz (300 K) using a MAS frequency of 20 kHz.

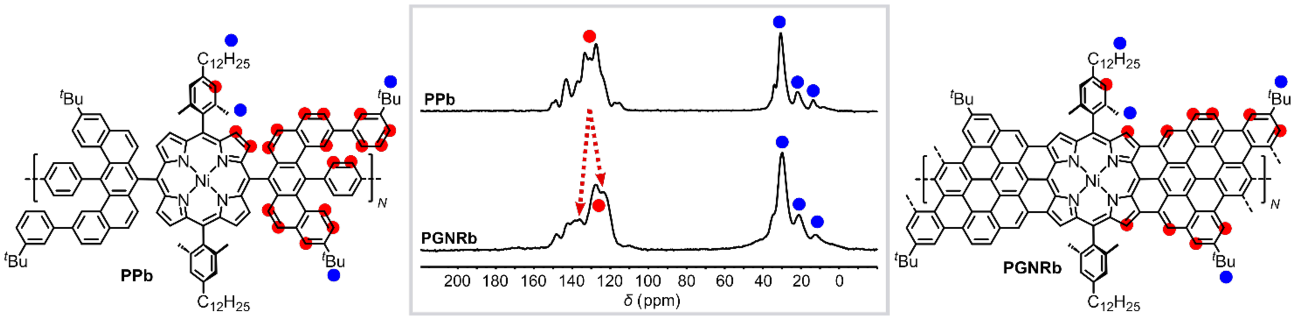

Supplementary Figure 16. Solid-state CP-MAS ^{13}C NMR spectra of **PPb** and **PGNRb** (101 MHz, 300 K) recorded using a MAS rate of 12 kHz and a sequence with a variable X-amplitude spin-lock pulse and spinal64 proton decoupling. 24000 transients were acquired using a contact time of 2.5–5.0 ms, an acquisition time of 25 ms (2048 data points zero filled to 24 K) and a recycle delay of 0.5–2.0 s.

7. FT-IR and Raman Spectroscopy

FT-IR spectra of **PPb** and **PGNRb** before and after the planarization reveal attenuation of the out-of-plane aromatic C-H bending bands located at 706, 716, 724, 730, 797 and 837 cm^{-1}, which are typical for *meta*- and *para*-disubstituted phenyl groups, and weaker signals of the aromatic C-H bond stretching modes between 3000 and 3100 cm^{-1}. These observations are in good agreement with the expected dehydrogenation.

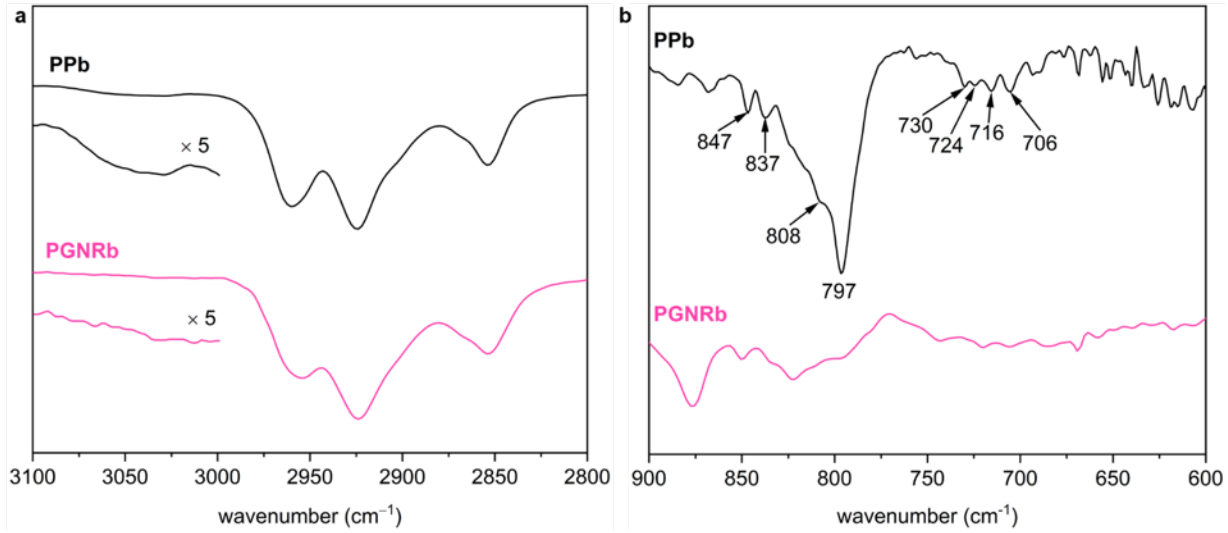

Supplementary Figure 17. Comparison of FT-IR spectra of polyphenylene precursor **PPb** and porphyrin-fused graphene nanoribbon **PGNRb** after cyclodehydrogenation, showing the disappearance of aromatic C-H vibration modes (~3050 cm^{-1}) (**a**) and phenyl rings out-of-plan C-H bending modes located at 706, 716, 724, 730, 797 and 837 cm^{-1}, which are typical for *meta*- and *para*- disubstituted phenyl groups (**b**).

The Raman spectra of the polymer precursor **PPb** only has broad C-H stretching bands in the region of 2000–3250 cm^{-1}, and the intensity of these peaks decreases significantly after planarization. The Raman spectra of **PGNRb** displays four main regions, with the center peaks located at 1001, 1214, 1315 and 1576 cm^{-1}, that are also present in the model ***f*-P2Ng1b**, ***f*-P3Ng2b**, reflecting their similar structural motifs.

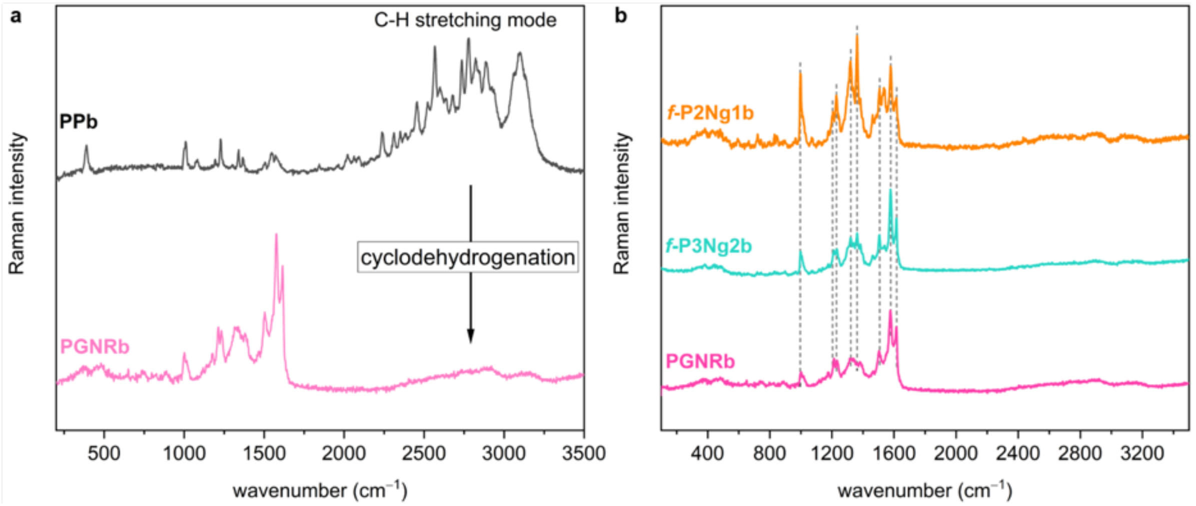

Supplementary Figure 18. Comparison of Raman spectra of **a**) **PPb** and **PGNRb**, which exhibits disappearance of C-H stretching modes located in the range of 2000 – 3250 cm^{-1}, indicating the successful removal of hydrogens from **PPb** after cyclodehydrogenation reaction; **b**) model compounds ***f*-P2Ng1b**, ***f*-P3Ng2b** and **PGNRb**, having the same characteristic peaks in the main D-band (~1350 cm^{-1}) and G-band (~1580 cm^{-1}), which reflects their structural similarity. All the samples were measured using 532 nm (2.33 eV) laser on a powder sample with power of 1.0 mW.

8. XPS Analysis

X-ray photoelectron spectroscopy (XPS) was applied to analyse the element composition of the polymer precursor (**PPb**) and **PGNRb**. The full scan spectra of **PPb**/**PGNRb** show expected signals for C1s (285.0/284.5 eV), N1s (398.9/398.8 eV) and Ni2p3 (855.7/855.1 eV). Their C1s peaks were measured and then deconvoluted using Gaussian-LorenCross curves. For **PPb**, the main peaks centred at 284.5 eV and 285.5 eV are assigned to C=C and C-C/C-H carbons, respectively. In **PGNRb**, these two peaks do not show significant changes. The component located at 288.8 eV corresponds to the π-π* shake up peaks of graphitic materials and does not exist in **PPb**[14]. This observation also reflects the successful formation of graphitic structure in **PGNRb**.

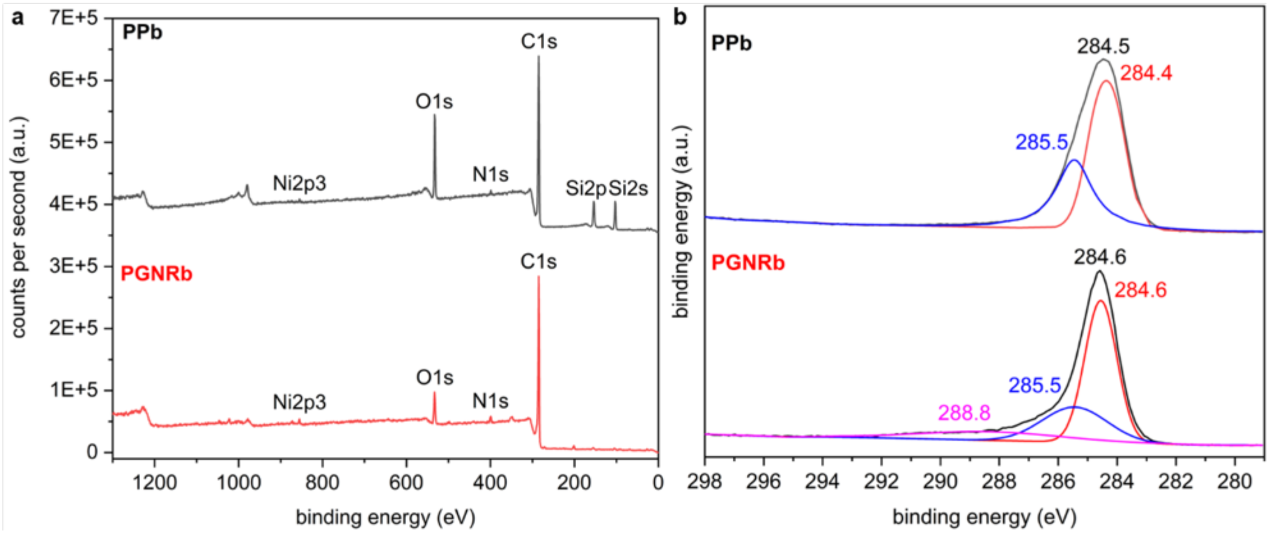

Supplementary Figure 19. a, XPS survey spectra of **PPb** (black) and **PGNRb** (red). **b**, High resolution XPS C1s spectra of **PPb** (black) and **PGNRb** (red). For **PPb**, the peak could be split to two Gaussian-LorenCross peaks located at 284.4 eV and 285.5 eV, respectively. For **PGNRb**, one broad peak centred at 288.8 eV appears, which belongs to the π-π* shake up peaks of graphitic materials.

9. UV-vis-NIR Absorption Spectra

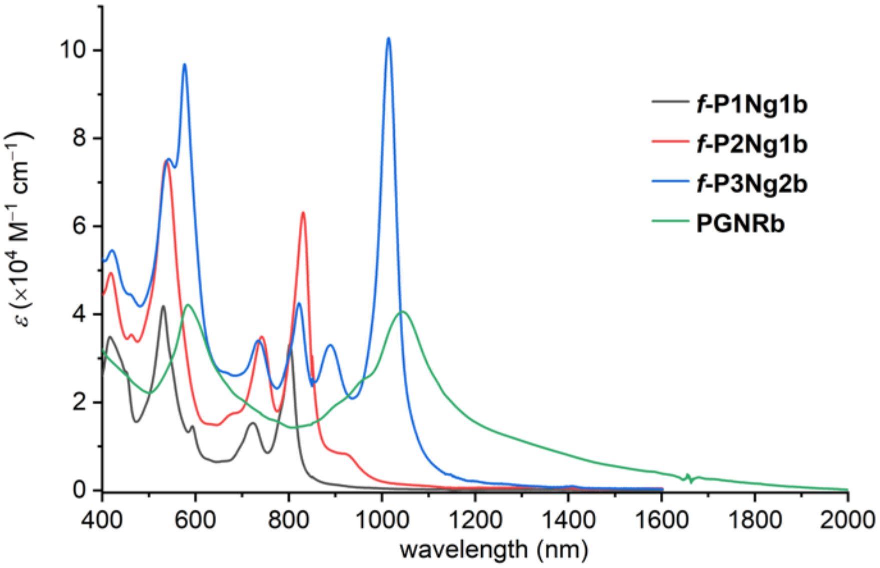

Supplementary Figure 20. UV-vis-NIR absorption spectra of ***f*-P1Ng1b**, ***f*-P2Ng1b**, ***f*-P3Ng2b**, and **PGNRb** measured in 1,2,4-trichlorobenzene at 298 K (the molar extinction coefficient of **PGNRb** is normalized to the number of porphyrins).

10. DFT Calculations

Density functional theory (DFT) calculations on fused porphyrin oligomers were performed using Gaussian 16/A.03 software package[15] and the B3LYP level of theory with the 6-31G(d,p) basis set for C, N, H atoms and LanL2DZ basis set for Ni atoms. TD-DFT calculations were carried out using B3LYP, BLYP35 and LC-wHPBE (w = 0.1) functionals and used polarizable continuum model (PCM) with chloroform as solvent. The simulated CD spectra of ***f*-P2Ng1a-MM** are sensitive to the selected functional and the LC-ωHPBE (ω = 0.1) provides spectra that agree best with the experimental results.

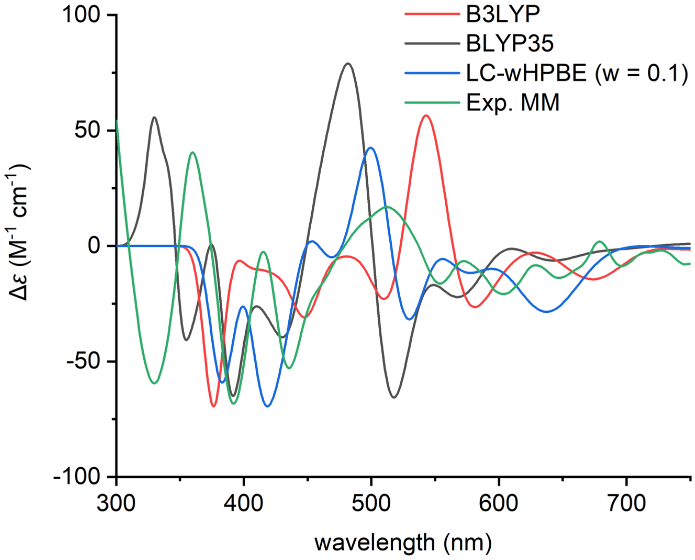

Supplementary Figure 21. Comparison of simulated CD spectra of ***f*-P2Ng1a-MM** with B3LYP, BLYP35, LC-ωHPBE (ω = 0.1) functionals and experimental result (chloroform, concentration 10^{-5} M).

The band structures were calculated using density functional theory (DFT) implemented in SIESTA[16]. We employed Perdue-Burke-Ernzerhof (PBE) generalized gradient approximation (GGA) functional[17] with DZP basis set. The energy shift of the localized basis was set to 100 meV. Energy cut-off of 1000 Ry and the Monkhorst-Pack grid of (50,1,1) were used to ensure the convergence of the results. A vacuum region of at least 30 Å is used in nonperiodic directions to prevent unwanted interactions. The structure was optimized until the maximum force on the atoms is less than 0.01 eV/Å. In the density of states (DOS) calculation Gaussian broadening of 0.01 eV is used for all bands.

The twisted GNR has large band gap (0.93 eV), while the fused Ni-porphyrin nanoribbon has very narrow bandgap (0.32 eV). When fusing the porphyrin unit and twisted GNR unit, the resulting PGNR has a moderate band gap (0.76 eV). The effective mass of PGNR is estimated excluding the d-orbital bands, since they are localized leading to flat bands that do not contribute to transport.

The frontier bands of PGNR resemble those of the GNR (without porphyrin units), except for the d-orbital bands above the valence band. The effective masses of PGNR (m_{VB} = 0.326, m_{CB} = 0.615) are closer to that of GNR (m_{VB} = 0.640, m_{CB} = 0.802), while much larger than that of fused Ni-porphyrin ribbon (m_{VB} = 0.054, m_{CB} = 0.053). The fjord structure of the GNR backbone hinders the delocalization of electron waves, increasing the effective mass of the charge carriers.

Two simplifications were made when calculating the band structures of GNR and PGNR. The first concerns sequential twists along the ribbon. A PGNR chain can be represented by a minimal repeat unit, as highlighted by the blue dashed box in Supplementary Figure 21. There is a twist between two adjacent repeat units and consecutive twists can be either clockwise or anticlockwise. In reality, the twists along a ribbon may be randomly clockwise/anticlockwise and the system lacks translational symmetry. To calculate the band structure, however, we have to use periodic boundary condition and assume translational symmetry. To overcome this problem, we define a supercell that is comprised of two minimal repeat units, as highlighted in red dashed boxed in Supplementary Figure 21. We assume that every clockwise twist is followed by an anticlockwise twist. By using this supercell as unit cell, we can set up the periodic boundary condition and calculate the band structures for GNR and PGNR.

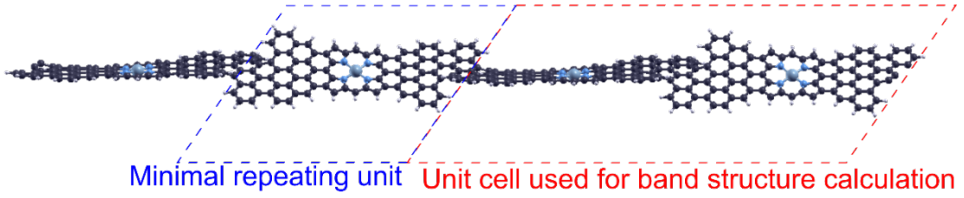

Supplementary Figure 22. Minimal repeating cell and unit cell used for band structure calculation.

The other simplification concerned the substituents on the nanoribbon. We investigated the effect on the band structure of different substituents at the fjord-edge, by considering ribbons with H or *t*-Bu substituents. The first structure shown in Supplementary Figure XX, flat-GNR, has no fjord edges and no twist. It has a small band gap and large dispersion in the conduction and valence bands. The other two structures have a fjord edge and H or *t*-Bu substituents. These two structures have very similar twist angles (GNR-H: 36.0°; GNR-H: 36.4°) and band structures. By comparing flat-GNR with GNR-H, we can see that removing the C-C bond at the fjord

edge has a dramatic effect on the band structure, whereas the effect of H versus *t*-Bu substituents is insignificant. Thus it is justified to replace the *t*-Bu groups with hydrogen atoms to simplify the band structure calculations.

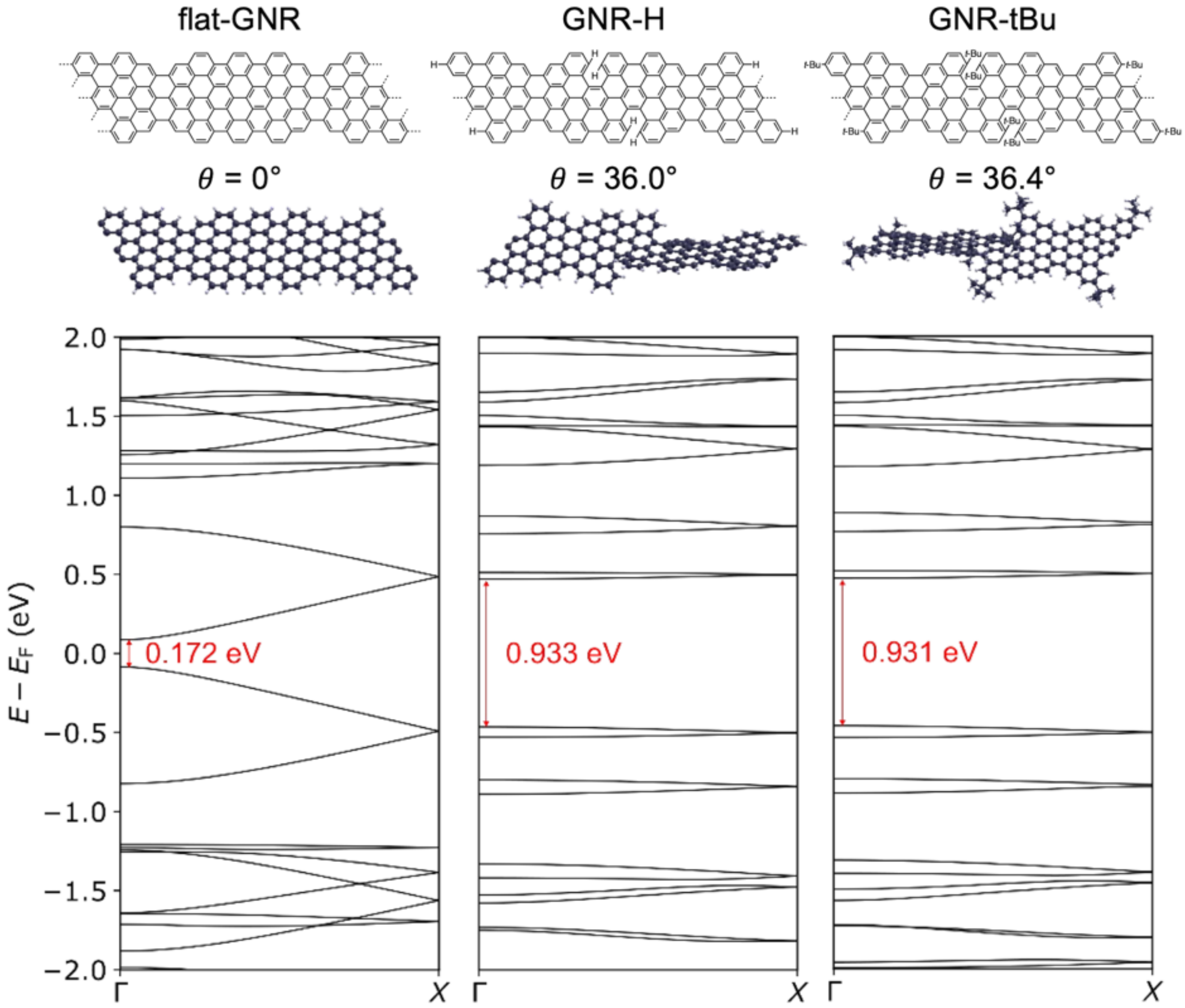

Supplementary Figure 23. Effect of substituents on band structure: flat-GNR has no fjord edge and no twist; GNR-H and GNR-tBu have the same fjord edge with -H or -*t*-Bu substituents, respectively. The band gaps are labelled in red. Angles are calculated between the mean planes of 18-atom hexabenzocoronene units at the ends of each ribbon.

Supplementary Table 2. Summary of the conduction-band (CB) effective mass m_{CB}, valence-band (VB) effective mass m_{VB} and effective reduced mass m^* from band structure calculations.

	GNR	PGNR	porphyrin ribbon
m_{CB}	0.802	0.615	0.053
m_{VB}	0.640	0.326	0.054
m^*	0.356	0.213	0.027

11. THz Spectroscopy

Experimental set-up for optical pump–THz probe spectroscopy

The optical pump-THz probe setup is driven by a commercial mode-locked titanium sapphire femtosecond laser with central wavelength of 800 nm, pulse duration of 50 fs and repetition rate of 1 kHz. The output laser is separated into three beamlines for THz generation, sampling and pump. The THz is generated by optical

rectification in a 1 mm <110>-oriented ZnTe crystal upon 800 nm laser impingement. This THz pulse will be focused and transmitted through the sample (**PGNRb** solution in 2 mm-thick cuvette or **PGNRb** film on fused silica substrate), and collected by a 90° off-axis parabolic mirror before re-focused onto another ZnTe crystal. The transmitted single cycle THz waveform is then detected on the second ZnTe crystal by a time-delayed weak 800 nm pulse through electrooptic sampling. To optically pump the sample, a frequency-doubled 400 nm pulse via barium borate crystal was employed to propagate collinearly through the sample with THz pulse. The relative time delay between THz pulse and pump pulse is realized by a mechanically adjustable delay stage.

Calculations of photoconductivity in solution and thin film

To extract the photoconductivity of **PGNRb** in solution in 2 mm-thick cuvette, a transfer matrix is applied through air, cuvette windows and unexcited (or excited) sample[18]. While for the thin film, we utilized thin film approximation:[19, 20]

$$\sigma(t) = -\frac{(n_1 + n_2)}{Z_0 \cdot l} \cdot \frac{\Delta E(t)}{E(t)} \tag{1}$$

Here, $\Delta E(t) = E_{\text{pump}}(t) - E(t)$ is the pump-induced THz electric field changes. n_1 and n_2 are the refractive indices of the media before and after the sample (in this work, n_1 and n_2 are 1 and 1.95 for vacuum and fused silica, respectively), $Z_0 = 377\ \Omega$ the impedance of free space, $l = 200$ nm the film thickness. By applying the Fourier transformation, the equation (1) is still valid in the frequency domain:

$$\sigma(t) = -\frac{(n_1 + n_2)}{Z_0 \cdot l} \cdot \frac{\Delta E(\omega)}{E(\omega)} \tag{2}$$

By doing so, we can obtain the frequency resolved photoconductivity.

Photoconductivity in thin films

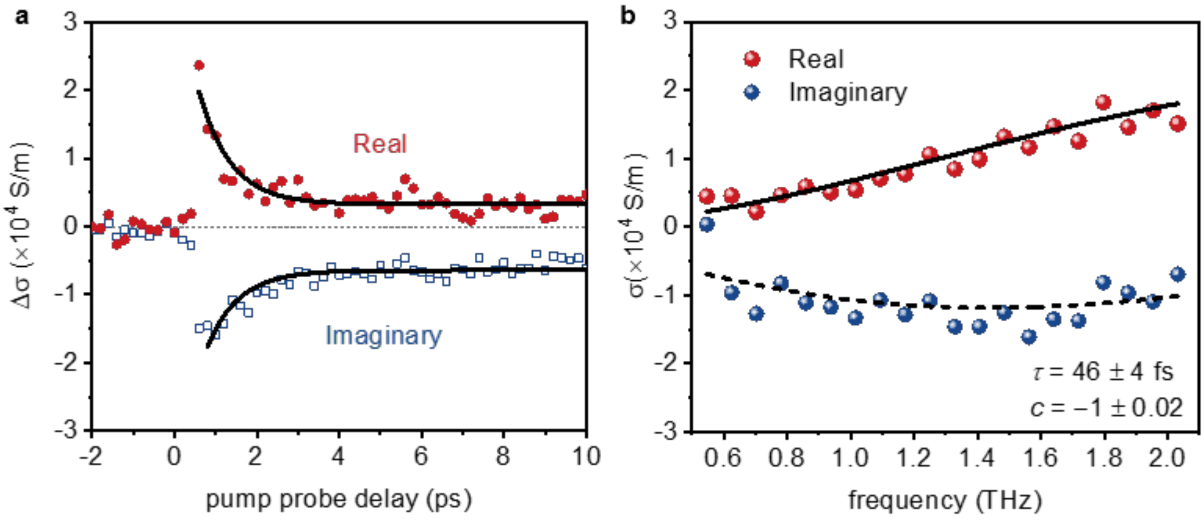

Supplementary Figure 24. a, Photoconductivity dynamics measured for a thin film of **PGNRb** at the main peak of THz electric field as a function of pump-probe delay time. The solid thick lines represent simulated photoconductivity dynamics of free-carriers with lifetime of ~2 ps. **b**, Frequency-resolved THz conductivity of **PGNRb** measured at $t_p = 2$ ps and a Drude–Smith fit.

Free-carrier generation quantum yield

Based on the Drude-Smith fitting, the plasma frequency can be extracted. The free carrier density n then can be estimated to be 2.24 $\times 10^{12}$ cm^{-3} following: $\omega_p = \sqrt{\frac{e^2 \cdot n}{\varepsilon_0 \cdot m^*}}$, where e is the electron charge, ε_0 is the vacuum permittivity and m^* is the effective mass. The absorbed photon density N_{abs} is estimated through the incident photon density N and the absorption A (in %) of **PGNRb** in solution following: $N_{abs} = N_{in} * A$. The free-carrier generation quantum yield η is then defined by $\eta = n/N_{abs}$, which yields 0.1%. The order of magnitude of the obtained value is in line with that reported for semiconducting polymers[21] (in the range of 10^{-5}–10^{-3}).

12. Single-Molecule Devices

12.1 Experimental set-up and device fabrication

Devices applied for transport measurements were fabricated using the following procedure: photolithography was used to pattern arrays of gate electrodes (Ti/Pd 5/25 nm) on a Si wafer with 300 nm of SiO_2. A 10 nm thick layer of HfO_2 was deposited using atomic layer deposition onto the gate to serve as a gate dielectric. A second photolithography step was used to pattern source and drain electrodes (Ti/Au 5/65 nm). This lithography step was aligned with the already patterned gate electrodes so that the gate electrode sits between source and drain. Chemical vapor deposited (CVD) graphene was transferred onto the entire wafer by Graphenea.

After graphene transfer was complete, the wafer was protected by spin coating a layer of poly(methyl methacrylate) (PMMA) resist before dicing into 1 × 1 cm chips. Each chip contains 874 individual devices. The protective PMMA layer was removed overnight in warm acetone and the graphene layer was patterned into bow-tie shapes using electron-beam lithography with AR-N 7500 negative resist. Bow-ties were patterned using a dose of 340 μC cm^{-2} and beam current of 100 pA and were developed for 60 seconds in MF-CD-26. Unwanted graphene was etched in an O_2 plasma (Henniker Plasma HPT-100 for 15 minutes, 25 sccm and 50 % power) and afterwards the remaining resist was removed by immersing the chip in 1165 Remover for 5 minutes followed by overnight in acetone. Finally, the chips were washed in isopropanol (IPA) and blown dry with nitrogen.

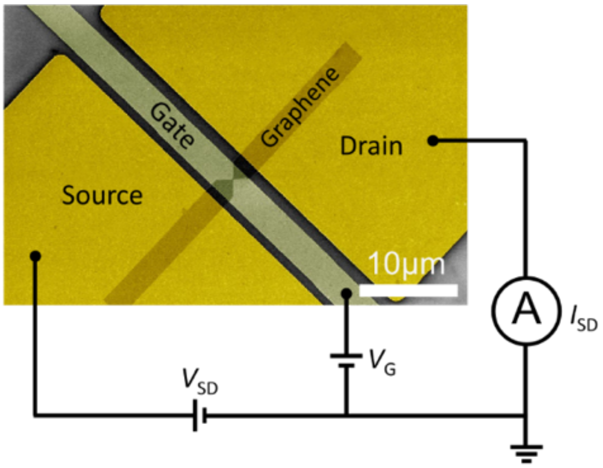

Supplementary Figure 25. False-color scanning electron microscopy (SEM) image of a single device, showing the electrodes layout and circuit connections. Current is then converted by a current-to-voltage converter and read back by a digital-to-analog converter.

Feedback controlled electroburning was used to produce nanogaps, localized at the bow-tie constriction, with widths of 3–7 nm[22, 23]. Several cycles of the electroburning protocol were performed, up to three different gate voltages (V_G) for each device, and each time scanning the gate voltage at fixed bias (V_{SD}) while monitoring I_{SD} to check for residual graphene quantum dots. If any are found, the electroburning process is restarted at that gate voltage to clear the residual graphene. For all devices, the resistance limit was set at 2.3 GΩ. I_{SD}-V_{SD}

characteristics were recorded for all devices immediately after nanogap formation and after drop-casting a ~8 μL suspension of the **PGNRb**. Suspensions were prepared by sonicating a dry powder of the **PGNRb** in toluene (0.02 mg mL^{-1}) for 60 min. Devices which show an increase in current after deposition of the PGNR were selected for wire bonding and further measurements. Transport measurements were performed in an Oxford Instruments Triton 200 dilution refrigerator using low noise DC electronics. All low-temperature measurements were performed at a temperature of 25 mK. Differential conductance is retrieved by numerical differentiation of the measured current.

12.2 Results from low temperature measurements

In order to shed light onto device performance, we measured the conductivity at mK temperatures of several devices. Transport spectroscopy at mK temperatures offers a unique perspective onto individual charge states, which are paramount to understand limiting factors of device performance from a fundamental point of view. In what follows, we provide an overview of a few typical **PGNRb** devices that were measured across several cooldowns.

Device 1

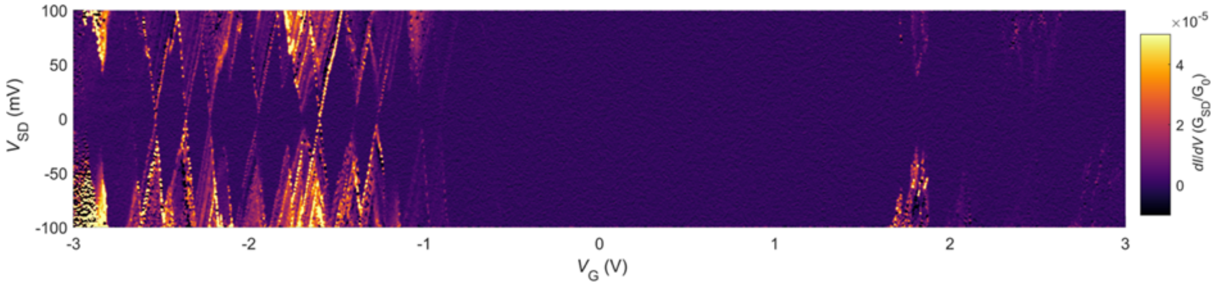

Supplementary Figure 26. Stability diagram for device 1 measured at 0.025 K. The map shows three regions. Negative voltages have discernible conductance peaks, while positive voltages have a gap region ΔV_G ~ 2.7 V and faint resonances.

The full charge stability diagram of device 1 is shown in Supplementary Figure 26. Yellow colors indicate regions of higher differential conductance while darker colors indicate regions of lower differential conductance. At gate voltages below –1.0 V clear signs of Coulomb blockade diamonds are present. A large gap exists between $V_G = -0.9$ V and $V_G = +1.8$ V.

Above $V_G = +1.8\ V$, the conductance increases, although features are poorly defined, with the level of current close to the noise limit of the current to voltage converter used in our transport setup. In the absence of a discernible repeating pattern, it is possible that the faint diamonds visible at higher gate voltages are caused by spurious charges injected through the gate oxide or via surface states.

From Supplementary Figure 27 we can read the addition energies for the different charge states, from the height of each diamond, within the assumption that only one electron is exchanged for each charge transition. Furthermore, two peaks at V_G ~2.5 mV and V_G ~1.5 mV are closed, so we can retrieve the lifetime broadening from a low bias (V_{SD} ~2.6 mV) gate trace and fitting it with a Lorentzian function. Supplementary Table 3 reports the fitting parameters, which allows us to retrieve the tunnel coupling with the leads, hence the lifetime broadening of ground state of two different charge states.

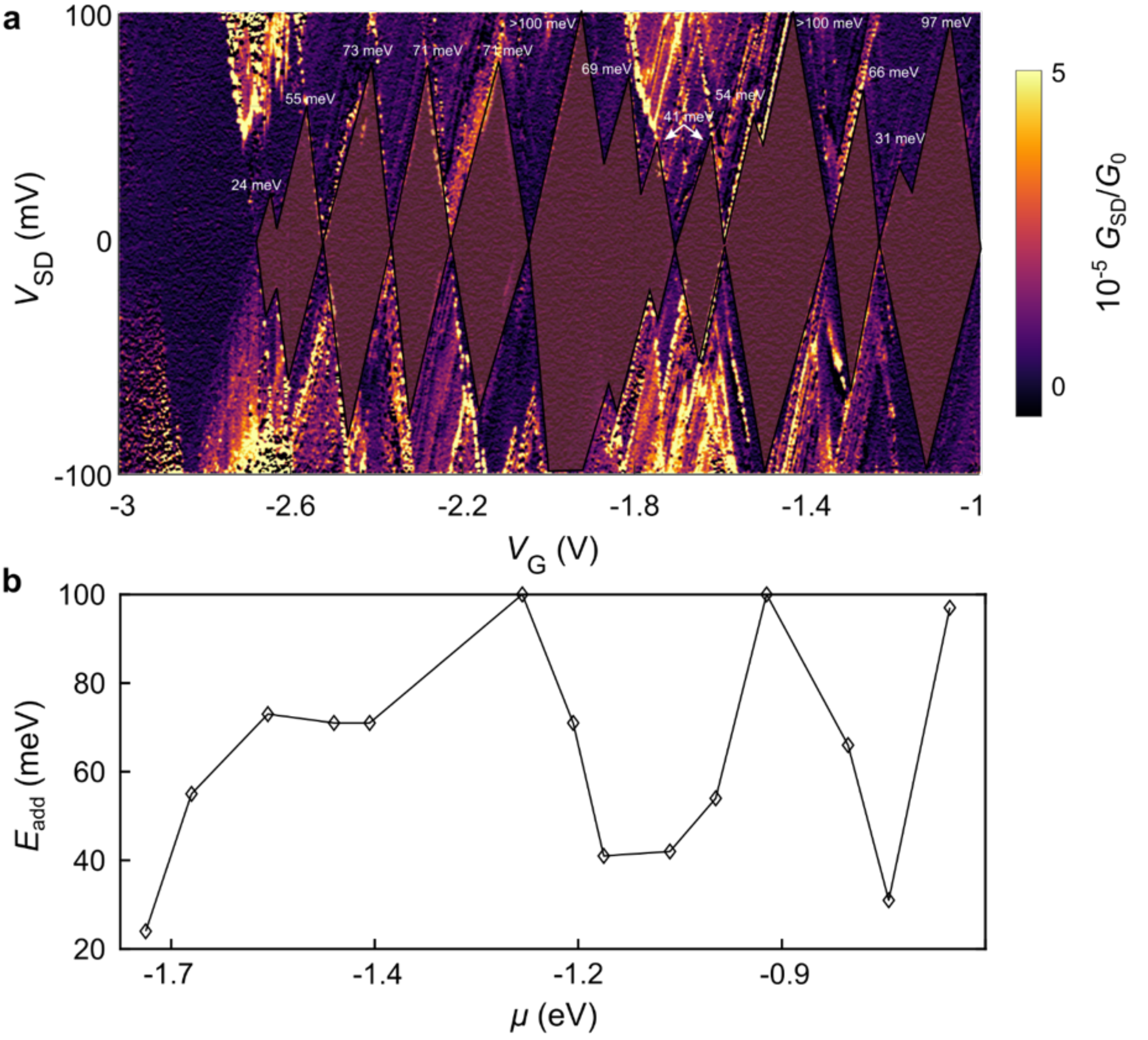

Supplementary Figure 27. a, Low-temperature stability map demonstrating Coulomb blockade and well-defined charge states. **b**, We extract the addition energies from the diamond height and plot them as a function of the chemical potential felt by the ribbon as a result of the application of a gate voltage, assuming a constant gate coupling of $\boldsymbol{\alpha_G \sim 0.65}$ eV/V for all charge states.

Supplementary Table 3. Lorentzian fit parameters extracted by fitting a low bias gate trace. The two peaks are separated by 600 meV in voltage, which could be assigned to the single-electron charge transition *N*/*N*+1, where *N* is the number of electrons on the ribbons which is unknown, so that *N*/*N*+1 means the neutral / radical cation transition.

Peak Position (eV)	Lifetime Broadening (meV)	G_{max}/G_0
–1.63	2.6	4.5E–7
–1.04	2.8	2.8E–7

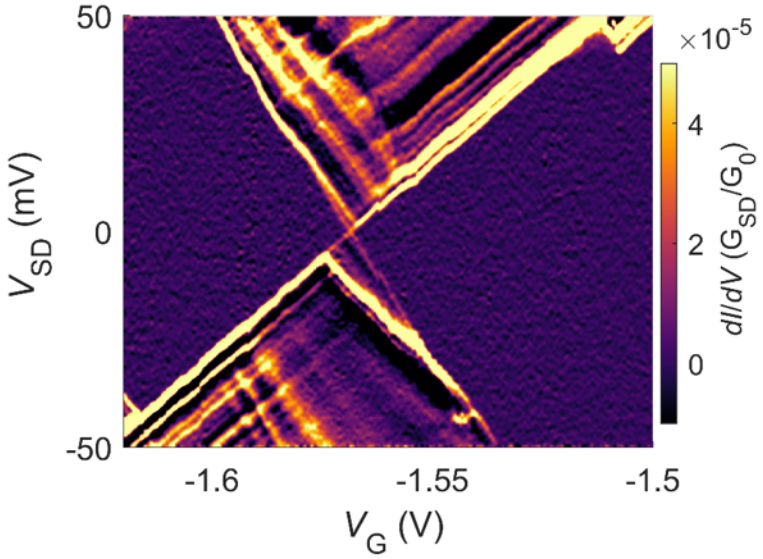

Supplementary Figure 28. Enlargement of a single diamond in the stability diagram for device 1. The well-defined structure of ribbon hybrid allows us to resolve several excited lines within a single diamond at low temperatures, which we attribute to vibrational and electronic excitations.

A higher resolution scan of a peak at $V_G = -1.57$ V demonstrates the possibility of obtaining very high-quality transport data with this combination of device architecture and molecular material. Visually inspecting Supplementary Figure 28, excited states visible as lines were observed, which run parallel to the diamond edge as well as regions of negative differential conductance. From diamonds as the one shown in Supplementary Figure 28, we can extract the gate coupling which measures the effectiveness of the gate voltage to change the chemical potential of the ribbon. We found $\bar{\alpha}_G \sim 0.65$ eV/V averaged across all the measurable charge states, with a slight asymmetric coupling with the drain lead for this particular device.

In the transport data, there are several approximately equally spaced states. By following a harmonic oscillator mode, we can tentatively attribute these states to vibrational modes of the **PGNRb** (Supplementary Table 3). In particular the 42 meV level corresponds to the 350 cm^{-1} energy of Ni-N A_{1g} out-of-plane vibration modes[24], while the B_{1g} Ni-N, in plane vibration mode is observed at 20 meV[25]. The remaining 35, 28, 10, and 8 meV modes are also all reported in the literature for Ni-porphyrins as carbon-carbon bending modes[25]. This shows excellent agreement between quantum transport signal and the structural features of the PGNR hybrids.

Supplementary Table 4. Assignment of mode frequencies.

number	energy level (meV)	frequency (cm^{-1})	assignment
1	42	339	A_{1g} ν(Ni-N)
2	35	282	A_{1g} δ(C-C)$_{sym}$
3	28	226	A_{2g} δ(C-C)$_{asym}$
4	20	161	B_{1g} δ(Ni-N)
5	10	81	B_{2g} δ(C-C)

Device 2

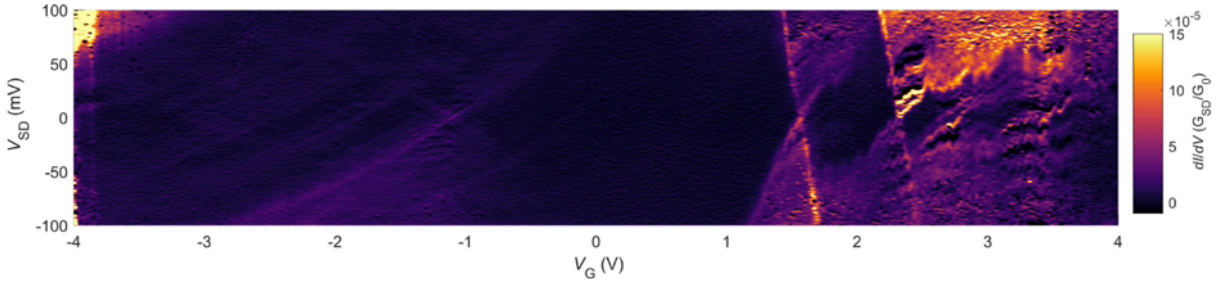

Supplementary Figure 29. Conductance map at 0.025 K for device 2.

Lines are visible within the conducting regions which run non-parallel to the diamond edges. These are probably graphene lead states which are due to an electrostatic variation in the density of states in the graphene leads[26]. Analysis of the diamond between the two clearest Coulomb peaks at a gate voltage $V_G \approx 2$ V gives values for $E_{add} = 93$ meV and $\alpha = 0.125$ eV/V.

Device 3

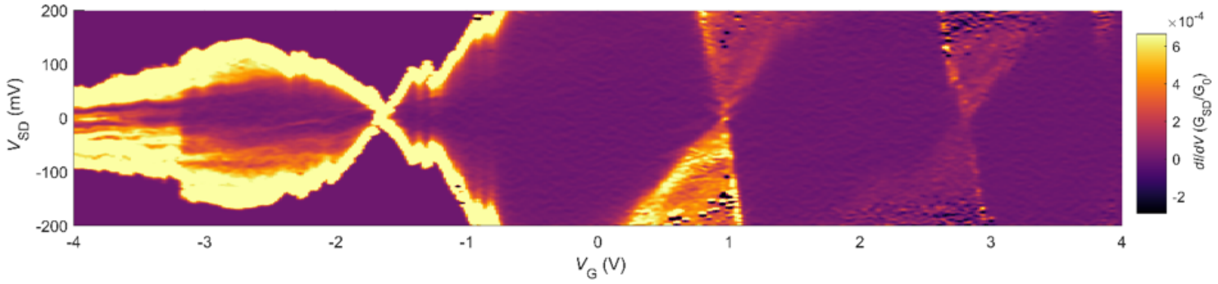

Supplementary Figure 30. Conductance map at 0.025 K for device 3.

Supplementary Figure 30 shows three Coulomb peaks at 0.025 K. Analysis of the diamond between the two clearest Coulomb peaks at a gate voltage $V_G \approx 2$ V gives values for $E_{add} = 390$ meV and $\alpha = 0.125$ eV/V.

Some signatures of cotunneling are visible especially around the charge degeneracy of the peak at $V_G = 0.97$ V. Current in the regions of the stability diagram at $V_G < -1$ V are not measured as the current exceeded the limit of the current to voltage converter and the ${}^{\mathrm{d}I}/_{\mathrm{d}V}$ appears flat in these regions.

Determination of PGNR length

We can estimate the length, L, of the **PGNR** by assuming it forms a rectangular quantum dot of width w = 1.12 nm using equation S1:

$$L = \frac{e^2\left[8d\arctan\left(\frac{w}{4d}\right)+w\log\left(1+\frac{16d^2}{w^2}\right)\right]}{4\pi\varepsilon_0\varepsilon_r E_{add}} \qquad \text{(S1)}$$

Where e is the electron charge, ε_0 is the vacuum permittivity and $\varepsilon_r$is the relative permittivity of the HfO_2 layer of thickness $d = 10$ nm between the gate electrode and **PGNRb**. Using the extracted values of devices 1, 2 and 3 we can estimate the lengths of 40 ± 3, 35 ± 3, and 10 ± 3 nm, respectively.

Determination of ε_r:

A parameter needed for the determination of the **PGNRb** length is the relative permittivity of the gate oxide ε_r. Due to the ultra-thin nature of the oxide used in these devices, it is not possible to rely on the bulk value for HfO_2 and it must be experimentally determined[27].

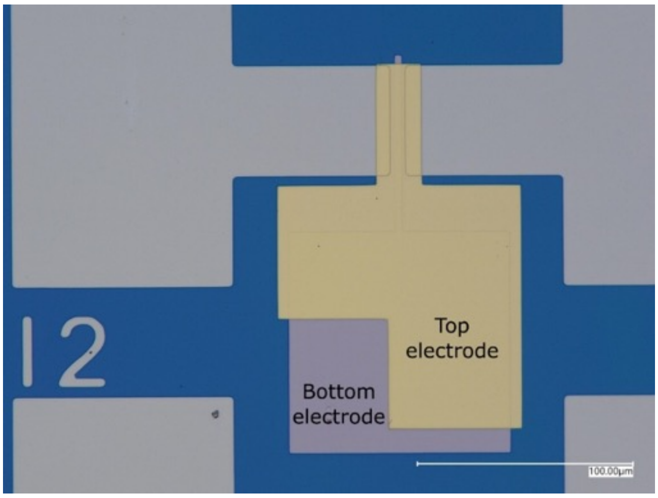

Supplementary Figure 31. Capacitors fabricated through electron-beam photolithography showing top and bottom electrodes.

This was achieved by microfabricating capacitors on the devices using the following approach. A chip was selected from the same wafer used to fabricate devices 1, 2 and 3. The substrate was O_2 plasma etched to remove the CVD graphene layer and prepare the surface for lithography (20 minutes, 25 sccm and 100 % power). An array of top electrodes was patterned using electron beam lithography, metal deposition and lift-off. The design consists of overlapping top electrode of varying areas with the gate contact pad separated by the ALD deposited HfO_2 dielectric layer as shown in Supplementary Figure 31. A small region was removed from the top electrode, allowing a W probe to make contact through the oxide and electrically contact the bottom electrode.

An Andeen-Hagerling 2550A ultra-precision capacitance bridge was used to measure the capacitance between the top electrode and the bottom gate electrode. The measurement was carried out at room temperature in a probe station. The stray capacitance of the probe station and experimental wiring was measured and found to be insignificant when compared to the values obtained from the microfabricated capacitors. The excitation voltage applied by the bridge was limited to 1.0 V RMS to reduce the risk of dielectric breakdown. ε_r was determined using Equation S2:

$$\varepsilon_r = \frac{Cd}{\varepsilon_0 A} \quad \text{(S1)}$$

Where the oxide thickness d = 10 nm and ε_0 is the permittivity of free space and A is overlapping areas of the top and bottom electrode measured using microscopy. The obtained values for ε_r are shown in Supplementary Table 5. An average value of ε_r = 15.4 was used in all length calculations.

Supplementary Table 5. Measured values of electrode area, capacitance and $\boldsymbol{\varepsilon_r}$ for several capacitors. These are consistent across various randomly selected points onto a single chip.

Device	Electrode area/ µm^2	Measured capacitance/ pF	ε_r
A1	7454.51	101.13	15.32
A2	4541.95	62.38	15.51
C3	2402.16	32.46	15.26
E1	7438.05	100.57	15.27
I1	7432.99	103.25	15.69

Negative differential conductance – values and applications

As shown in Supplementary Figure 32, regions exist where an increase in V_{SD} results in a reduction in the overall current flowing through the system. These features are referred to as negative differential conductance (NDC) and are of particular interest as they enable the possibility of creating new classes of molecular scale components. NDC components based on conventional semiconductor technologies already exist and include switching elements, nanoscale amplifiers[28], and oscillators[29] that can operate in the GHz and above. The use of systems exhibiting NDC may also simplify existing digital logic circuitry, reducing the number of components needed and give the potential for an increase integration densities and reduced power consumption[30].

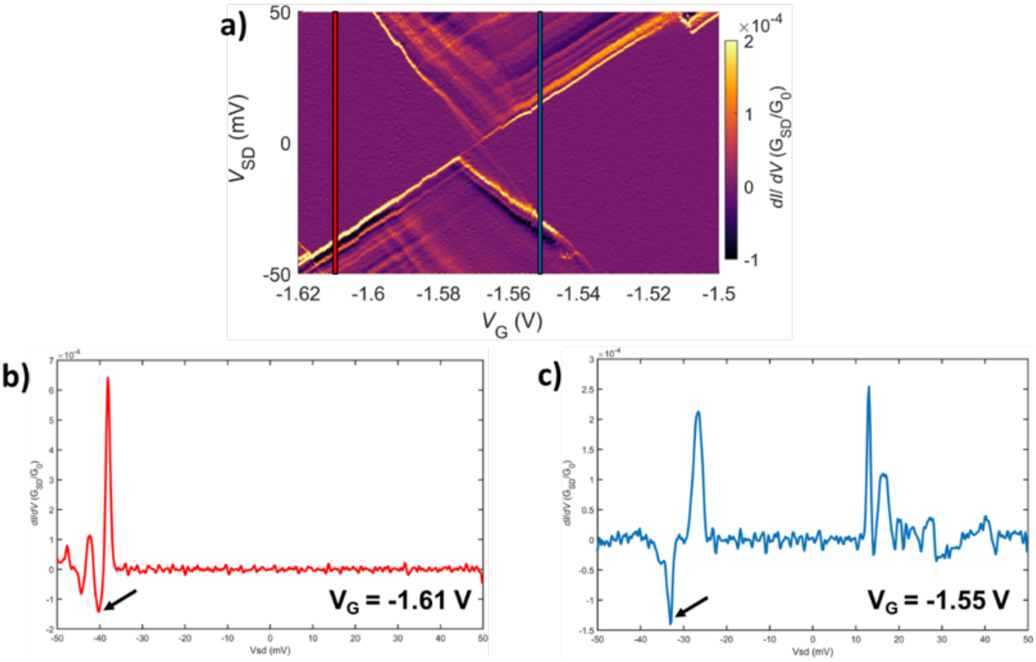

Supplementary Figure 32. **a**, Enlargement of a single diamond in the stability diagram for device 1. The well-defined structure of the ribbon allows us to resolve several excited lines within a single diamond at low temperatures, which we attribute to vibrational and electronic excitations. **b**, dI/dV along red line in **a**. **c**, dI/dV along blue line in **a**.

Regions of NDC with gradients similar to that of the diamond edges allows us to distinguish them as not being related to universal conductance fluctuations (UCFs) which arise as a result of the graphene electrodes[26]. The value of NDC in this region reaches a maximum value of -1.42×10^{-4} G/G$_0$. The origin of these NDC features are probably related to transport through multiple excited states which come into and out of the transport window as V_{SD} and V_G are swept. Another region of strong NDC appears in a region probably related to interference of the **PGNR** states with those of the electrode states. The value of NDC shares a similar value of -1.40×10^{-4} G/G$_0$.

12.3 Results from room temperature measurements

This section presents several devices with field-effect transistor characteristics. All measurements were taken in ambient conditions with a semi-automated, 3-probe system Cascade Microtech Summit 12000. CuBe probes with tip diameters of 30 μm were used to contact the source and drain Au pads, while W probes with tip diameters of 10 μm were used for the gate probe. Supplementary Figure 33 shows an example of the device characteristics. The device exhibits p-type of doping and the ON-state channel conductance G is 0.06 S mm^{-1} when normalized by the width of the **PGNRb** (w_{PGNR} = 1.12 nm), and the ON-state conductivity $\sigma = G * \frac{L}{w_{\mathrm{GNR}}} \sim 2.5$ μS, for a channel length $L \sim 40$ nm (the length is obtained by averaging the addition energies on the same device at low temperature). The device was switched off when the gate voltage V_{G} was larger than 0.5 V and had good $\frac{I_{\mathrm{ON}}}{I_{\mathrm{OFF}}} \sim 10^3$ as the source-drain voltage V_{SD} was equal to 0.1 V. The OFF-state conductance was calculated to be $G_{\mathrm{OFF}} \sim 6$ pS nm^{-1} and increasing as the V_{SD} is increased, despite exhibiting consistent switching behavior. The performance of these field-effect transistors can be quantified by the subthreshold swing SS, which is the inverse of the subthreshold slope from a plot of logI_{SD} *vs.* V_G, i.e. SS = dV_G/d(logI_{SD}) measured at constant bias V_{SD}. These devices give SS ~ 400 mV/dec, indicating good gate control over the conduction channel. On top of that, almost no hysteresis was measured under ambient condition (see Supplementary Figure 33, device **e5**). The hydrophobic nature of the sandwich established by the **PGNRb** and graphene leads considerably minimizes the undesired hysteresis in the transfer characteristics of the transistors, which can be caused by the presence of water close to the **PGNRb**/dielectric contact (gate voltage sweep rate was 44 mV s^{-1}), and outperforming early carbon nanotube FET[31].

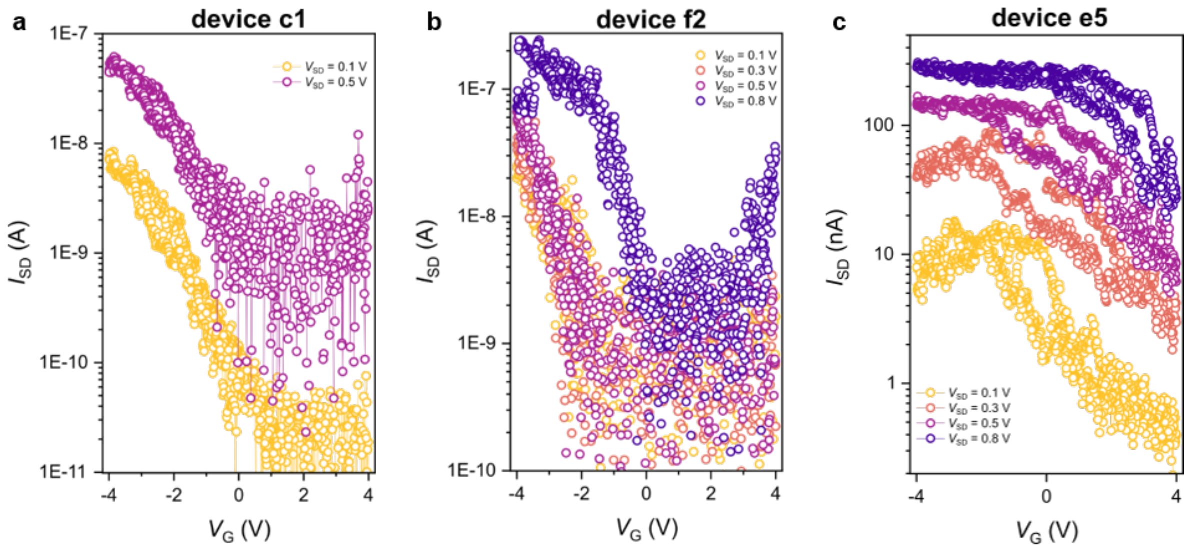

Supplementary Figure 33. Transfer characteristics of three **PGNR** FET devices at room temperature, showing some hysteresis behavior in device **e5**.

While the ON-state current is not directly related to the bandgap energy E_g, the OFF-state current strongly depends on E_g[32]. Assuming that **PGNRb** is an intrinsic semiconductor and its band structure has a mid-gap alignment with the source/drain contacts, the smallest (OFF-state) current will occur when both the conduction and valence bands are flat. If transport is controlled by thermal carrier emission the ON/OFF ratio will depend exponentially on the temperature. It is then possible to estimate the bandgap for this particular device to be:

$$2k_B T * \log\left(\frac{I_{\mathrm{ON}}}{I_{\mathrm{OFF}}}\right) = E_{\mathrm{g}} \approx 327 \text{ meV} \quad \text{(S2)}$$

Since it is typically difficult to establish the threshold voltage in a transfer characteristics without ambiguity, conventional FETs are frequently compared using the so-called field-effect mobility. This is device-specific rather than material-specific, and includes effects such as contact resistances and surface effects[33]. Two are the main types of FET mobilities, namely linear and saturation, and we have estimated both for several devices.

The conduction channel is pinched off when $V_{\mathrm{SD}} = V_{\mathrm{G}} - V_{\mathrm{Th}}$, where V_{Th} is the threshold voltage at which the device switches on. In this condition, the current cannot increase substantially anymore and saturates to a value $I_{\mathrm{SD,sat}}$, identifiable as a plateau by plotting the semi-logarithmic plot of either a transfer characteristics or by simply visualizing the output characteristics. If channel shortening effects caused by the depletion region at the drain are neglected, the saturation current can be obtained by substituting V_{SD} with $V_{\mathrm{G}} - V_{\mathrm{Th}}$, and within the gradual channel approximation the drain saturation current may be written as:[34]

$$I_{\mathrm{SD,sat}} = \frac{W}{2L} \mu_{\mathrm{sat}} C_i (V_{\mathrm{G}} - V_{\mathrm{Th}})^2 \quad \text{(S3)}$$

where C_i is the gate capacitance per unit area. From the capacitance measurements detailed above, we extract a $C_i = 1.4 \times 10^{-2}$ $\mathrm{Fm^{-2}}$. We estimate a saturation mobility (also called field-effect mobility) for this particular device to be 0.8 $\mathrm{cm^2\ V^{-1}\ s^{-1}}$. The field-effect mobility is device-specific, not material-specific, and includes effects such as contact resistances and surface effects[33]. We further estimate the field-effect mobility in the linear regime ($V_{\mathrm{G}} \gg V_{\mathrm{SD}}$) by extracting the gradient of I_{SD} versus V_{G} at constant $V_{\mathrm{SD}} = 0.1$ V:

$$\mu_{\mathrm{lin}} = \frac{\partial I_{\mathrm{SD}}}{\partial V_{\mathrm{G}}} \frac{L}{W C_i V_{\mathrm{SD}}} = \frac{g_m L^2}{C_{\mathrm{GS}} V_{\mathrm{SD}}} \quad \text{(S4)}$$

We remark that the capacitance values C_i and C_{GS} differ for Equation S5. In the rhs of Equation S5, C_{GS} represents the capacitance between the nanoribbon segment that spans across the gate and the gate itself. Therefore, we have estimated that the capacitance per unit area for a ribbon of $L \sim 40$ nm and $w_{\mathrm{GNR}} = 1.12$ nm placed on top of a gate of thickness $d \sim 10$ nm is:

$$C_{\mathrm{GS}} = \frac{\varepsilon_0 \varepsilon_r A}{d} = \frac{8.85*10^{-12}*15.47*(40*10^{-9}*1.2*10^{-9})}{10*10^{-9}} = 0.7 \text{ aF} \quad \text{(S5)}$$

We estimate a linear field-effect mobility of about 4 $cm^2\ V^{-1}\ s^{-1}$ for this particular device.

Supplementary Figure 33b demonstrates a similar device (**f2**) to Figure 27a (**c1**), with good reproducibility of qualitative and semi-quantitative features. In comparison with device **c1**, this one exhibits $\frac{I_{\mathrm{ON}}}{I_{\mathrm{OFF}}} \sim 10^3$, which corresponds to $E_g \sim 220$ meV. The peculiarity of this device is that a current saturation limit is reached only at bias close to 0.8 V, with a $\Delta V_{\mathrm{gap}} \sim 3$ V, and the OFF-state current tends to increase by increasing the bias. The gate has good control (*SS* ~ 400 mV/dec) over the conductive channel at small bias, and excellent control (*SS* ~ 160 mV/dec) at 0.8 V, amongst the highest value measured across our devices. Furthermore, the ON-state channel conductance *G* normalized by the channel width reaches values as high as 0.2 S mm^{-1} with ON-state conductivity as high as 8 μS. We report slightly higher values for the mobility of this device as we extracted $\mu_{\mathrm{sat}} = 3.5$ $cm^2\ V^{-1}\ s^{-1}$ and $\mu_{\mathrm{lin}} = 16$ $cm^2\ V^{-1}\ s^{-1}$.

Supplementary Table 6. Linear and saturation mobilities extracted from the transfer characteristics of several devices.

Device	μ_{lin} ($cm^2\ V^{-1}s^{-1}$)	μ_{sat} ($cm^2\ V^{-1}s^{-1}$)
E5	5	1
C1	4	0.8
F2	16	3.5
C6	39	4
C4	11	4.3

In Supplementary Table 6, we present the linear and saturation mobilities for a variety of devices. Since **PGNRb** has molecularly defined edges, we explain these values by assuming that the effective mobility decreases as the ribbon width approaches sub-5 nm. The linear mobility values correlate well with those expected by theoretical estimation[35], however the saturation mobility values have not yet been found. At room temperature, the effective mobilities are predicted to be limited by acoustic phonons. Nonetheless, if the surface impurity density is large, this may become the major scattering mechanism, and values in the range of 10–1,000 are expected[36], which is consistent with the values we measured.

13. NMR and MALDI-TOF Mass Spectra

13.1 NMR Spectra

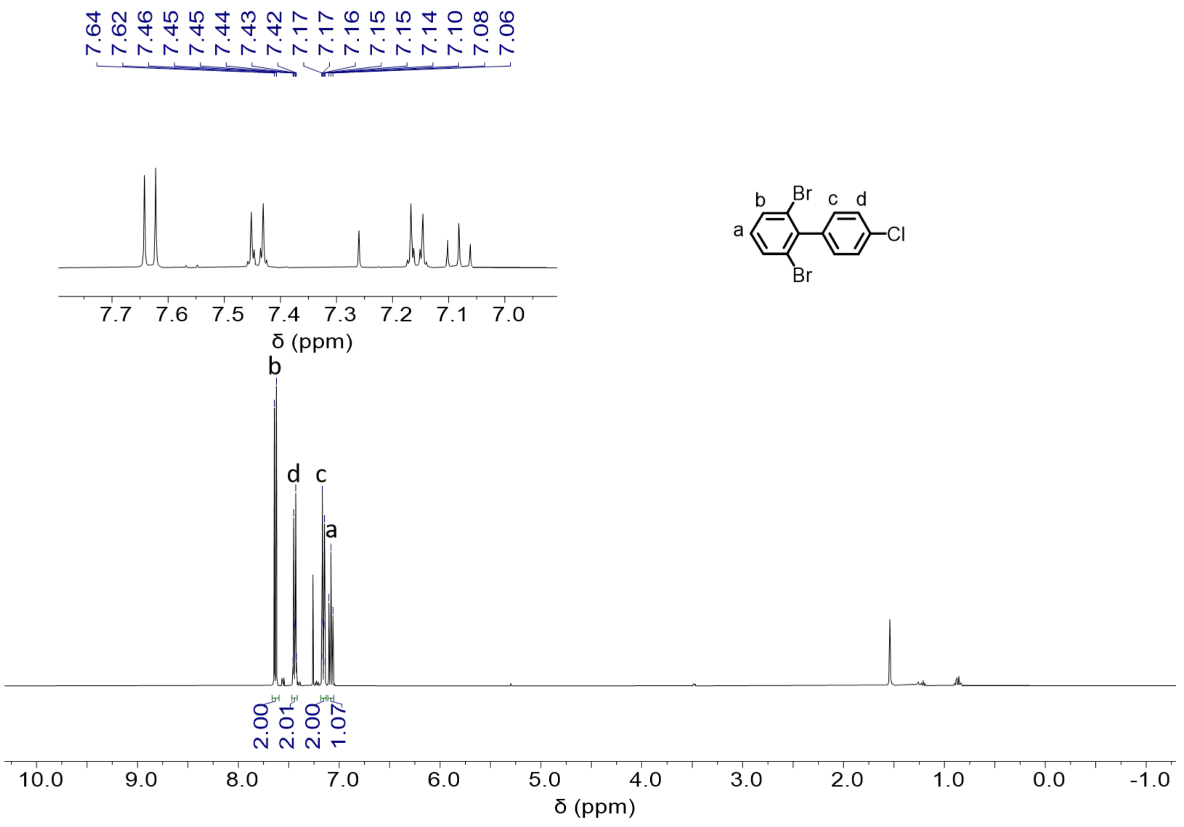

Supplementary Figure 34. ^{1}H NMR spectrum of 2,6-dibromo-4'-chloro-1,1'-biphenyl (**4**) (400 MHz, $CDCl_3$, 298 K).

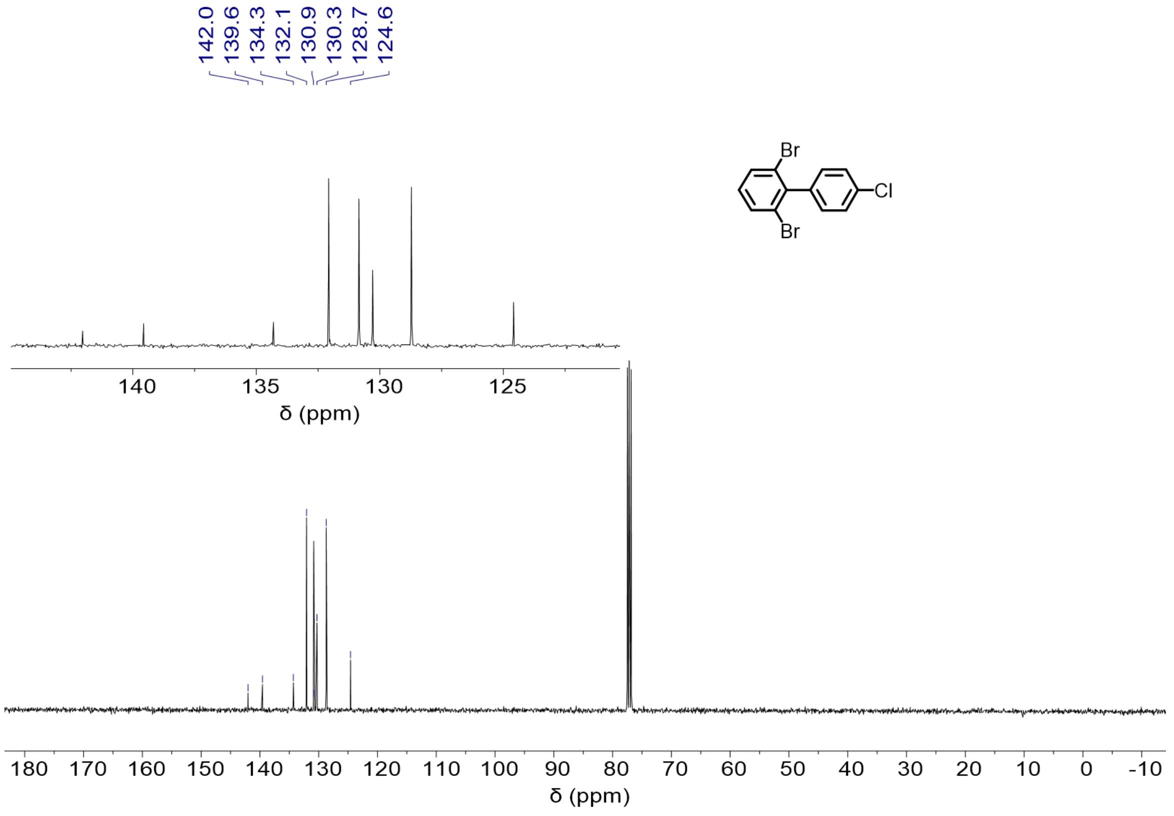

Supplementary Figure 35. ^{13}C NMR spectrum of 2,6-dibromo-4'-chloro-1,1'-biphenyl (**4**) (100 MHz, $CDCl_3$, 298 K).

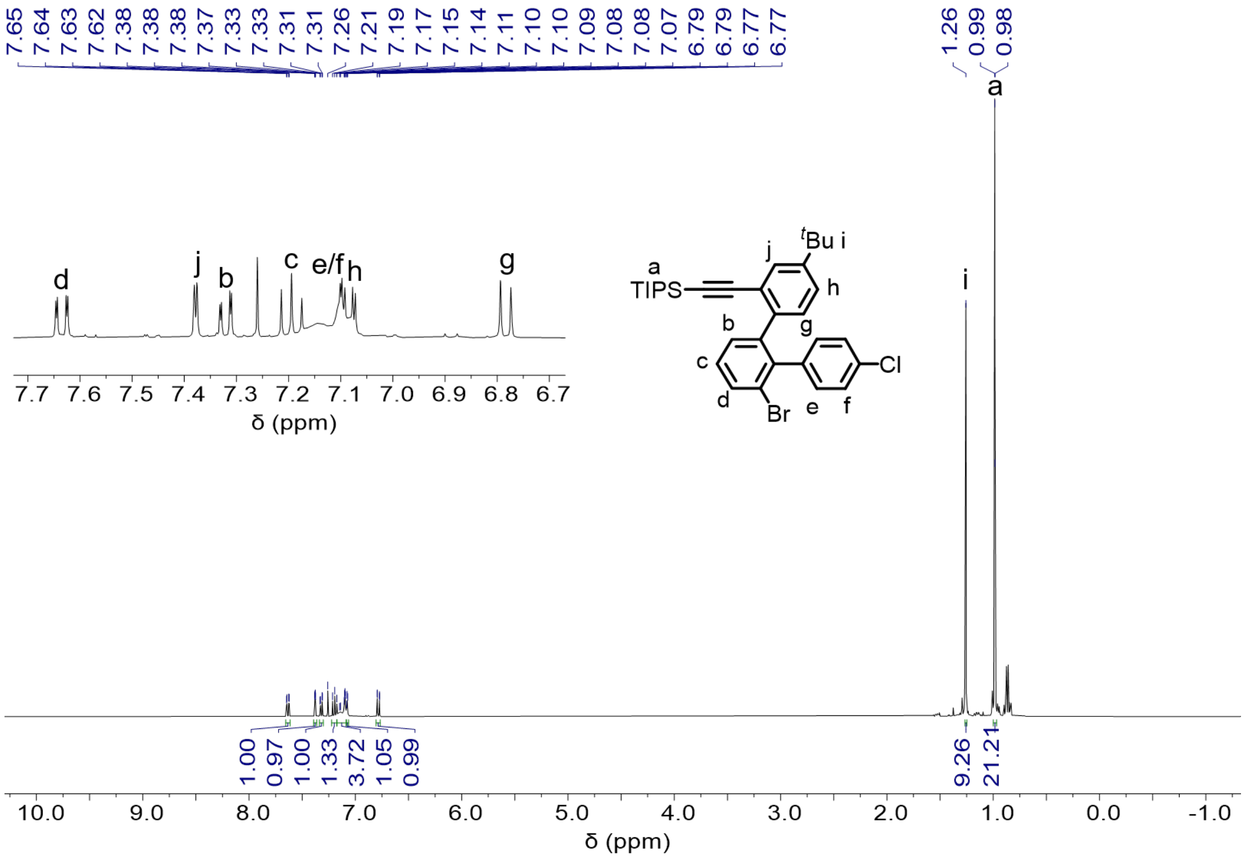

Supplementary Figure 36. ^{1}H NMR spectrum of ((3'-bromo-4-(*tert*-butyl)-4''-chloro-[1,1':2',1''-terphenyl]-2-yl)ethynyl)triisopropylsilane (**6**) (400 MHz, $CDCl_3$, 298 K).

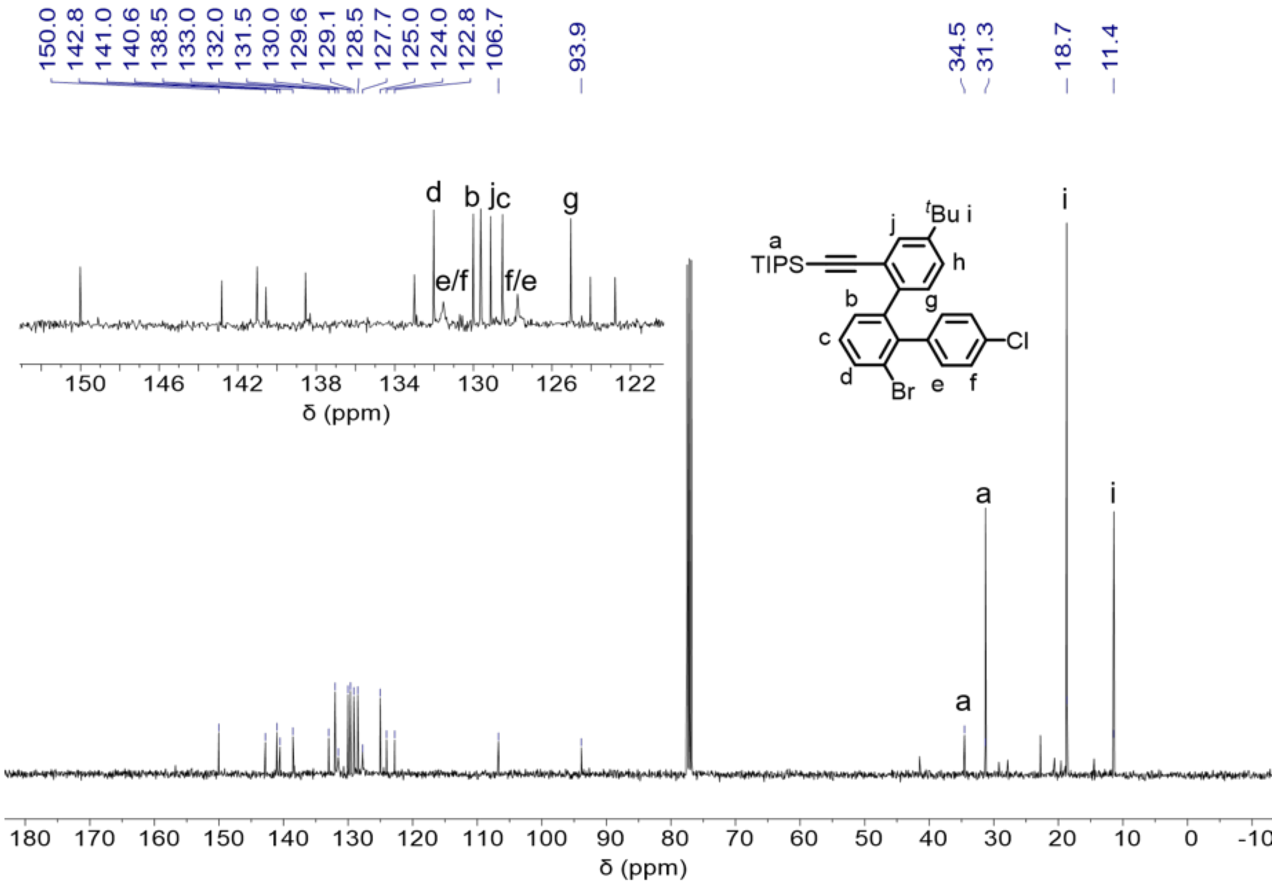

Supplementary Figure 37. ^{13}C NMR spectrum of ((3'-bromo-4-(*tert*-butyl)-4''-chloro-[1,1':2',1''-terphenyl]-2-yl)ethynyl)triisopropylsilane (**6**) (100 MHz, $CDCl_3$, 298 K).

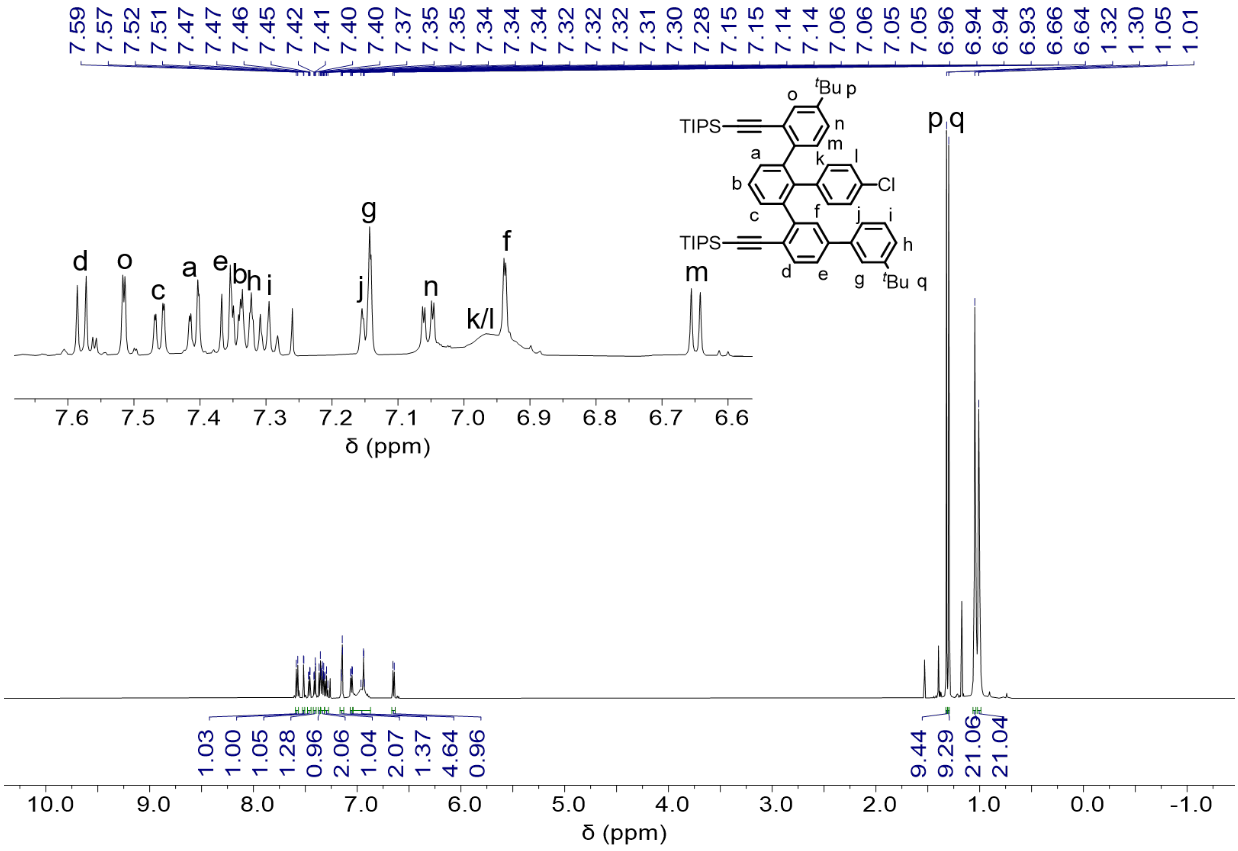

Supplementary Figure 38. ^{1}H NMR spectrum of ((3''',4-di-*tert*-butyl-2'-(4-chlorophenyl)-[1,1':3',1'':3'',1'''-quaterphenyl]-2,6''-diyl)bis(ethyne-2,1-diyl))bis(triisopropylsilane) (**8**) (600 MHz, $CDCl_3$, 298 K).

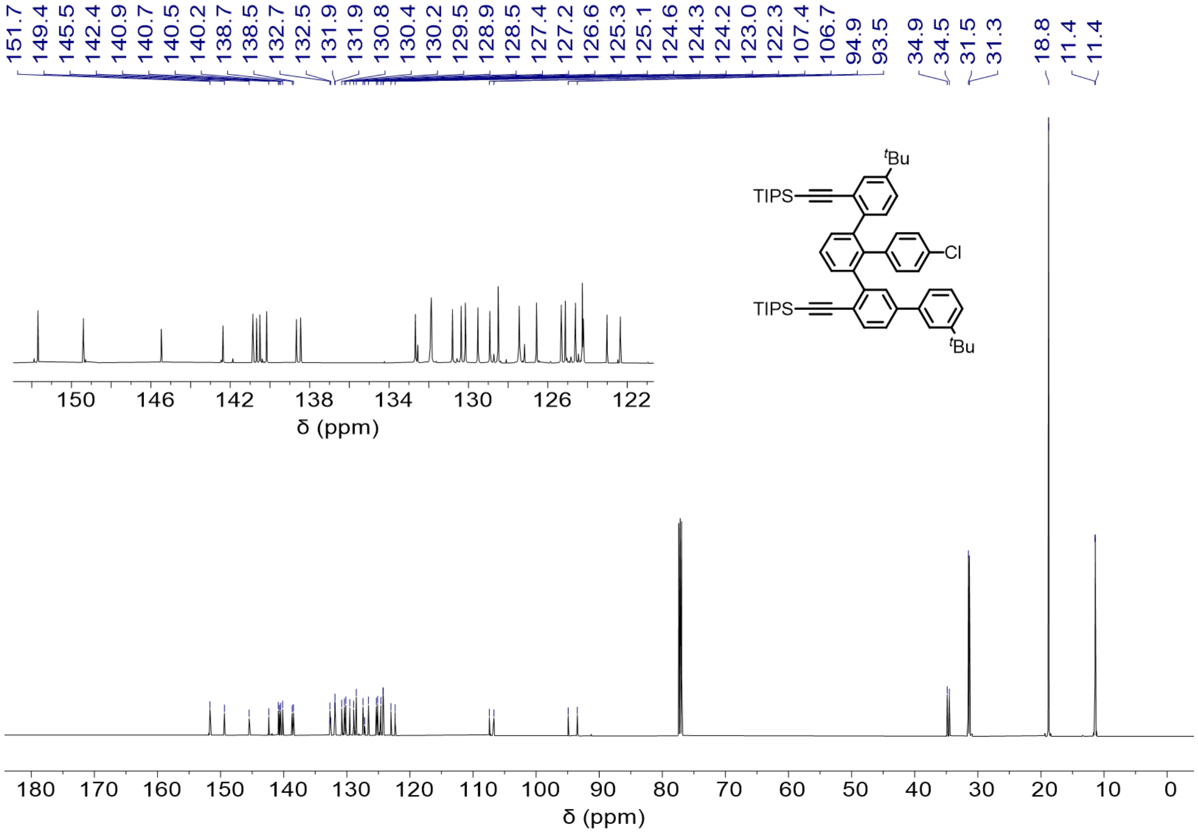

Supplementary Figure 39. ^{13}C NMR spectrum of ((3''',4-di-*tert*-butyl-2'-(4-chlorophenyl)-[1,1':3',1'':3'',1'''-quaterphenyl]-2,6''-diyl)bis(ethyne-2,1-diyl))bis(triisopropylsilane) (**8**) (150 MHz, $CDCl_3$, 298 K).

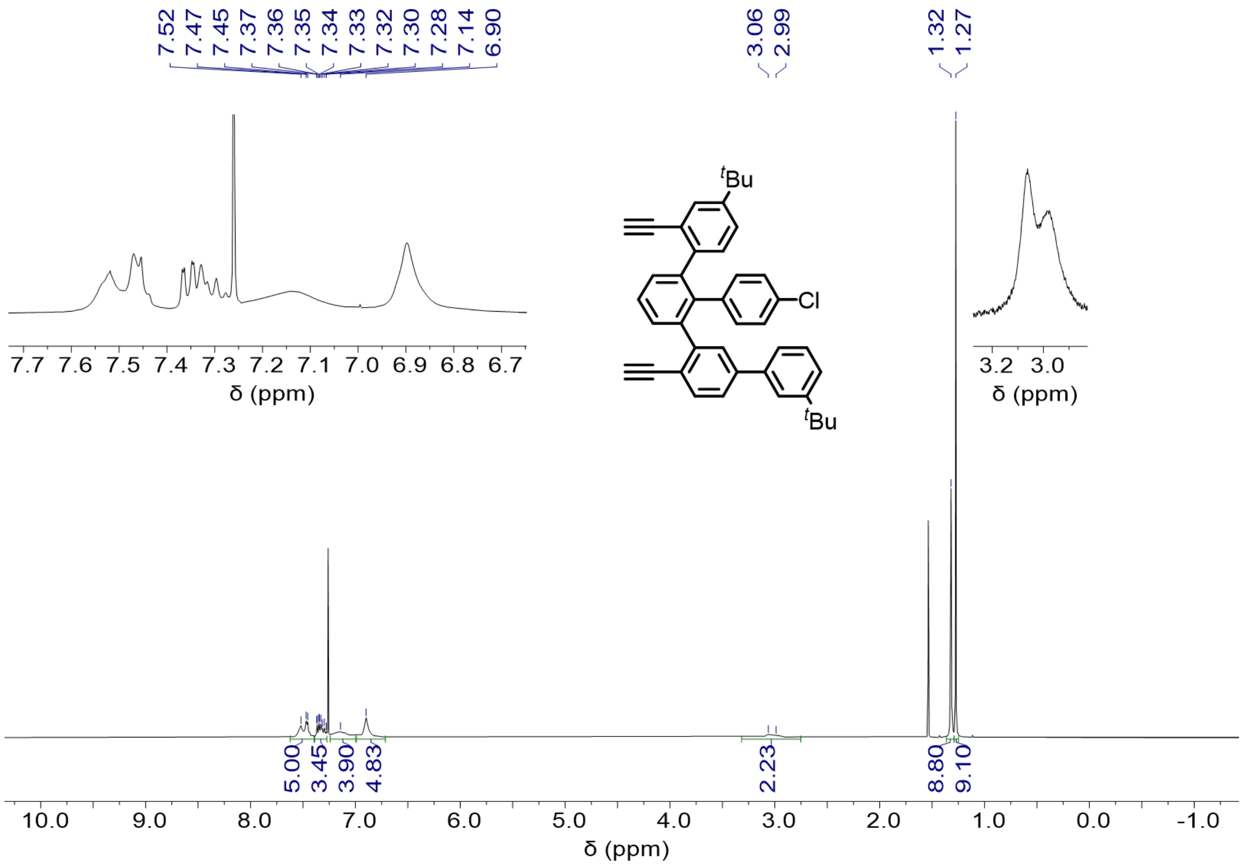

Supplementary Figure 40. ^{1}H NMR spectrum of 3'''-(*tert*-butyl)-6'-(4-(*tert*-butyl)-2-ethynylphenyl)-4-chloro-6''-ethynyl-1,1':2',1'':3'',1'''-quaterphenyl (**9**) (400 MHz, $CDCl_3$, 298 K).

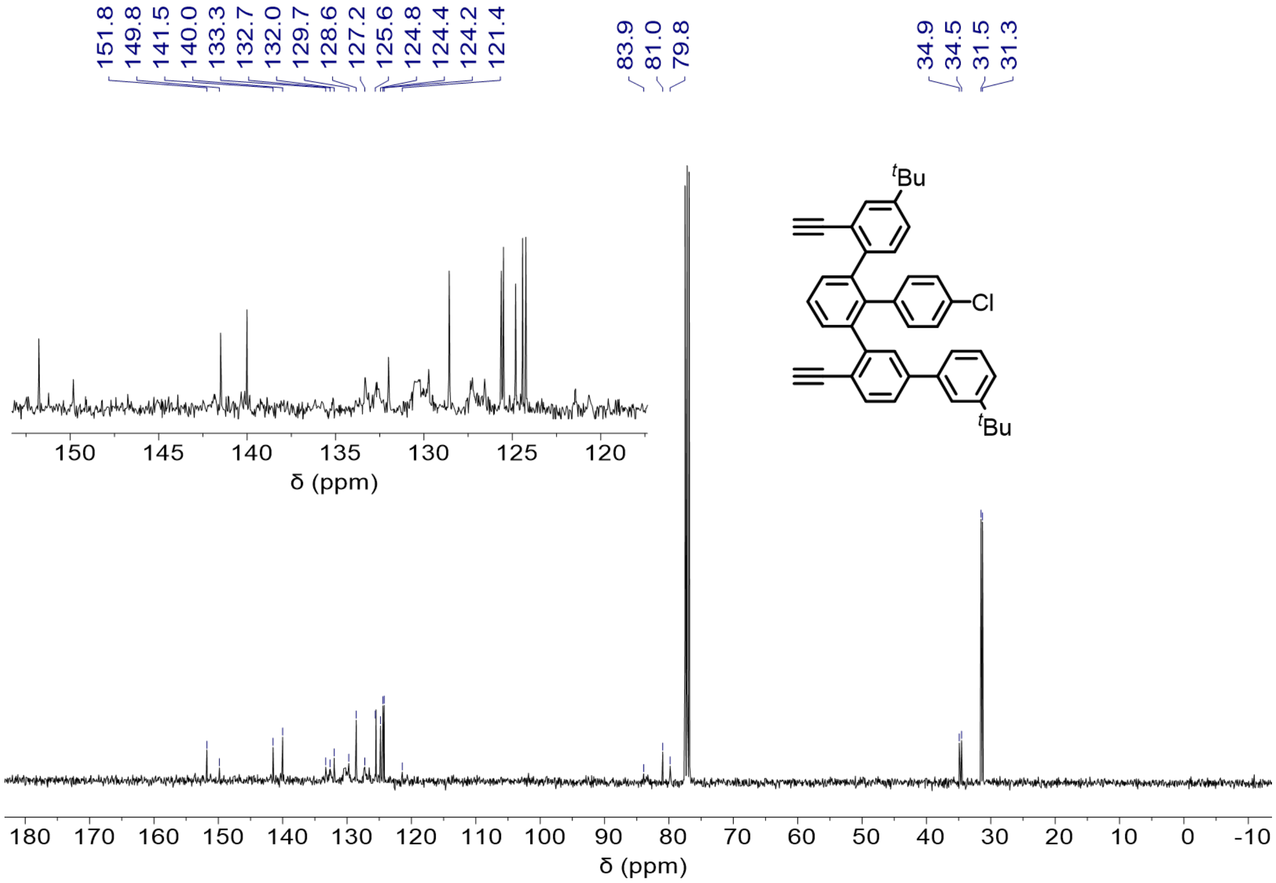

Supplementary Figure 41. ^{13}C NMR spectrum of 3'''-(*tert*-butyl)-6'-(4-(*tert*-butyl)-2-ethynylphenyl)-4-chloro-6''-ethynyl-1,1':2',1'':3'',1'''-quaterphenyl (**9**) (100 MHz, $CDCl_3$, 298 K).

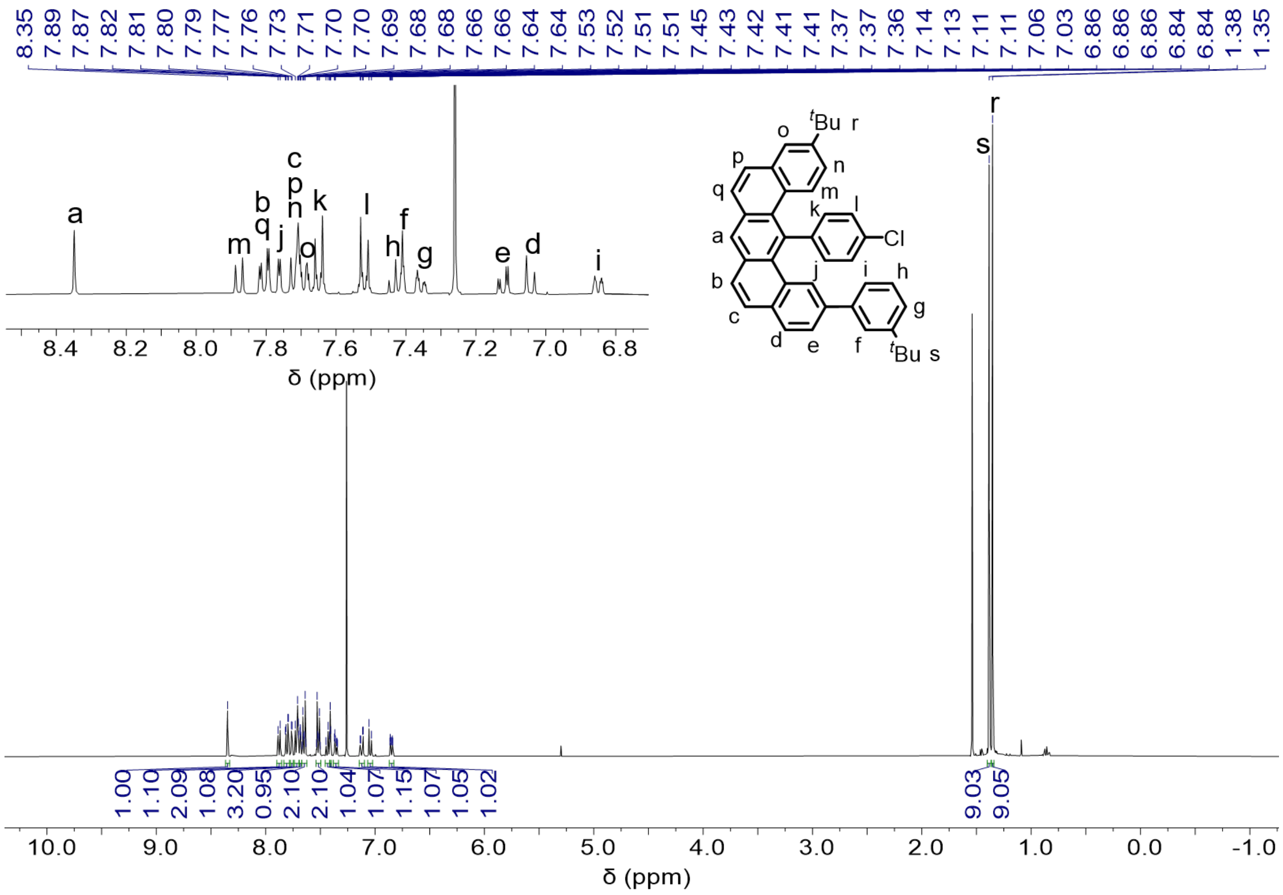

Supplementary Figure 42. ^{1}H NMR spectrum of dibenzo[*m*]tetraphene (**10**) (400 MHz, $CDCl_3$, 298 K).

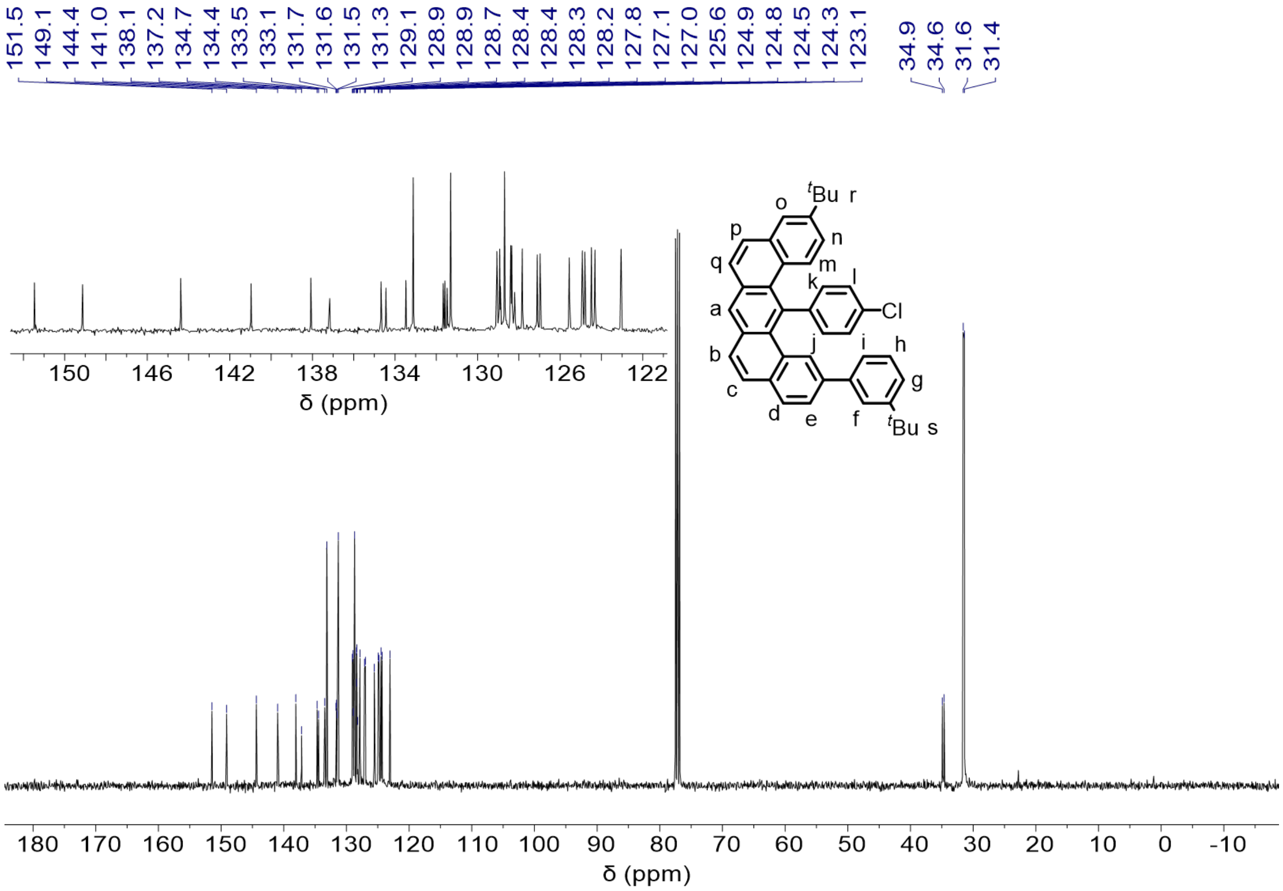

Supplementary Figure 43. ^{13}C NMR spectrum of dibenzo[*m*]tetraphene (**10**) (100 MHz, $CDCl_3$, 298 K).

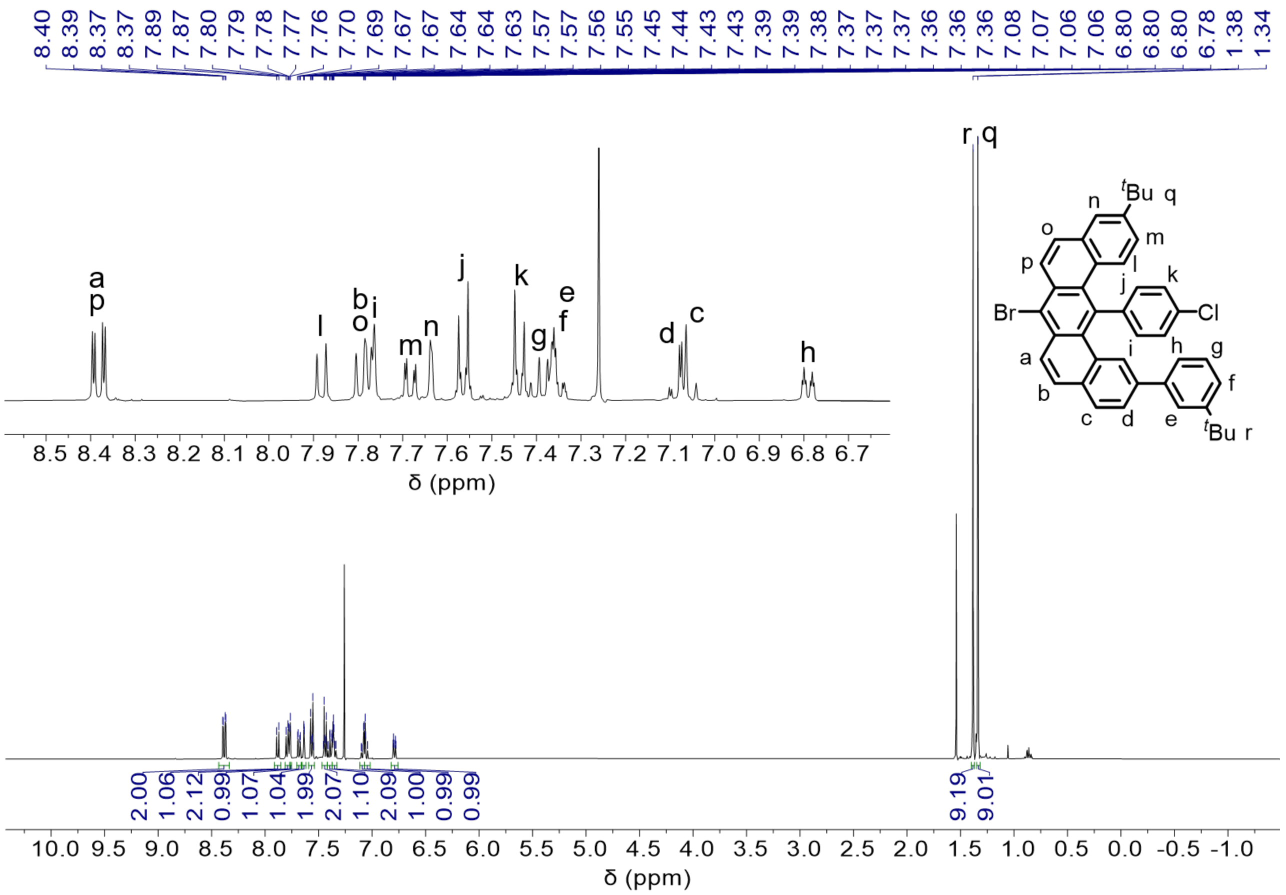

Supplementary Figure 44. ^{1}H NMR spectrum of bromodibenzo[*m*]tetraphene (**11**) (400 MHz, $CDCl_3$, 298 K).

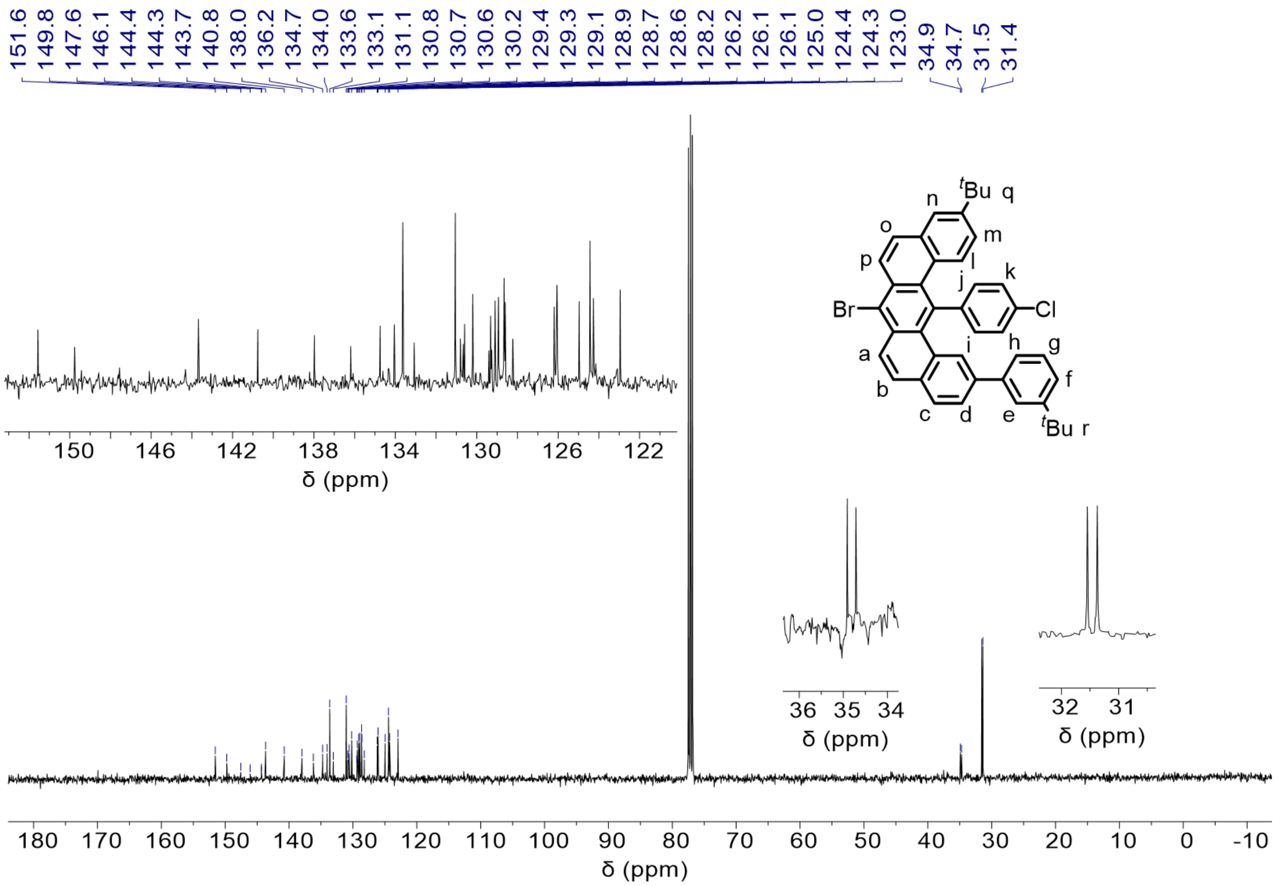

Supplementary Figure 45. ^{13}C NMR spectrum of bromodibenzo[*m*]tetraphene (**11**) (100 MHz, $CDCl_3$, 298 K).

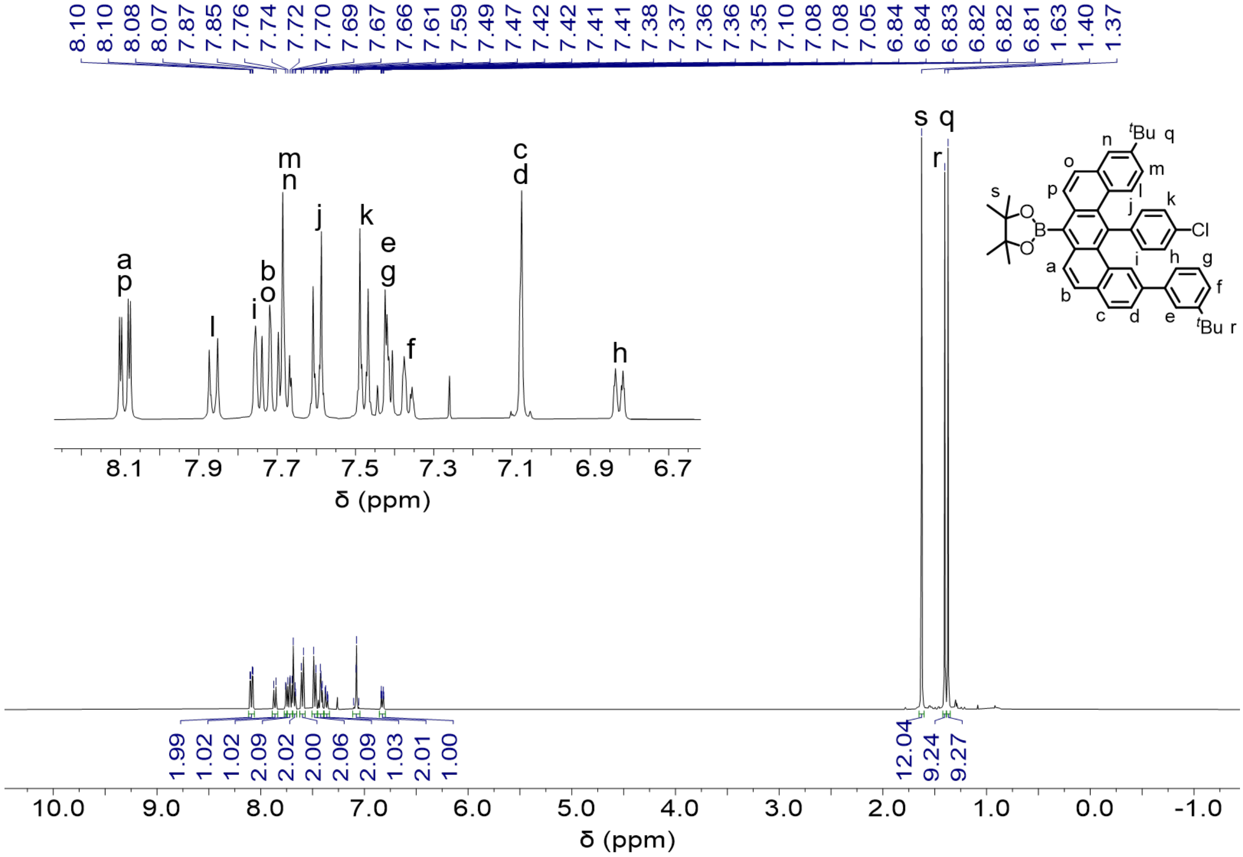

Supplementary Figure 46. ^{1}H NMR spectrum of benzo[*m*]tetraphene pinacol borate ester **12** (400 MHz, $CDCl_3$, 298 K).

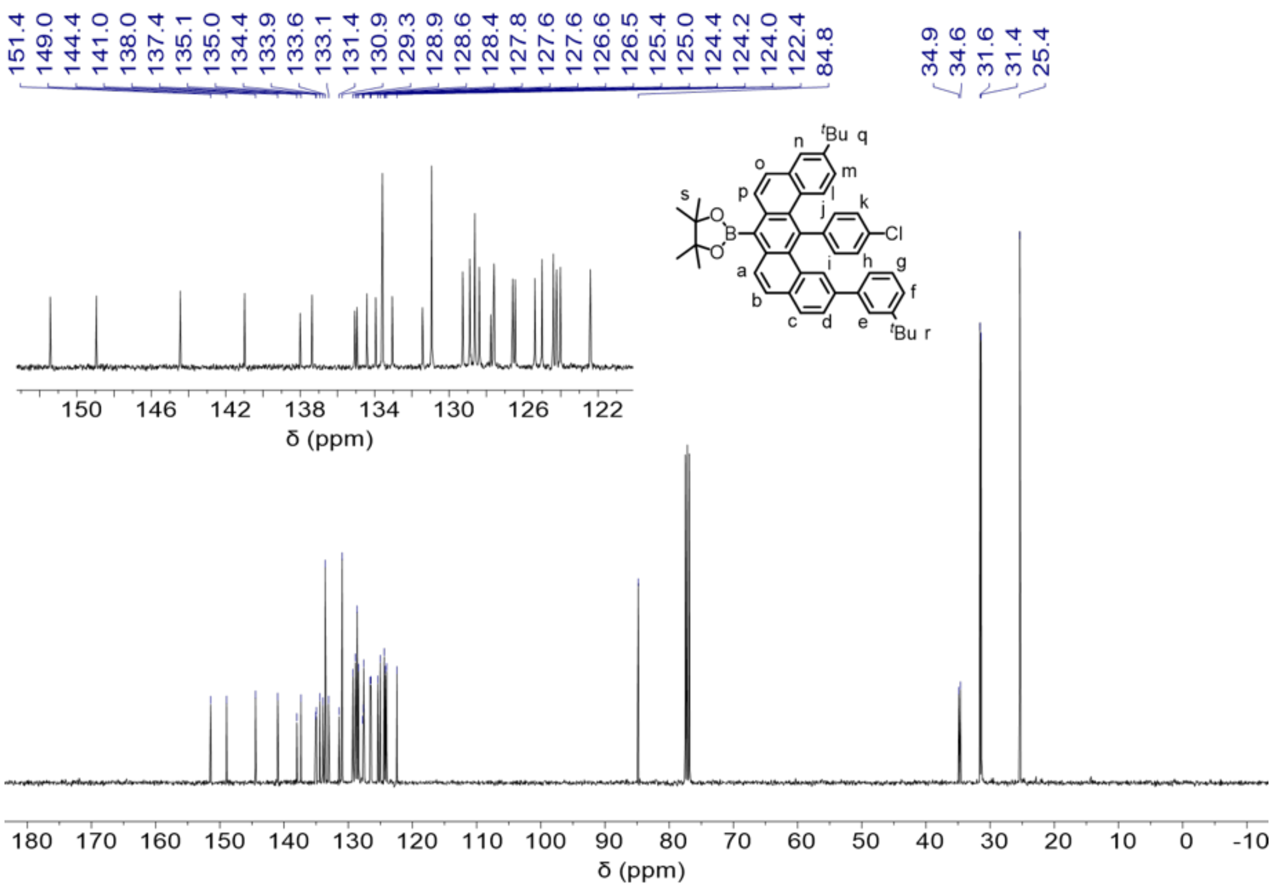

Supplementary Figure 47. ^{13}C NMR spectrum of benzo[*m*]tetraphene pinacol borate ester **12** (100 MHz, $CDCl_3$, 298 K).

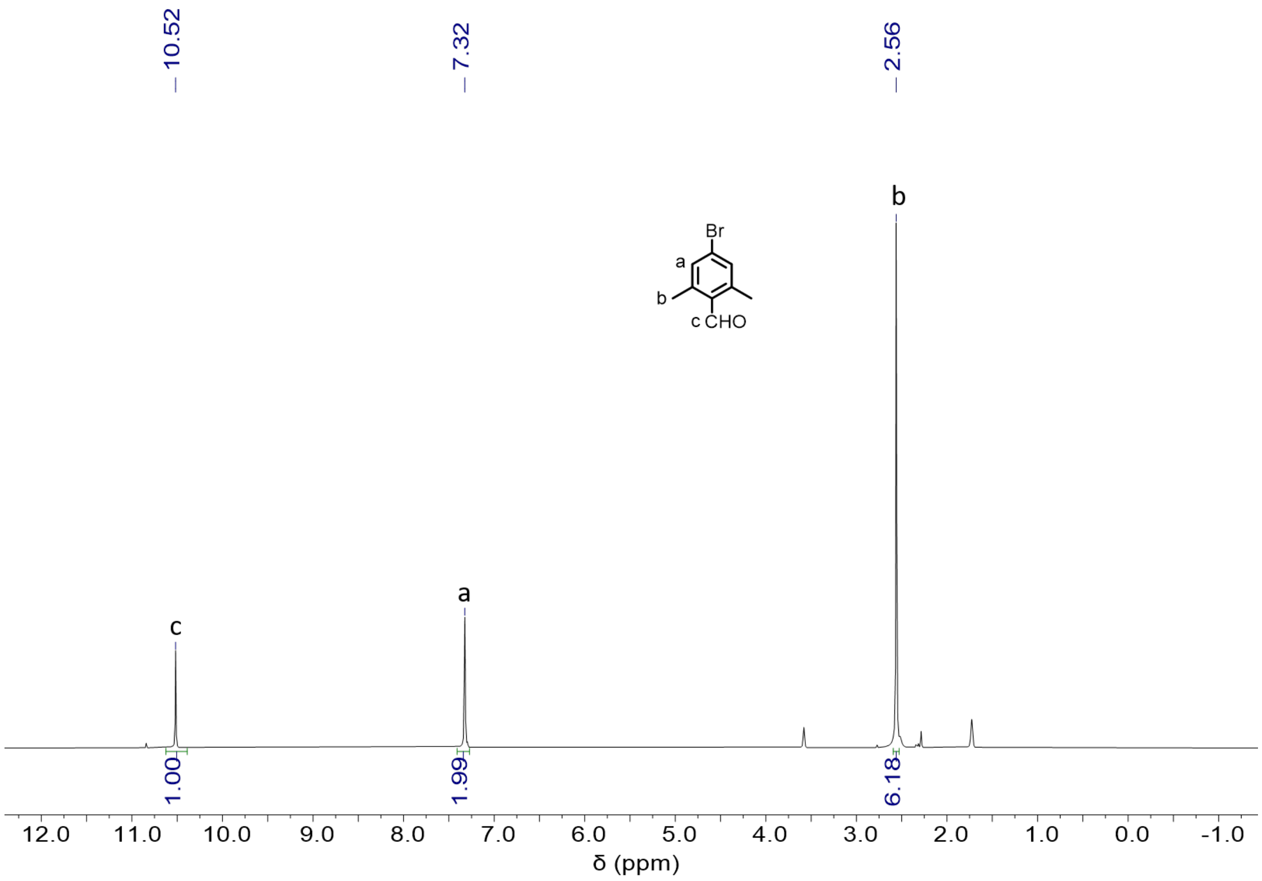

Supplementary Figure 48. ^{1}H NMR spectrum of 4-bromo-2,6-dimethylbenzaldehyde (**14**) (300 MHz, tetrahydrofuran-*d*8, 298 K).

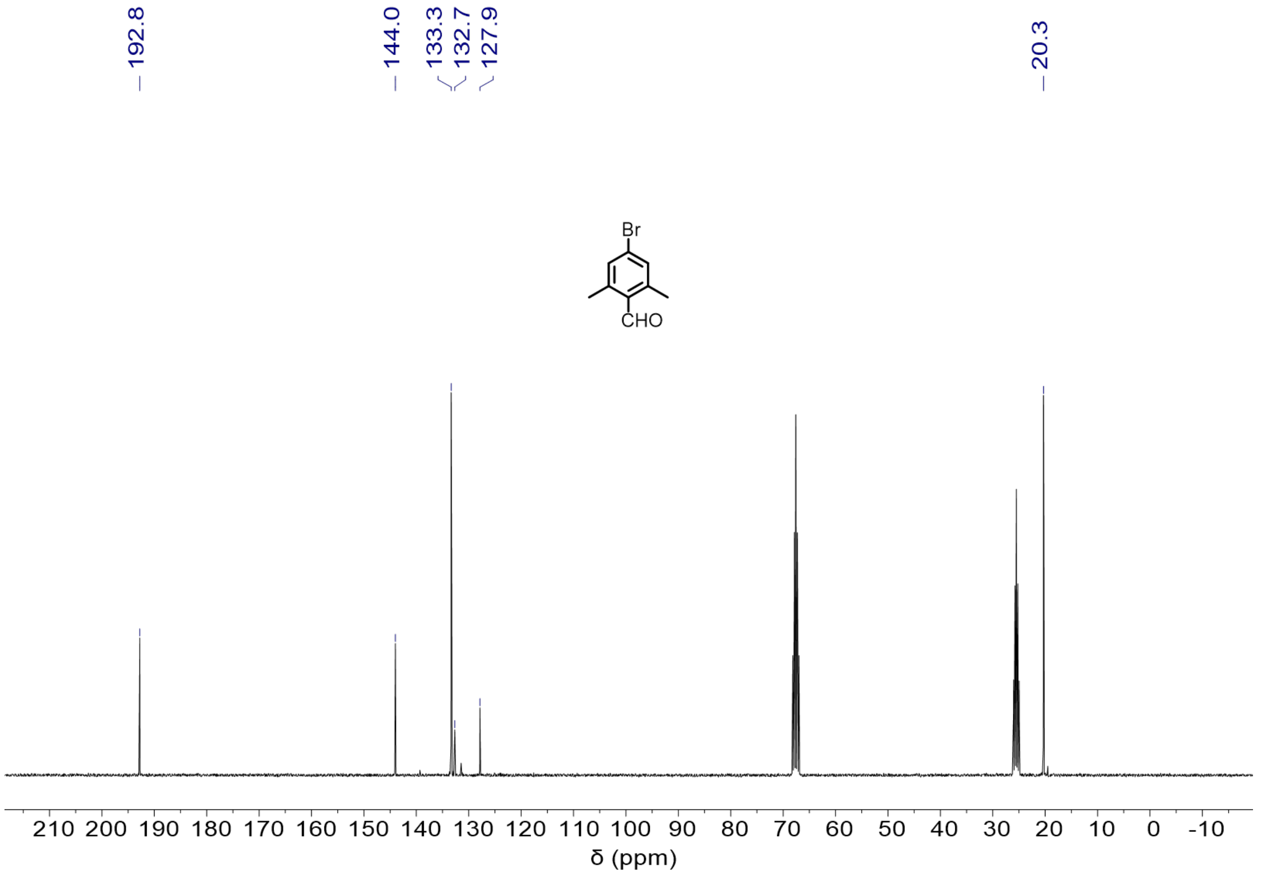

Supplementary Figure 49. ^{13}C NMR spectrum of 4-bromo-2,6-dimethylbenzaldehyde (**14**) (75 MHz, tetrahydrofuran-*d*8, 298 K).

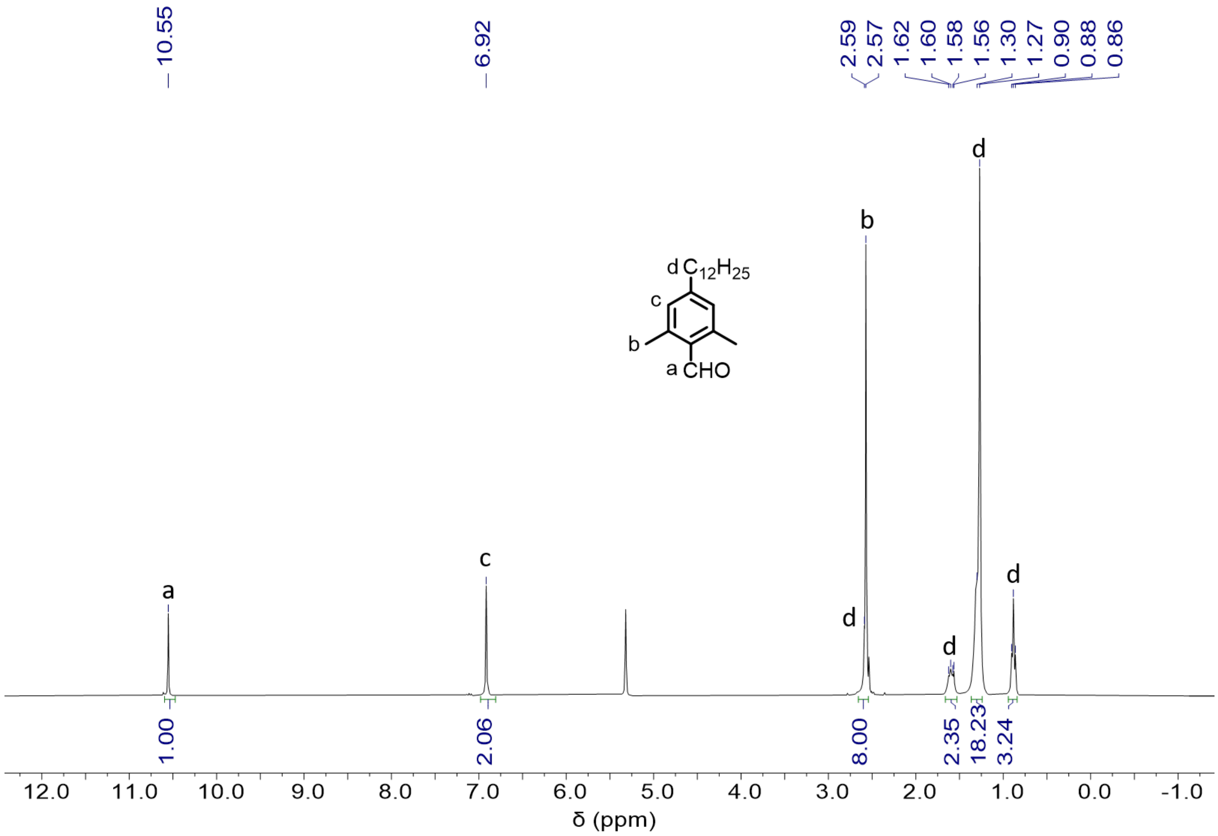

Supplementary Figure 50. ^{1}H NMR spectrum of 4-dodecyl-2,6-dimethylbenzaldehyde (**15**) (300 MHz, dichloromethane-d_2, 298 K).

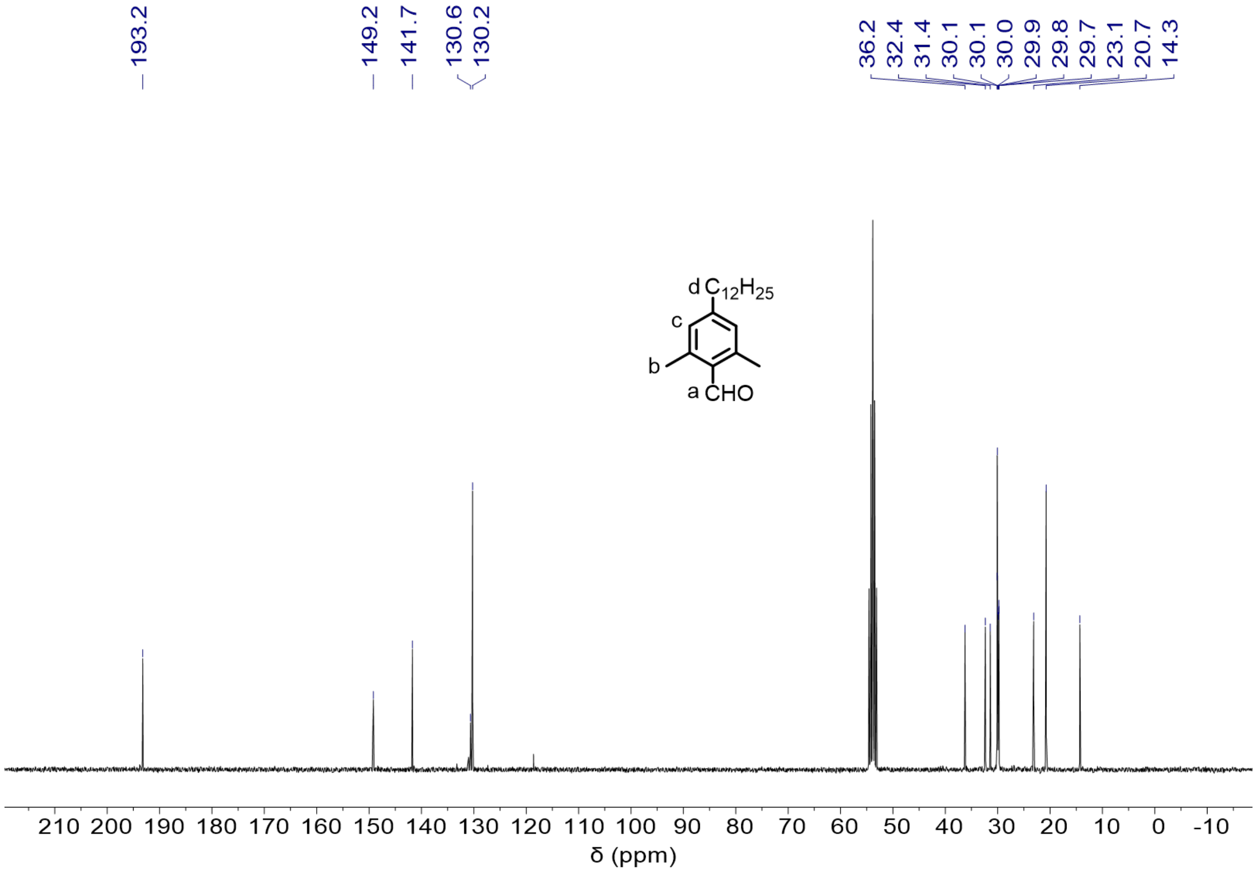

Supplementary Figure 51. ^{13}C NMR spectrum of 4-dodecyl-2,6-dimethylbenzaldehyde (**15**) (75 MHz, dichloromethane-d_2, 298 K).

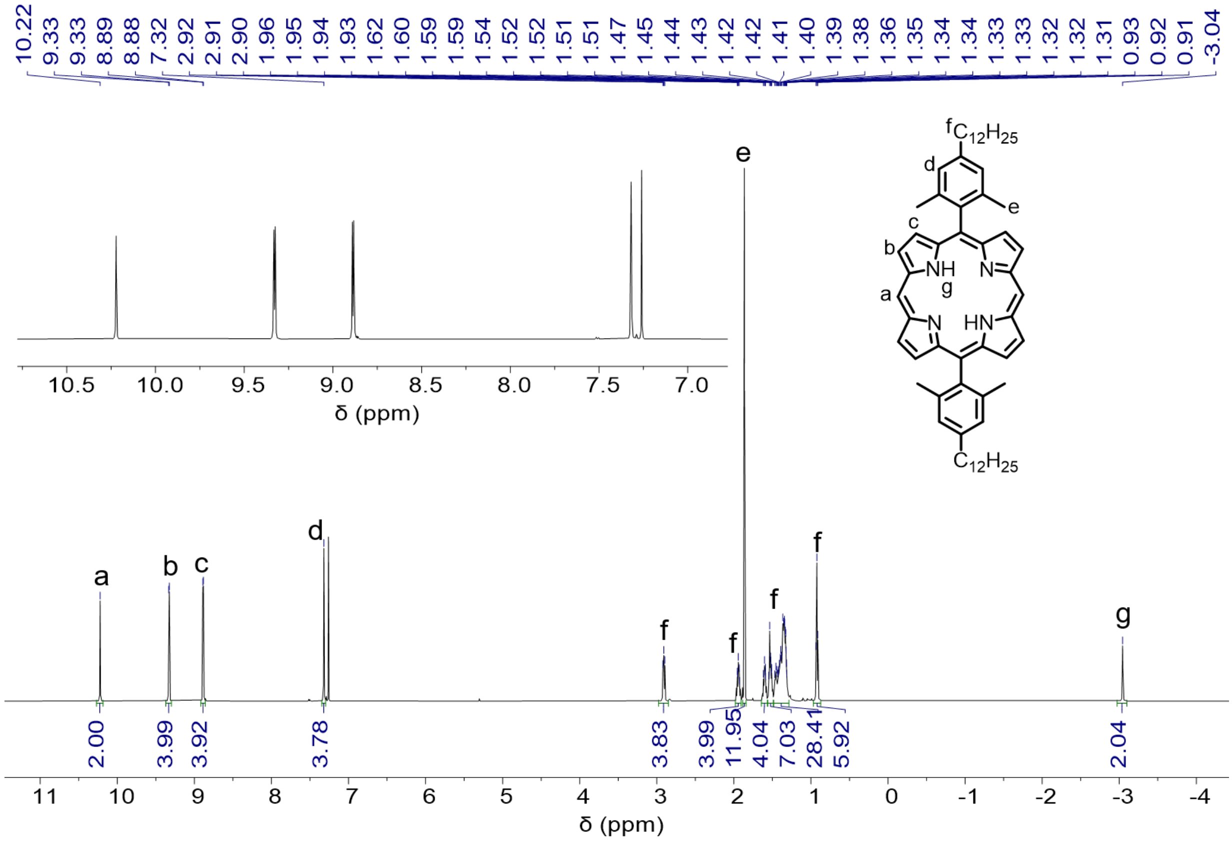

Supplementary Figure 52. ^{1}H NMR spectrum of 5,15-bis(2,6-dimethyl-4-dodecylphenyl)porphyrin (**16**) (600 MHz, $CDCl_3$, 298 K).

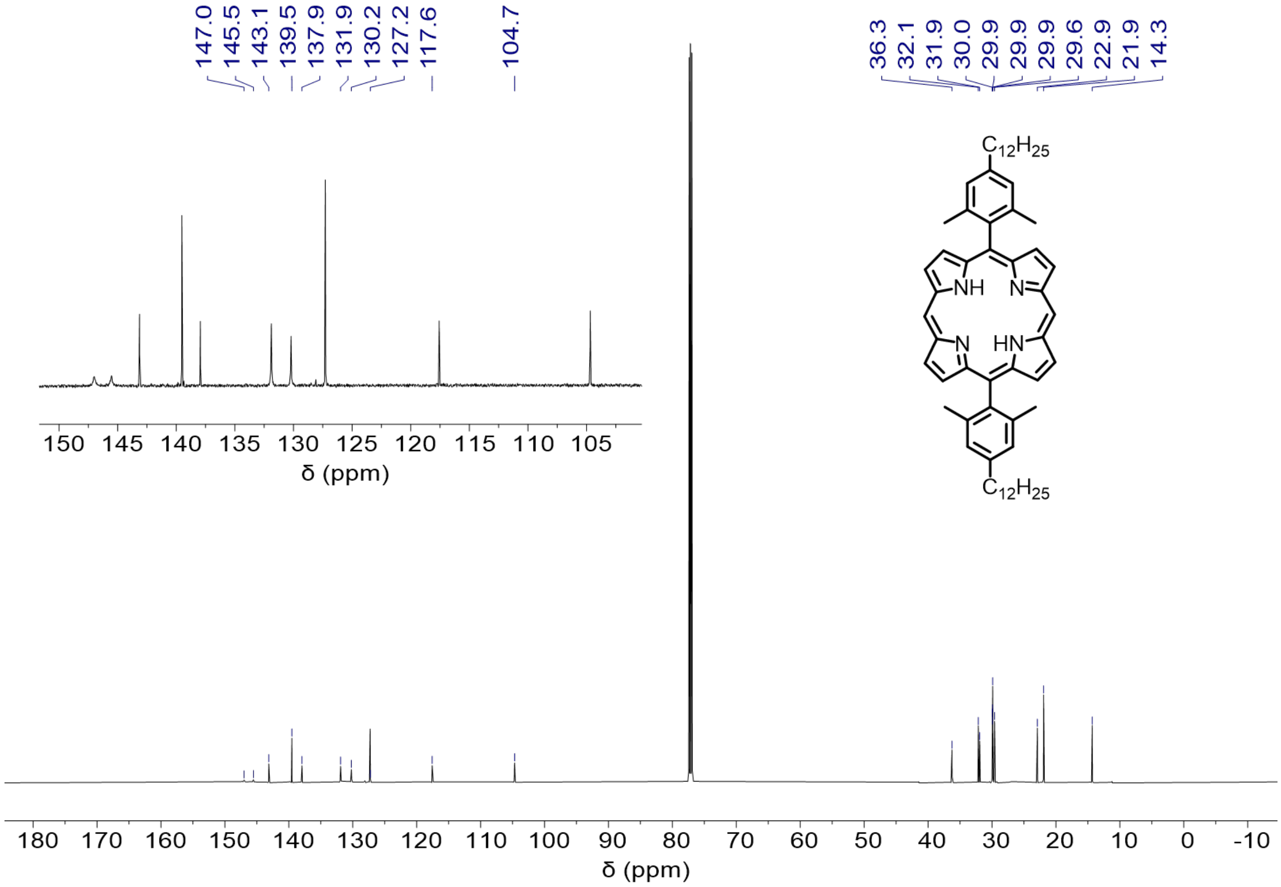

Supplementary Figure 53. ^{13}C NMR spectrum of 5,15-bis(2,6-dimethyl-4-dodecylphenyl)porphyrin (**16**) (150 MHz, $CDCl_3$, 298 K).

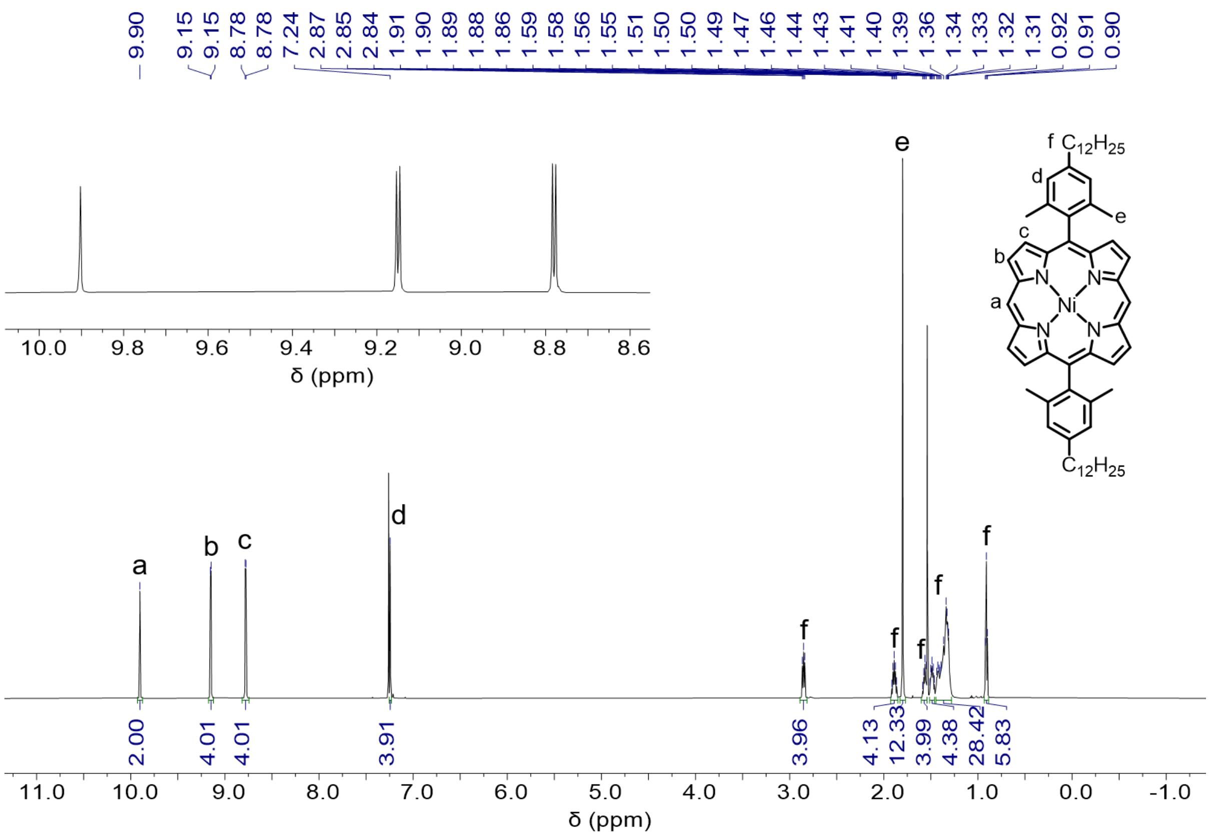

Supplementary Figure 54. ^{1}H NMR spectrum of 5,15-bis(2,6-dimethyl-4-dodecylphenyl)porphyrin (Ni) (**17**) (600 MHz, $CDCl_3$, 298 K).

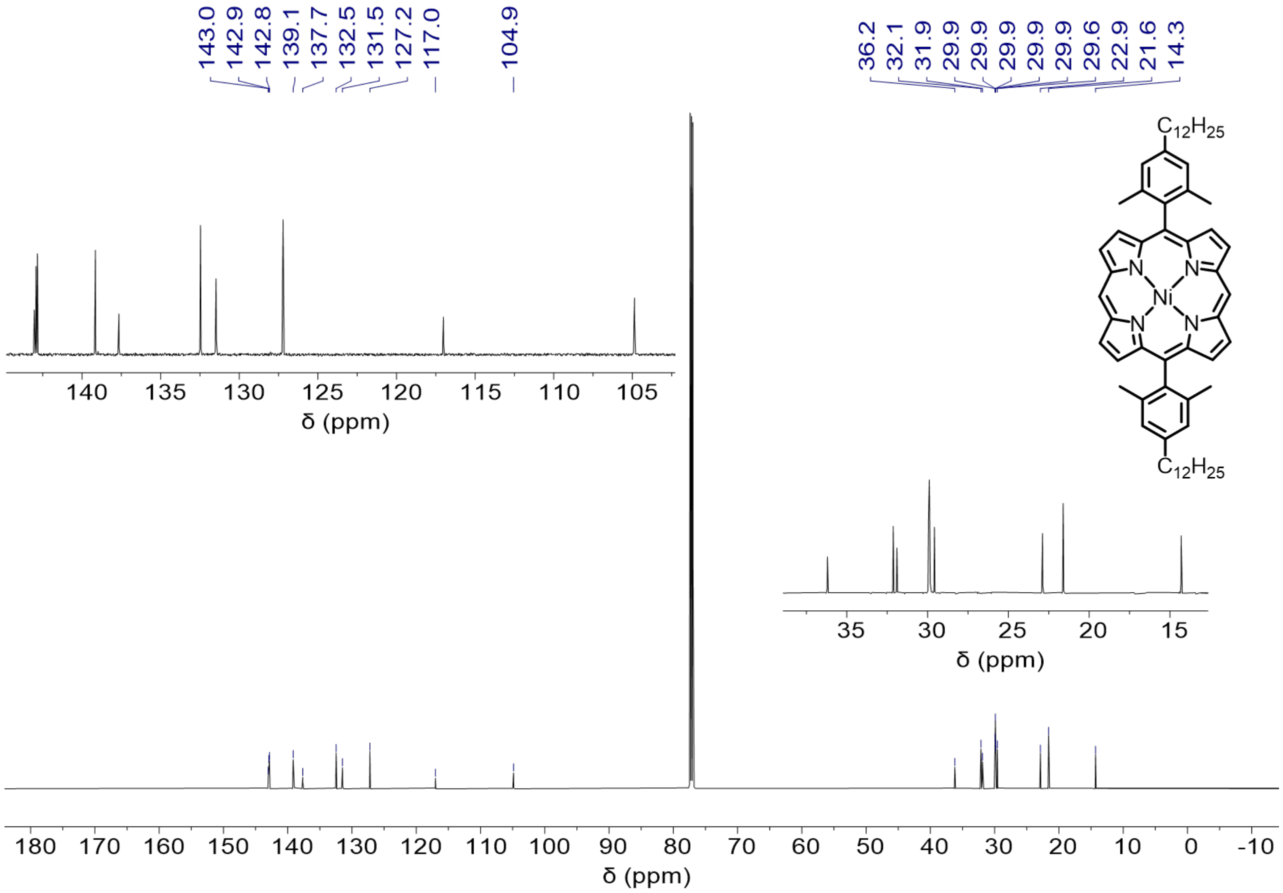

Supplementary Figure 55. ^{13}C NMR spectrum of 5,15-bis(2,6-dimethyl-4-dodecylphenyl)porphyrin (Ni) (**17**) (150 MHz, $CDCl_3$, 298 K).

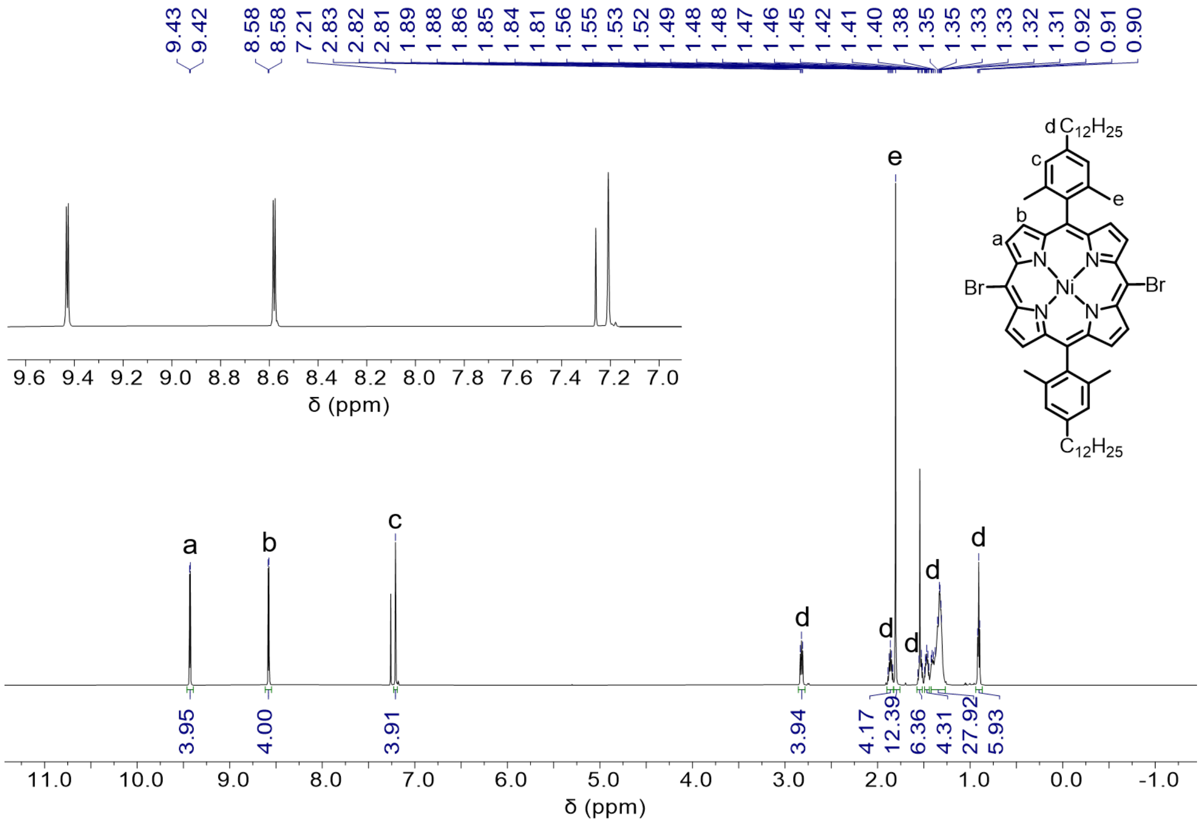

Supplementary Figure 56. ^{1}H NMR spectrum of 5,15-dibromo-10,20-bis(2,6-dimethyl-4-dodecylphenyl)porphyrin (Ni) (**18b**) (600 MHz, $CDCl_3$, 298 K).

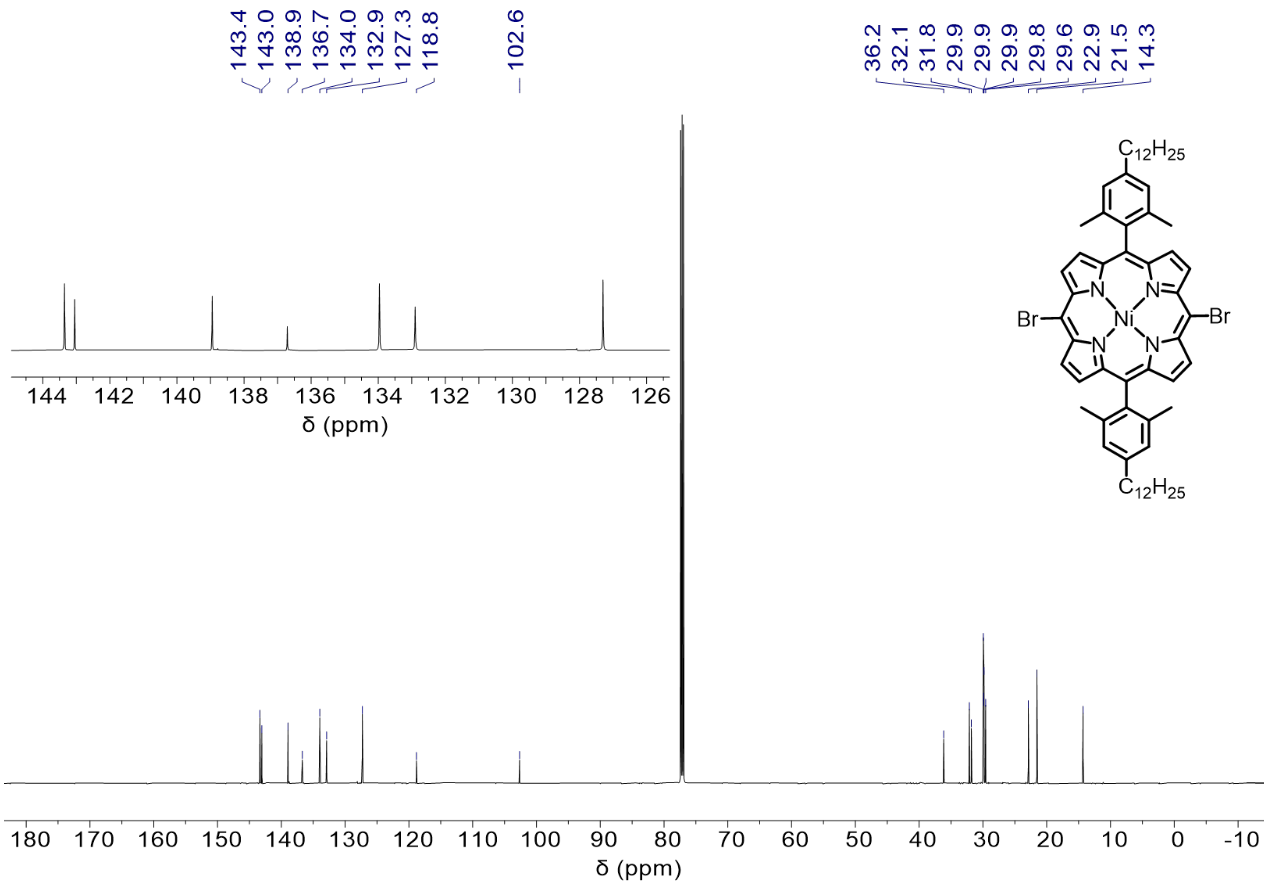

Supplementary Figure 57. ^{13}C NMR spectrum of 5,15-dibromo-10,20-bis(2,6-dimethyl-4-dodecylphenyl)porphyrin (Ni) (**18b**) (150 MHz, $CDCl_3$, 298 K).

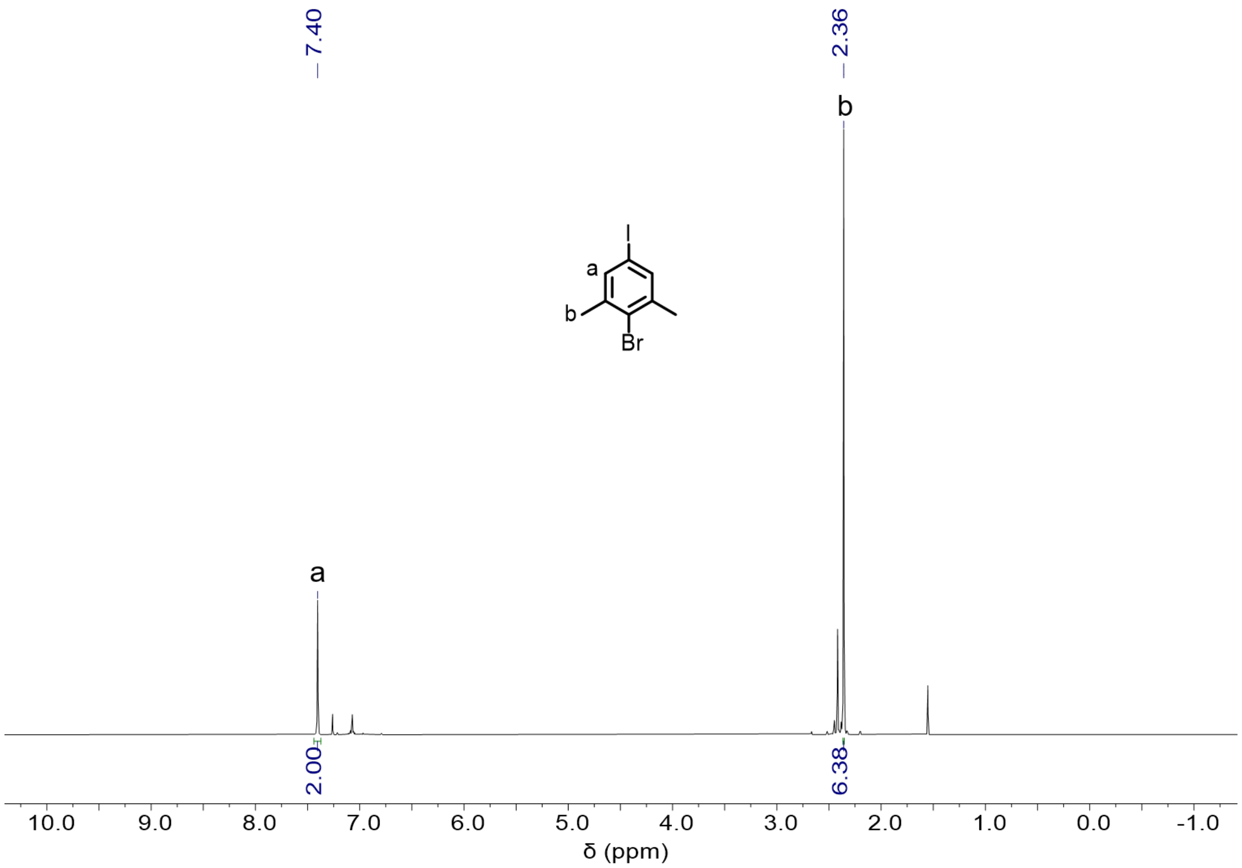

Supplementary Figure 58. ^{1}H NMR spectrum of 2-bromo-5-iodo-1,3-dimethylbenzene (**20**) (400 MHz, $CDCl_3$, 298 K).

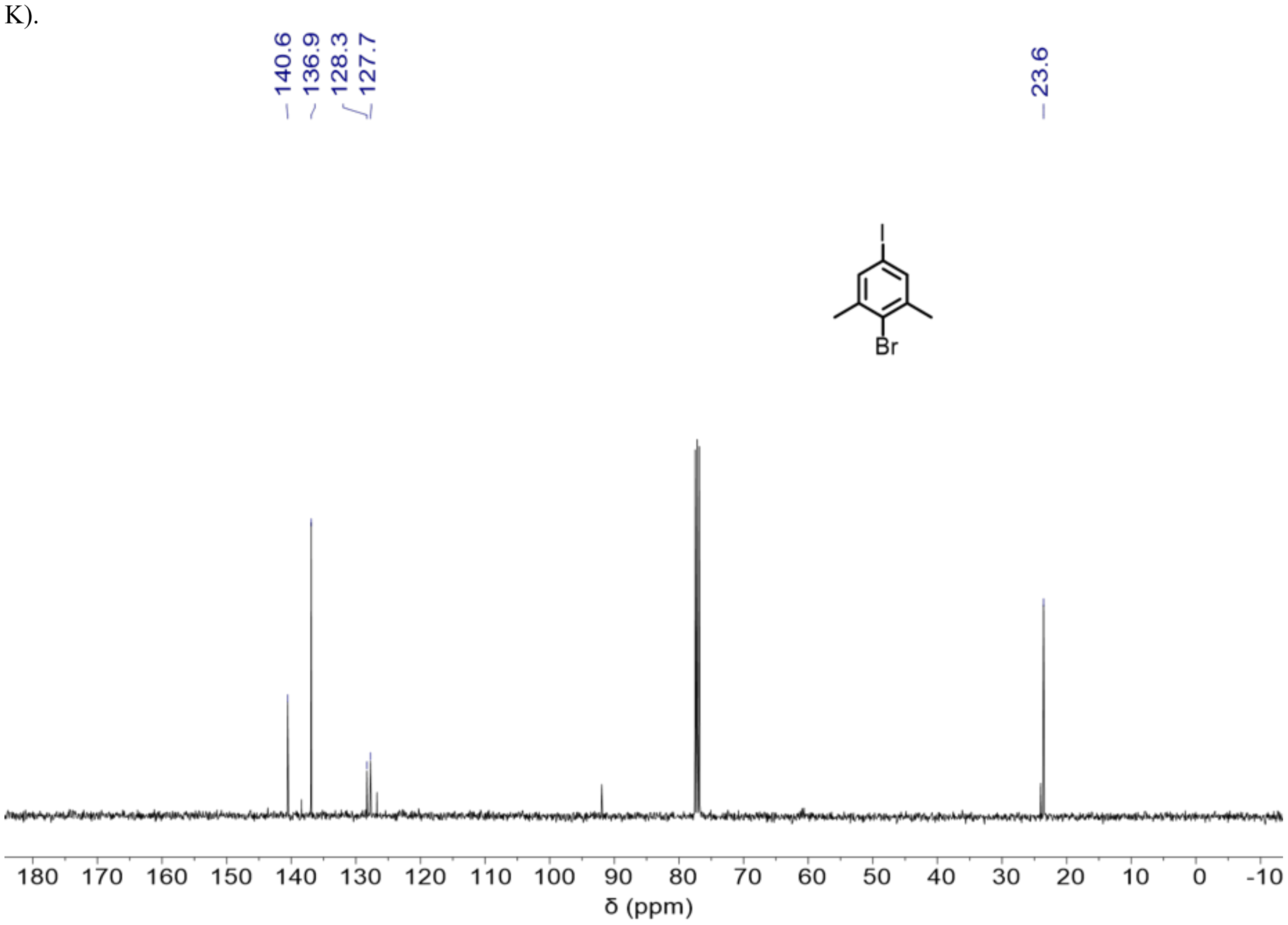

Supplementary Figure 59. ^{1}H NMR spectrum of 2-bromo-5-iodo-1,3-dimethylbenzene (**20**) (125 MHz, $CDCl_3$, 298 K).

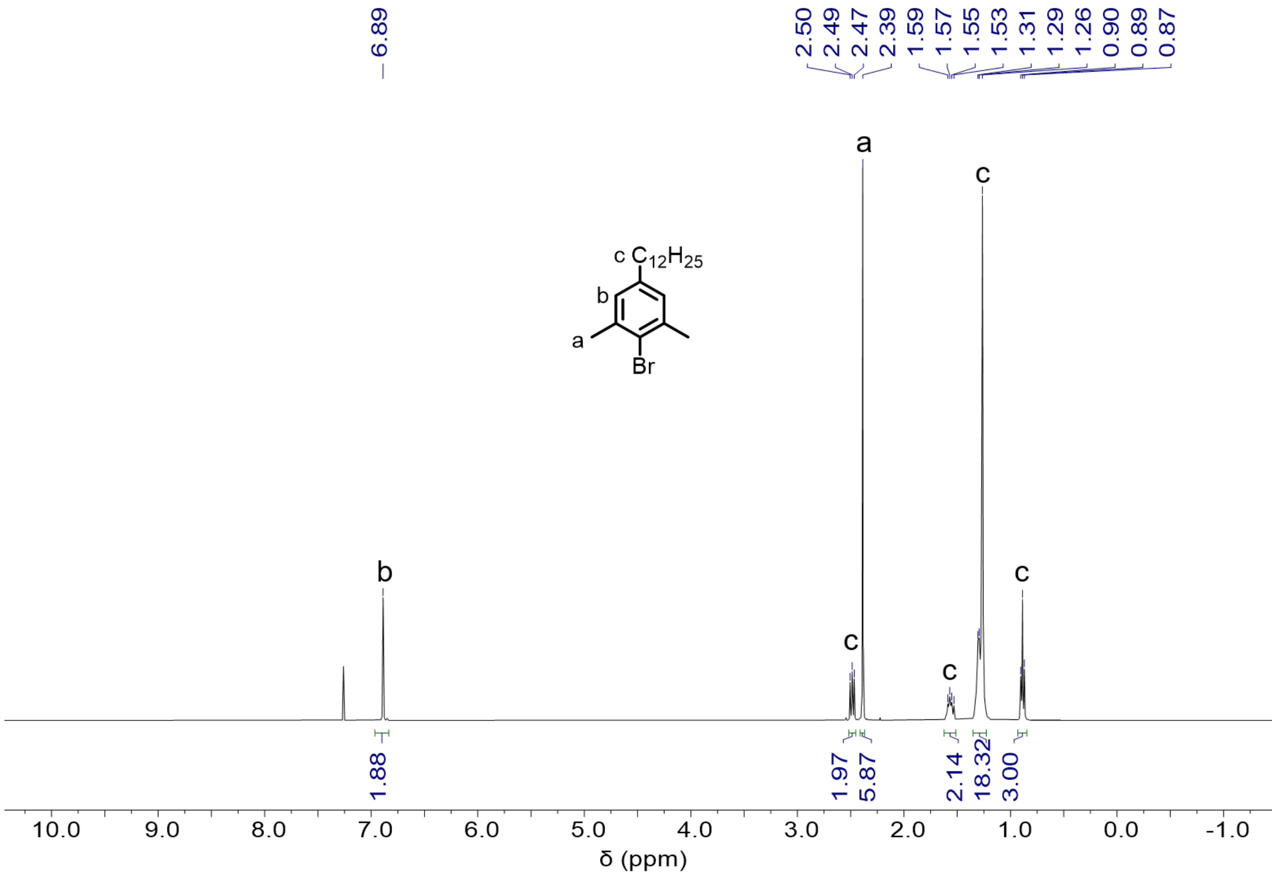

Supplementary Figure 60. ^{1}H NMR spectrum of 2-bromo-5-dodecyl-1,3-dimethylbenzene (**21**) (400 MHz, $CDCl_3$, 298 K).

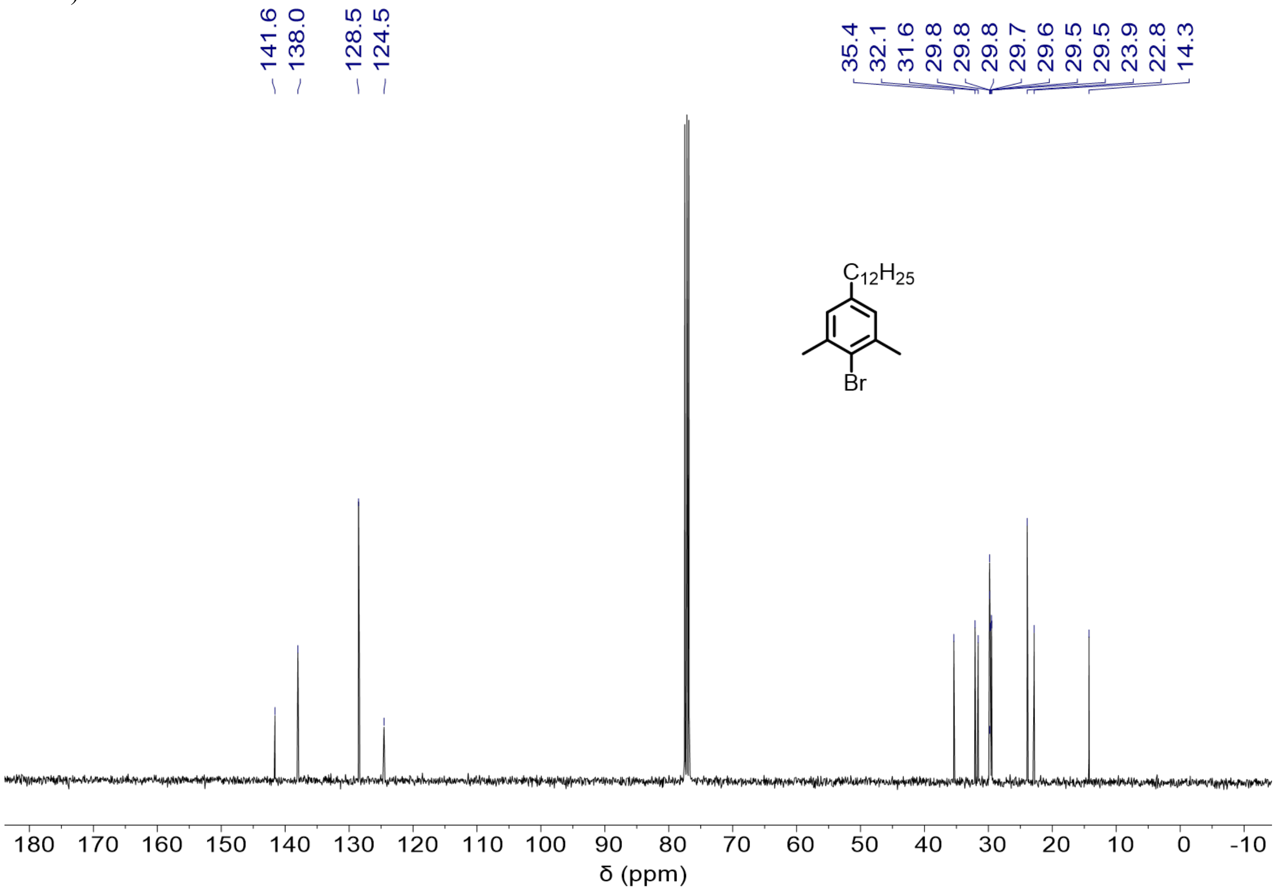

Supplementary Figure 61. ^{13}C NMR spectrum of 2-bromo-5-dodecyl-1,3-dimethylbenzene (**21**) (125 MHz, $CDCl_3$, 298 K).

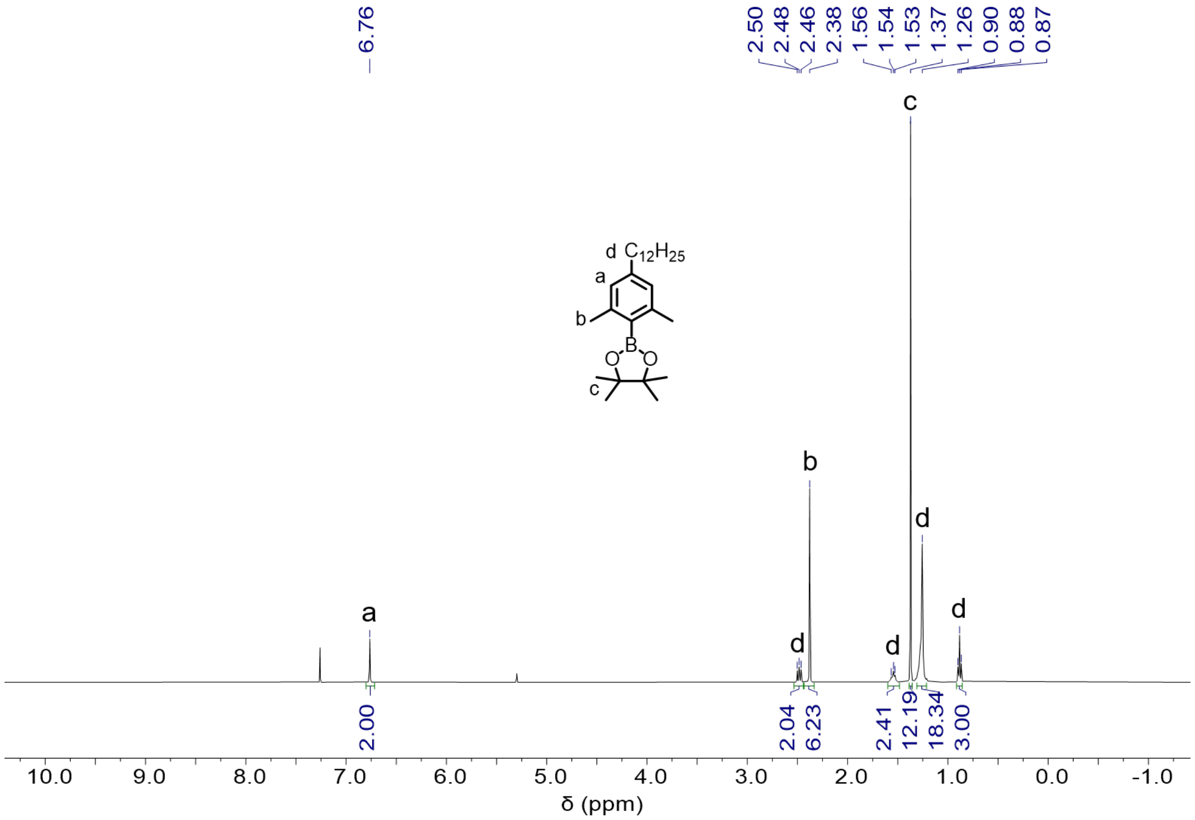

Supplementary Figure 62. ^{1}H NMR spectrum of 2,6-dimethyl-4-dodecylphenylboronic acid pinacol ester (**22b**) (400 MHz, $CDCl_3$, 298 K).

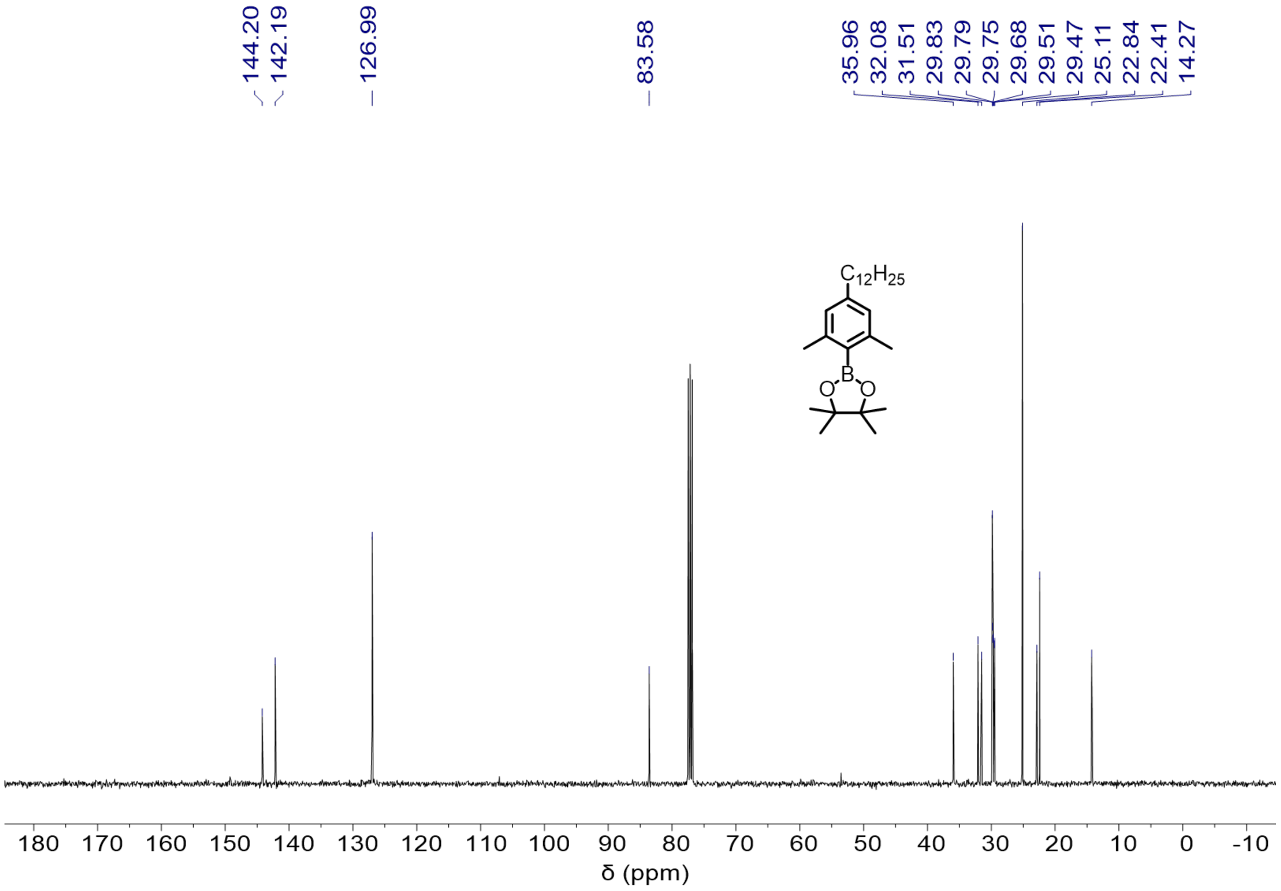

Supplementary Figure 63. ^{13}C NMR spectrum of 2,6-dimethyl-4-dodecylphenylboronic acid pinacol ester (**22b**) (75 MHz, $CDCl_3$, 298 K).

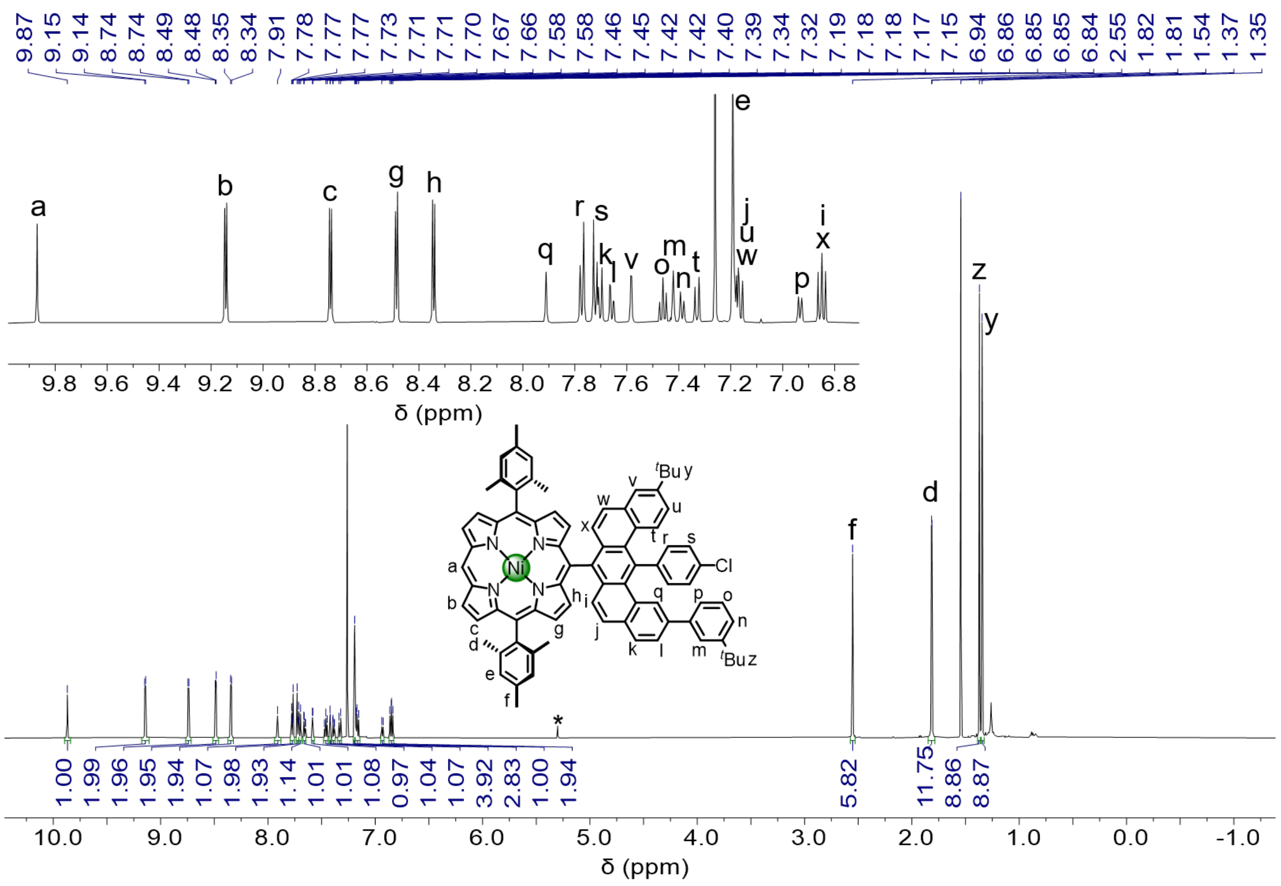

Supplementary Figure 64. ^{1}H NMR spectrum of porphyrin-benzo[*m*]tetraphene conjugate **23a** (600 MHz, $CDCl_3$, 298 K). * indicates residue dichloromethane peak.

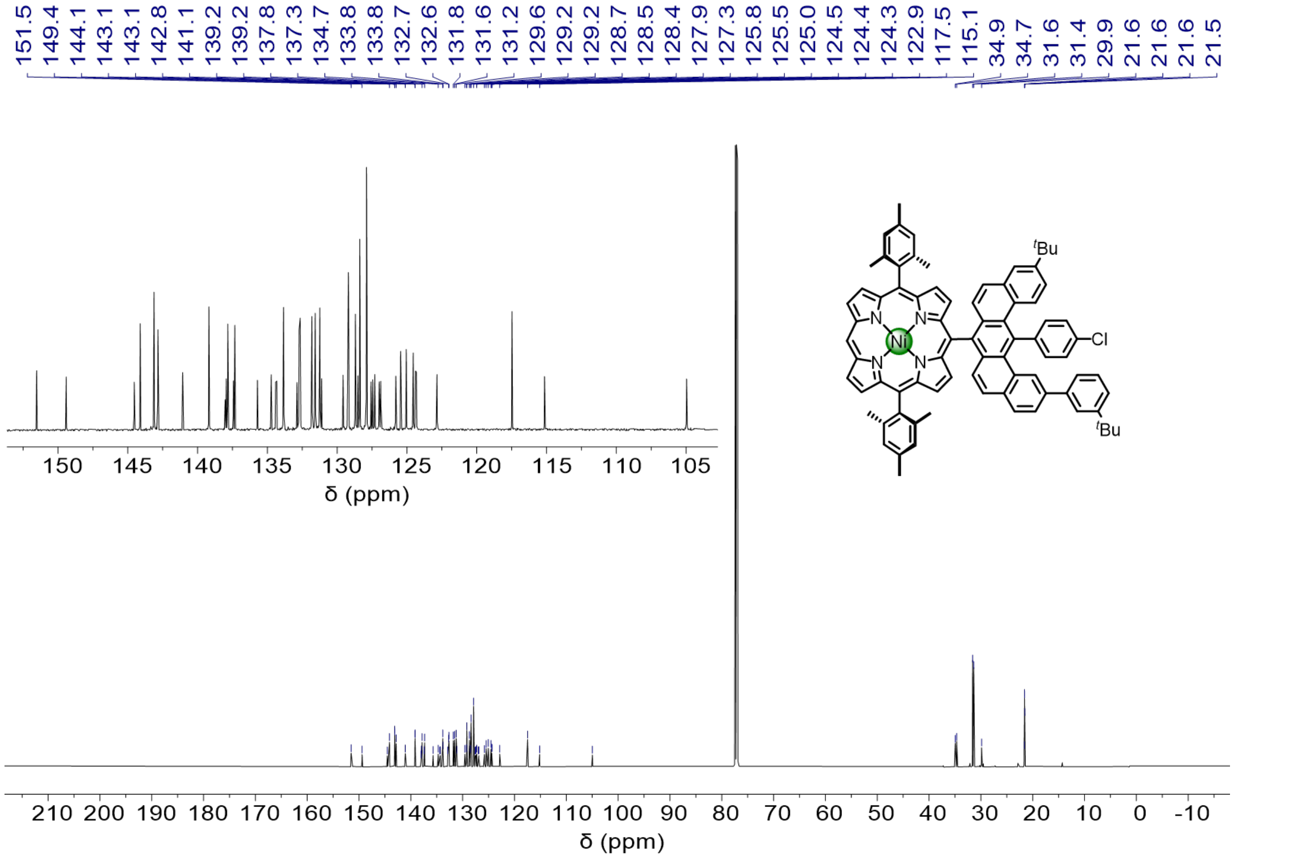

Supplementary Figure 65. ^{13}C NMR spectrum of porphyrin-benzo[*m*]tetraphene conjugate **23a** (150 MHz, $CDCl_3$, 298 K).

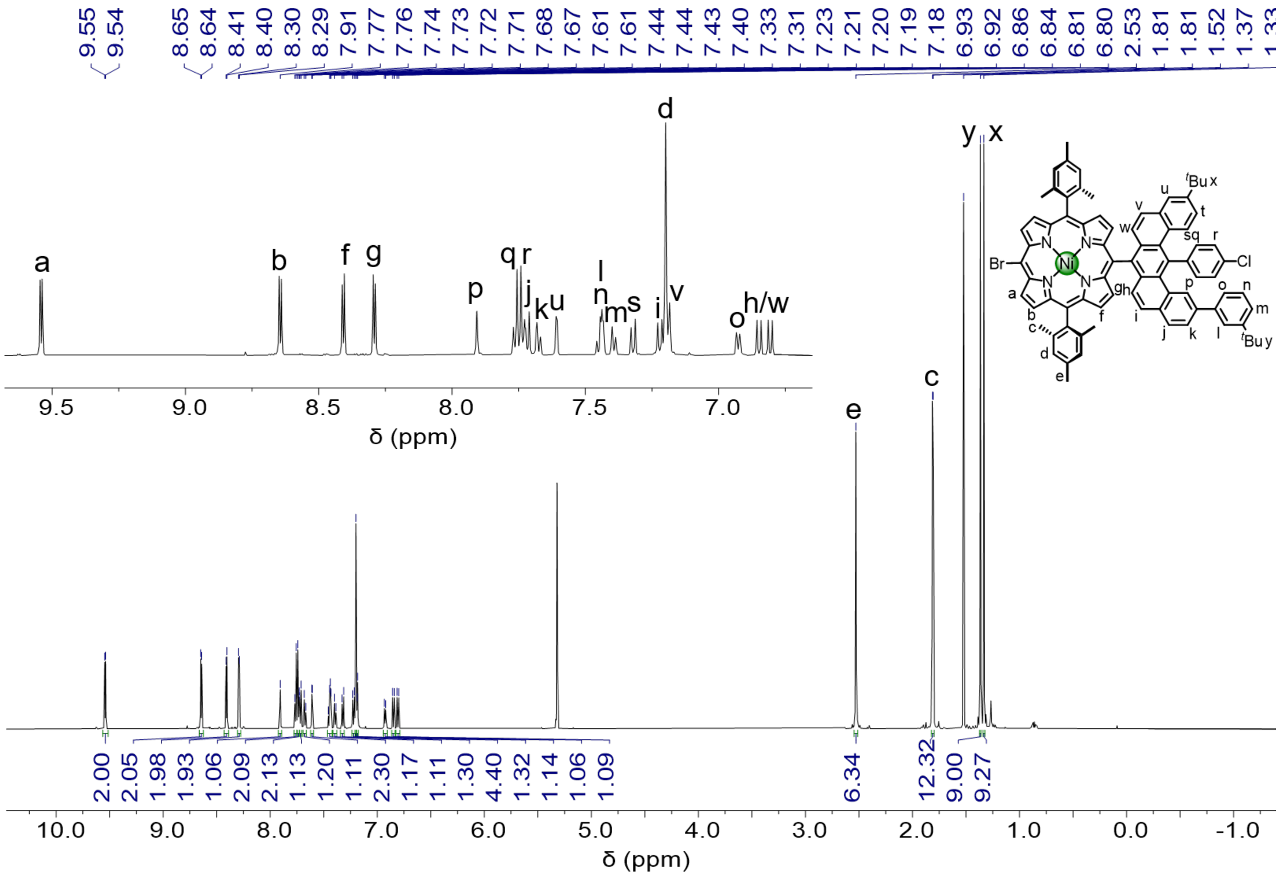

Supplementary Figure 66. ^{1}H NMR spectrum of *meso*-monobromoporphyrin **24a** (600 MHz, CD_2Cl_2, 298 K).

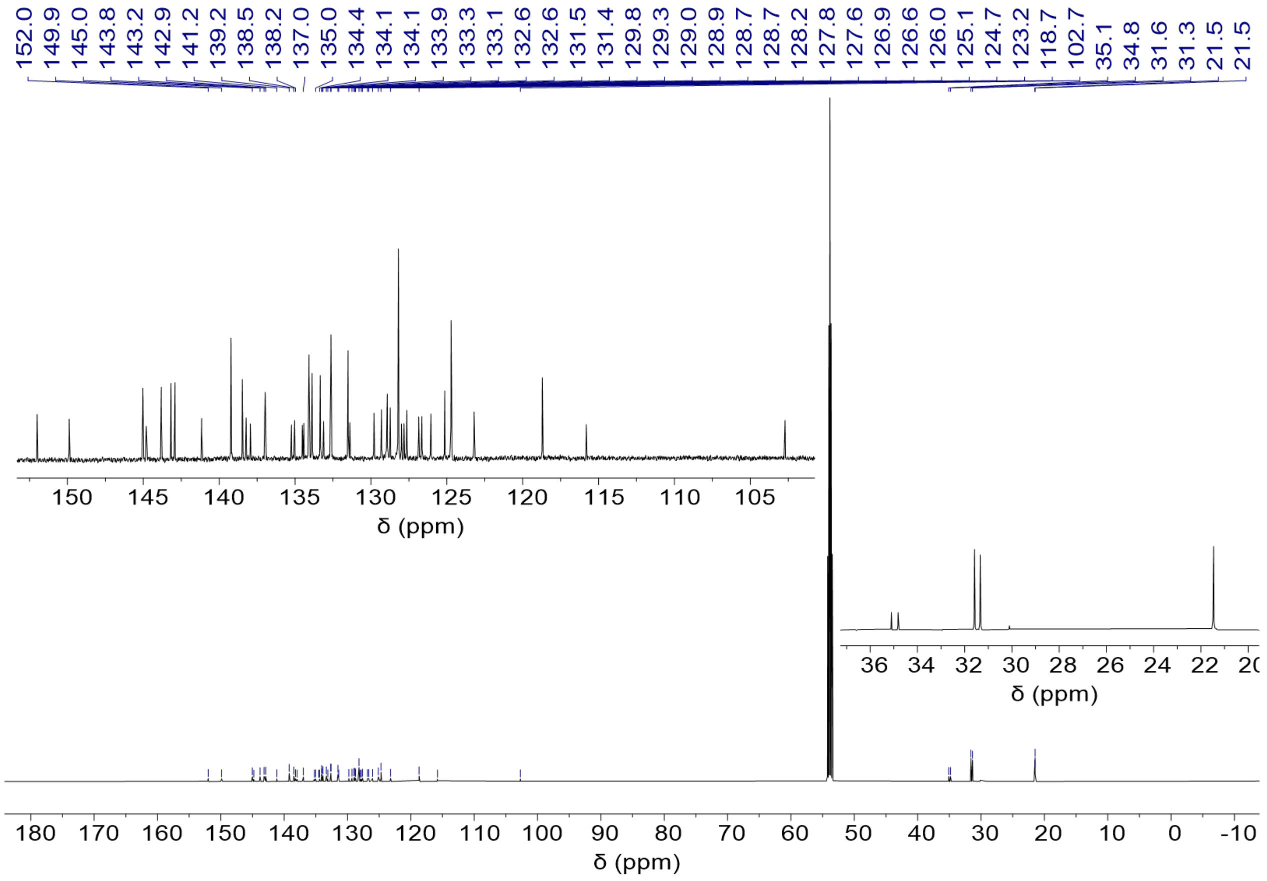

Supplementary Figure 67. ^{13}C NMR spectrum of *meso*-monobromoporphyrin **24a** (150 MHz, CD_2Cl_2, 298 K).

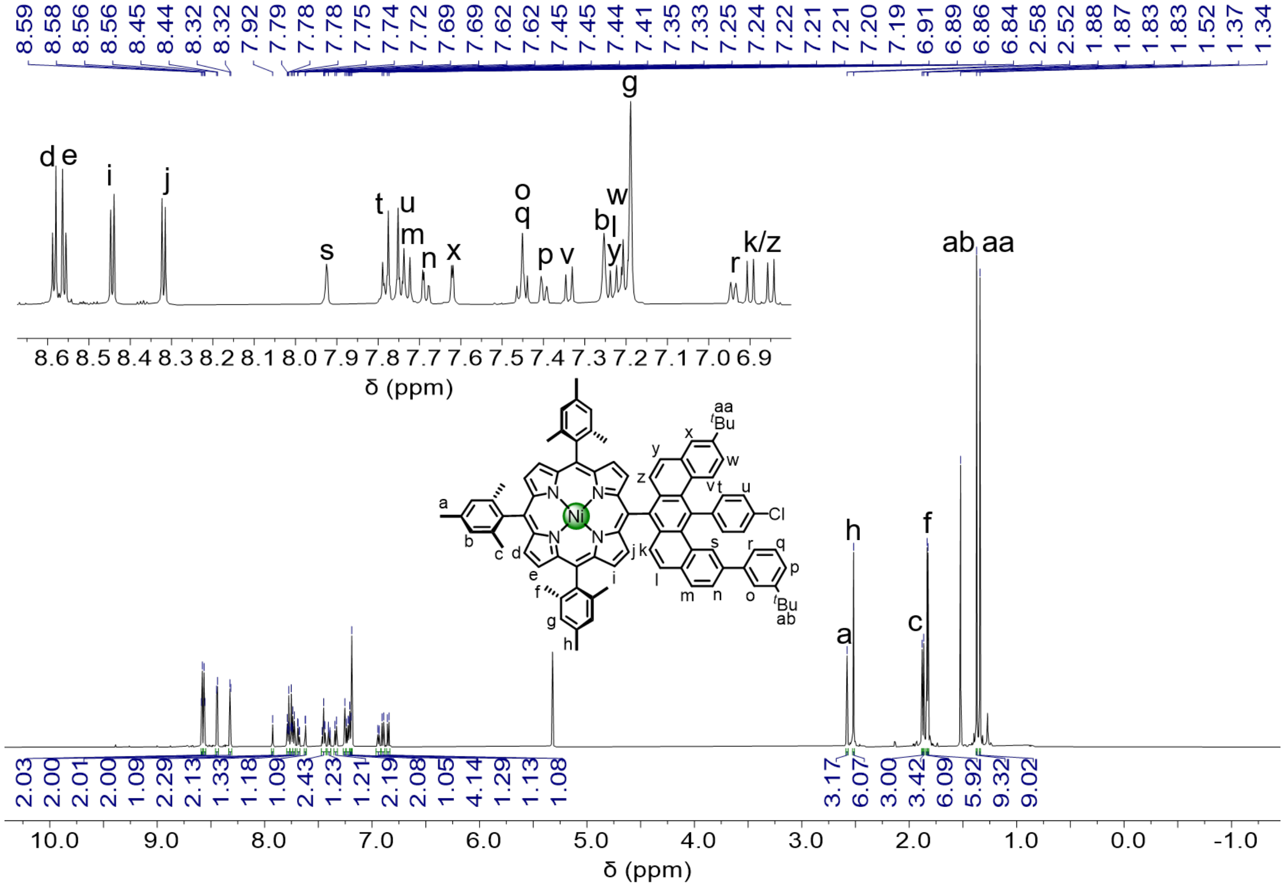

Supplementary Figure 68. ^{1}H NMR spectrum of trimesitylporphyrin **1a** (600 MHz, CD_2Cl_2, 298 K).

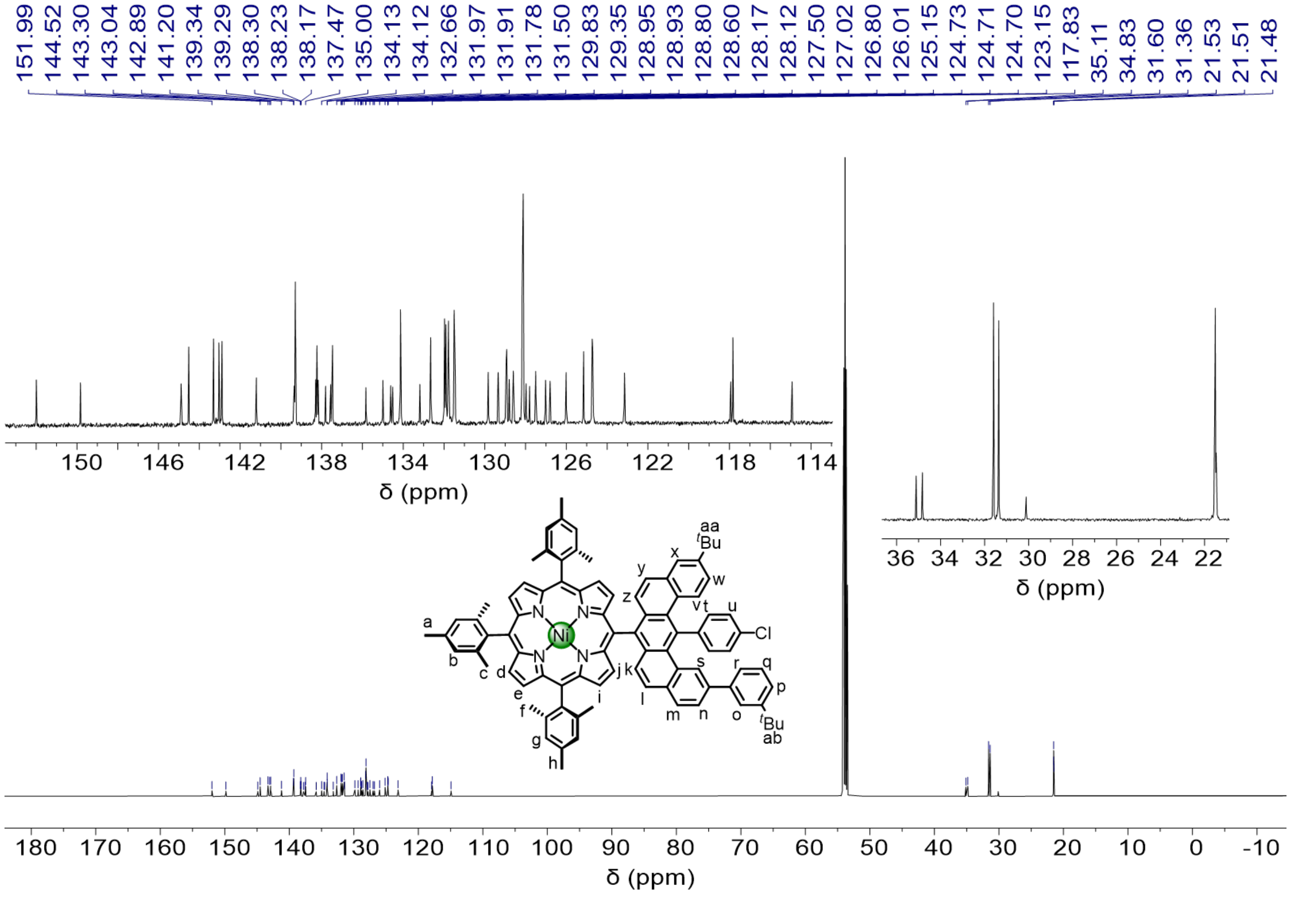

Supplementary Figure 69. ^{13}C NMR spectrum of trimesitylporphyrin **1a** (150 MHz, CD_2Cl_2, 298 K).

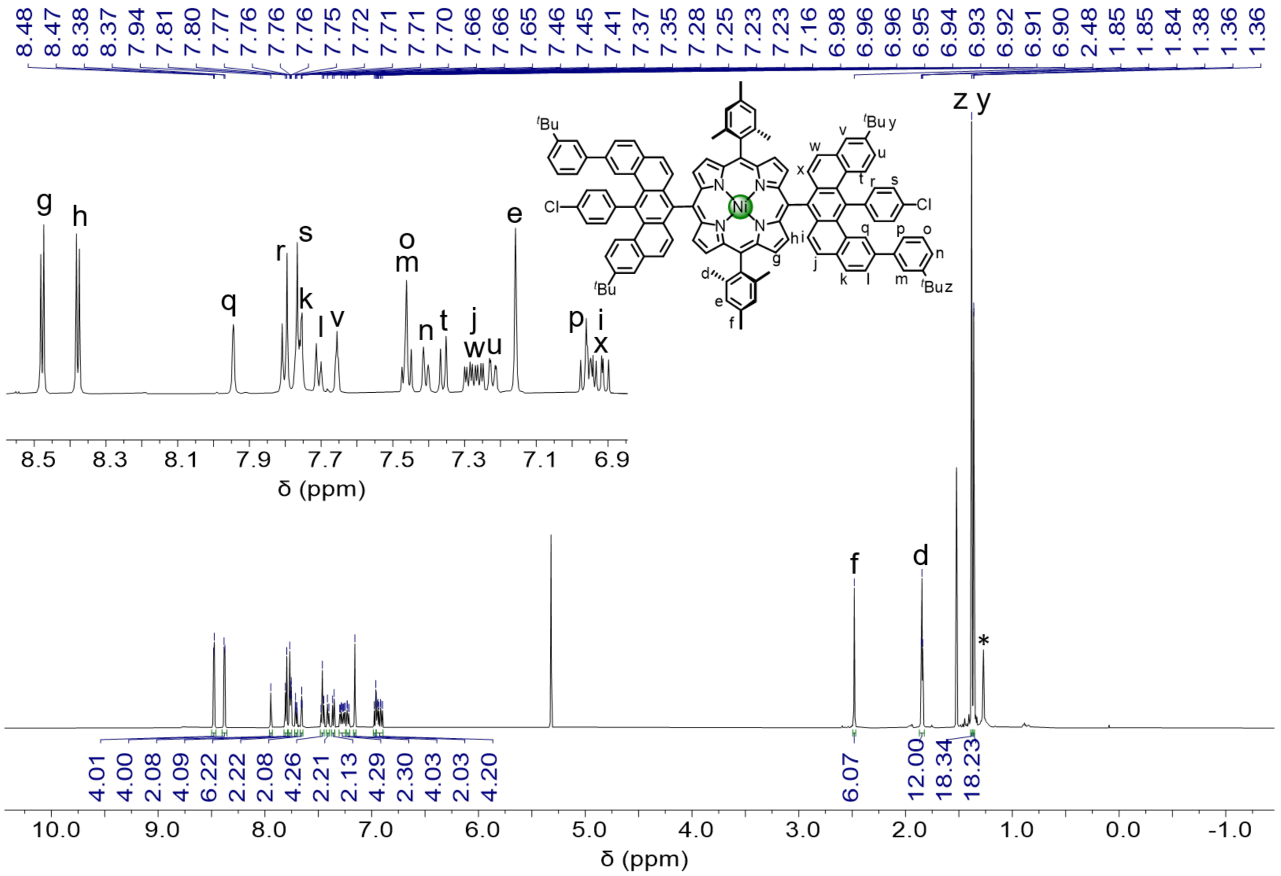

Supplementary Figure 70. ^{1}H NMR spectrum of porphyrin **2a** (600 MHz, CD_2Cl_2, 298 K). (*) indicates H-grease.

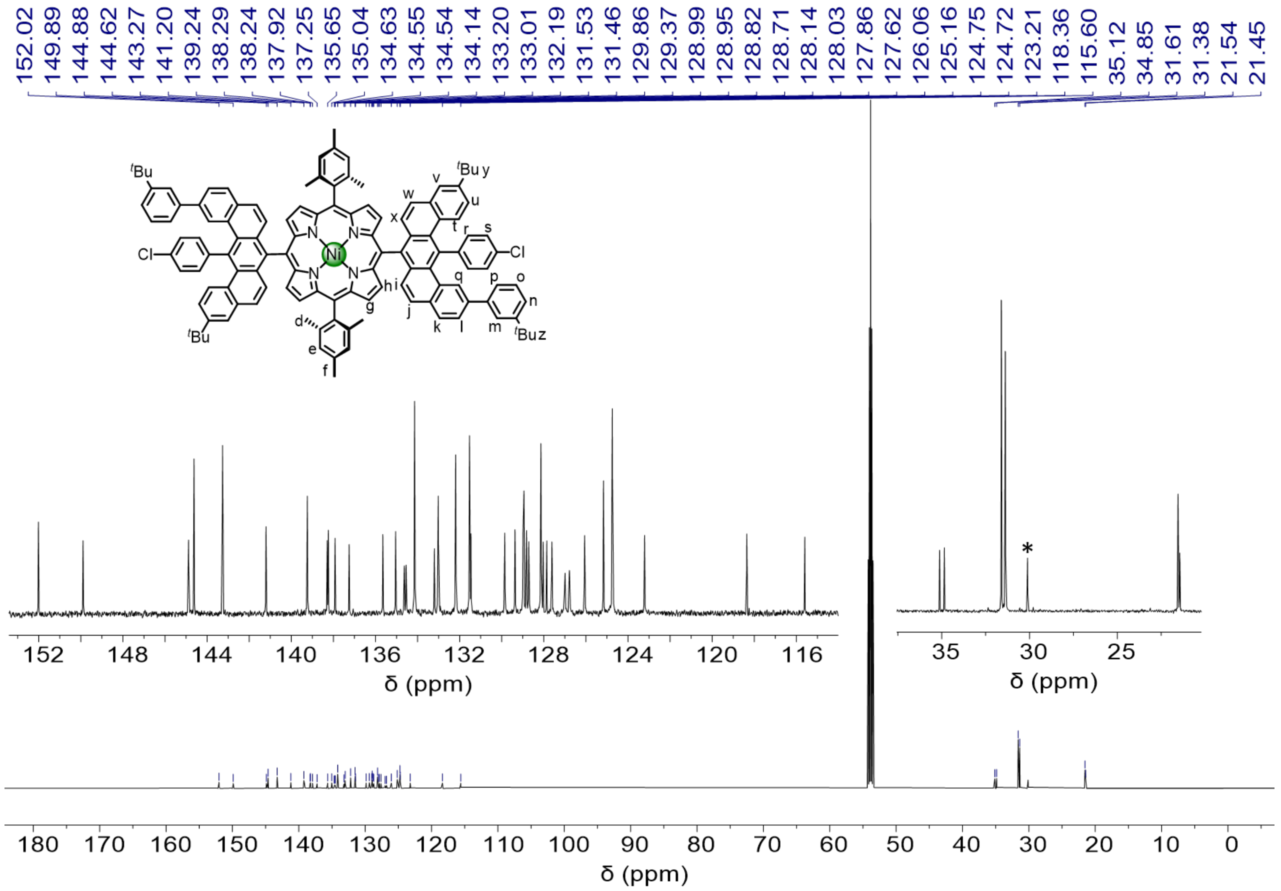

Supplementary Figure 71. ^{13}C NMR spectrum of porphyrin **2a** (150 MHz, CD_2Cl_2, 298 K). (*) indicates H-grease.

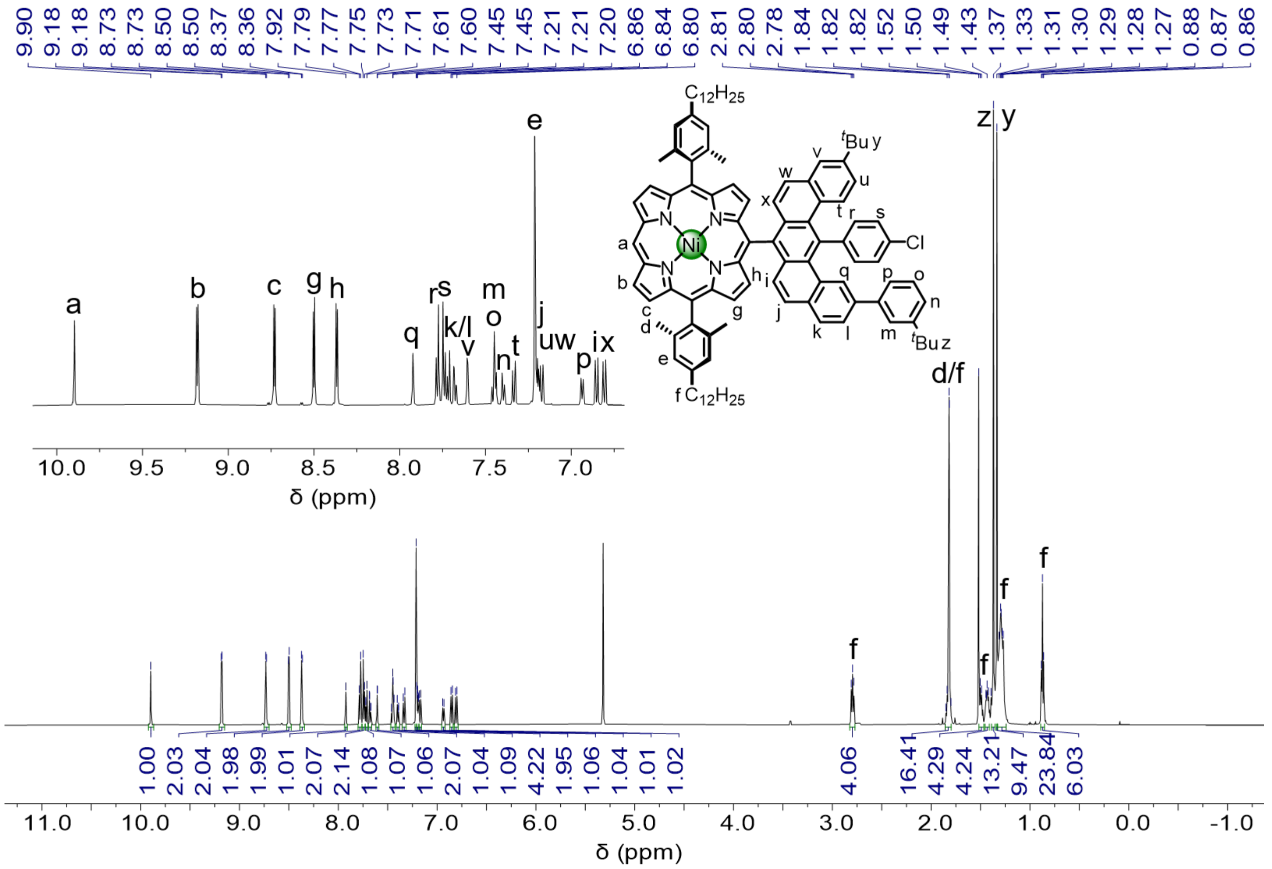

Supplementary Figure 72. ^{1}H NMR spectrum of porphyrin **23b** (600 MHz, CD_2Cl_2, 298 K).

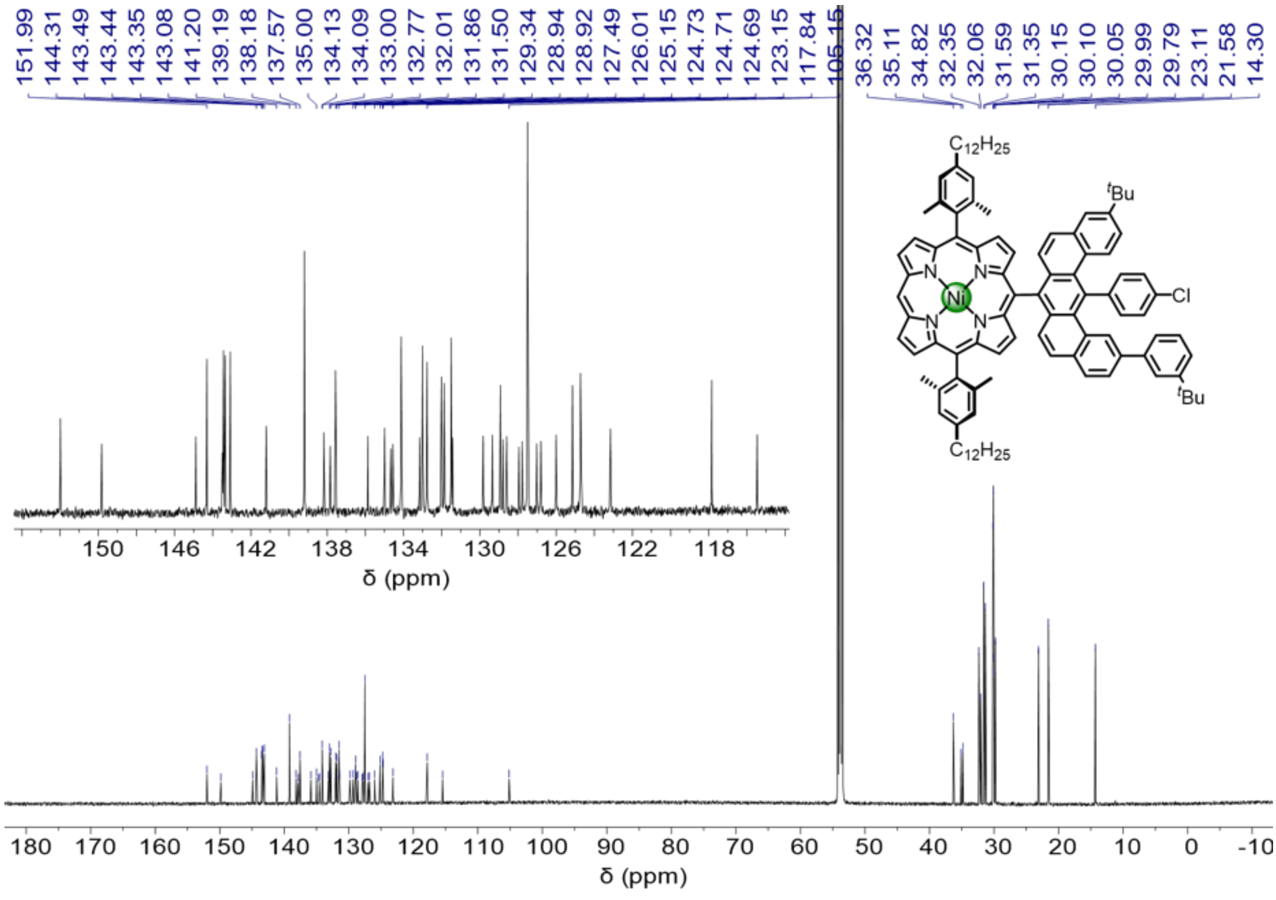

Supplementary Figure 73. ^{13}C NMR spectrum of porphyrin **23b** (150 MHz, CD_2Cl_2, 298 K).

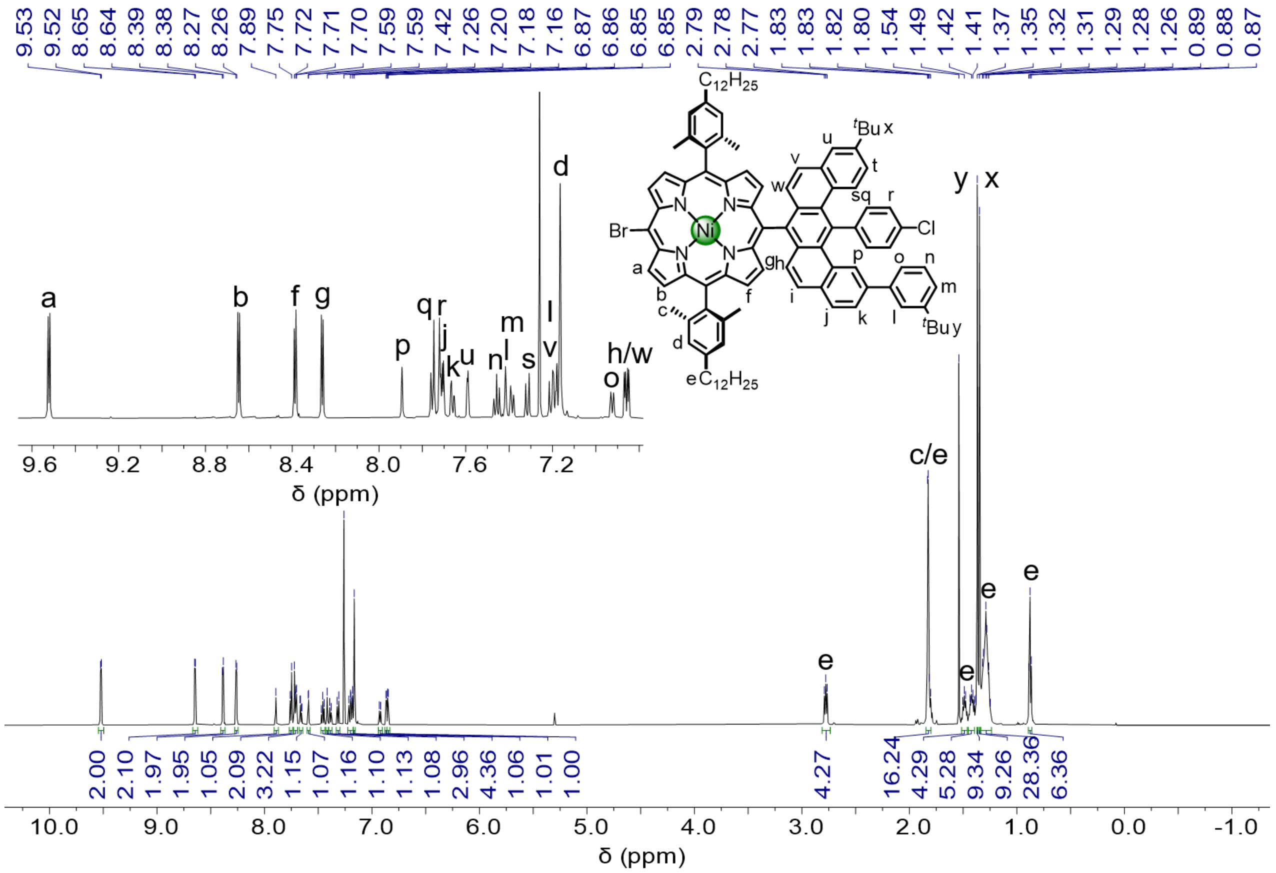

Supplementary Figure 74. ^{1}H NMR spectrum of meso-bromoporphyrin **24b** (600 MHz, $CDCl_3$, 298 K).

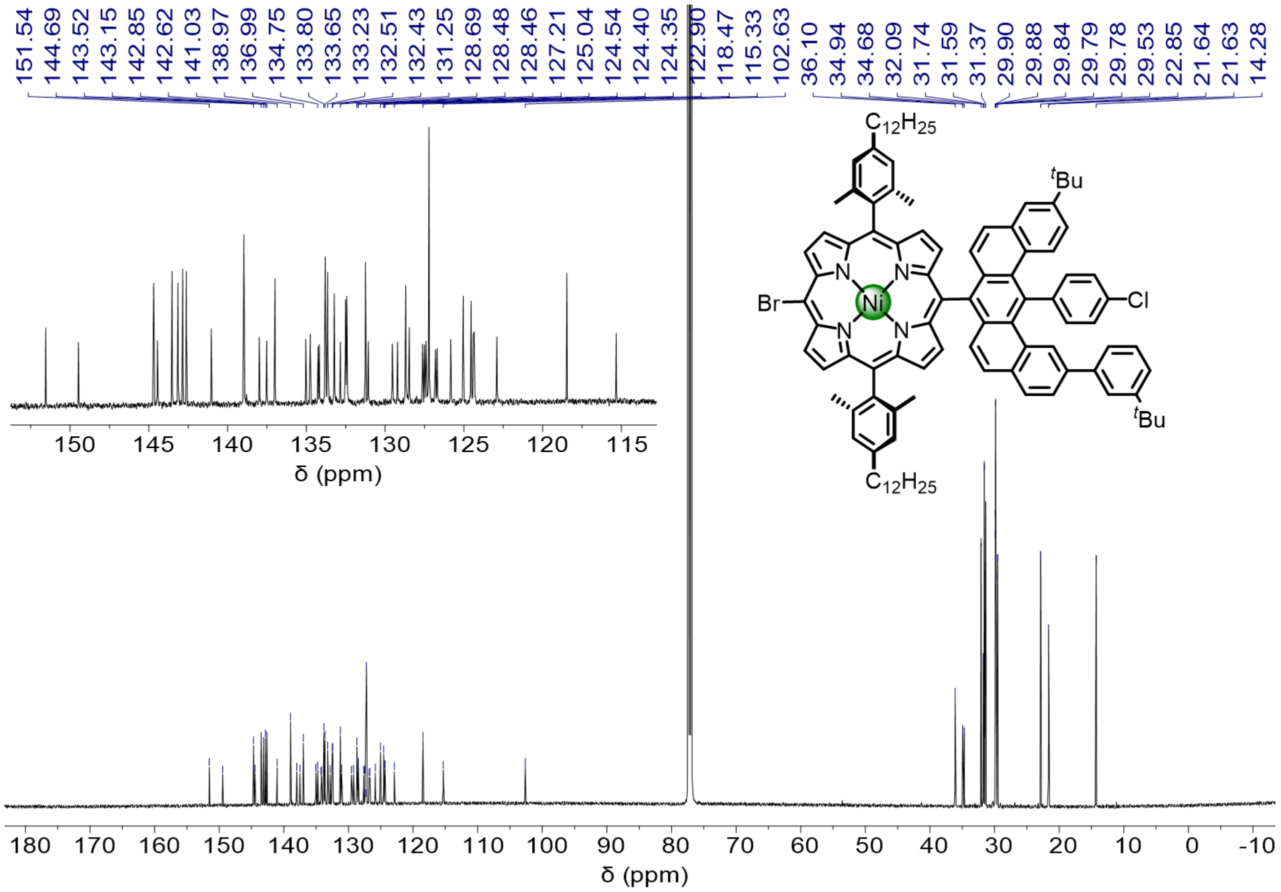

Supplementary Figure 75. ^{13}C NMR spectrum of meso-bromoporphyrin **24b** (150 MHz, $CDCl_3$, 298 K).

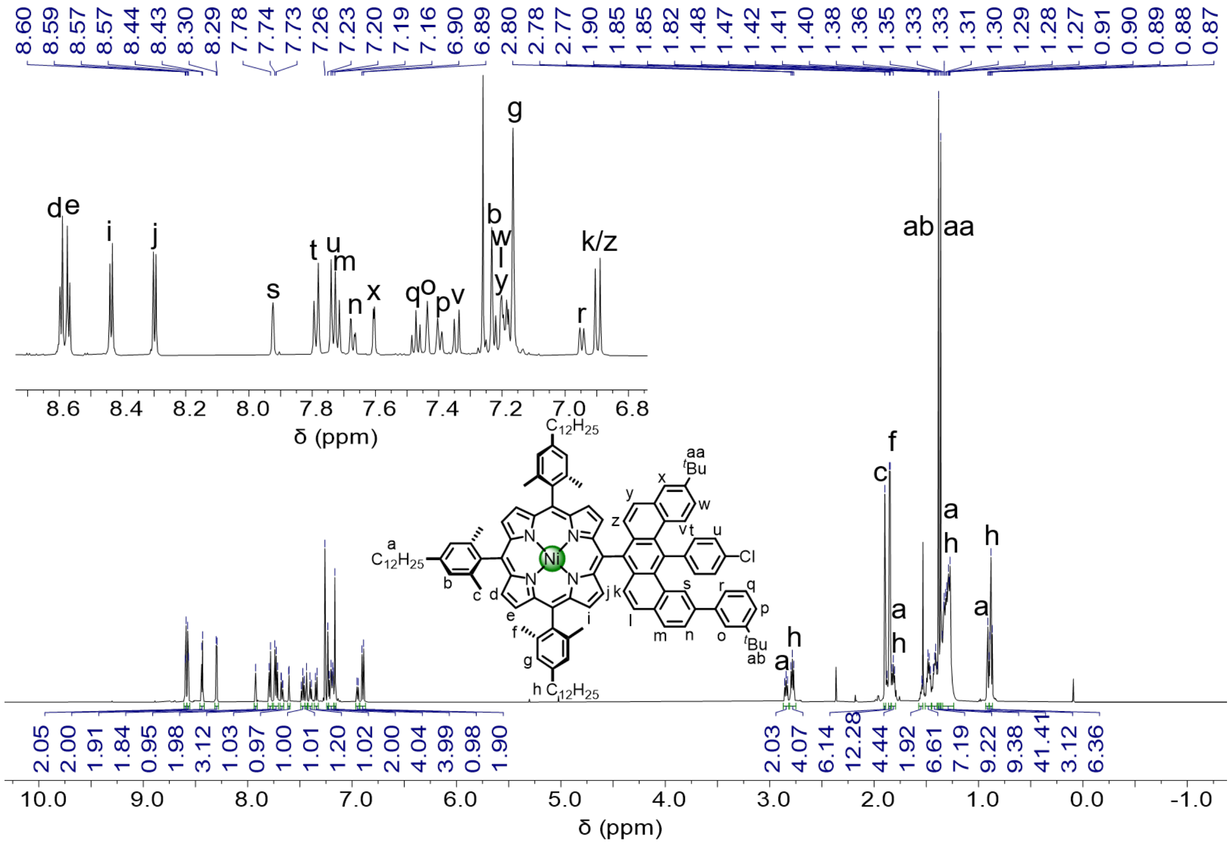

Supplementary Figure 76. ^{1}H NMR spectrum of porphyrin **1b** (600 MHz, $CDCl_3$, 298 K).

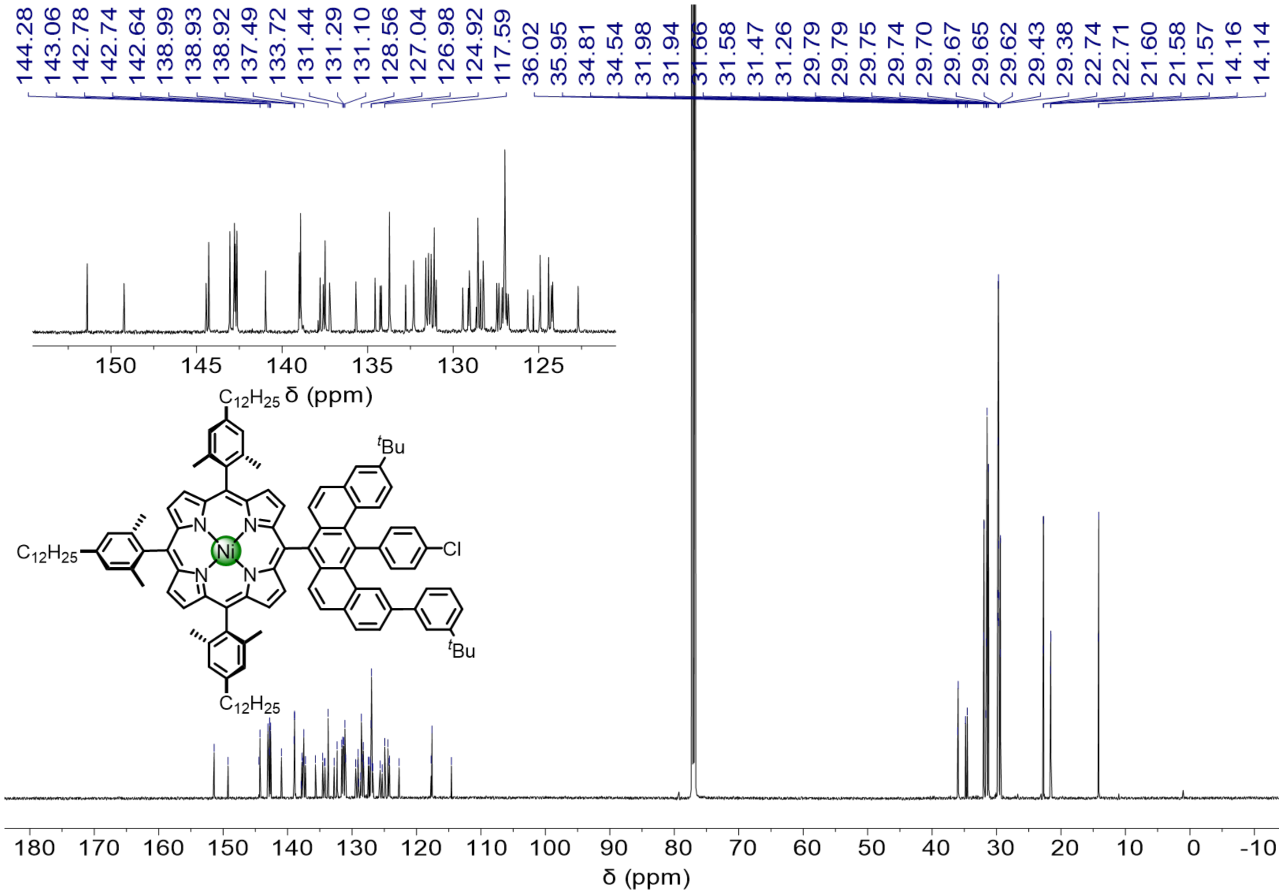

Supplementary Figure 77. ^{13}C NMR spectrum of porphyrin **1b** (150 MHz, $CDCl_3$, 298 K).

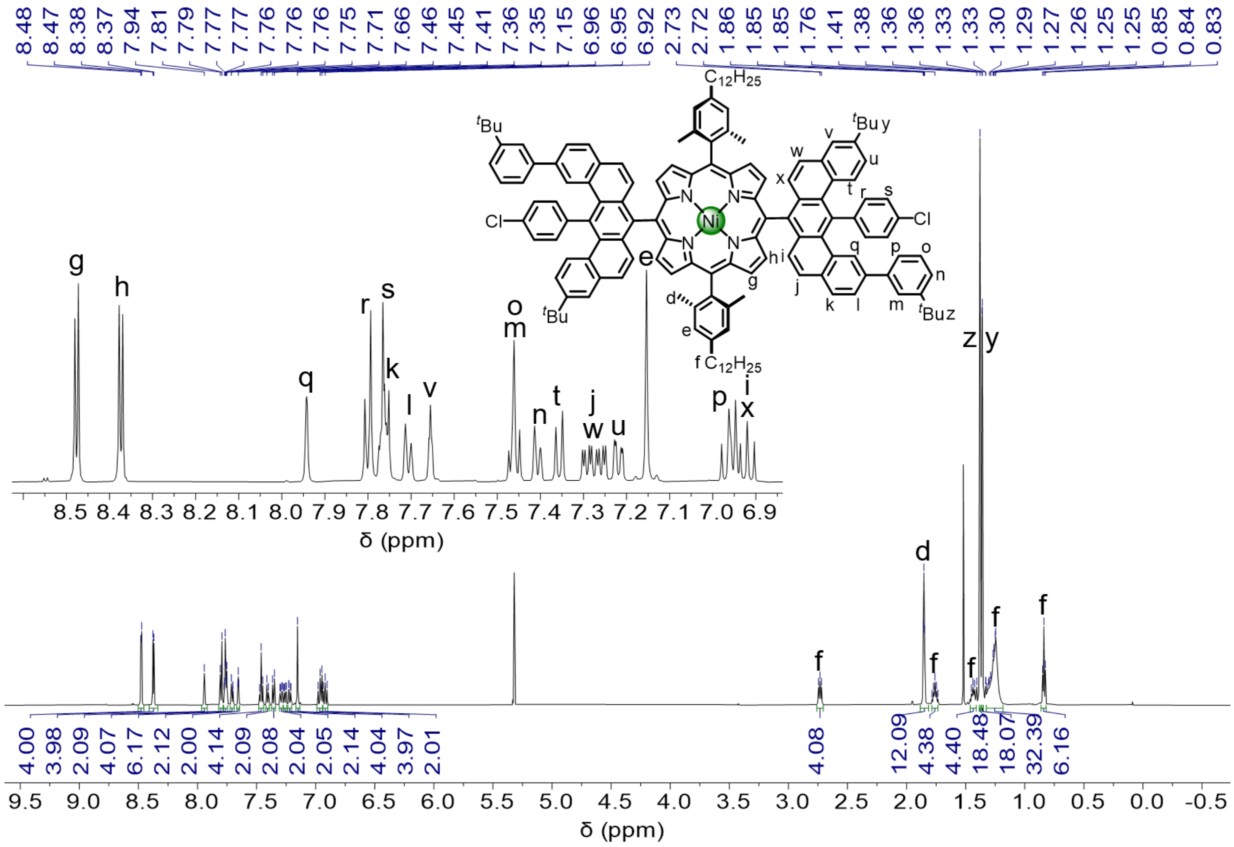

Supplementary Figure 78. ^{1}H NMR spectrum of porphyrin **2b** (600 MHz, CD_2Cl_2, 298 K).

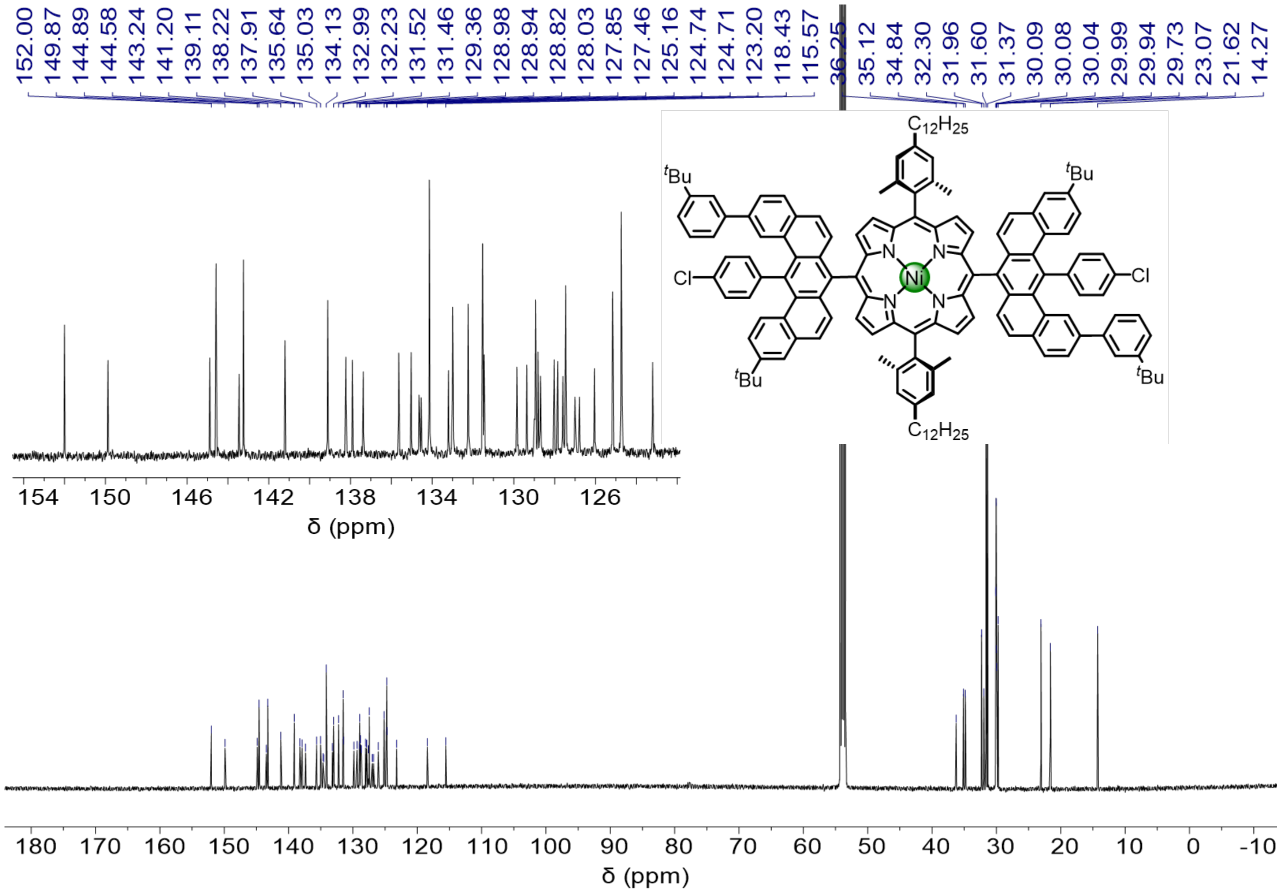

Supplementary Figure 79. ^{13}C NMR spectrum of porphyrin **2b** (150 MHz, CD_2Cl_2, 298 K).

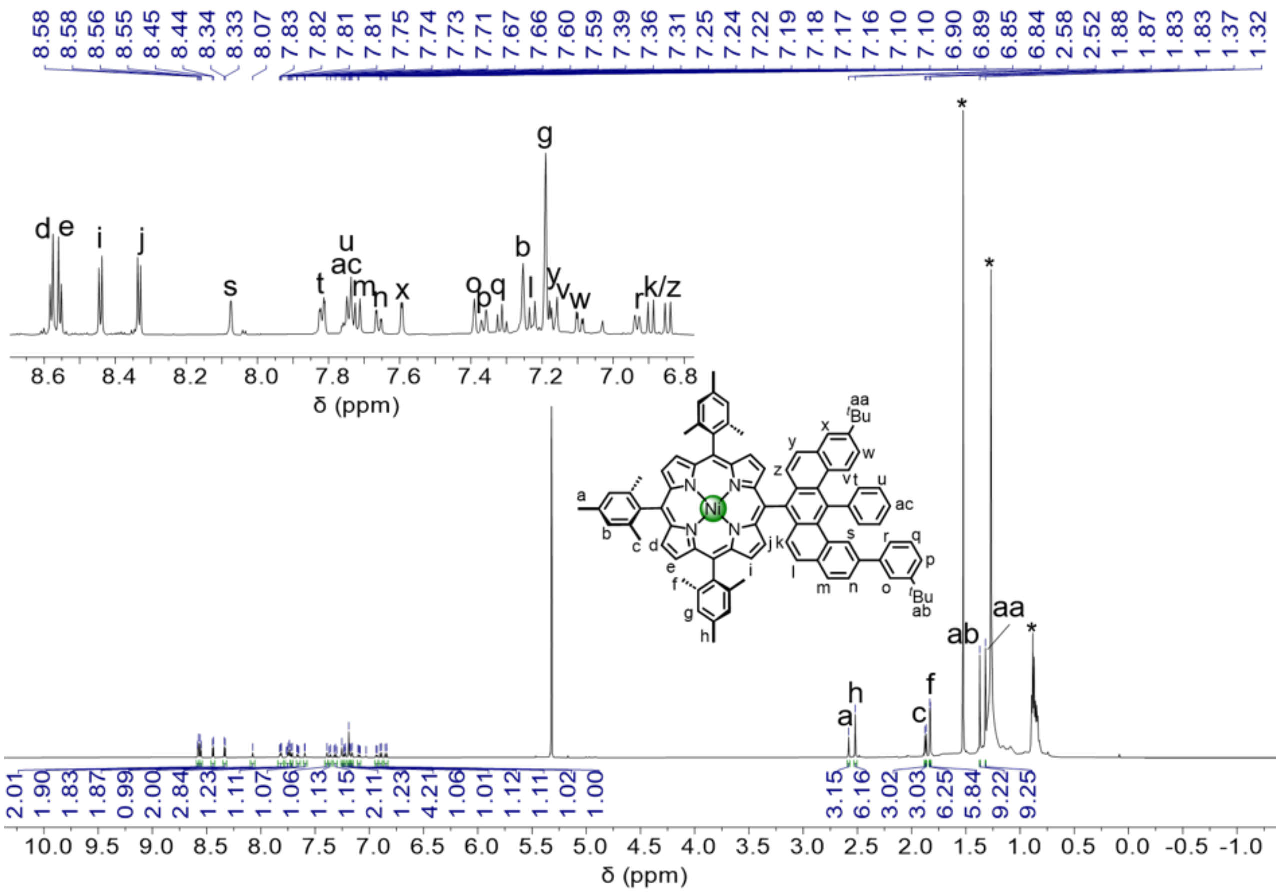

Supplementary Figure 80. ^{1}H NMR spectrum of trimesitylporphyrin **25a** (600 MHz, CD_2Cl_2, 298 K). * indicates water and H-grease peaks.

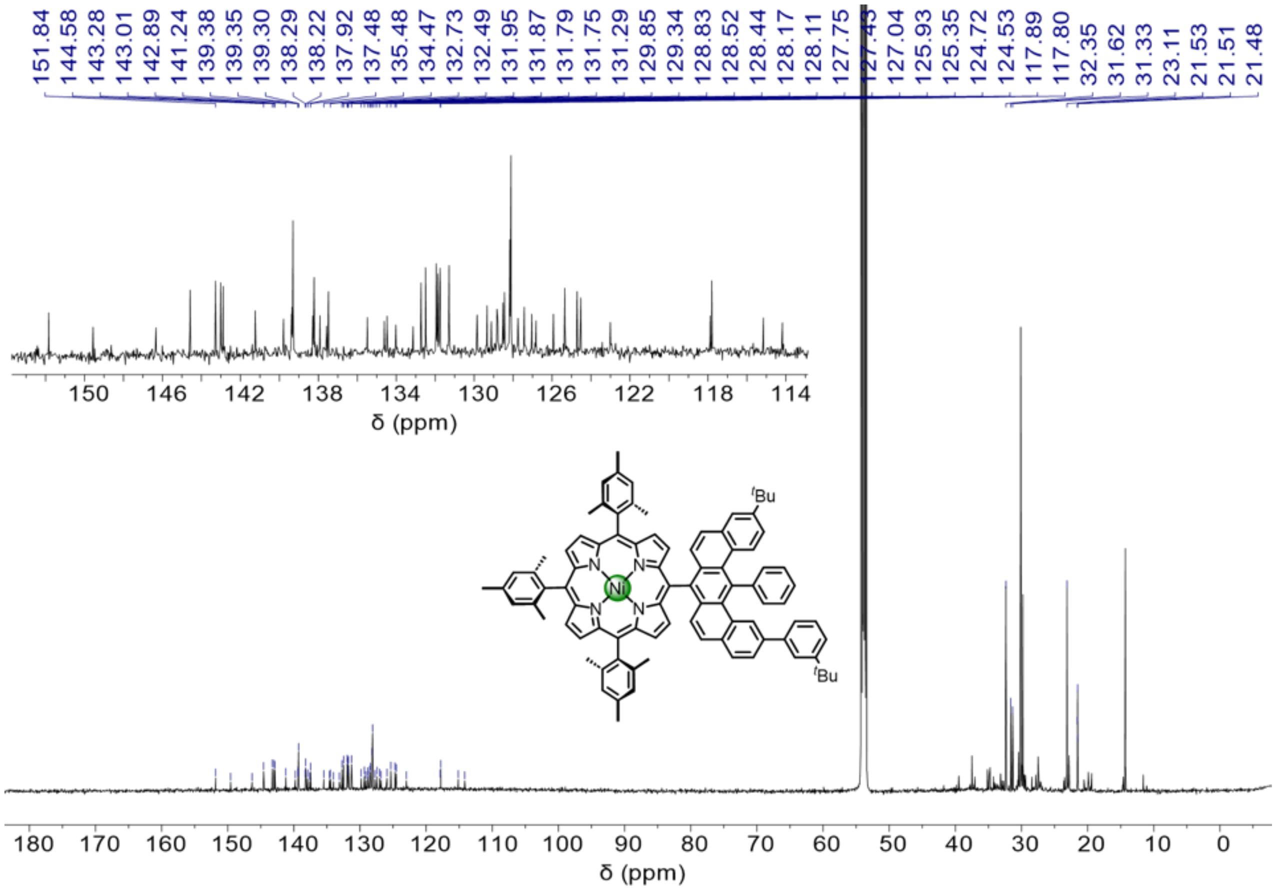

Supplementary Figure 81. ^{13}C NMR spectrum of trimesitylporphyrin **25a** (150 MHz, CD_2Cl_2, 298 K).

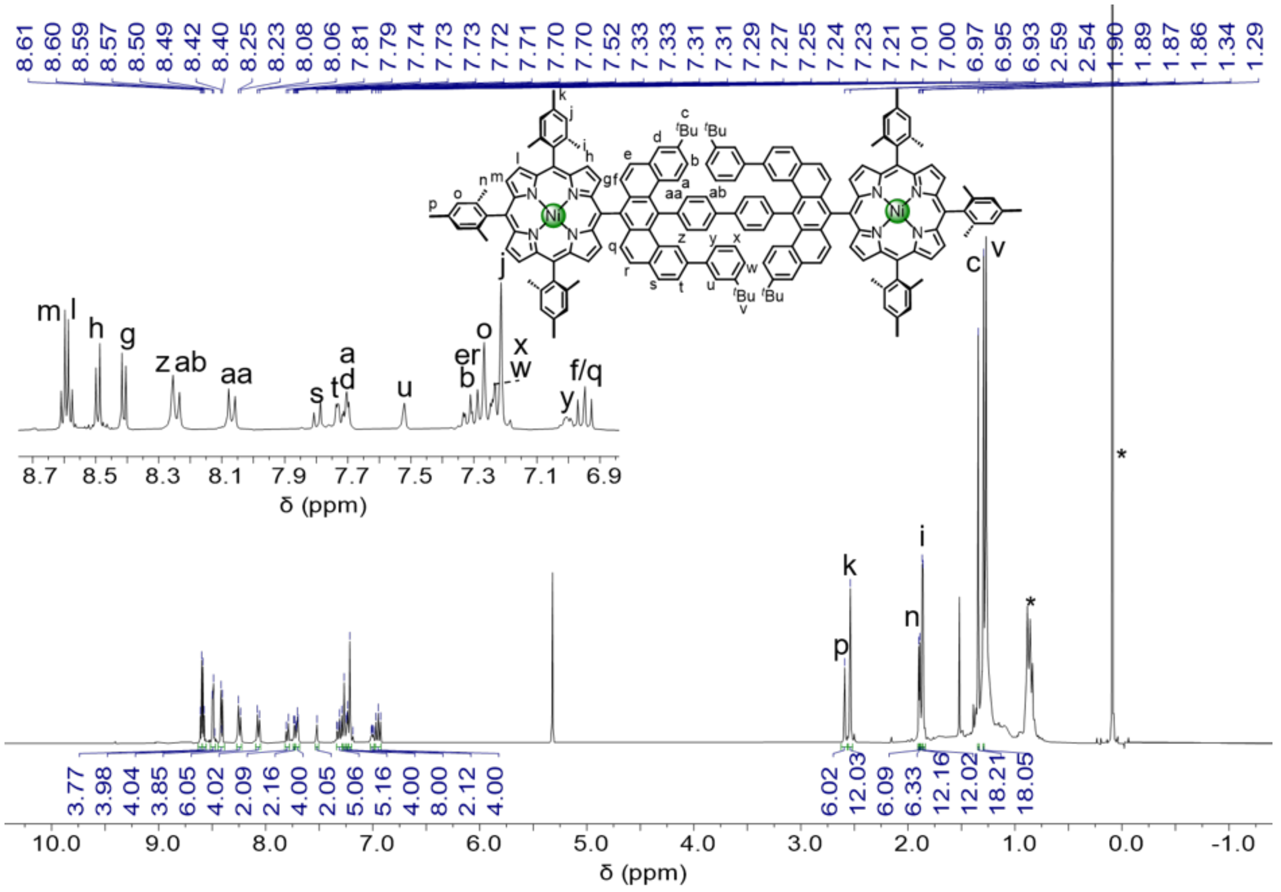

Supplementary Figure 82. ^{1}H NMR spectrum of trimesitylporphyrin **26a** (400 MHz, CD_2Cl_2, 298 K). * indicates grease peaks.

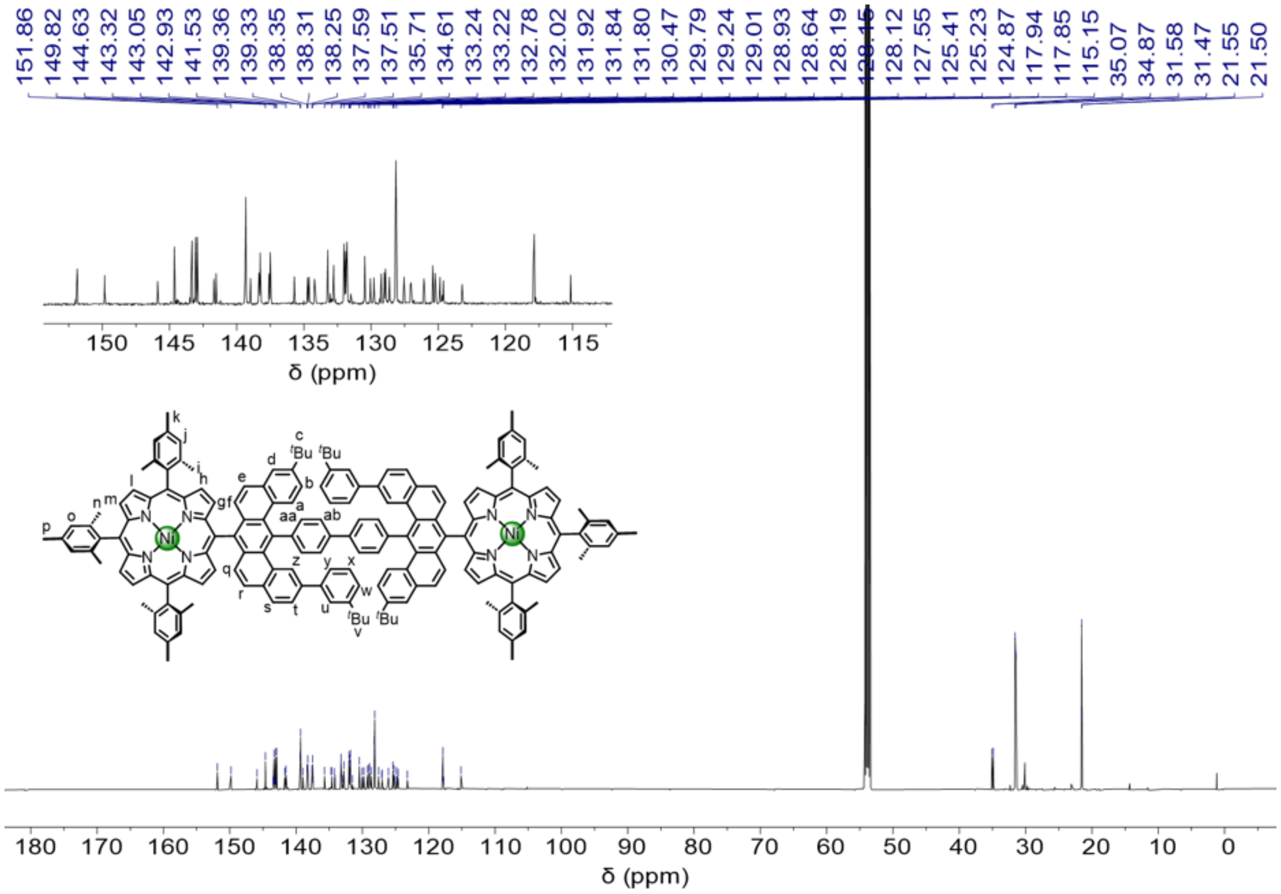

Supplementary Figure 83. ^{13}C NMR spectrum of trimesitylporphyrin **26a** (150 MHz, CD_2Cl_2, 298 K).

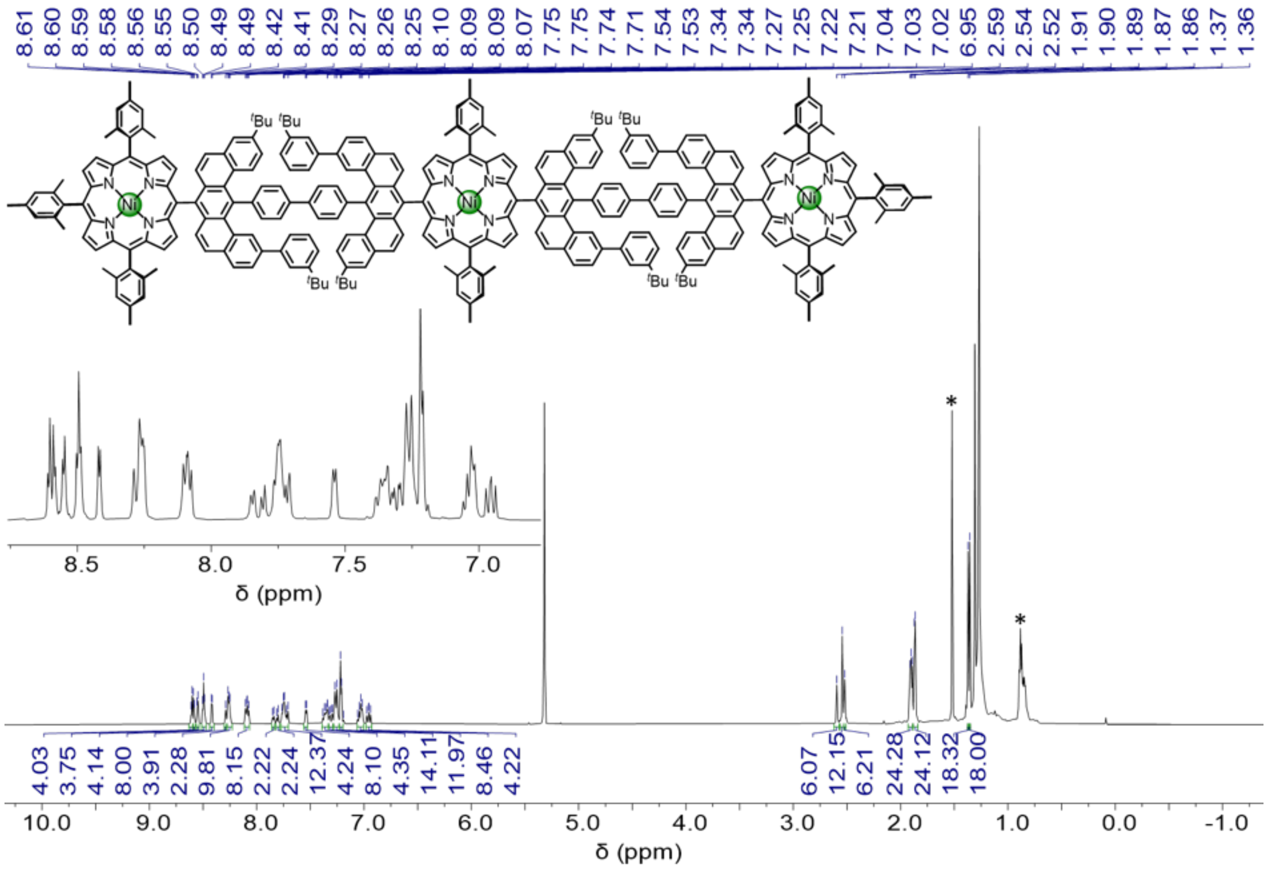

Supplementary Figure 84. ^{1}H NMR spectrum of trimesitylporphyrin **27a** (600 MHz, CD_2Cl_2, 298 K). * indicates water and grease peaks.

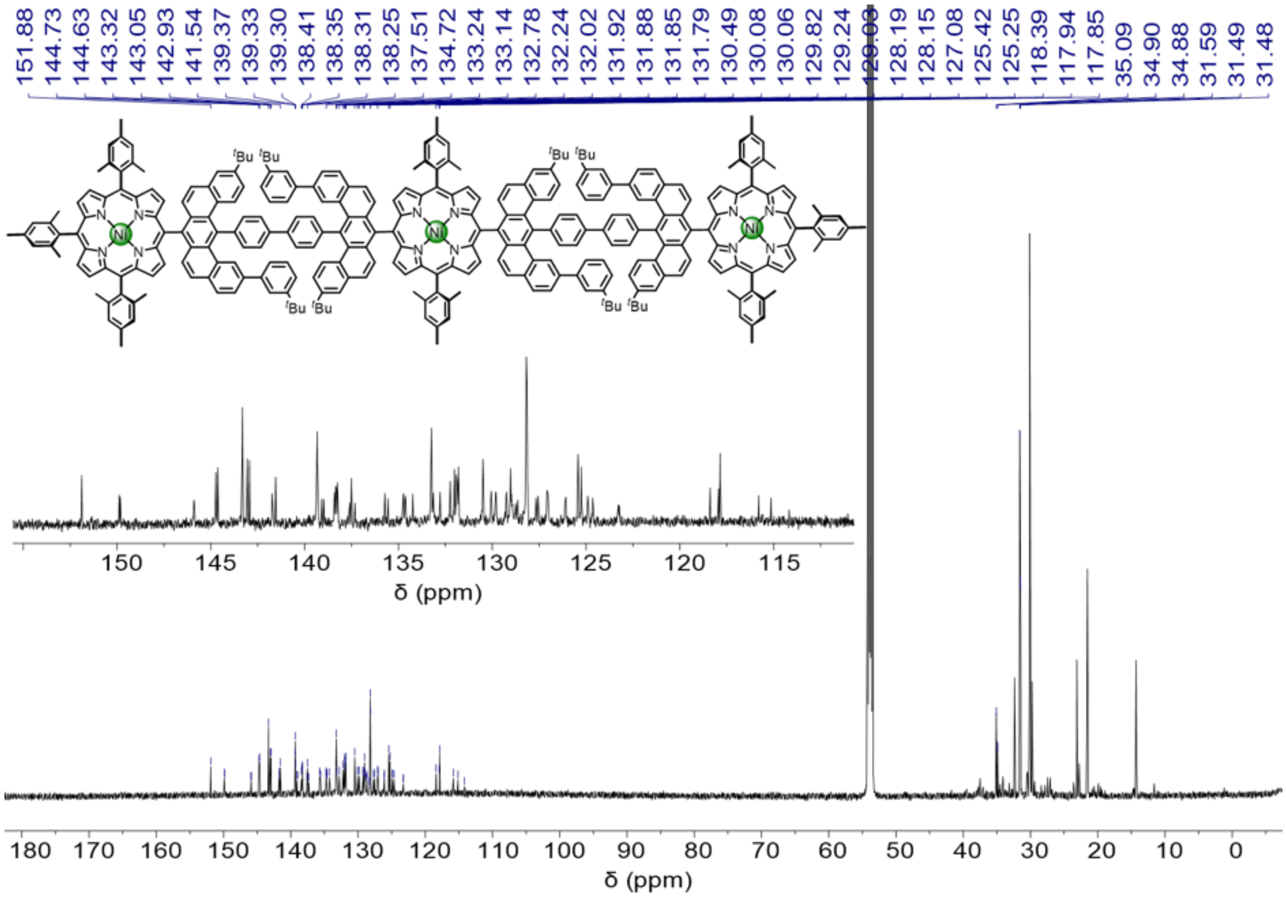

Supplementary Figure 85. ^{13}C NMR spectrum of trimesitylporphyrin **27a** (150 MHz, CD_2Cl_2, 298 K).

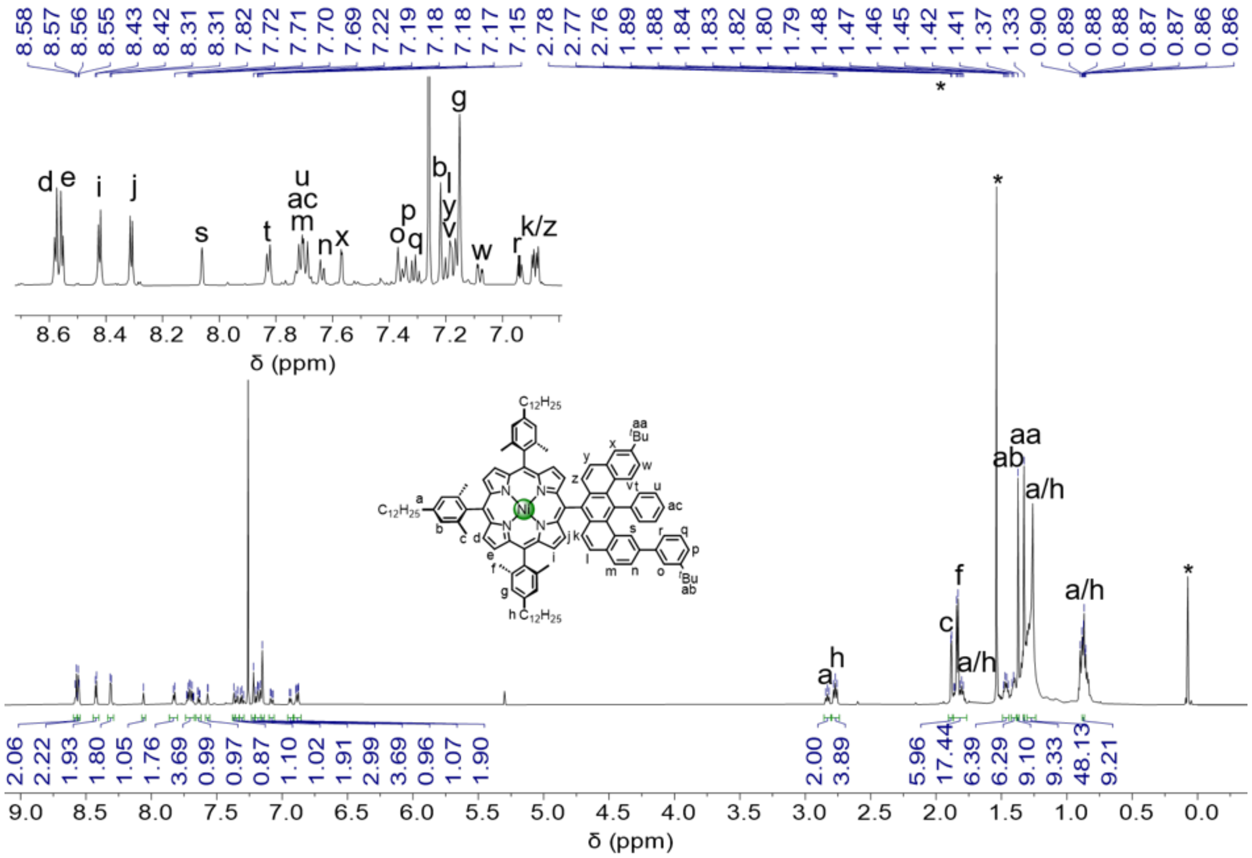

Supplementary Figure 86. ^{1}H NMR spectrum of triarylporphyrin **25b** (600 MHz, $CDCl_3$, 298 K). * indicates water and H-grease peaks.

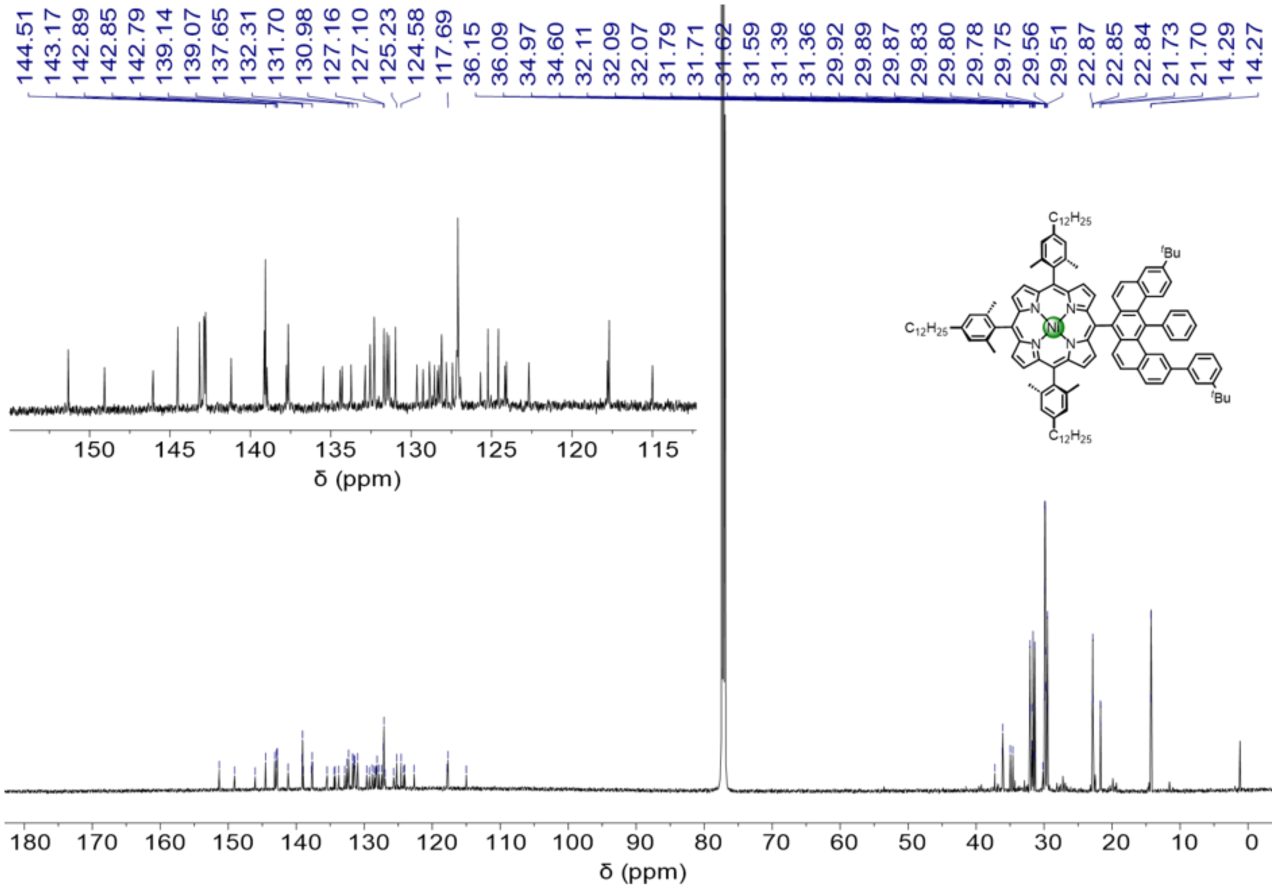

Supplementary Figure 87. ^{13}C NMR spectrum of triarylporphyrin **25b** (150 MHz, $CDCl_3$, 298 K).

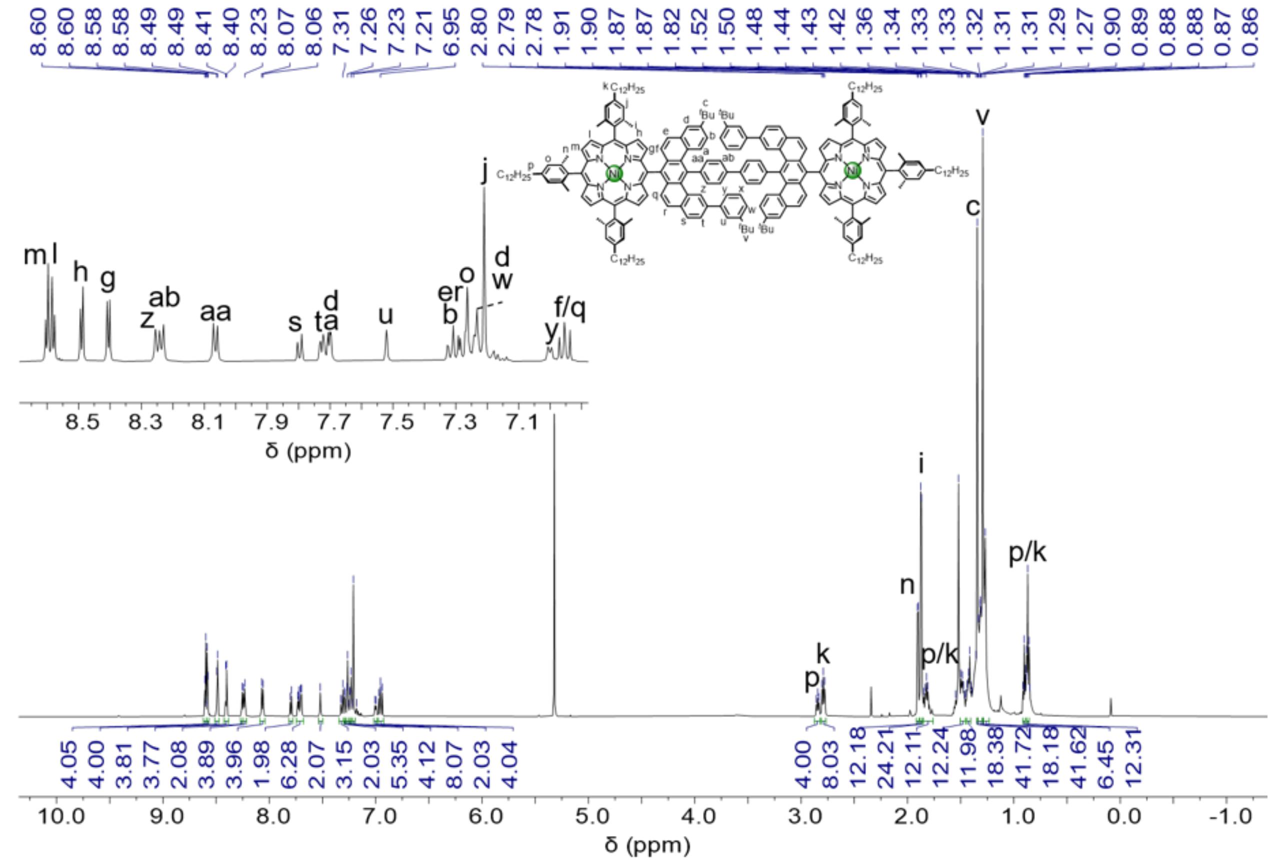

Supplementary Figure 88. ^{1}H NMR spectrum of porphyrin dimer **26b** (600 MHz, CD_2Cl_2, 298 K).

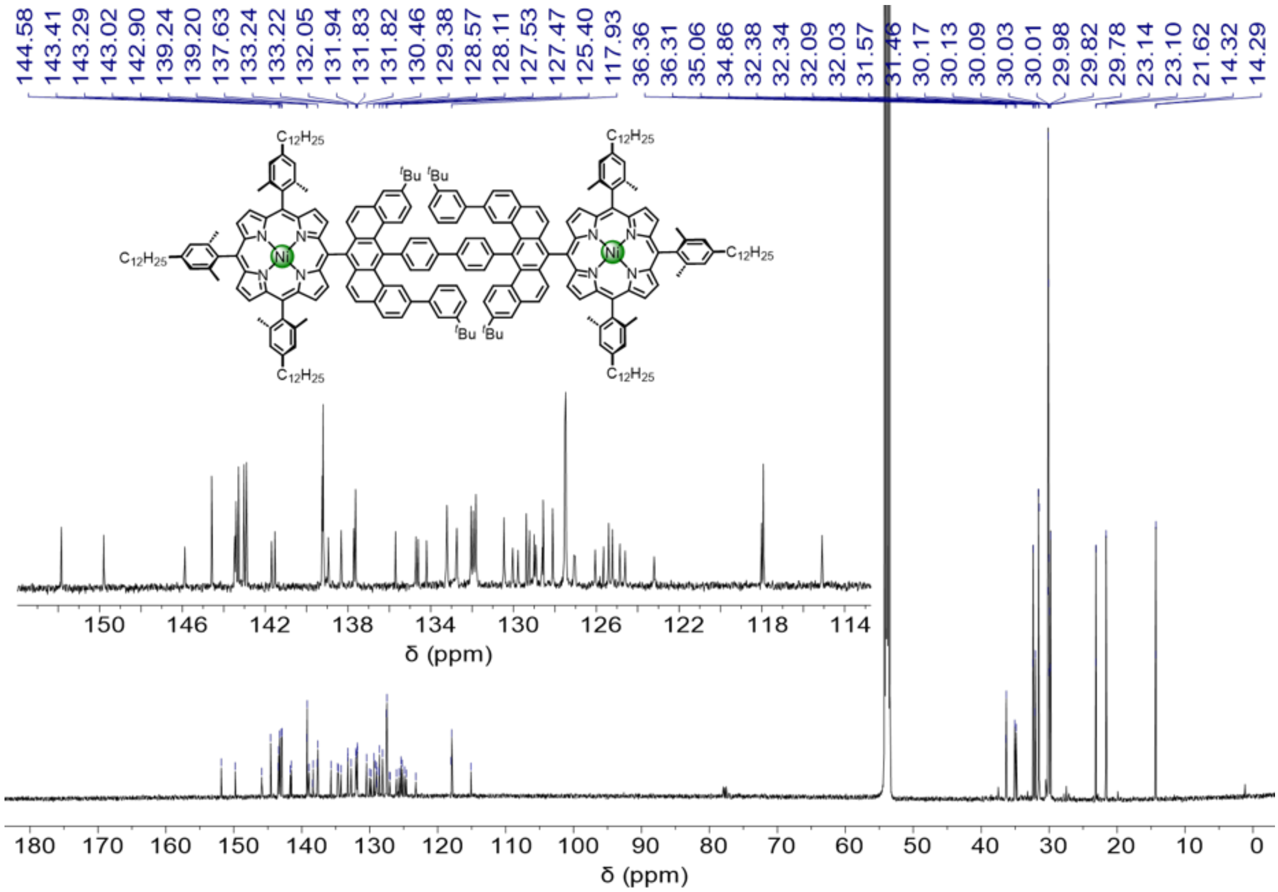

Supplementary Figure 89. ^{13}C NMR spectrum of porphyrin dimer **26b** (150 MHz, CD_2Cl_2, 298 K).

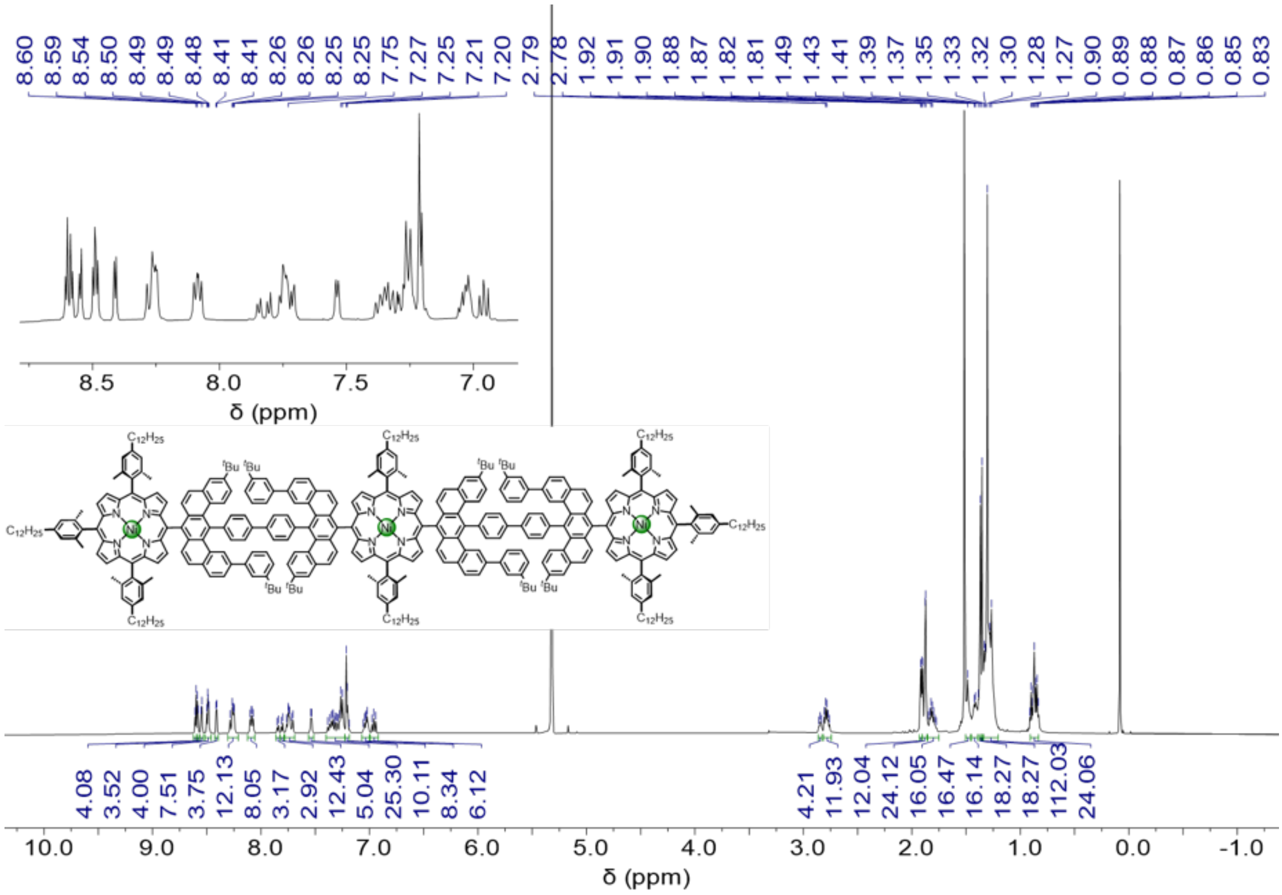

Supplementary Figure 90. ^{1}H NMR spectrum of porphyrin trimer **27b** (600 MHz, CD_2Cl_2, 298 K).

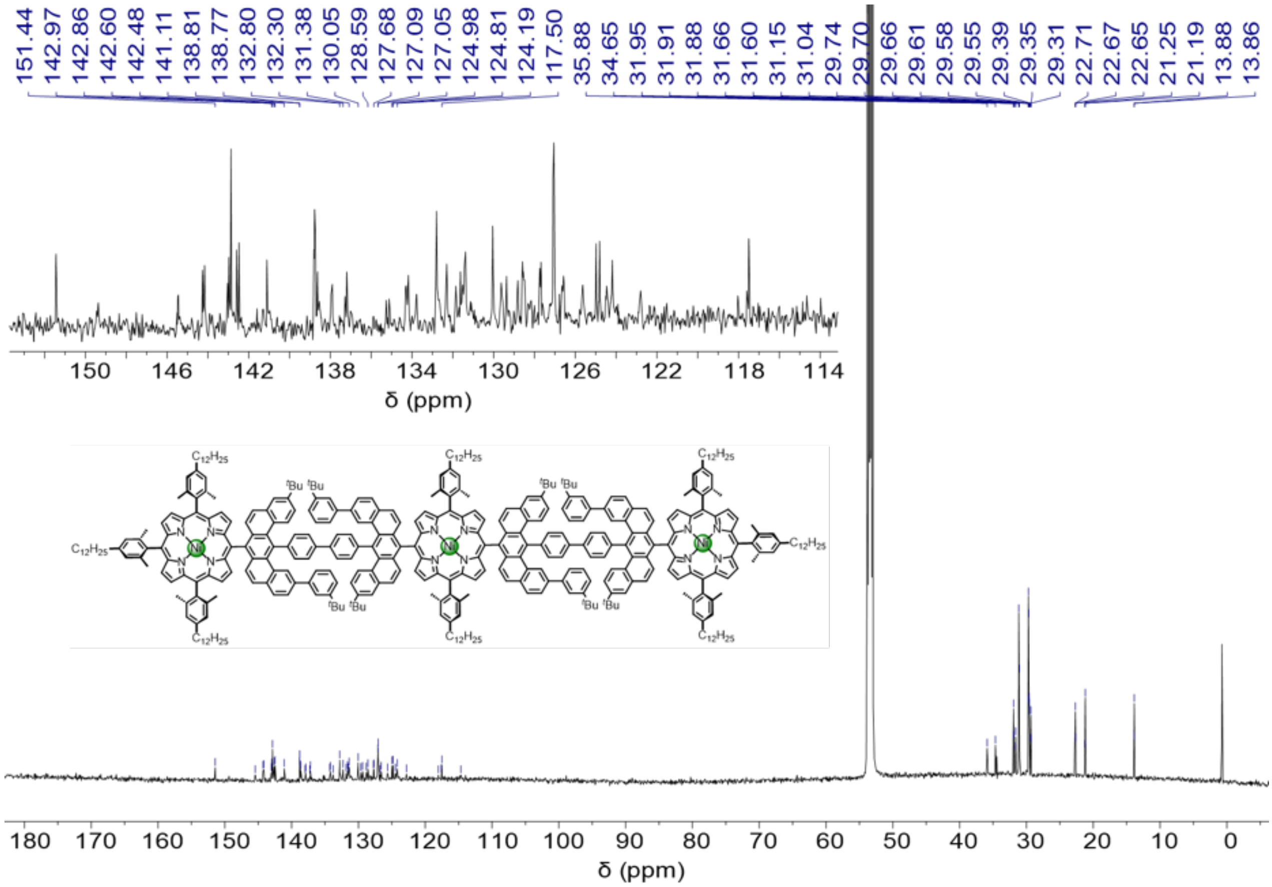

Supplementary Figure 91. ^{13}C NMR spectrum of porphyrin trimer **27b** (150 MHz, CD_2Cl_2, 298 K).

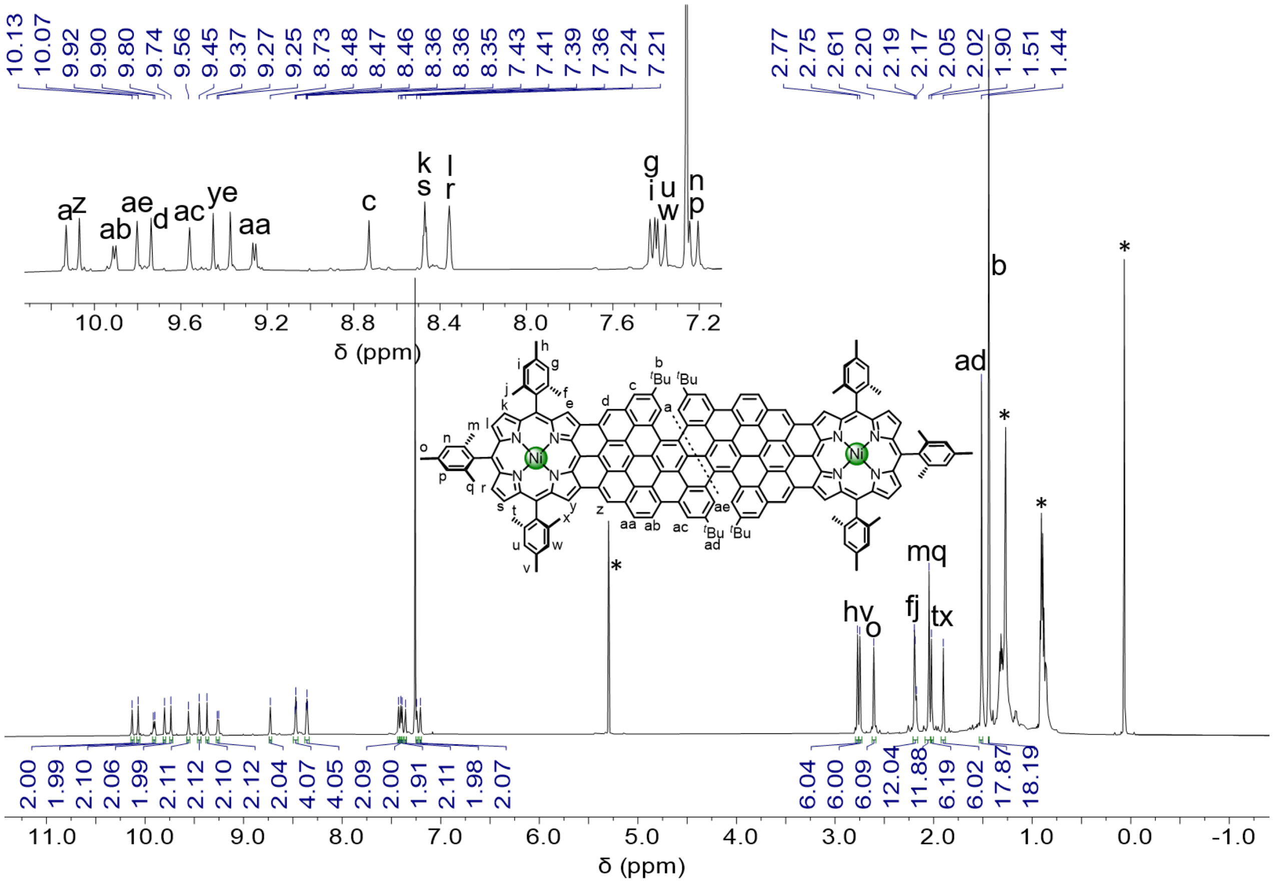

Supplementary Figure 92. ^{1}H NMR spectrum of ***f*-P2Ng1a** (600 MHz, $CDCl_3$:CS_2 = 1:1, 298 K). (*) indicates DCM and H-grease.

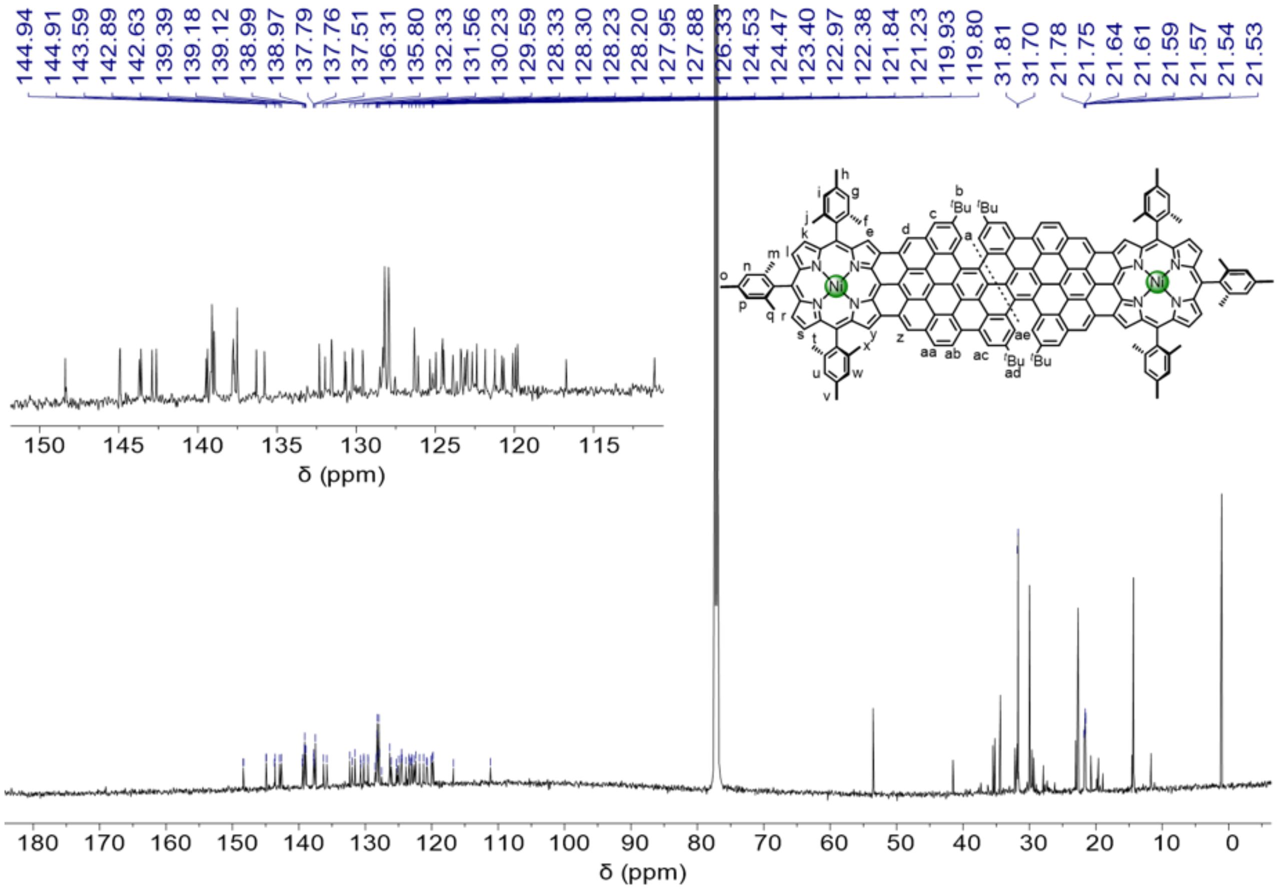

Supplementary Figure 93. ^{13}C NMR spectrum of ***f*-P2Ng1a** (150 MHz, $CDCl_3$:CS_2 = 1:1, 298 K).

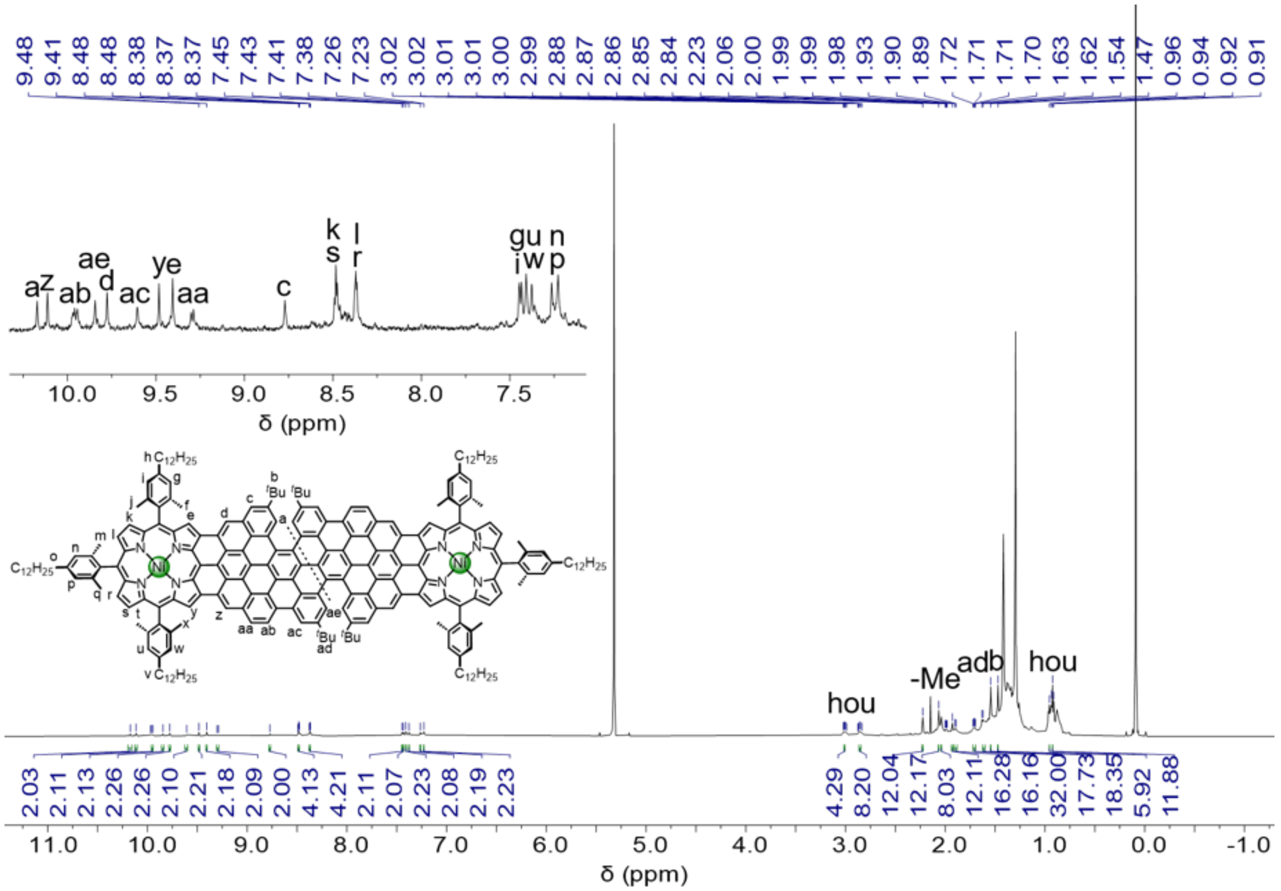

Supplementary Figure 94. ^{1}H NMR spectrum of ***f*-P2Ng1b** (600 MHz, CD_2Cl_2:CS_2 = 1:1, 298 K).

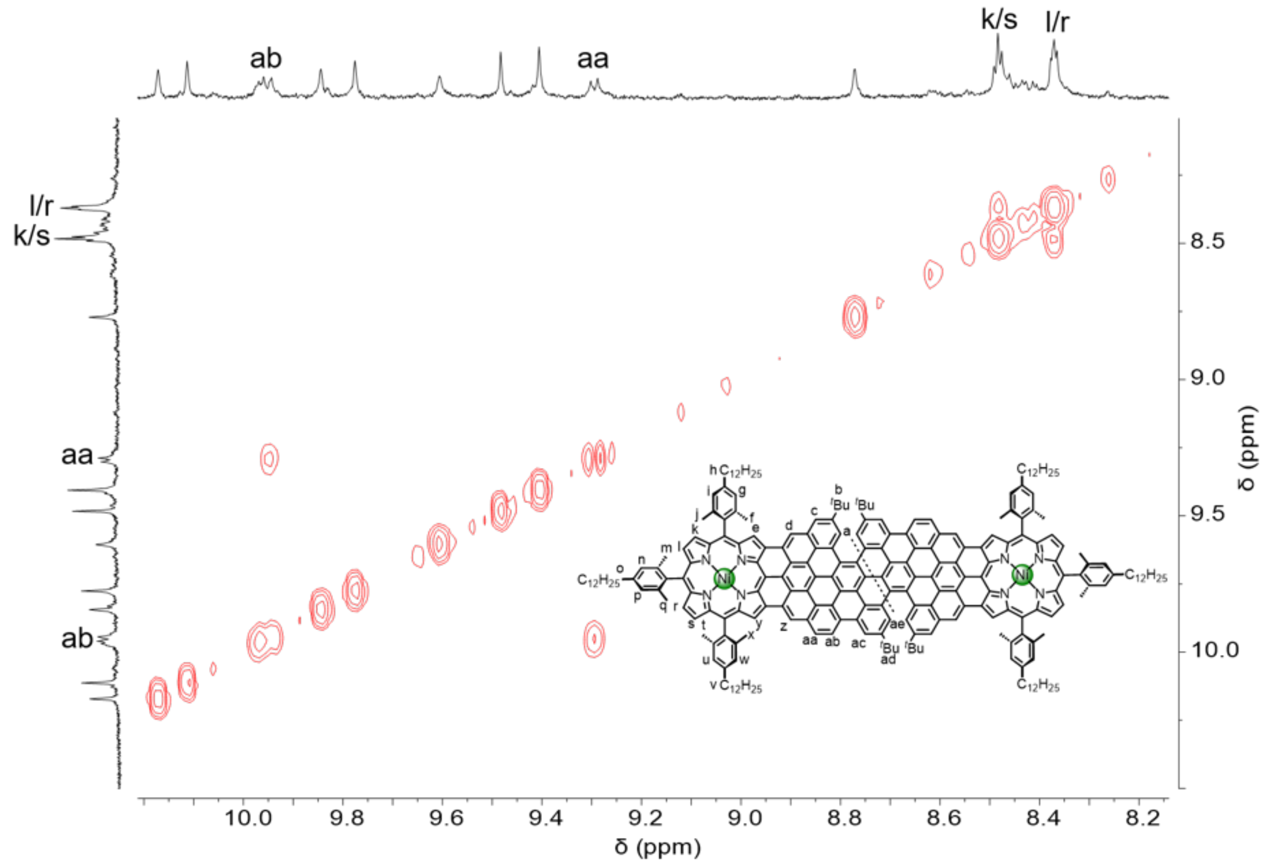

Supplementary Figure 95. Aromatic region of the ^{1}H,^{1}H-COSY spectrum of ***f*-P2Ng1b** (600 MHz, CD_2Cl_2:CS_2 = 1:1, 298 K).

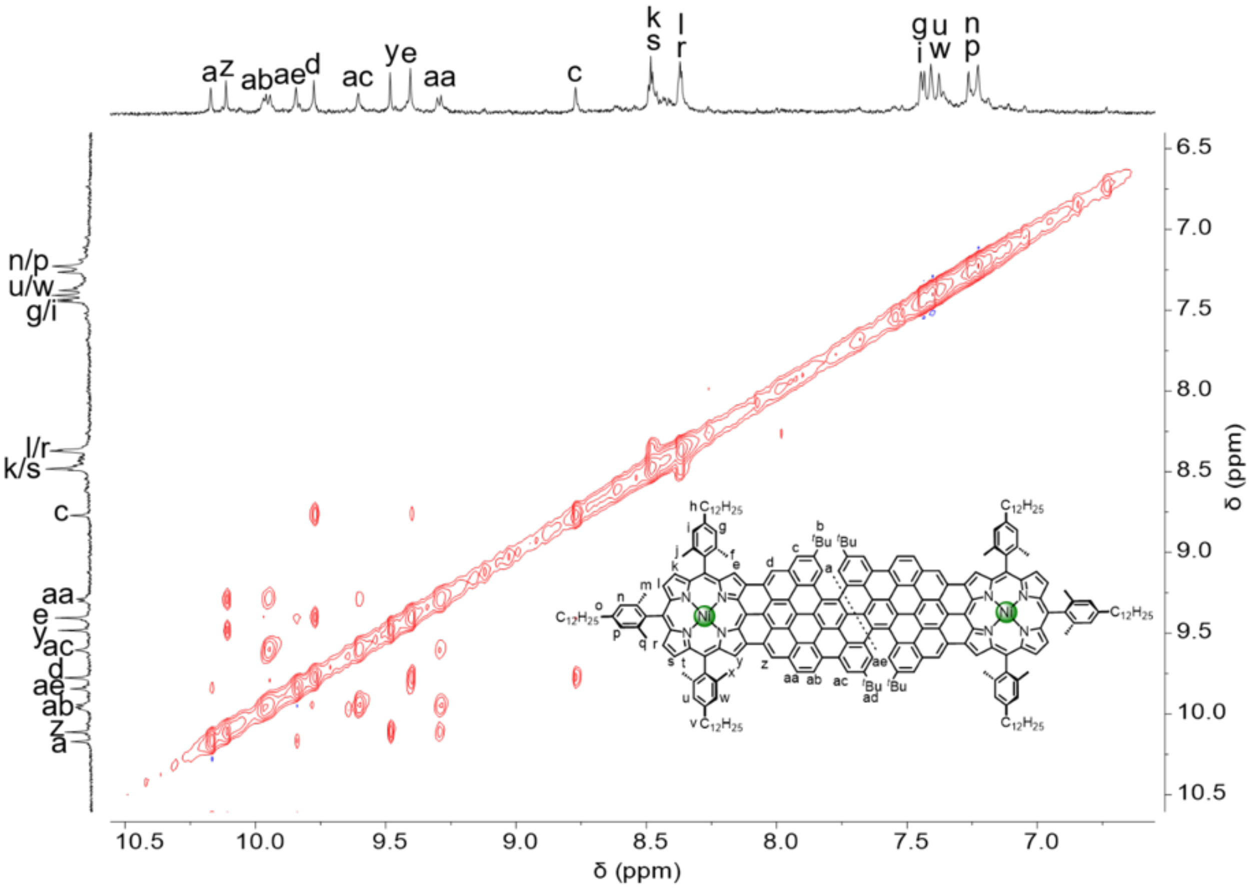

Supplementary Figure 96. Aromatic region of the ^{1}H,^{1}H-NOESY spectrum of ***f*-P2Ng1b** (600 MHz, CD_2Cl_2:CS_2 = 1:1, 298 K).

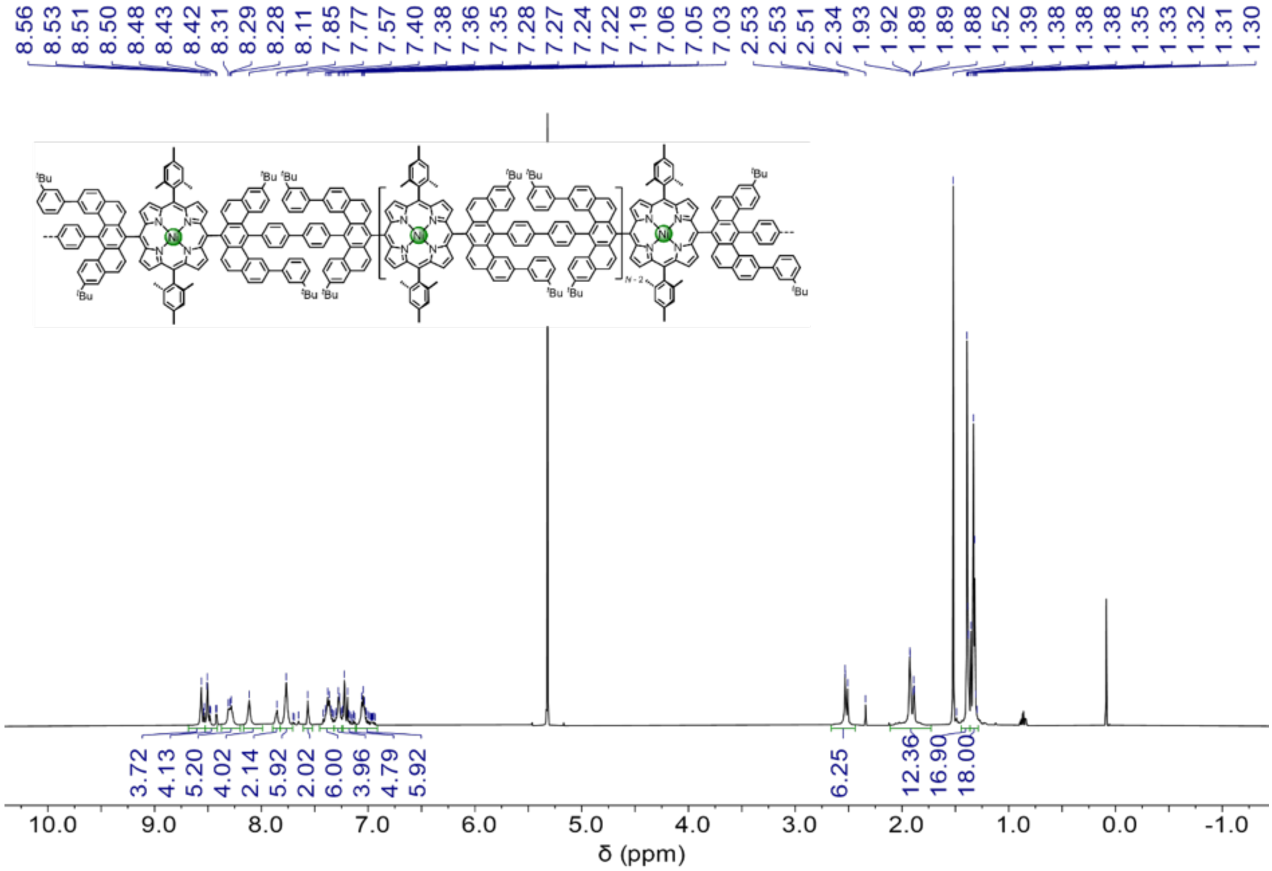

Supplementary Figure 97. ^{1}H NMR spectrum of **PPa** (600 MHz, CD_2Cl_2, 298 K).

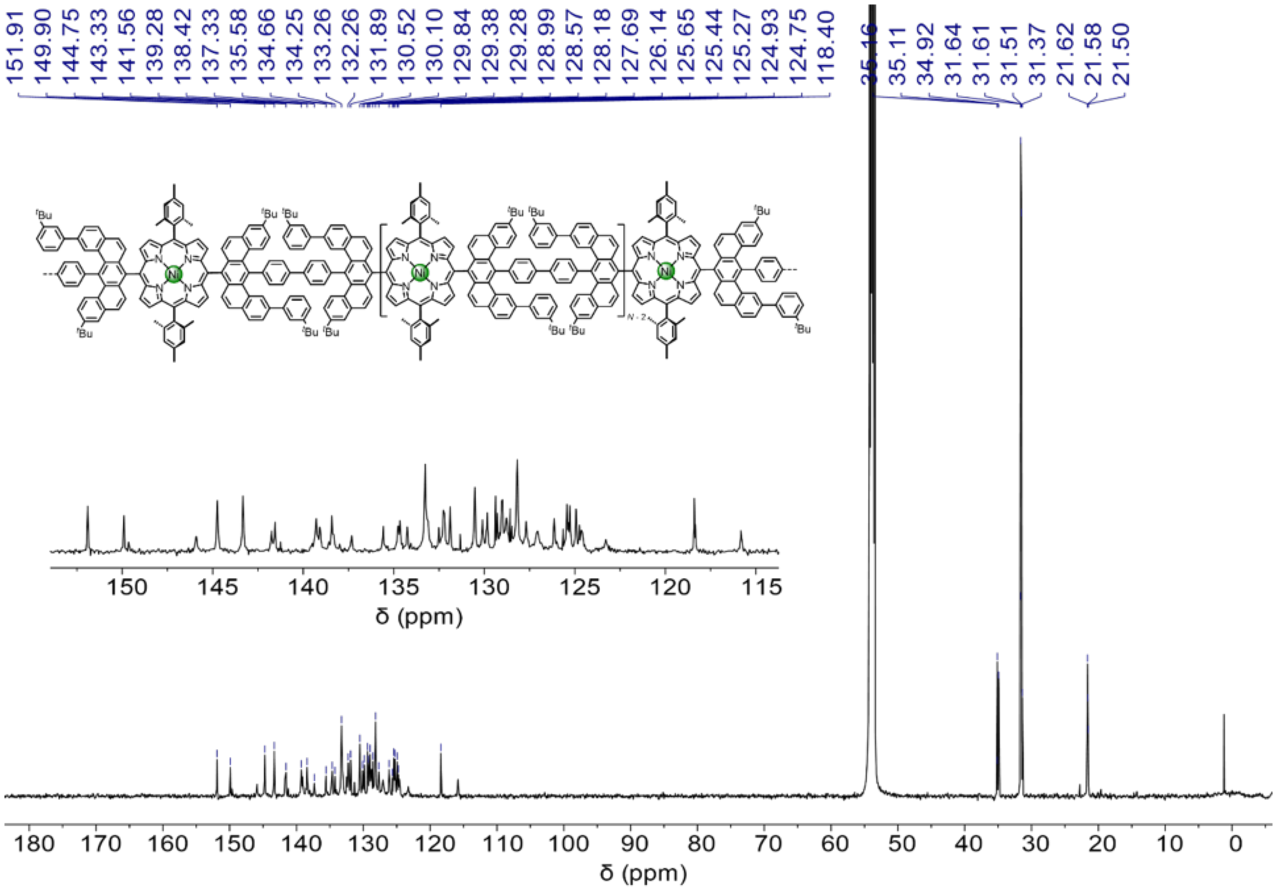

Supplementary Figure 98. ^{13}C NMR spectrum of **PPa** (150 MHz, CD_2Cl_2, 298 K).

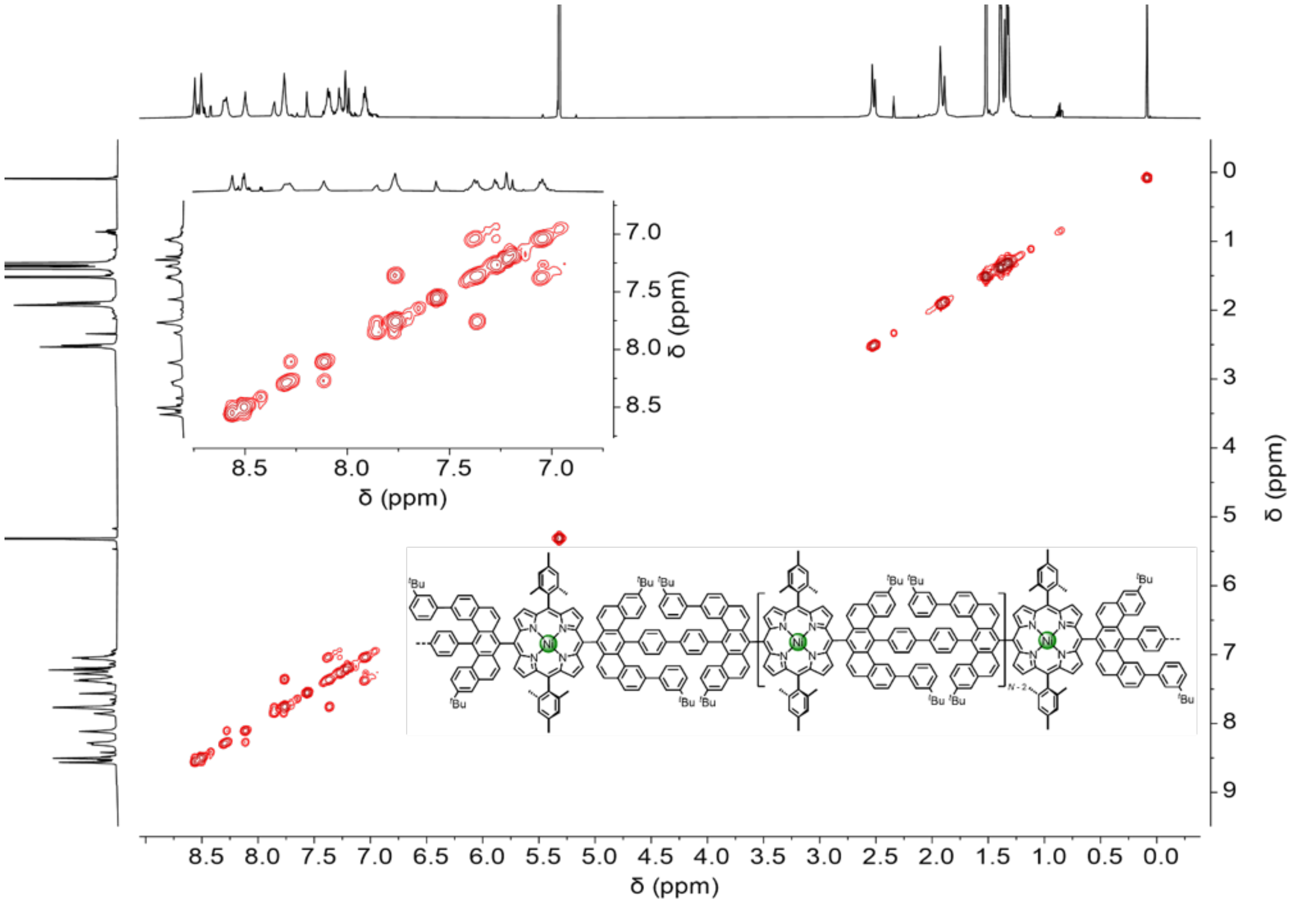

Supplementary Figure 99. ^{1}H,^{1}H-COSY spectrum of **PPa** (600 MHz, CD_2Cl_2, 298 K).

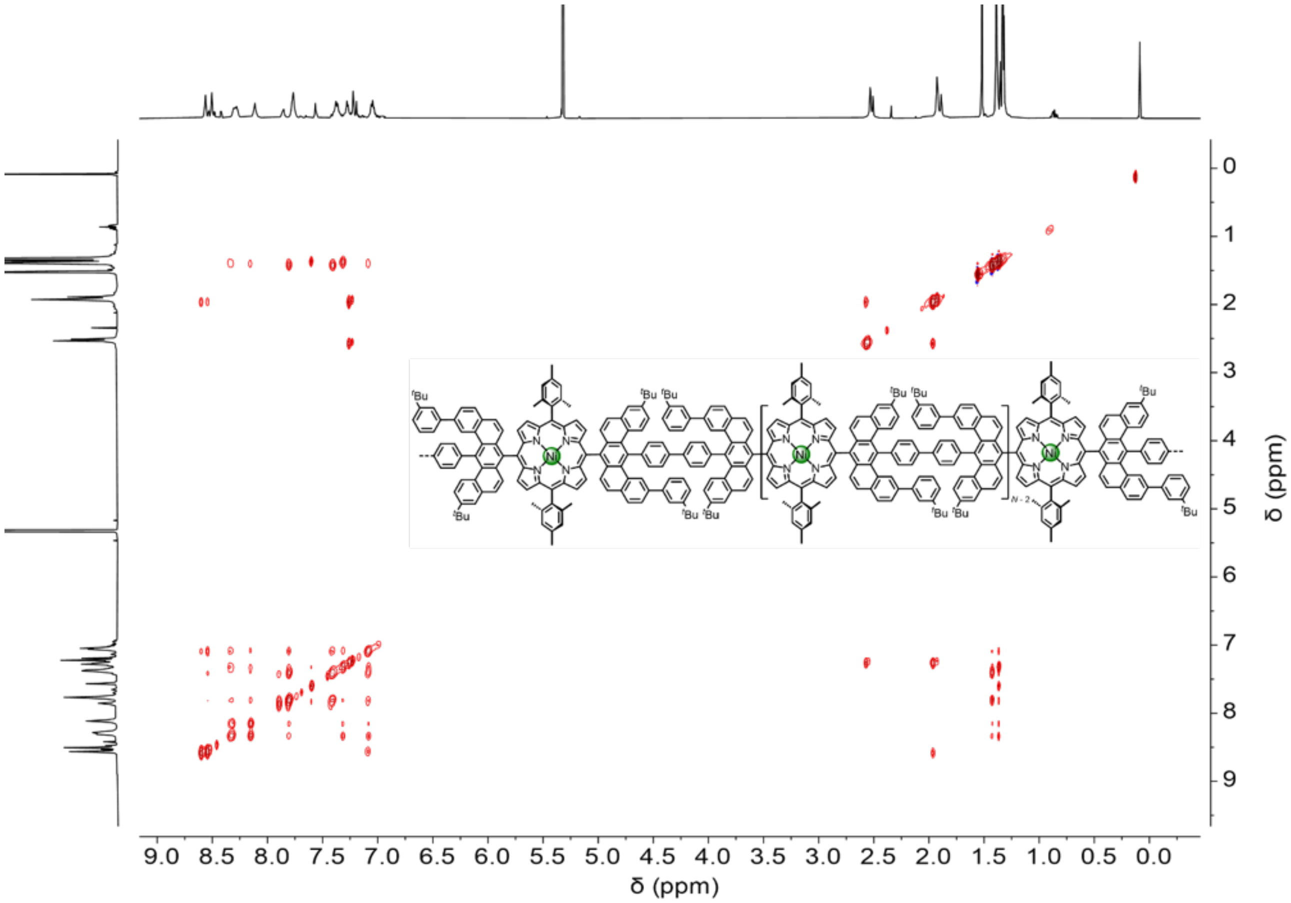

Supplementary Figure 100. $^{1}H,^{1}H$-NOESY spectrum of **PPa** (600 MHz, CD_2Cl_2, 298 K).

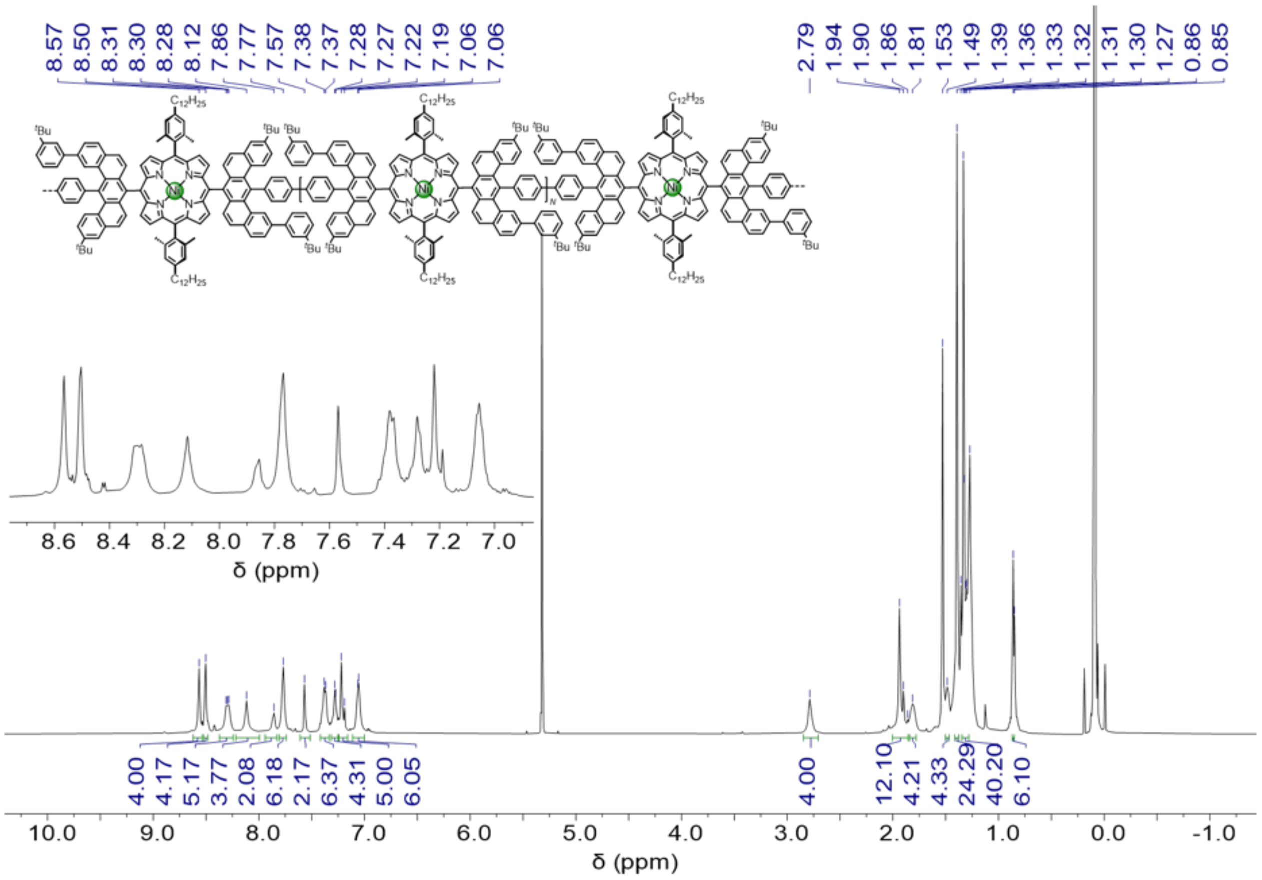

Supplementary Figure 101. ^{1}H NMR spectrum of **PPb** (600 MHz, CD_2Cl_2, 298 K).

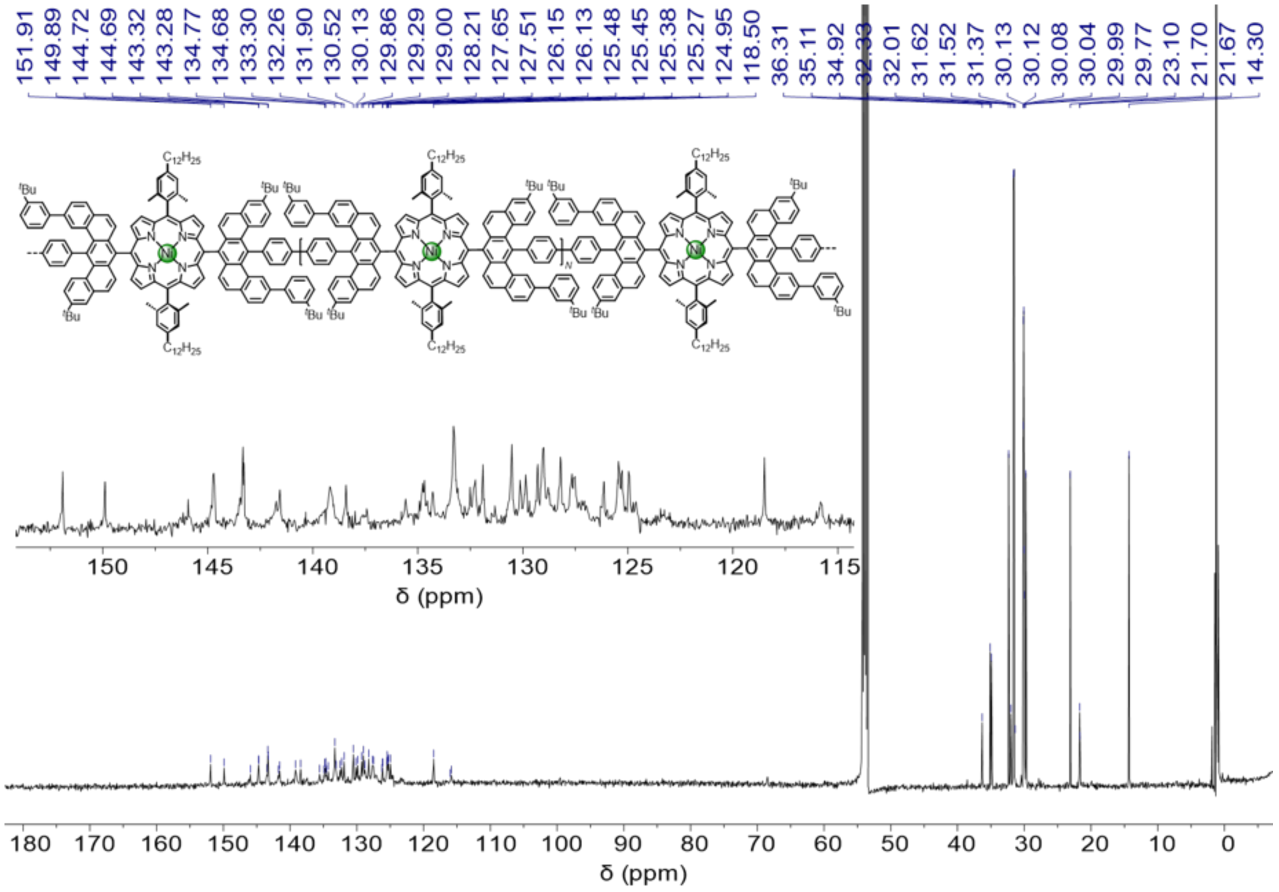

Supplementary Figure 102. ^{13}C NMR spectrum of **PPb** (150 MHz, CD_2Cl_2, 298 K).

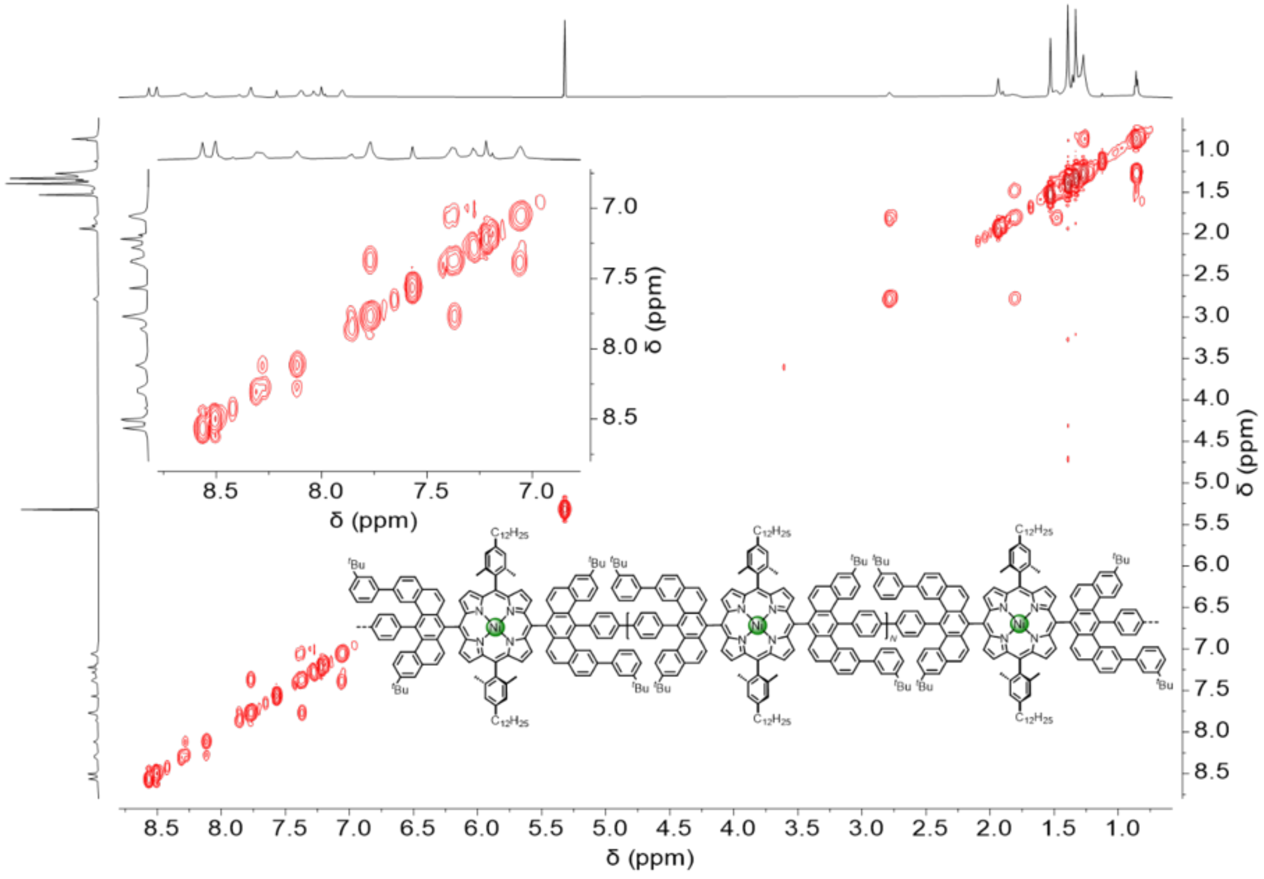

Supplementary Figure 103. ^{1}H,^{1}H-COSY spectrum of **PPb** (600 MHz, CD_2Cl_2, 298 K).

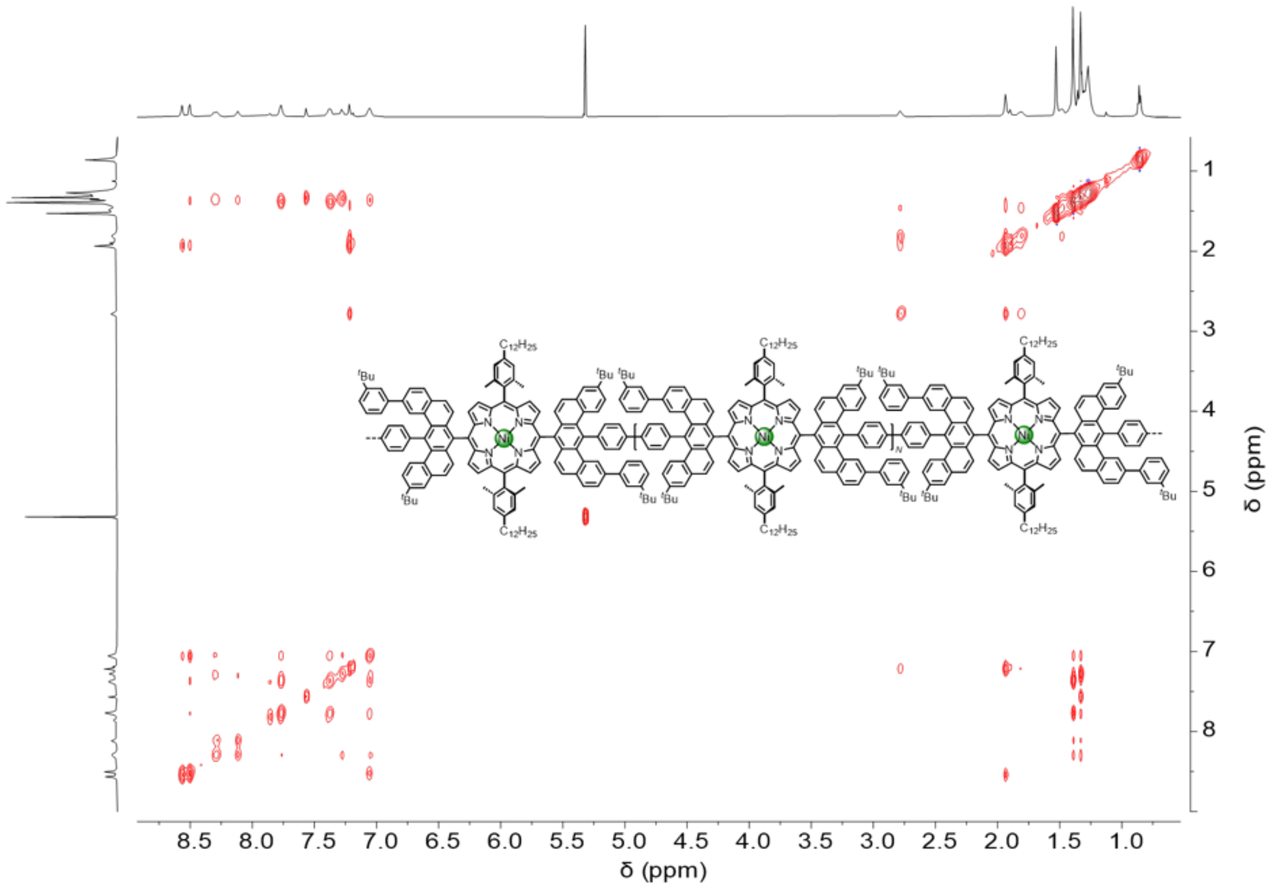

Supplementary Figure 104. $^{1}H,^{1}H$-NOESY spectrum of **PPb** (600 MHz, CD_2Cl_2, 298 K).

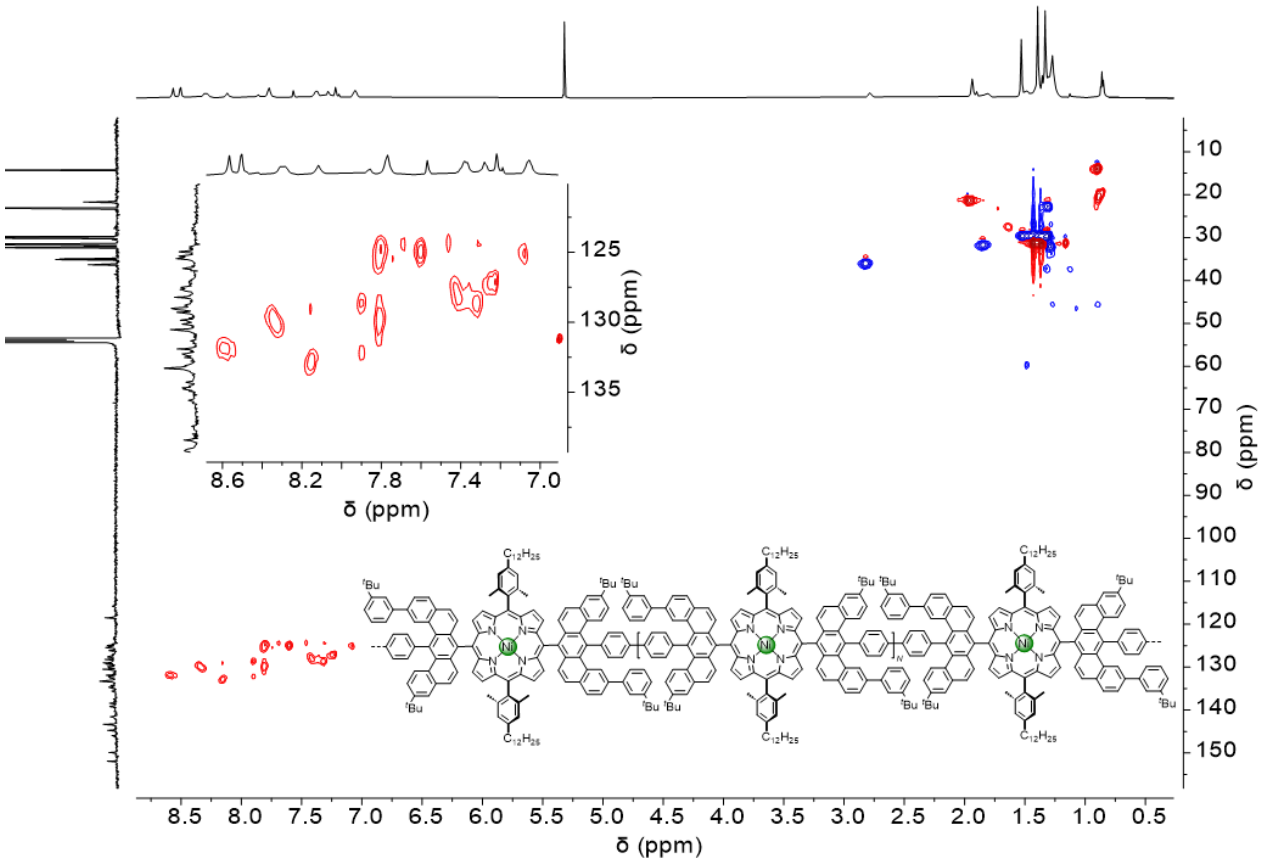

Supplementary Figure 105. $^{1}H,^{13}C$-HSQC spectrum of **PPb** (600 MHz, CD_2Cl_2, 298 K).

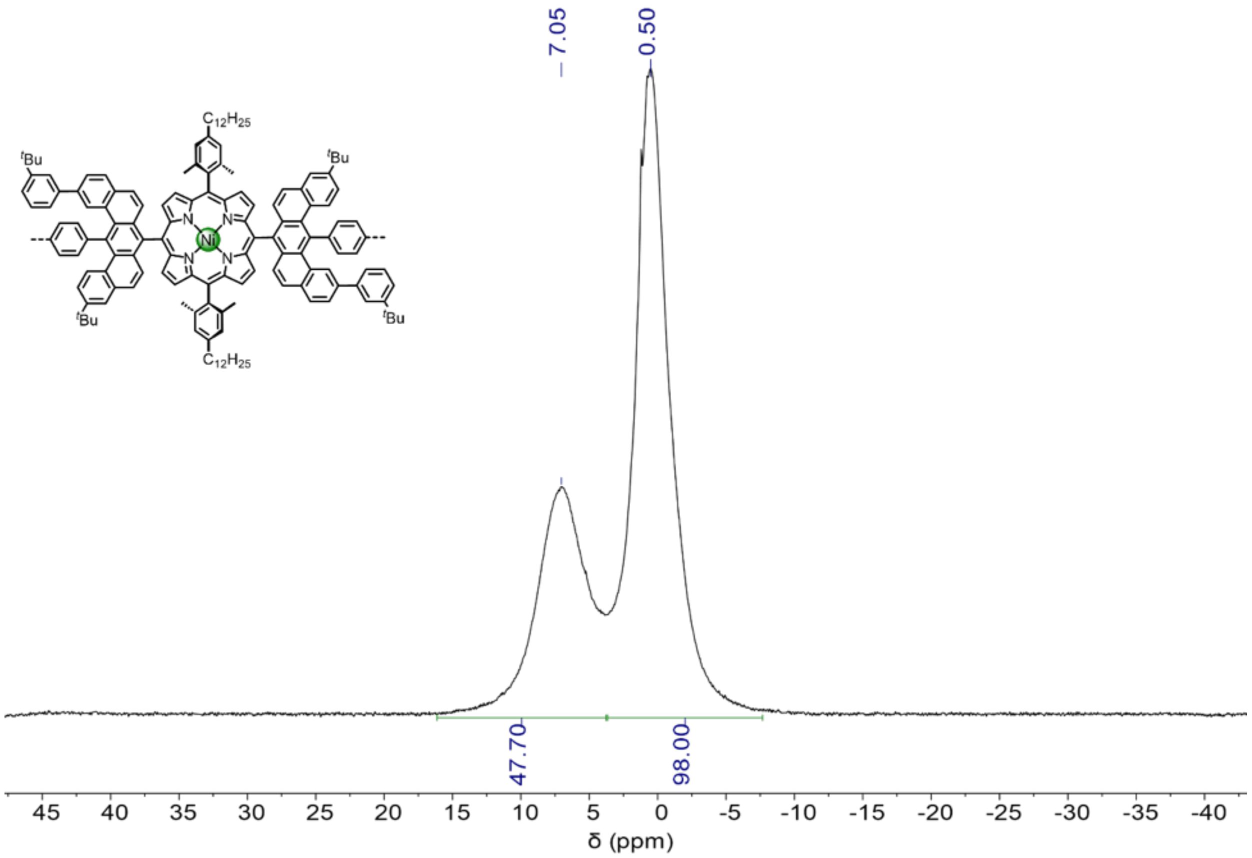

Supplementary Figure 106. Solid-state ^{1}H CP-MAS NMR spectrum of **PPb** (400 MHz, 300 K).

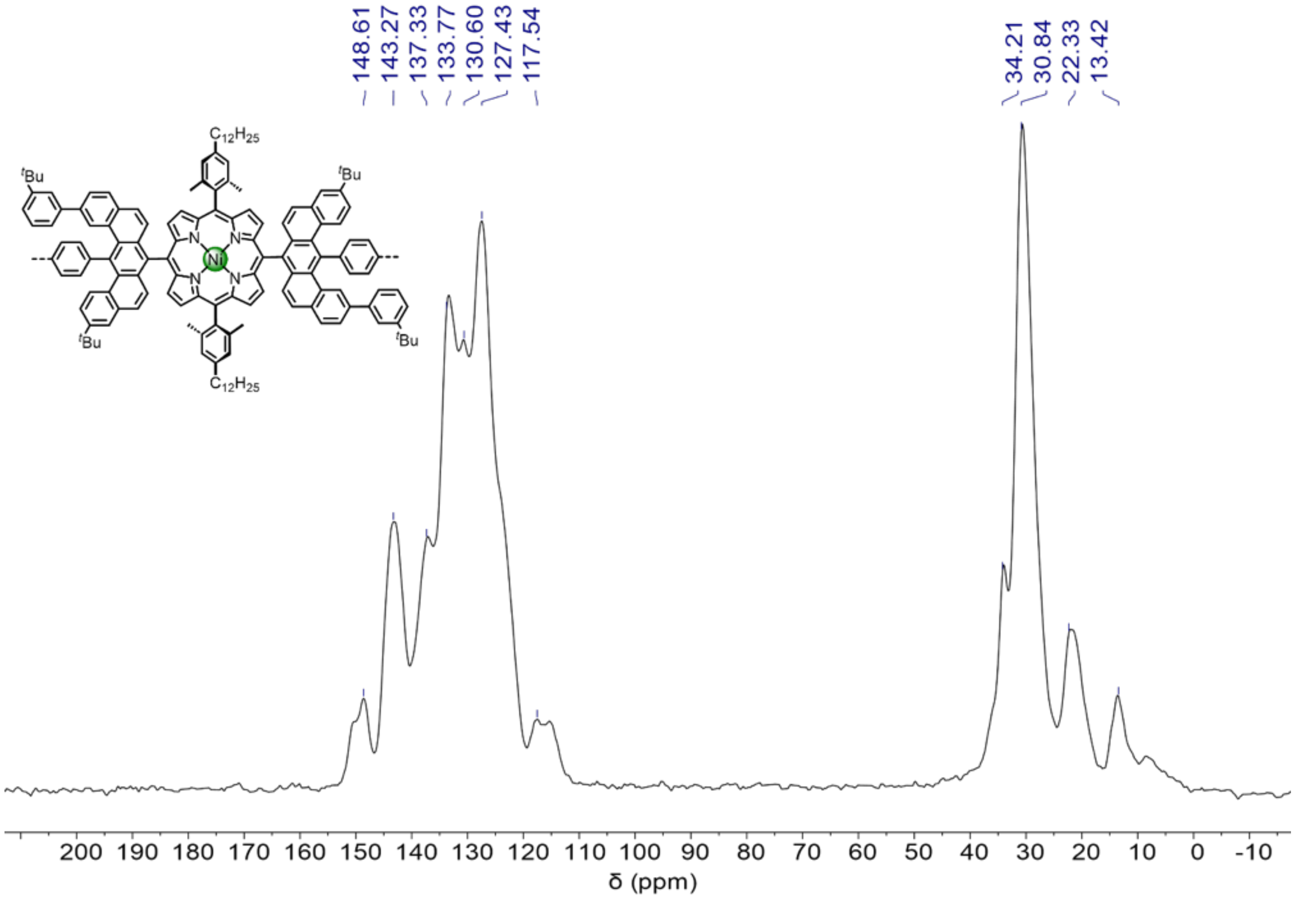

Supplementary Figure 107. Solid-state ^{13}C CP-MAS NMR spectrum of **PPb** (101 MHz, 300 K).

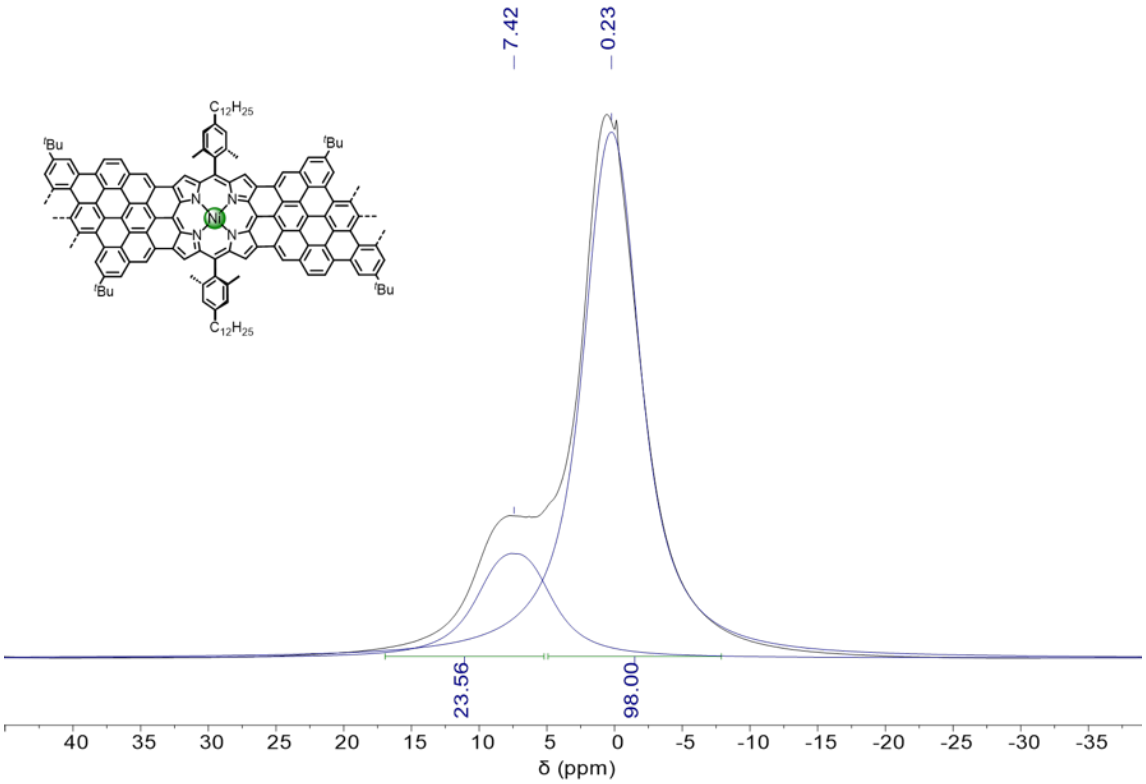

Supplementary Figure 108. Solid-state ^{1}H CP-MAS NMR spectrum of **PGNRb** (400 MHz, 300 K).

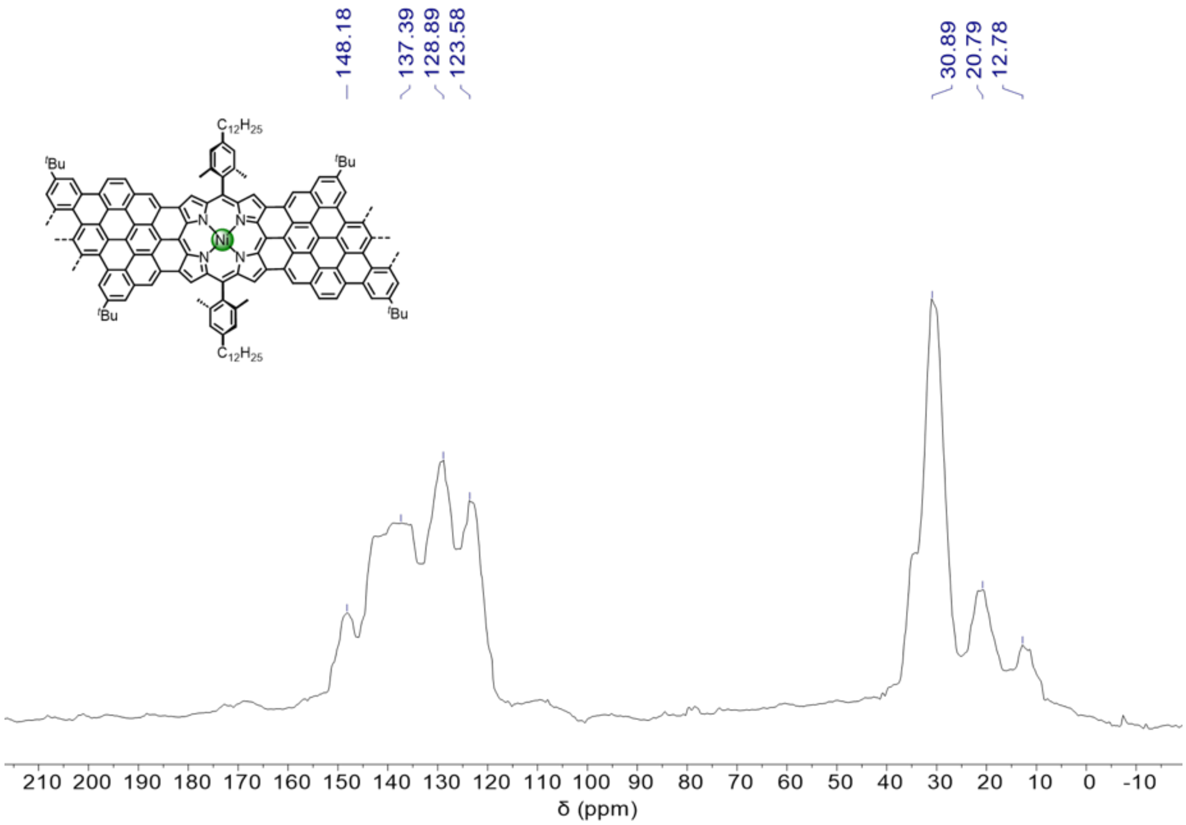

Supplementary Figure 109. Solid-state ^{13}C CP-MAS NMR spectrum of **PGNRb** (101 MHz, 300 K).

13.2 MALDI-TOF Mass Spectra

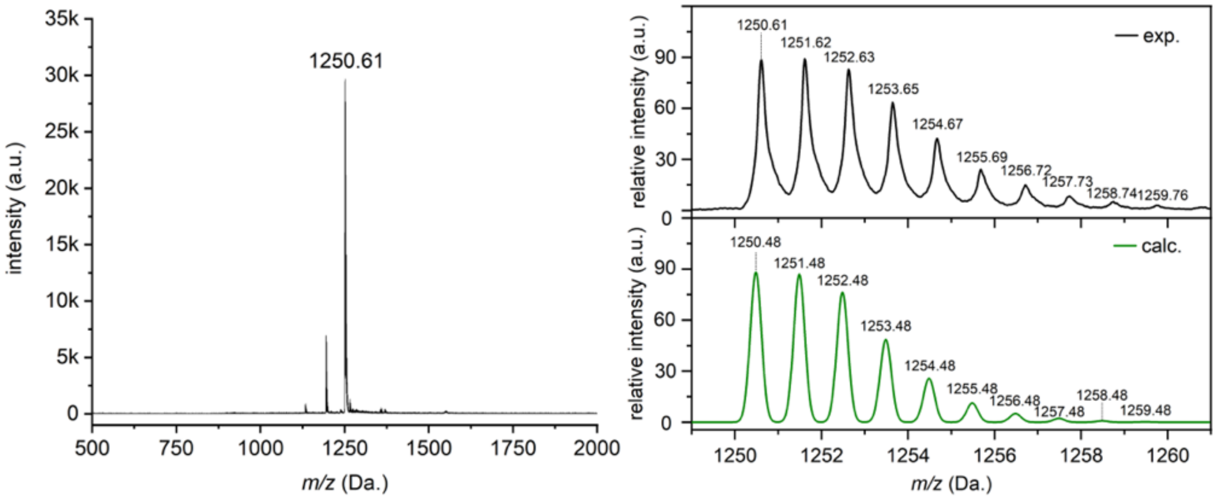

Supplementary Figure 110. MALDI-TOF MS (DCTB in tetrahydrofuran as matrix) spectrum of ***f*-P1Ng1a**, right: comparison of experimental isotropic distribution pattern with simulation. mMass 5.5.0 software was used to simulate theoretical mass spectra.[37-39]

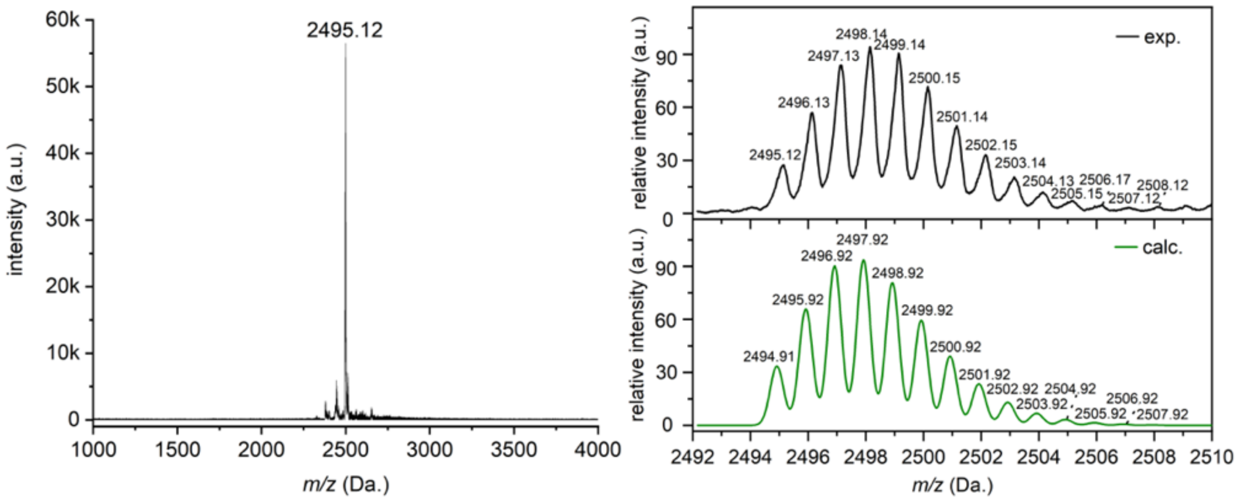

Supplementary Figure 111. MALDI-TOF MS (DCTB in tetrahydrofuran as matrix) spectrum of ***f*-P2Ng1a**, right: comparison of experimental isotropic distribution pattern with simulation. mMass 5.5.0 software was used to simulate theoretical mass spectra.[37-39]

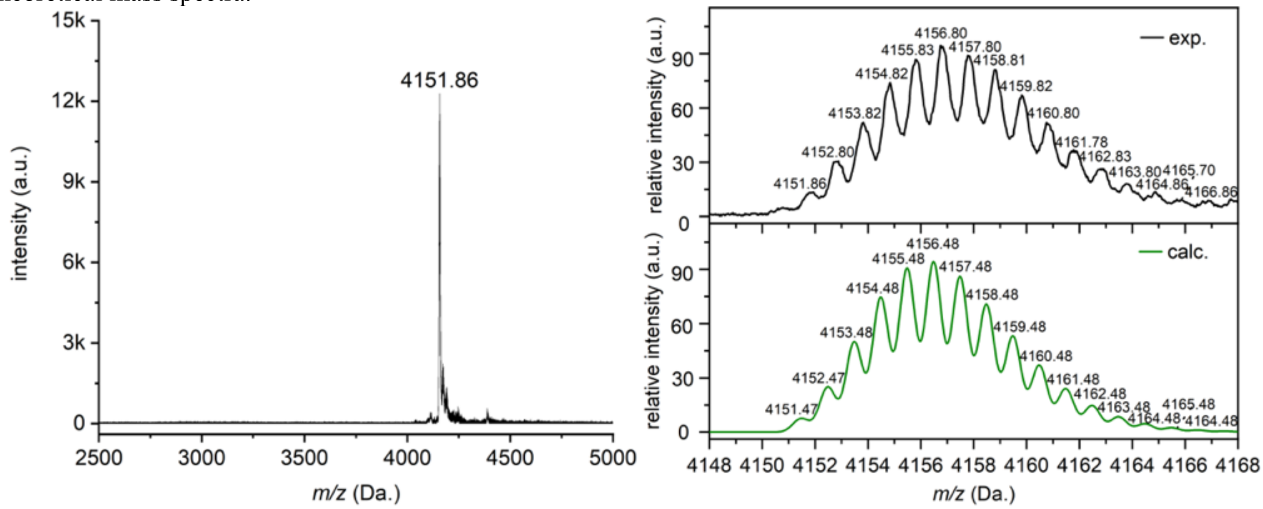

Supplementary Figure 112. MALDI-TOF MS (DCTB in tetrahydrofuran as matrix) spectrum of ***f*-P3Ng2a**, right: comparison of experimental isotropic distribution pattern with simulation. mMass 5.5.0 software was used to simulate theoretical mass spectra.[37-39]

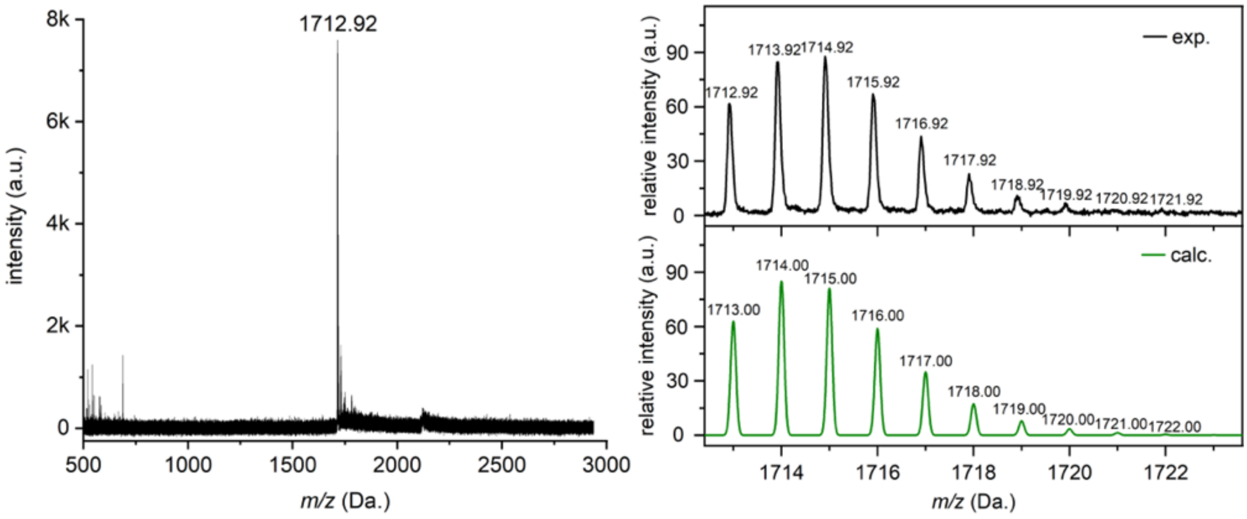

Supplementary Figure 113. MALDI-TOF MS (DCTB in tetrahydrofuran as matrix) spectrum of ***f*-P1Ng1b**, right: comparison of experimental isotropic distribution pattern with simulation. mMass 5.5.0 software was used to simulate theoretical mass spectra.[37-39]

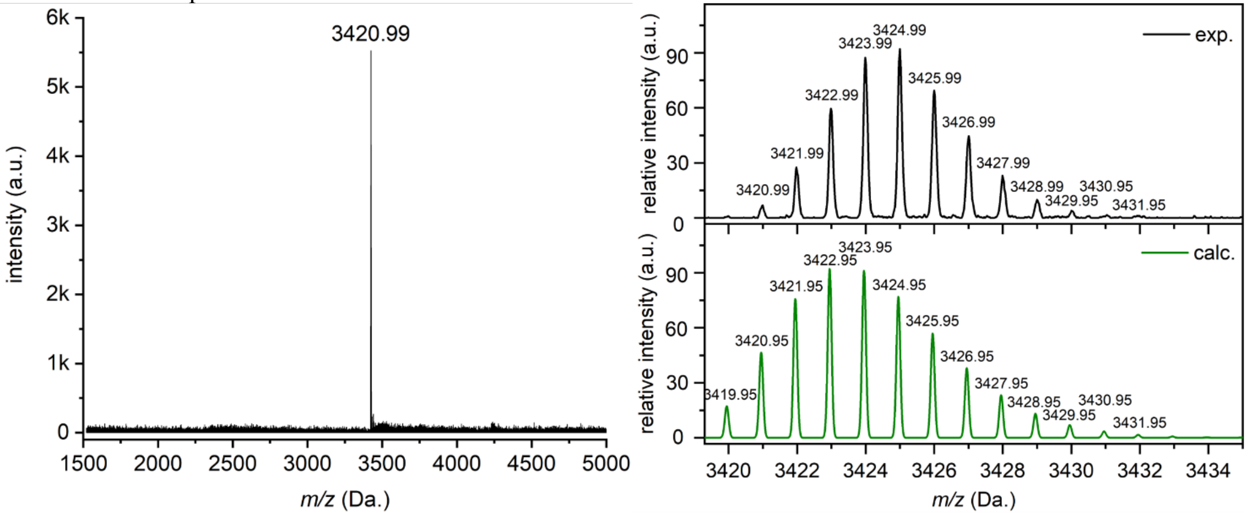

Supplementary Figure 114. MALDI-TOF MS (DCTB in tetrahydrofuran as matrix) spectrum of ***f*-P2Ng1b**, right: comparison of experimental isotropic distribution pattern with simulation. mMass 5.5.0 software was used to simulate theoretical mass spectra.[37-39]

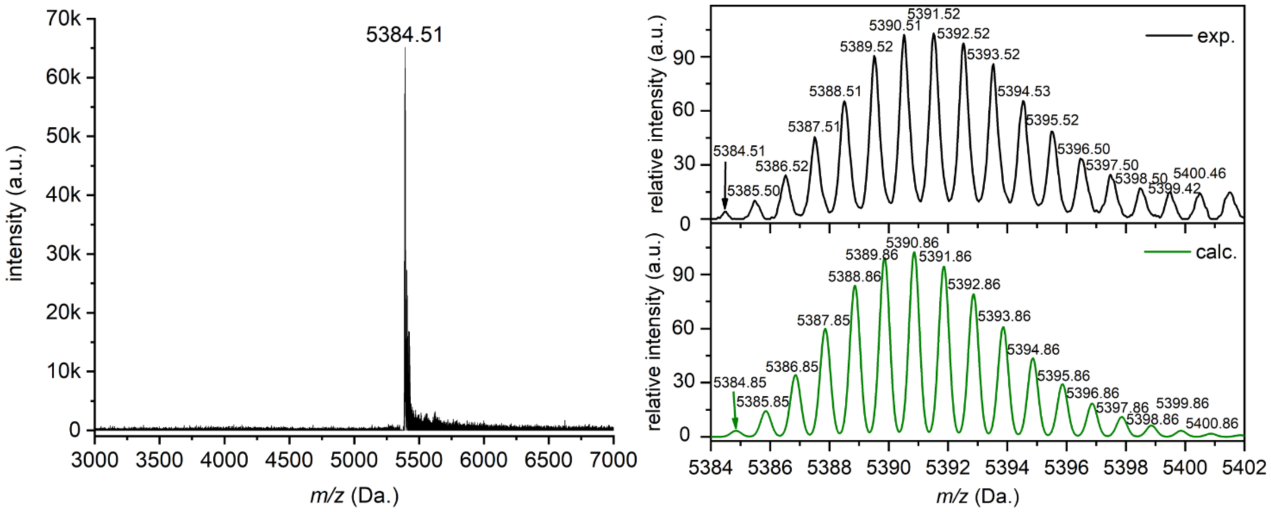

Supplementary Figure 115. MALDI-TOF MS (DCTB in tetrahydrofuran as matrix) spectrum of ***f*-P3Ng2b**, right: comparison of experimental isotropic distribution pattern with simulation. mMass 5.5.0 software was used to simulate theoretical mass spectra.[37-39]

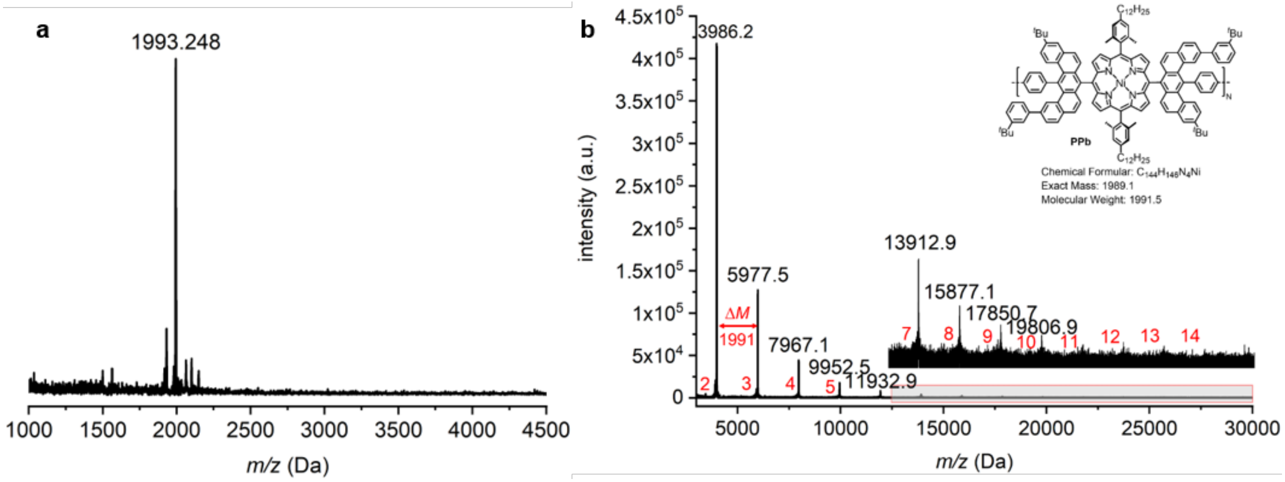

Supplementary Figure 116. a, MALDI-TOF MS (DCTB in tetrahydrofuran as matrix) spectrum of isolated monomer after Yamamoto polymerization of **2b** in tetrahydrofuran, showing clean protodechlorination. **b**, Reflection-mode MALDI-TOF MS spectrum of **PPb** measured in the low molecular weight region (DCTB in tetrahydrofuran as matrix).

14. Supplementary References

1. Peersen, O. B., Wu, X. L., Kustanovich, I. & Smith, S. O. Variable-amplitude cross-polarization MAS NMR. *J. Magn. Reson., Ser. A* **104**, 334-339 (1993).
2. Cory, D. G. & Ritchey, W. M. Suppression of signals from the probe in bloch decay spectra. *J. Magn. Reson.* **80**, 128-132 (1988).
3. Feike, M., *et al.* Broadband multiple-quantum NMR spectroscopy. *J. Magn. Reson., Ser. A* **122**, 214-221 (1996).
4. Yang, X., *et al.* Two-dimensional graphene nanoribbons. *J. Am. Chem. Soc.* **130**, 4216-4217 (2008).
5. Talirz, L., *et al.* On-surface synthesis and characterization of 9-atom wide armchair graphene nanoribbons. *ACS Nano* **11**, 1380-1388 (2017).
6. Yang, X., Dou, X. & Mullen, K. Efficient synthesis of symmetrically and unsymmetrically substituted hexaphenylbenzene analogues by Suzuki-Miyaura coupling reactions. *Chem. Asian J.* **3**, 759-766 (2008).
7. Chen, Q., *et al.* Synthesis of triply fused porphyrin-nanographene conjugates. *Angew. Chem. Int. Ed.* **57**, 11233-11237 (2018).
8. Nakae, T., *et al.* Effective synthesis of diiodinated picene and dibenzo[*a*,*h*]anthracene by AuCl-catalyzed double cyclization. *Tetrahedron Lett.* **53**, 1617-1619 (2012).
9. Sheldrick, G. M. SHELXT– Integrated space-group and crystal-structure determination. *Acta Crystallogr. A: Found. Adv.* **71**, 3-8 (2015).
10. Dolomanov, O. V., Bourhis, L. J., Gildea, R. J., Howard, J. A. K. & Puschmann, H. OLEX2: a complete structure solution, refinement and analysis program. *J. Appl. Crystallogr.* **42**, 339-341 (2009).
11. Sheldrick, G. M. Crystal structure refinement with SHELXL. *Acta Crystallogr. C Struct. Chem.* **71**, 3-8 (2015).
12. Spek, A. L. PLATON SQUEEZE: a tool for the calculation of the disordered solvent contribution to the calculated structure factors. *Acta Crystallogr. C Struct. Chem.* **71**, 9-18 (2015).
13. Brown, S. P. & Spiess, H. W. Advanced solid-state NMR methods for the elucidation of structure and dynamics of molecular, macromolecular, and supramolecular systems. *Chem. Rev.* **101**, 4125-4156 (2001).
14. Biesinger, M. C. Accessing the robustness of adventitious carbon for charge referencing (correction) purposes in XPS analysis: Insights from a multi-user facility data review. *Appl. Surf. Sci.* **597**, (2022).
15. Frisch, M. J.; Trucks, G. W.; Schlegel, H. B.; Scuseria, G. E.; Robb, M. A.; Cheeseman, J. R.; Scalmani, G.; Barone, V.; Petersson, G. A.; Nakatsuji, H.; Li, X.; Caricato, M.; Marenich, A. V.; Bloino, J.; Janesko, B. G.; Gomperts, R.; Mennucci, B.; Hratchian, H. P.; Ortiz, J. V.; Izmaylov, A. F.; Sonnenberg, J. L.; Williams-Young, D.; Ding, F.; Lipparini, F.; Egidi, F.; Goings, J.; Peng, B.; Petrone, A.; Henderson, T.; Ranasinghe, D.; Zakrzewski, V. G.; Gao, J.; Rega, N.; Zheng, G.; Liang, W.; Hada, M.; Ehara, M.; Toyota, K.; Fukuda, R.; Hasegawa, J.; Ishida, M.; Nakajima, T.; Honda, Y.; Kitao, O.; Nakai, H.; Vreven, T.; Throssell, K.; Montgomery, J. A., Jr.; Peralta, J. E.; Ogliaro, F.; Bearpark, M. J.; Heyd, J. J.; Brothers, E. N.; Kudin, K. N.; Staroverov, V. N.; Keith, T. A.; Kobayashi, R.; Normand, J.; Raghavachari, K.; Rendell, A. P.; Burant, J. C.; Iyengar, S. S.; Tomasi, J.; Cossi, M.; Millam, J. M.; Klene, M.; Adamo, C.; Cammi, R.; Ochterski, J. W.; Martin, R. L.; Morokuma, K.; Farkas, O.; Foresman, J. B.; Fox, D. J. Gaussian 16, Revision A.03. Wallingford CT: Gaussian, Inc.; 2016.
16. Soler, J. M., *et al.* The SIESTA method for *ab initio* order-*N* materials simulation. *J. Phys. Condens. Matter.* **14**, 2745-2779 (2002).
17. Perdew, J. P., Burke, K. & Ernzerhof, M. Generalized Gradient Approximation Made Simple. *Phys. Rev. Lett.* **77**, 3865-3868 (1996).

18. Narita, A., *et al.* Synthesis of structurally well-defined and liquid-phase-processable graphene nanoribbons. *Nat. Chem.* **6**, 126-132 (2014).
19. Zhang, H., *et al.* Highly mobile large polarons in black phase $CsPbI_3$. *ACS Energy Lett.* **6**, 568-573 (2021).
20. Zhang, H., *et al.* Highly mobile hot holes in $Cs_2AgBiBr_6$ double perovskite. *Sci. Adv.* **7**, eabj9066 (2021).
21. Hendry, E., *et al.* Interchain effects in the ultrafast photophysics of a semiconducting polymer:THztime-domain spectroscopy of thin films and isolated chains in solution. *Phys. Rev. B* **71**, (2005).
22. Limburg, B., *et al.* Anchor groups for graphene-porphyrin single-molecule transistors. *Adv. Funct. Mater.* **28**, (2018).
23. Mol, J. A., *et al.* Graphene-porphyrin single-molecule transistors. *Nanoscale* **7**, 13181-13185 (2015).
24. Zhang, Y. & Straub, J. E. Direct evidence for mode-specific vibrational energy relaxation from quantum time-dependent perturbation theory. III. The nu(4) and nu(7) modes of nonplanar nickel porphyrin models. *J. Chem. Phys.* **130**, 215101 (2009).
25. Piffat, C., Melamed, D. & Spiro, T. G. Ruffling effects on porphyrin vibrations: normal-mode analysis for nickel octaethyltetraphenylporphine from resonance Raman and IR spectra of isotopomers. *J. Phys. Chem.* **97**, 7441-7450 (2002).
26. Gehring, P., *et al.* Distinguishing lead and molecule states in graphene-based single-electron transistors. *ACS Nano* **11**, 5325-5331 (2017).
27. Robertson, J. High dielectric constant oxides. *EPJ Appl. Phys.* **28**, 265-291 (2004).
28. Laskar, J., *et al.* Gate-controlled negative differential resistance in drain current characteristics of AlGaAs/InGaAs/GaAs pseudomorphic MODFETs. *IEEE Electron Device Lett.* **10**, 528-530 (1989).
29. Dragoman, D. & Dragoman, M. Terahertz oscillations in semiconducting carbon nanotube resonant-tunneling diodes. *Physica E Low Dimens. Syst. Nanostruct.* **24**, 282-289 (2004).
30. Haddad, G. I. & Mazumder, P. Tunneling devices and applications in high functionality/speed digital circuits. *Solid State Electron.* **41**, 1515-1524 (1997).
31. Weitz, R. T., *et al.* High-performance carbon nanotube field effect transistors with a thin gate dielectric based on a self-assembled monolayer. *Nano Lett.* **7**, 22-27 (2007).
32. Bishop, M. D., *et al.* Fabrication of carbon nanotube field-effect transistors in commercial silicon manufacturing facilities. *Nat. Electron.* **3**, 492-501 (2020).
33. Dürkop, T., Getty, S. A., Cobas, E. & Fuhrer, M. S. Extraordinary mobility in semiconducting carbon nanotubes. *Nano Lett.* **4**, 35-39 (2003).
34. Zaumseil, J. & Sirringhaus, H. Electron and ambipolar transport in organic field-effect transistors. *Chem. Rev.* **107**, 1296-1323 (2007).
35. Geng, Z., *et al.* Graphene Nanoribbons for Electronic Devices. *Ann. Phys. (Berl.)* **529**, (2017).
36. Fang, T., Konar, A., Xing, H. & Jena, D. Mobility in semiconducting graphene nanoribbons: Phonon, impurity, and edge roughness scattering. *Phys. Rev. B* **78**, (2008).
37. Niedermeyer, T. H. & Strohalm, M. mMass as a software tool for the annotation of cyclic peptide tandem mass spectra. *PLoS One* **7**, e44913 (2012).
38. Strohalm, M., Kavan, D., Novak, P., Volny, M. & Havlicek, V. mMass 3: a cross-platform software environment for precise analysis of mass spectrometric data. *Anal. Chem.* **82**, 4648-4651 (2010).
39. Strohalm, M., Hassman, M., Kosata, B. & Kodicek, M. mMass data miner: an open source alternative for mass spectrometric data analysis. *Rapid Commun. Mass Spectrom.* **22**, 905-908 (2008).
